# Cobalt-catalyzed C–H cyanations: Insights into the reaction mechanism and the role of London dispersion

**DOI:** 10.3762/bjoc.14.130

**Published:** 2018-06-25

**Authors:** Eric Detmar, Valentin Müller, Daniel Zell, Lutz Ackermann, Martin Breugst

**Affiliations:** 1 Department für Chemie, Universität zu Köln, Greinstraße 4, 50939 Köln, Germany; 2 Institut für Organische und Biomolekulare Chemie, Georg-August-Universität Göttingen, Tammannstraße 2, 37077 Göttingen, Germany

**Keywords:** catalysis, C–H activation, density functional theory, London dispersion, reaction mechanisms

## Abstract

Carboxylate-assisted cobalt(III)-catalyzed C–H cyanations are highly efficient processes for the synthesis of (hetero)aromatic nitriles. We have now analyzed the cyanation of differently substituted 2-phenylpyridines in detail computationally by density functional theory and also experimentally. Based on our investigations, we propose a plausible reaction mechanism for this transformation that is in line with the experimental observations. Additional calculations, including NCIPLOT, dispersion interaction densities, and local energy decomposition analysis, for the model cyanation of 2-phenylpyridine furthermore highlight that London dispersion is an important factor that enables this challenging C–H transformation. Nonbonding interactions between the Cp* ligand and aromatic and C–H-rich fragments of other ligands at the cobalt center significantly contribute to a stabilization of cobalt intermediates and transition states.

## Introduction

**Scheme 1:**
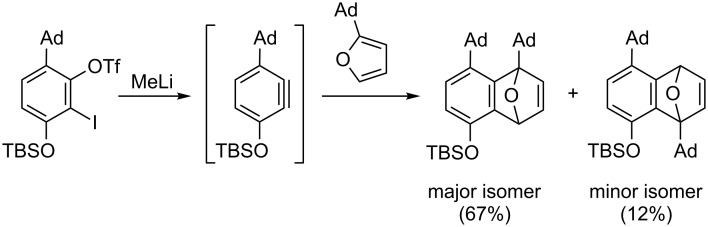
Cycloaddition reaction of in situ generated benzynes resulting in the sterically more hindered adduct (Ad = 1-adamantyl) [8].

For a long time, large and bulky substituents have intuitively been considered to act through unfavorable steric interactions, although London dispersion – the attractive part of the van-der-Waals interaction – is known for more than 100 years [1–2]. The stabilizing nature of C–H···H–C interactions and their importance for organic transformations has only been fully realized within the last decades [3]. Among others, these interactions explain the hexaarylethane riddle [4] and are responsible for the high stability of singly bonded diamondoid dimers resulting in very long C–C bonds [5–6], or very short H···H contacts in tris(3,5-di-*tert*-butylphenyl)methane [7]. Besides a remarkable effect on organic structures, dispersion can also affect the outcome of chemical transformations. Presumably due to attractive dispersive interactions between two adamantyl groups in the transition state of a [4 + 2] cycloaddition of benzynes (Scheme 1), the seemingly sterically more hindered product is formed preferentially [8].

Similar to other noncovalent interactions [9–11], London dispersion can also play a crucial role in different transition-metal-catalyzed reactions [12–17]. The C–H-rich di-1-adamantylphosphine oxide – a typical dispersion element – was experimentally found to be an excellent preligand for ruthenium- and palladium-catalyzed C–H functionalizations [18–23]. Similarly, computational studies revealed the importance of dispersion effects in palladium-catalyzed cross-coupling reactions [24–27]. For example, the contribution of London dispersion (up to 37 kcal mol^−1^) has a huge influence on the ligand dissociation process within the Pd(PPh_3_)_4_ system [25]. Furthermore, only the results obtained from dispersion-corrected density functional theory [28–29] were in agreement with the experimental observations and dispersion reduces the activation free energies by up to 30 kcal mol^–1^ [27].

**Scheme 2:**
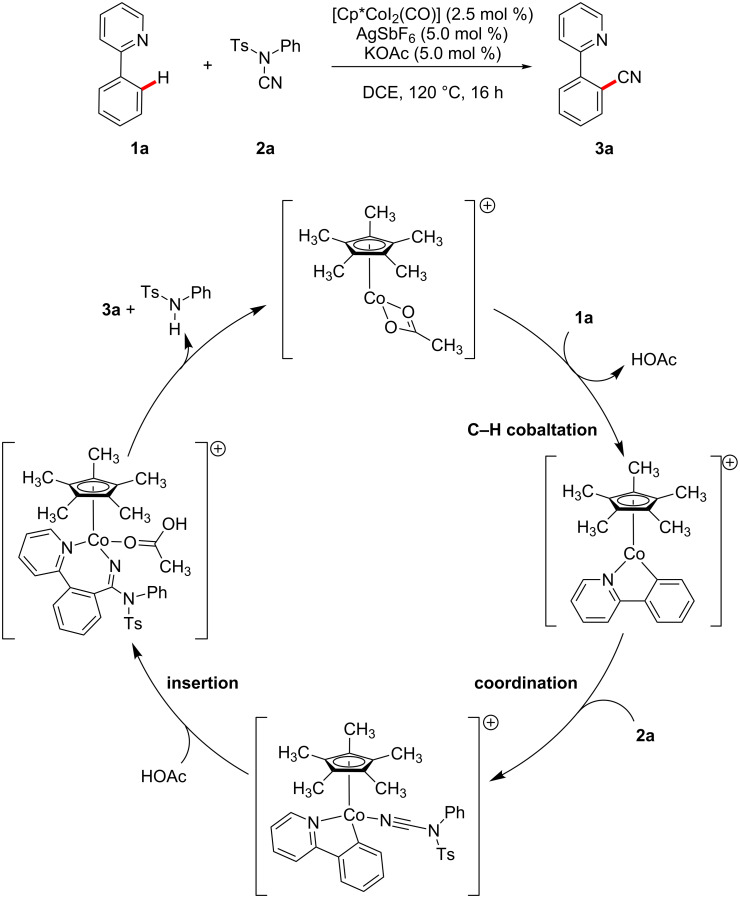
Recently developed cobalt-catalyzed C–H cyanation [30].

Currently, the strategic application of London dispersion in catalysis is still very difficult to achieve and, as a consequence, detailed insights in how dispersion influences organic reactions continue to be in high demand. Therefore, we have computationally analyzed the recently developed cobalt-catalyzed C–H cyanation of arenes (Scheme 2) [30–34]. Dispersion effects can be envisioned to be highly important in this system, as the relatively C–H-rich ligand Cp* can interact with both substrates within the cobalt complexes. In 2015, Li and Ackermann have proposed the catalytic cycle (C–H cobaltation, ligand coordination, insertion) shown in Scheme 2 which served as the starting point of this investigation [30]. We now report on our computational findings supported by novel kinetic investigations to establish the reaction mechanism of this synthetically useful C–H activation and to elucidate the role of London dispersion in these transformations.

## Results and Discussion

### Analysis of the reaction mechanism

**Figure 1:**
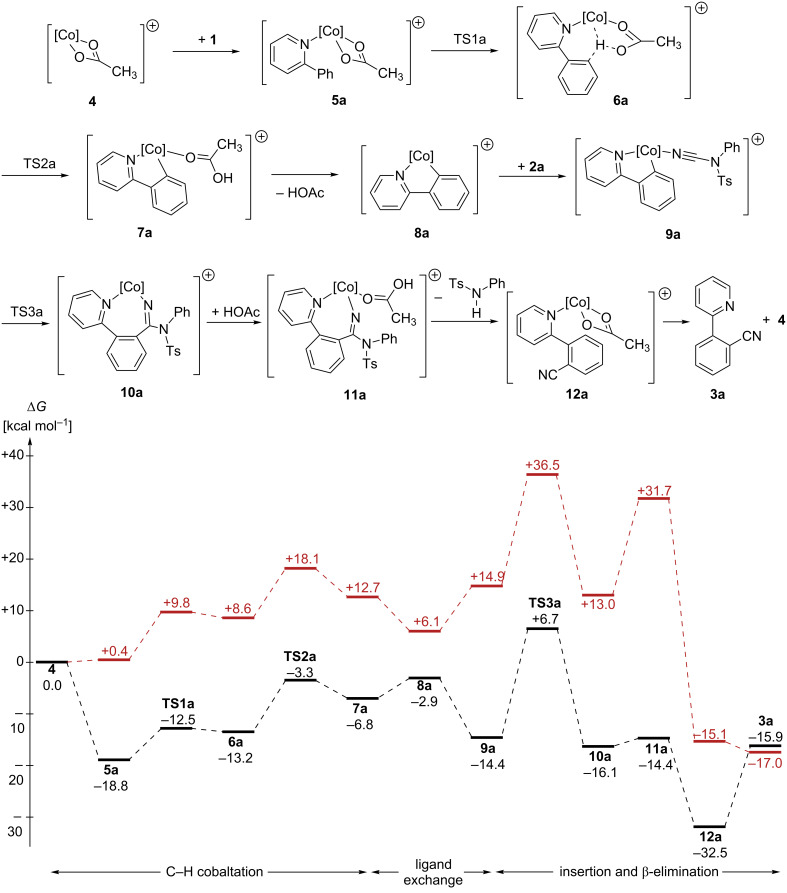
Calculated free-energy profile for the cobalt-catalyzed C–H cyanation of 2-phenylpyridine (**1a**) [in kcal mol^−1^, [Co] = Cp*Co, black lines indicate that dispersion (D3 correction with Becke–Johnson damping) [35–36] was included in the calculations while red lines indicate that dispersion was not included].

**Figure 2:**
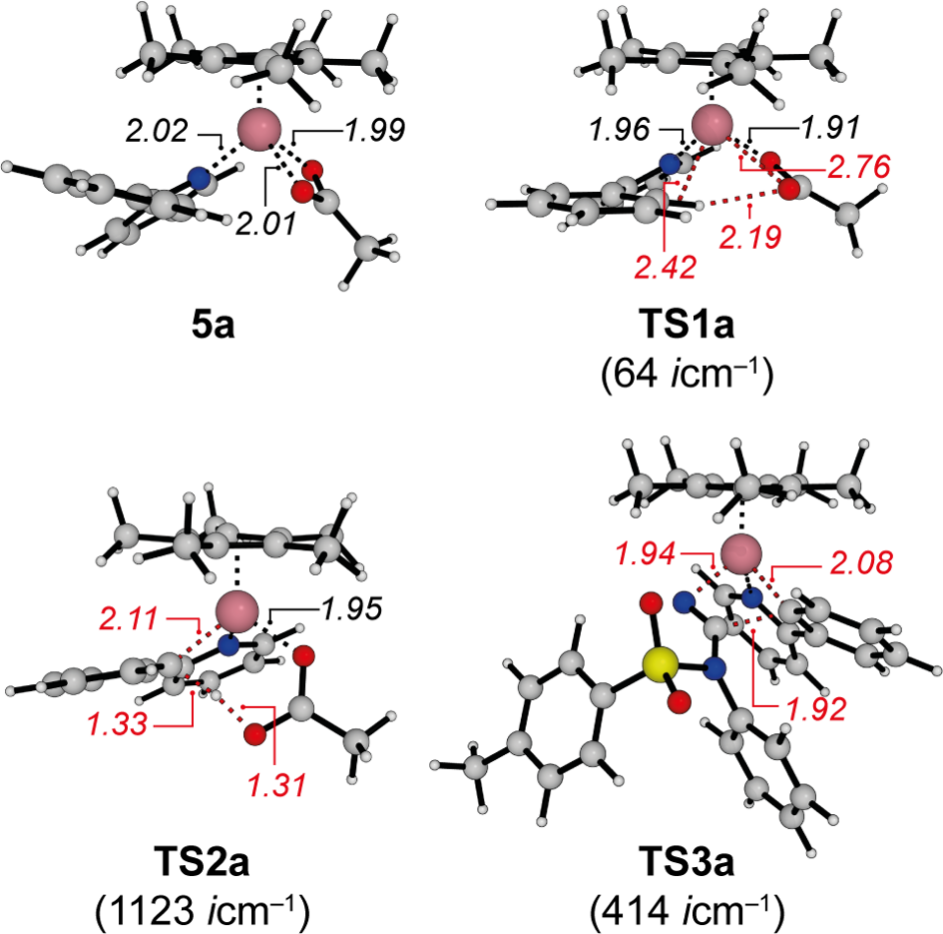
Calculated structures, selected bond lengths (in Å), and imaginary frequencies for representative intermediates and transition states for the cobalt-catalyzed C–H cyanation of 2-phenylpyridine (**1a**).

To unravel the importance of London dispersion on the cobalt-catalyzed C–H cyanation of 2-phenylpyridine (**1a**), the underlying catalyst’s mode of action has to be fully understood. The available experimental data indicated a reversible C–H metalation, which led to the suggested catalytic cycle of Scheme 2 [30]. As computational investigations also allow the study of intermediates that are too unstable to be observed under the experimental conditions, we have analyzed the underlying reaction mechanism in more detail employing density functional theory. A complete free energy profile on the B3LYP-D3BJ/def2-QZVP/COSMO//B3LYP-D3BJ/def2-TZVP potential energy surface is depicted in Figure 1 (black line), while the free-energy profile on the M06-L surface is summarized in Supporting Information File 1. Selected intermediates and transition states are shown in Figure 2.

The computational analysis starts with the catalytically active cobalt(III) acetate complex **4** which is generated in situ from the precatalyst [Cp*CoI_2_(CO)], AgSbF_6_, and KOAc. While the iodine ions are captured by Ag^+^, carbon monoxide dissociates and leaves the reaction mixture as a gas. Although the SbF_6_^–^ counter ion to the cationic cobalt complexes is considered to be weakly coordinating [37], specific interactions cannot be completely ruled out. We have assumed that all of the positively charged cobalt complexes on the reaction path are similarly affected by ion pairing and therefore, we base the following investigation mainly on the reactions of the cobalt complexes and do not include ion pairing in our analysis. Coordination of 2-phenylpyridine (**1a**) to this 16-electron species leads to the intermediate **5a** (Figure 2) in a highly exergonic reaction step (Δ*G* = −18.8 kcal mol^−1^), which also is the resting state of the catalytic cycle. This intermediate could therefore be amenable to spectroscopic characterization. Based on our computational analysis, the subsequent C–H cobaltation (**5a** → **7a**) is endergonic (ΔΔ*G* = +12 kcal mol^−1^) and proceeds in a step-wise fashion. A similar mechanism has previously been described by McMullin, Williams, and Frost [38], as well as by Ackermann [39–40] for ruthenium-catalyzed C–H alkenylations. In the first transition state (**TS1a**, Figure 2), the κ^2^-coordination of the acetate ligand changes to a κ^1^-coordination. The resulting intermediate **6a** is stabilized by an agostic interaction between the C–H bond and the metal atom as well as by an additional weak hydrogen bond between the C–H bond and the acetate oxygen (O^…^H distance 2.26 Å). A natural population analysis of structure **6a** further confirms the stabilizing nature of these interactions. In the second transition state **TS2a** (Figure 2), the C–H bond is broken and the proton is transferred to the acetate which results in the formation of the cobaltacycle **7a**.

Acetic acid dissociates, and *N*-cyano-*N*-phenyl-*p*-toluenesulfonamide (**2a**) coordinates to the 16-electron intermediate **8a** yielding **9a**. Next, the insertion of the cyanating agent **2a** into the cobalt–carbon bond takes place through **TS3a**. Within the four-membered transition state (Figure 2), the C–C bond to be formed is still rather long (C–C distance 1.92 Å), while the C–N distance is already significantly elongated (1.15 Å in **9a**, 1.22 Å in **TS3a**, 1.26 Å in **10a**). Furthermore, a significant reorganization has to take place during this step: the former almost linear N–C–N fragment (179.2°) changes to 137.9° in **TS3a** and 124.4° in **10a**, which results in a high barrier for this step. Subsequent coordination of acetic acid leads to intermediate **11a**. No transition states could be obtained for the following β-elimination and proto-demetalation resulting in product **3a**, the cobalt(III) acetate complex **4**, and *N*-phenyl-*p*-toluenesulfonamide. All attempts starting from different potential transition state structures resulted in barrierless reactions when a proton approaches the amidine substructure (→ **12a**). As the cyanated 2-phenylpyridine **3a** is less Lewis-basic compared to the starting material **1a**, **12a** could also react with **1a** in a thermodynamically favorable ligand exchange reaction (Δ*G* = −2.2 kcal mol^−1^) to yield complex **5a**.

In contrast to previous computational studies on manganese(I)-catalyzed fluoro-allylation reactions where β-fluoride and HF eliminations played an important role [41], similar reactions involving amine eliminations seem to be not relevant in this reaction. Furthermore, a comparison with previous computational investigations on copper-catalyzed *ortho* C–H cyanations of vinylarenes revealed that those reactions take place via a completely different mechanism involving two distinct catalytic cycles (copper-catalyzed electrophilic cyanative dearomatization and base-catalyzed hydrogen transposition) [42–43].

**Scheme 3:**
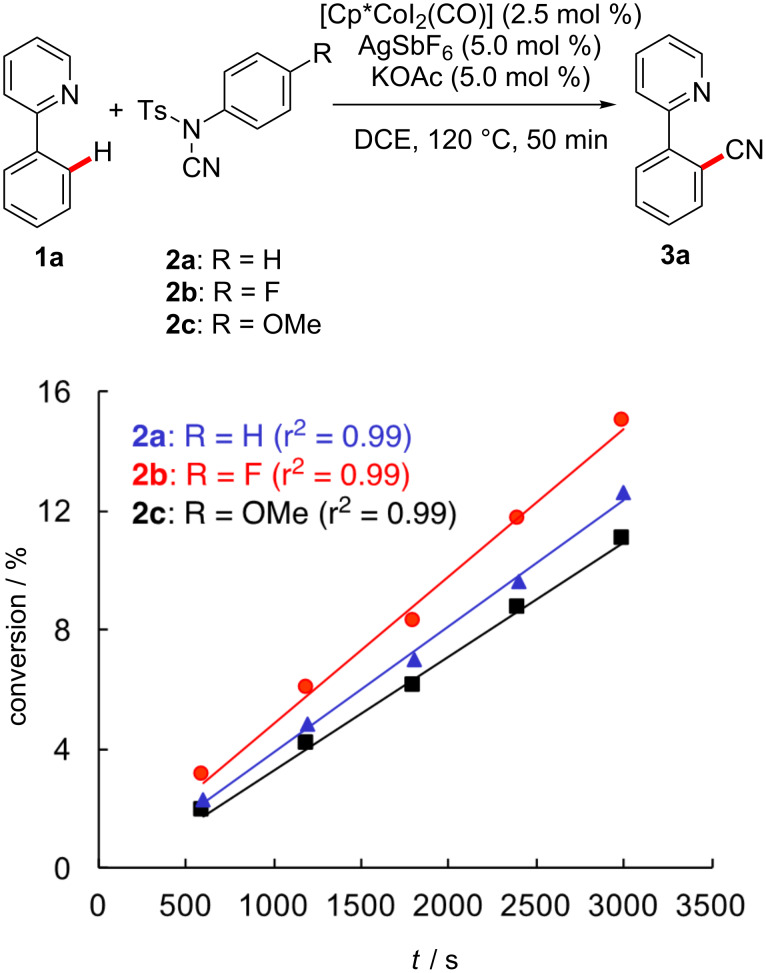
Kinetic profile of the cobalt-catalyzed C–H cyanation with differently substituted cyanating agents **2**.

Inspired by this computational analysis, we experimentally probed the effect of differently substituted cyanation agents **2** on the kinetics of the cobalt(III)-catalyzed C–H cyanation (Scheme 3). Thus, we observed that electron-withdrawing groups significantly facilitated the desired transformation. As the calculated rate-limiting transition state **TS3** benefits from a stabilization of the developing negative charge on the sulfonamide, the relative rates of Scheme 3 provide further support for the migratory insertion representing the rate determining step [44].

As differently substituted 2-phenylpyridines **1** have been employed experimentally, we included five representative substrates (R = H, CH_3_, F, C(O)CH_3_, CN) into the computational analysis as well. For these calculations, only one functional (B3LYP-D3BJ) and a smaller basis set (def2-SVP for non-metals and def2-TZVP for Co) were employed during the optimization to reduce the computational cost. These results are summarized in Table 1.

**Table 1:** Calculated free energies for the reaction mechanism involving differently substituted 2-phenylpyridines **1a–e** [B3LYP-D3BJ/def2-QZVP/COSMO//B3LYP-D3BJ/def2-SVP, def2-TZVP for Co].

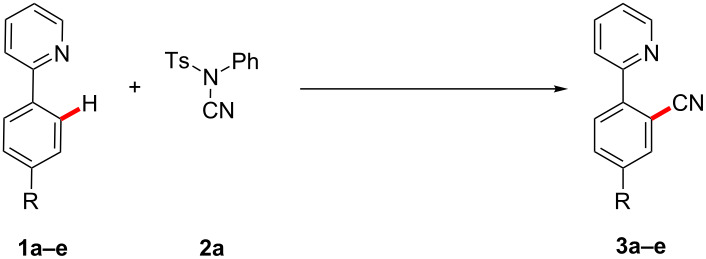													

	**4**	**5**	**TS1**	**6**	**TS2**	**7**	**8**	**9**	**TS3**	**10**	**11**	**12**	**3**

R = H (**1a**)	0.0	−20.1	−12.4	−14.1	−5.4	−8.4	−4.2	−15.5	+3.4	−18.3	−16.0	−34.5	−18.0
R = CH_3_ (**1b**)	0.0	−21.0	−14.2	−15.7	−7.1	−9.6	−4.5	−18.5	+0.6	−18.8	−16.1	−35.5	−18.9
R = F (**1c**)	0.0	−19.8	−12.0	−12.4	−4.8	−9.7	−4.4	−17.5	+4.5	−17.3	−14.9	−32.3	−16.8
R = C(O)CH_3_ (**1d**)	0.0	−18.1	−9.6	−11.2	−3.4	−7.4	−2.1	−17.7	+3.4	−17.5	−13.2	−30.9	−16.4
R = CN (**1e**)	0.0	−18.0	−9.9	−9.9	−3.1	−8.9	−3.5	−16.8	+5.4	−14.9	−12.4	−29.4	−15.5

For the unsubstituted 2-phenylpyridine (**1a)**, both computational methods (Figure 1 and Table 1) and the optimized structures are generally rather similar to one another. Based on the computational analysis depicted in Table 1, the turnover-limiting step for all substrates **1** is represented by the insertion of the cyanating agent **2a** into the cobalt–carbon bond, which can also be concluded based on the kinetic data of Scheme 3.

Based on the computational analysis of Figure 1 and the experimental data depicted in Scheme 2, the turnover-limiting step for this transformation is the insertion of **2a** with an overall barrier of 25.5 kcal mol^−1^. The initial C–H cobaltation occurs with a smaller activation free energy of 15.5 kcal mol^−1^. These values are also in good qualitative agreement with the experimental findings: The calculated high barriers match the prolonged reaction times and high temperature required in the experimental studies and the reversible C–H metalation [30].

### Influence of London dispersion

In recent years, London dispersion, the attractive part of the van-der-Waals force, has been repeatedly identified as key to stabilizing organic structures and facilitating novel reactivities [3]. As the Cp* ligand is a C–H-rich molecule, we envisioned that dispersive interactions should be important for this transformation as well. As a consequence, we have analyzed this reaction additionally with B3LYP without dispersion correction and the dispersion-corrected M06-L functional under otherwise identical conditions as a first starting point. Independent of the computational method, the overall reaction free energy for the transformation of Scheme 2 is almost identical [−15.9 (B3LYP-D3BJ), −17.0 (B3LYP), and −15.9 (M06-L) kcal mol^−1^] indicating that dispersion is less important for the overall thermodynamics of this reaction. In contrast, a strong effect of the functional was observed for the complete energy profile. While the dispersion-corrected functional M06-L (see the Supporting Information File 1 for details) resulted in a comparable profile to that obtained with B3LYP-D3BJ (black lines in Figure 1), a significant deviation was observed when the latter was used without any dispersion correction (red lines in Figure 1). All cobalt complexes are substantially stabilized by dispersive interactions resulting in a significant net reduction of the activation free energy by 11 kcal mol^−1^. Comparable contributions of London dispersion have also been calculated with other functionals (TPSS [45] and PBE [46–47]). As expected, complexes with more nonbonding contacts (e.g., **10a**) are better stabilized than complexes where the Cp* ligand is located farther away from other ligands (e.g., **8a**). In comparison to computational investigations of Pd-catalyzed reactions [27], similar dispersive stabilizations of individual complexes have been calculated here.

**Figure 3:**
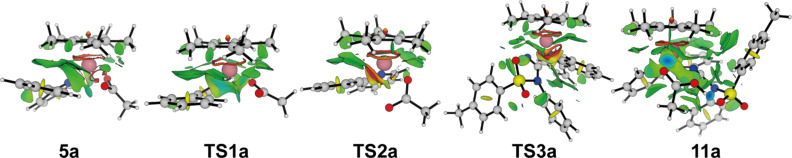
Noncovalent interaction (NCI) analysis for selected intermediates and transition states. The gradient isosurfaces (*s* = 0.5 au) are colored according to the sign of (λ_2_)ρ over the range of −0.05 (blue) to +0.05 (red).

A closer qualitative analysis of the intramolecular interactions in these complexes employing the NCIPLOT program [48–49] furthermore confirms these noncovalent interactions. While all plots are shown in Supporting Information File 1, Figure 3 summarizes those for selected intermediates and transition states. For all structures, significant interactions can be found between the Cp* ligand and the various phenyl groups of the reagents. In addition, the presence of additional stabilizing interactions such as further hydrogen bonds can also be confirmed by this analysis (e.g., in **TS3**, see also the Supporting Information File 1).

**Figure 4:**
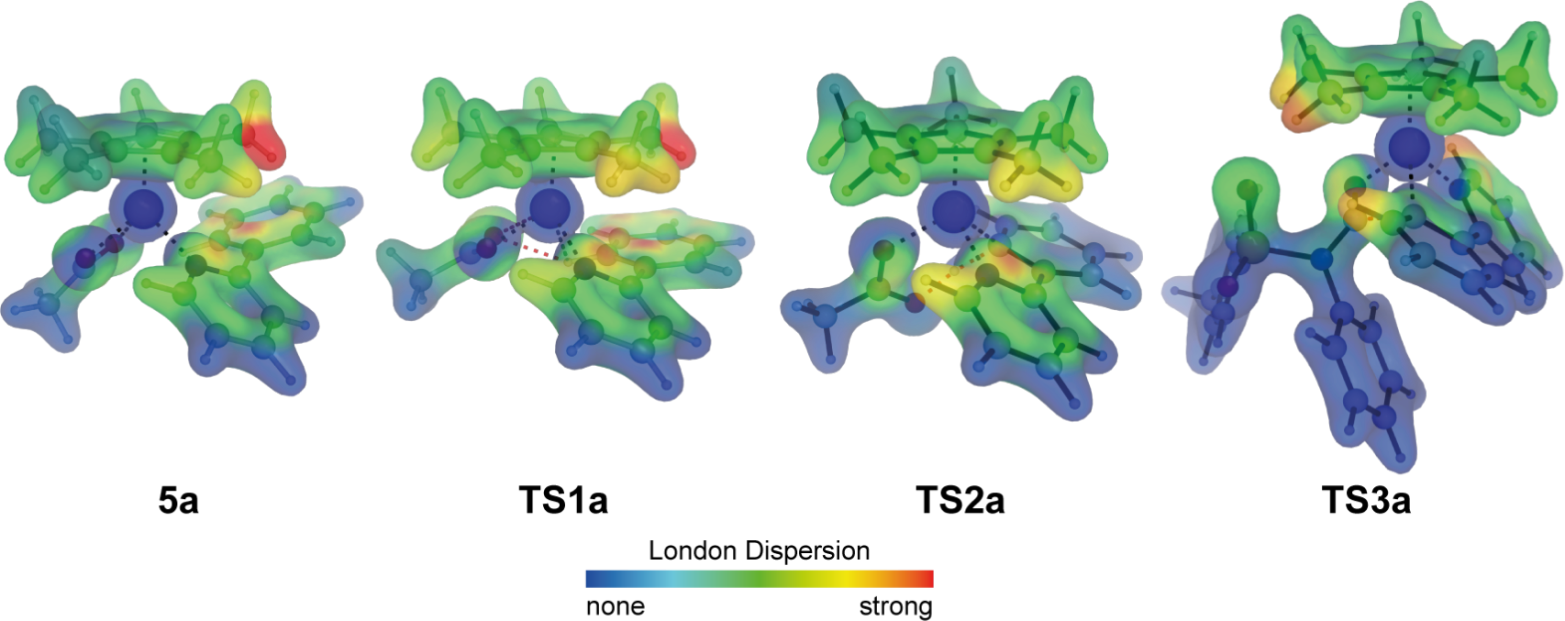
Projected dispersion interaction density (DID) plots for selected intermediates and transition states. The molecular density isosurfaces (0.1 *e*/Bohr^3^) are colored from zero interaction energy (blue) to the strongest dispersion interaction (red).

To further probe the dispersive interaction of the Cp* ligand and the other ligands, we have additionally calculated the dispersion interaction densities (DID) [50] for all intermediates and transition states at the SCS-LMP2/def2-TZVPP level of theory. The DID plots of Figure 4 reveal that medium to strong dispersive interactions can be found between the Cp* ligand and the aromatic and C–H-rich fragments in its proximity. In line with the analyses presented in Figure 3 and Figure 4, a local energy decomposition (LED) analysis [51] using DLPNO-CCSD(T)/cc-pVDZ also confirmed medium to strong dispersive interactions up to 12 kcal mol^−1^ between the Cp* ligand and the other ligands. Based on the computational analysis, London dispersion is not only highly beneficial for the synthetically important cobalt-catalyzed C−H cyanation reaction, but it also emphasises that the Cp* ligand does not exclusively act as a sterically demanding ligand in transition-metal-catalyzed reactions.

## Conclusion

We have analyzed the cobalt(III)-catalyzed C–H cyanation of differently substituted 2-phenylpyridines with *N*-cyano-*N*-aryl-*p*-toluenesulfonamide using density functional theory. On the basis of our computational and experimental data, we can propose a reaction mechanism for this transformation. After an initial and reversible C–H cobaltation, the subsequent insertion of the cyanating agents is the rate-limiting step. In addition, our calculations unravel that all the cobalt intermediates are considerably affected by London dispersion, which also results in a significant stabilization of the rate-limiting transition state.

## Computational Details

For all structures, geometry optimizations were performed with three different functionals using the def2-TZVP (def2-TZVPP for M06-L) basis set [52] and the m4 numerical quadrature grid in the gas phase. The hybrid functional B3LYP [53–54] with and without Grimme’s dispersion correction D3 (Becke–Johnson damping) [35–36] as well as Truhlar’s dispersion-corrected M06-L [55] functional were employed in this investigation. For the latter, the density fitting RI-J approach was used to accelerate the calculations [56–57]. For the analysis of the substituent effect, the B3LYP functional with Grimme’s dispersion correction D3 (Becke–Johnson damping) was employed together with the def2-SVP basis set for all non-metals and the def2-TZVP basis set for Co. Vibrational analysis verified that each structure was a minimum or transition state (*i*ω < 30 cm^−1^ were tolerated). Thermal corrections were calculated from unscaled harmonic vibrational frequencies at the same levels of theory and refer to a standard state of 298.15 K and 1 mol L^−1^. Entropic contributions to the reported free energies were obtained from partition functions evaluated with Truhlar’s quasi-harmonic approximation [58]. This method uses the same approximations as the usual harmonic oscillator approximation except that all vibrational frequencies lower than 100 cm^−1^ are set equal to 100 cm^−1^. Energies were subsequently derived from single-point calculations employing the functionals described above, the quadruple-ζ basis set def2-QZVP [52] and the COSMO solvation model [59] for dichloroethane (ε = 10.125). The dispersion interaction densities (DID) [50] were calculated at the SCS-LMP2/def2-TZVPP level of theory using MOLPRO 2015 [60–61]. The local energy decomposition analysis [51] was performed employing Neese’s domain-based local pair-natural orbital (DLPNO) approach to the CCSD(T) method [DLPNO-CCSD(T)] [62–64] with tightPNO settings and the double-ζ cc-pVDZ basis set as implemented in ORCA 4 [65]. All DFT calculations were performed with Turbomole 7.1 [66–67] and the NCIPLOT code was employed for the visualization non-covalent interactions [48–49].

## Experimental Details

**General remarks:** Catalytic reactions were carried out in Schlenk flasks under nitrogen atmosphere using predried glassware. 1,2-Dichlorethane (DCE) was dried and distilled over CaH_2_ under N_2_. *N*-Cyano-*N*-phenyl-*p*-toluenesulfonamide (**2a**) [68] and Cp*Co(CO)I_2_ [69] were synthesized according to previously described methods. Other chemicals were obtained from commercial sources and were used without further purification.

**Kinetic experiments of the cobalt(III)-catalyzed C–H cyanation:** A suspension of **1** (78 mg, 0.50 mmol), **2** (0.75 mmol), [Cp*Co(CO)I_2_] (6.0 mg, 2.5 mol %), AgSbF_6_ (8.6 mg, 5.0 mol %) and KOAc (2.5 mg, 5.0 mol %) in DCE (2.0 mL) was heated at 120 °C. Aliquots up to ca 15% conversion (25 µL; 10, 20, 30, 40, 50 min) were periodically removed by a syringe and directly analyzed by GC using *n*-dodecane (30 µL) as internal standard.

**2-(Pyridin-2-yl)benzonitrile (3a):** ^1^H NMR (CDCl_3_, 400 MHz) δ 8.73–8.70 (ddd, *J* = 4.7, 1.8, 0.9 Hz, 1H), 7.82–7.71 (m, 4H), 7.64 (dd, *J* = 7.6, 1.4 Hz, 1H), 7.49 (dd, *J* = 7.6, 1.2 Hz, 1H), 7.31 (ddd, *J* = 7.4, 4.7, 1.2 Hz, 1H); ^13^C NMR (CDCl_3_, 125 MHz) δ 155.1 (C_q_), 149.8 (CH), 143.4 (C_q_), 136.7 (CH), 134.0 (CH), 132.7 (CH), 129.9 (CH), 128.6 (CH), 123.2 (CH), 123.1 (CH), 118.6 (C_q_), 111.0 (C_q_); IR (ATR): 3350, 2224, 1560, 1464, 758, 509 cm^−1^. EIMS *m*/*z* (relative intensity): 180 (100) [M^+^], 154 (5), 140 (5), 126 (5), 102 (5), 75 (5); HRMS (EI) *m*/*z*: [M^+^] calcd. for C_12_H_8_N_2_, 180.0687; found, 180.0684. The analytical data are in accordance with those reported in literature [30].

## Supporting Information

File 1 Cartesian coordinates, energies of all calculated structures, and details of computational methods.
