# Supplementary material for: Cobalt-catalyzed C–H cyanations: Insights into the reaction mechanism and the role of London dispersion
Source: Beilstein J Org Chem. 2018 Jun 25;14:1537–45. doi: 10.3762/bjoc.14.130 (PMC6036974; doi:10.3762/bjoc.14.130)
Supplement: File 1 — Cartesian coordinates, energies of all calculated structures, and details of computational methods. [file Beilstein_J_Org_Chem-14-1537-s001.pdf]

Supporting Information

for

Cobalt-catalyzed C–H cyanations: Insights into the reaction mechanism and the role of London dispersion

Eric Detmar[1], Valentin Müller[2], Daniel Zell[2], Lutz Ackermann[2*] and Martin Breugst[1*]

Address: [1]Department für Chemie, Universität zu Köln, Greinstraße 4, 50939 Köln, Germany and [2]Institut für Organische und Biomolekulare Chemie, Georg-August-Universität Göttingen, Tammannstraße 2, 37077 Göttingen, Germany.

Email: Lutz Ackermann* - Lutz.Ackermann@chemie.uni-goettingen.de; Martin Breugst* - mbreugst@uni-koeln.de.

*Corresponding author

Cartesian coordinates, energies of all calculated structures, and details of computational methods

Table of contents

1	Computational details	S2
2	NCIPLOTS	S3
3	DID-PLOTS	S4
4	Optimization with B3LYP-D3BJ/def2-TZVP	S5
5	Optimization with B3LYP/def2-TZVP	S21
6	Optimization with M06-L/def2-TZVPP	S37
7	Optimization with B3LYP-D3BJ/def2-SVP for **1a** (R = H)	S53
8	Optimization with B3LYP-D3BJ/def2-SVP for **1b** (R = Me)	S69
9	Optimization with B3LYP-D3BJ/def2-SVP for **1c** (R = F)	S83
10	Optimization with B3LYP-D3BJ/def2-SVP for **1d** (R = COMe)	S97
11	Optimization with B3LYP-D3BJ/def2-SVP for **1e** (R = CN)	S111
12	References	S125

1 Computational details

For all structures, geometry optimizations were performed with three different functionals using the def2-TZVP (def2-TZVPP for M06-L) basis set[1] and the m4 numerical quadrature grid in the gas phase. The hybrid functional B3LYP[2-3] with and without Grimme's dispersion correction D3 (Becke–Johnson damping)[4-5] as well as Truhlar's dispersion-corrected M06-L[6] functional were employed in this investigation. For the latter, the density fitting RI-J approach was used to accelerate the calculations.[7-8] For the analysis of the substituent effect, the B3LYP functional with Grimme's dispersion correction D3 (Becke–Johnson damping) was employed together with the def2-SVP basis set for all non-metals and the def2-TZVP basis set for Co. Vibrational analysis verified that each structure was a minimum or transition state ($i\omega$ < 30 cm^{-1} were tolerated). Thermal corrections were calculated form unscaled harmonic vibrational frequencies at the same levels of theory (imaginary frequencies were ignored) and refer to a standard state of 298.15 K and 1 mol L^{-1}. Entropic contributions to the reported free energies were obtained from partition functions evaluated with Truhlar's quasi-harmonic approximation.[9] This method uses the same approximations as the usual harmonic oscillator approximation except that all vibrational frequencies lower than 100 cm^{-1} are set equal to 100 cm^{-1}. Energies were subsequently derived from single-point calculations employing the functionals described above, the quadruple-ζ basis set def2-QZVP[1] and the COSMO solvation model[10] for dichloroethane (ε = 10.125). The dispersion interaction densities (DID)[11] were calculated at the SCS-LMP2/def2-TZVPP level of theory using MOLPRO 2015.[12-13] The local energy decomposition analysis[14] was performed employing Neese's domain-based local pair-natural orbital (DLPNO) approach to the CCSD(T) method [DLPNO-CCSD(T)][15-17] with tightPNO settings and the double-ζ cc-pVDZ basis set as implemented in ORCA 4.[18] All DFT calculations were performed with TURBOMOLE 7.1[19][20] and the NCIPLOT code was employed for the visualization non-covalent interactions.[21-22]

2 NCIPLOTS

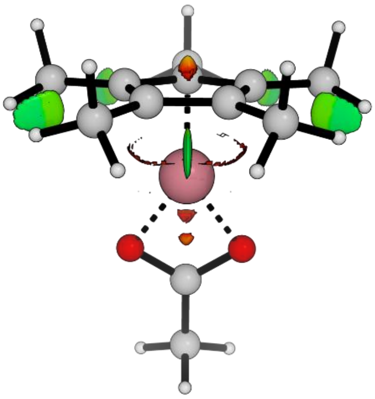

4

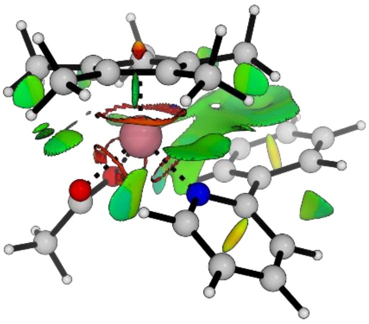

5a

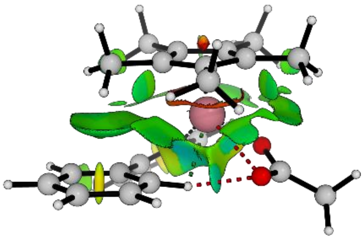

TS1a

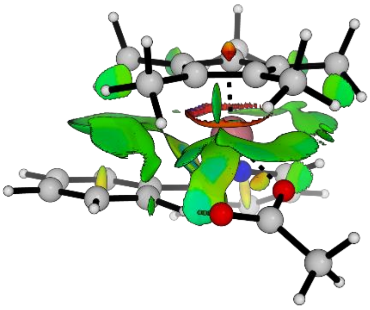

6a

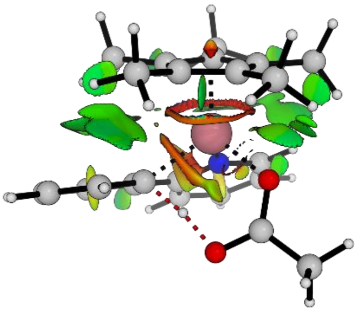

TS2a

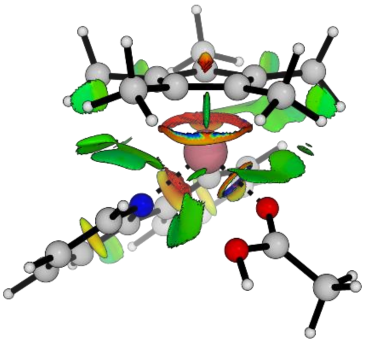

7a

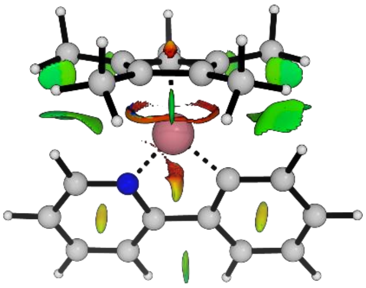

8a

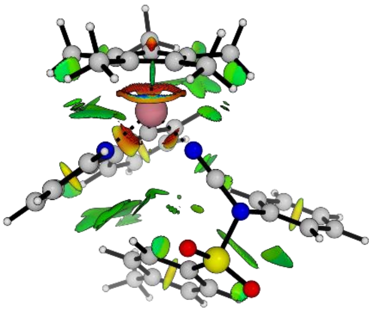

9a

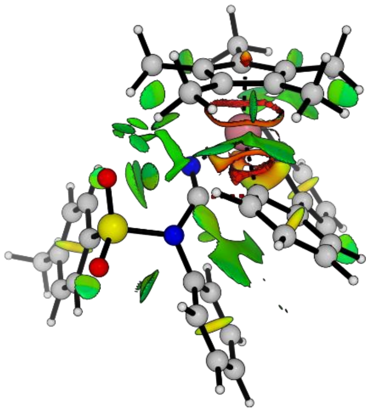

TS3a

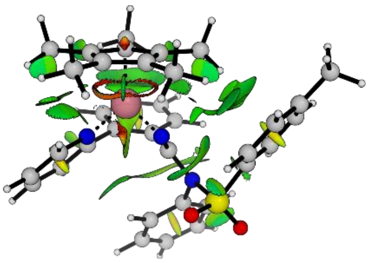

10a

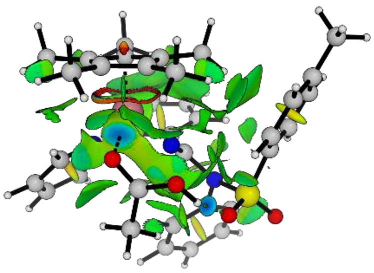

11a

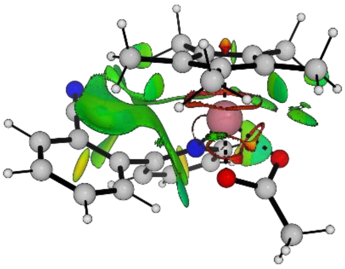

12a

3 DID-PLOTS

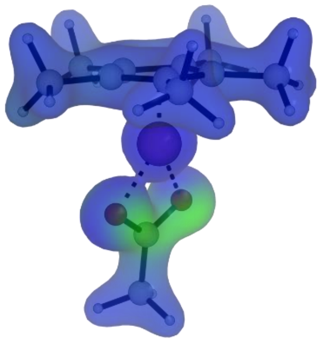

4

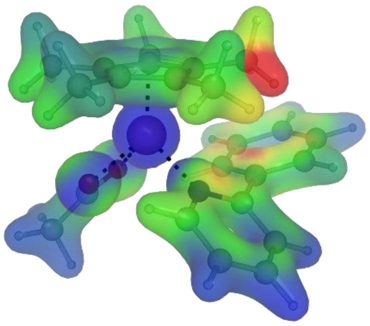

5a

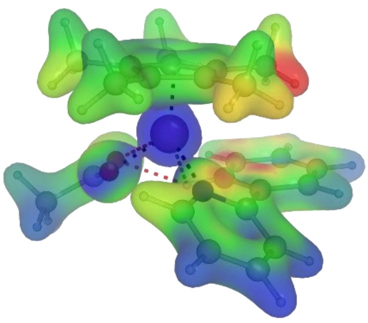

TS1a

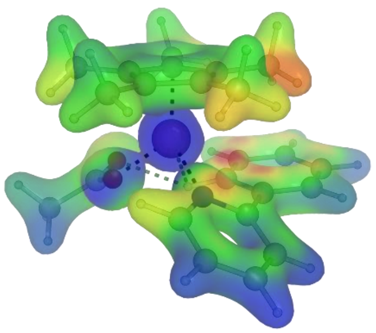

6a

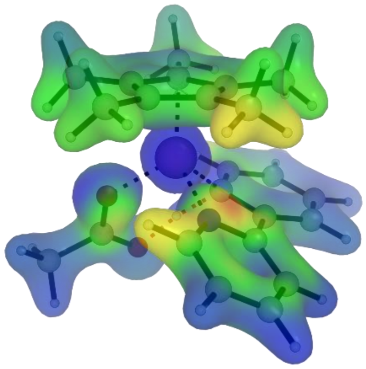

TS2a

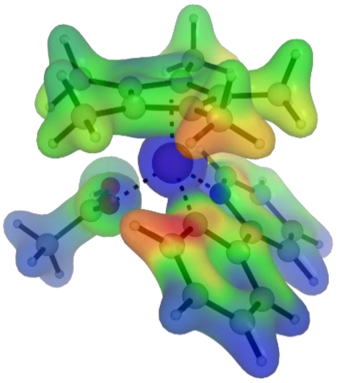

7a

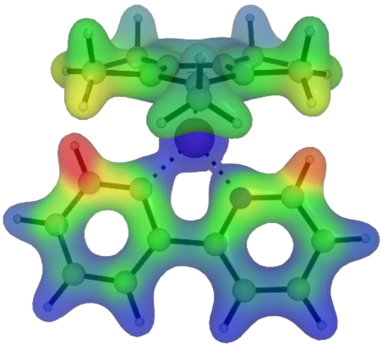

8a

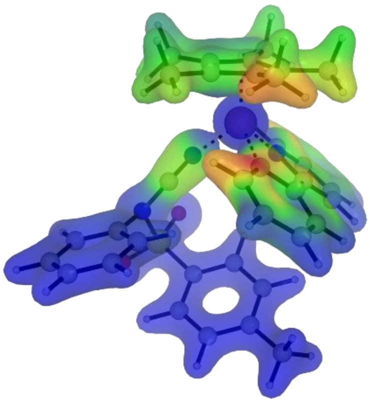

9a

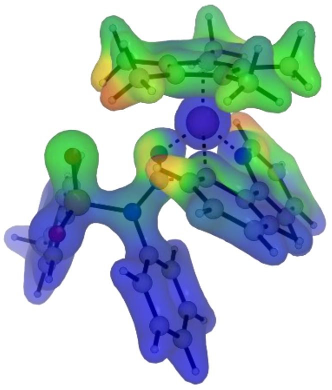

TS3a

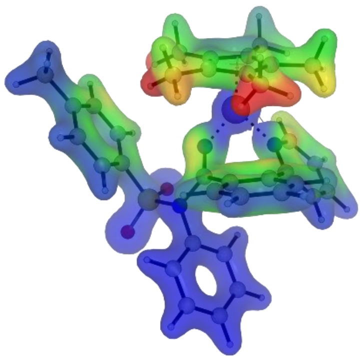

10a

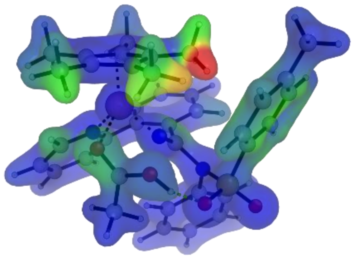

11a

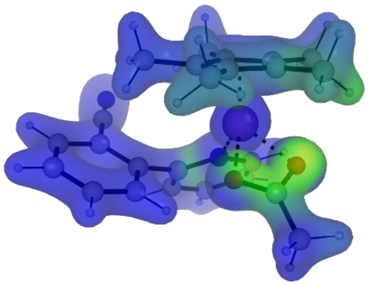

12a

4 Optimization with B3LYP-D3BJ/def2-TZVP

Co-Complex 4

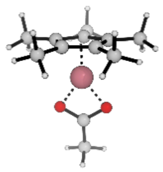

SCF energy:	–2001.183214 hartree
Zero-point correction:	+0.273354 hartree
Enthalpy correction:	+0.291133 hartree
Free energy correction:	+0.221482 hartree
Quasiharmonic free energy correction:	+0.236114 hartree

Cartesian Coordinates

Co	-0.3338849	0.0001070	-0.2138702
O	-1.9163639	-1.0791102	-0.4118014
O	-1.9165847	1.0790829	-0.4106056
C	1.1031015	1.1714013	0.6805372
C	0.7742690	-0.0015049	1.4556237
C	0.2167956	-0.0033694	2.8305587
C	0.8979384	2.5760744	1.1274778
C	1.5577385	0.7298303	-0.5794091
C	1.5582627	-0.7275320	-0.5810909
C	1.9387890	-1.5689934	-1.7464034
C	1.9378725	1.5744536	-1.7425635
C	1.1039342	-1.1723610	0.6778312
C	0.8994958	-2.5781330	1.1217172
C	-2.5943716	-0.0000244	-0.5390256
C	-4.0447257	0.0000572	-0.8380443
H	-4.5128513	0.8982602	-0.4399171
H	-4.5121903	-0.9003429	-0.4441817
H	-4.1702435	0.0027415	-1.9248096
H	0.9202373	-3.2736465	0.2862807
H	-0.0522112	-2.6980105	1.6387484
H	1.6925709	-2.8630294	1.8183322
H	-0.3854158	0.8823067	3.0252487
H	1.0427578	-0.0028470	3.5502993
H	-0.3830914	-0.8909379	3.0237365
H	0.9138177	3.2729014	0.2930128
H	1.6935599	2.8613708	1.8210403
H	-0.0516813	2.6934327	1.6488508
H	1.5771253	1.1539553	-2.6814776
H	3.0286860	1.6275615	-1.8133540
H	1.5588606	2.5895579	-1.6507574
H	1.5741157	-1.1484511	-2.6838130
H	1.5638363	-2.5856424	-1.6552602
H	3.0296109	-1.6177286	-1.8200208

Co-Complex 5a

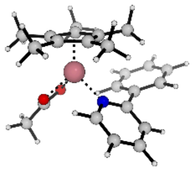

SCF energy:	–2480.538889 hartree
Zero-point correction:	+0.447793 hartree
Enthalpy correction:	+0.473175 hartree
Free energy correction:	+0.385805 hartree
Quasiharmonic free energy correction:	+0.400286 hartree

Cartesian Coordinates

Co	-0.9207628	-0.0130205	0.0338078
O	-0.8499343	-0.5474251	1.9649979
O	-2.1186232	1.0268694	1.2344035
N	0.4772438	1.4386639	0.1667530
C	1.8239737	1.3478216	0.2802207
C	-1.0752689	-0.0054769	-2.0419730
C	-0.2419858	-1.1129854	-1.6318185
C	1.1199788	-1.3988387	-2.1564835
C	-0.6537936	1.0840706	-2.9666977
C	-2.3574812	-0.1850651	-1.4649840

C	-2.3111824	-1.3561532	-0.6350529
C	-3.4370615	-1.9008180	0.1748679
C	-3.5319235	0.7186641	-1.5932535
C	-1.0123108	-1.9442482	-0.7816959
C	-0.5508203	-3.1756420	-0.0872617
C	-1.7017134	0.3547329	2.2305376
C	-2.1609689	0.6307006	3.6199113
H	-3.1343096	1.1164665	3.6102668
H	-1.4426950	1.3023719	4.0968118
H	-2.1932054	-0.2916810	4.1973201
H	-0.8393709	-3.1620833	0.9640635
H	0.5283531	-3.2899492	-0.1454132
H	-1.0145153	-4.0529223	-0.5466113
H	1.6702825	-0.4870506	-2.3743637
H	1.0281767	-1.9578649	-3.0929949
H	1.7113525	-1.9948356	-1.4670718
H	-1.2571392	1.9808183	-2.8393613
H	-0.7614740	0.7563177	-4.0042339
H	0.3904517	1.3533067	-2.8147557
H	-3.9614106	0.9448853	-0.6173004
H	-4.3055910	0.2302700	-2.1916472
H	-3.2739573	1.6551045	-2.0831084
H	-4.0951629	-1.1066938	0.5246678
H	-3.0723955	-2.4521355	1.0404121
H	-4.0384683	-2.5886231	-0.4259037
C	-0.0814708	2.6600045	0.0425562
C	0.6444959	3.8315045	0.0166731
C	2.0249474	3.7548710	0.1411925
C	2.6051028	2.5090684	0.2764950
C	2.5159503	0.0458119	0.3945634
H	2.6351036	4.6483723	0.1438608
H	3.6726332	2.4111289	0.4056077
H	-1.1577057	2.6814342	-0.0272326
H	0.1312086	4.7765919	-0.0881452
C	3.6724573	-0.1649651	-0.3665221
C	2.0959988	-0.9529558	1.2732544
C	2.8068495	-2.1428755	1.3702925
C	4.3735793	-1.3571817	-0.2750372
C	3.9400394	-2.3535322	0.5937952
H	1.2319502	-0.7954307	1.8978781
H	4.0078718	0.5979393	-1.0572825
H	5.2571481	-1.5092020	-0.8809098
H	4.4899288	-3.2821667	0.6731097
H	2.4794145	-2.9022894	2.0686755

Transition State TS1a

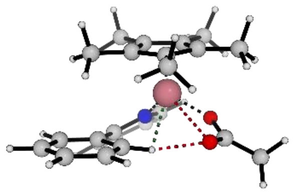

SCF energy:	–2480.526373 hartree
Zero-point correction:	+0.446721 hartree
Enthalpy correction:	+0.471636 hartree
Free energy correction:	+0.385789 hartree
Quasiharmonic free energy correction:	+0.397876 hartree
Imaginary Frequency	64.0 *i*cm^{-1}

Cartesian Coordinates

Co	-0.6547010	0.0387060	-0.0118089
O	-1.3165642	-1.3360092	2.2848486
N	0.6235632	1.5007648	0.2661507
C	1.9510926	1.2588141	0.2981552
C	-0.6899241	0.2602679	-2.0908243
C	-0.2769628	-1.0531846	-1.7943533
C	0.9551533	-1.7232326	-2.2919457
C	0.0733332	1.2631961	-2.8825611
C	-1.9720221	0.4754587	-1.4638564
C	-2.3813520	-0.7698544	-0.8596808
C	-3.6718122	-1.0076121	-0.1601235
C	-2.7989532	1.7108725	-1.5103029

C	-1.3302545	-1.6899357	-1.0233532
C	-1.2981349	-3.0799693	-0.4938692
C	-1.7951973	-0.2068772	2.3972734
C	-2.6287711	0.2000668	3.5840819
H	-2.6328754	1.2792863	3.7211359
H	-2.2630248	-0.3004490	4.4784467
H	-3.6567151	-0.1299553	3.4133777
H	-1.6636272	-3.1121615	0.5313496
H	-0.2922257	-3.4920078	-0.5145737
H	-1.9370106	-3.7207419	-1.1083179
H	1.7324511	-1.0061036	-2.5437396
H	0.7177511	-2.2875721	-3.1985509
H	1.3614652	-2.4220583	-1.5653056
H	-0.1754305	2.2827879	-2.5950932
H	-0.1684482	1.1513445	-3.9431948
H	1.1481913	1.1285353	-2.7737840
H	-3.2143113	1.9375982	-0.5279299
H	-3.6385742	1.5699230	-2.1966988
H	-2.2302455	2.5708630	-1.8583470
H	-4.0108435	-0.1132620	0.3599182
H	-3.5895749	-1.8191175	0.5596813
H	-4.4389823	-1.2793075	-0.8906778
C	0.1670835	2.7587665	0.3298689
C	1.0160394	3.8475116	0.3633675
C	2.3878522	3.6213305	0.3461631
C	2.8556591	2.3188515	0.3269326
C	2.3703662	-0.1480757	0.3723763
H	3.0828702	4.4500516	0.3750741
H	3.9145778	2.1103458	0.3724622
H	-0.9062853	2.8709120	0.3630735
H	0.6076787	4.8468001	0.4103083
C	3.5617467	-0.5968102	-0.2016106
C	1.5611908	-1.0600845	1.0594101
C	1.9266867	-2.3960911	1.1611205
C	3.9278009	-1.9311534	-0.0946480
C	3.1139841	-2.8333279	0.5871715
H	0.6866935	-0.7359984	1.6196580
H	4.1927251	0.0896415	-0.7510810
H	4.8500965	-2.2709116	-0.5469528
H	3.4128271	-3.8694737	0.6768906
H	1.2924442	-3.0757027	1.7142766
O	-1.6359123	0.7063767	1.4825947

Co-Complex 6a

SCF energy:	–2480.527492 hartree
Zero-point correction:	+0.446872 hartree
Enthalpy correction:	+0.472504 hartree
Free energy correction:	+0.385028 hartree
Quasiharmonic free energy correction:	+0.397824 hartree

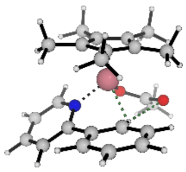

Cartesian Coordinates

Co	-0.5965599	0.0850292	-0.0510027
O	-1.5304679	-1.4323768	2.2364334
N	0.7295470	1.4877437	0.2773280
C	2.0297315	1.1378328	0.3632131
C	-0.6493305	0.4074960	-2.1195778
C	-0.3113863	-0.9392290	-1.8893478
C	0.8806435	-1.6553679	-2.4196939
C	0.1572392	1.4023949	-2.8783688
C	-1.9067220	0.6681896	-1.4595157
C	-2.3829773	-0.5793425	-0.9124715
C	-3.6816303	-0.7800779	-0.2172492
C	-2.6619003	1.9496579	-1.4495121
C	-1.3916601	-1.5511505	-1.1335691
C	-1.4516709	-2.9693808	-0.6859980
C	-1.8276153	-0.2496381	2.3762116

C	-2.6313542	0.2511933	3.5504335
H	-2.2762290	1.2260658	3.8812171
H	-2.5888809	-0.4695321	4.3632478
H	-3.6727872	0.3658174	3.2394457
H	-1.7522535	-3.0290133	0.3594957
H	-0.4920127	-3.4668173	-0.8019246
H	-2.1871851	-3.5127339	-1.2855401
H	1.6858828	-0.9697960	-2.6728323
H	0.6038659	-2.1931611	-3.3311713
H	1.2649293	-2.3850314	-1.7113478
H	0.0014399	2.4157174	-2.5130137
H	-0.1370103	1.3846245	-3.9314589
H	1.2219533	1.1808279	-2.8292877
H	-3.1130009	2.1301857	-0.4738787
H	-3.4709422	1.9101039	-2.1845276
H	-2.0300345	2.7978667	-1.7059794
H	-3.9816887	0.1119938	0.3297073
H	-3.6346542	-1.6156166	0.4772050
H	-4.4590451	-0.9933075	-0.9566615
C	0.3686792	2.7721150	0.3756326
C	1.2994092	3.7865517	0.4956246
C	2.6482462	3.4510519	0.5297128
C	3.0142222	2.1164405	0.4759210
C	2.3125262	-0.3044501	0.4007307
H	3.4036117	4.2197594	0.6249453
H	4.0502861	1.8218790	0.5585274
H	-0.6930088	2.9690080	0.3657282
H	0.9704294	4.8132159	0.5686226
C	3.4820024	-0.8513200	-0.1288434
C	1.3750545	-1.1562133	1.0008327
C	1.5967193	-2.5262991	1.0652960
C	3.7042511	-2.2201312	-0.0581850
C	2.7662433	-3.0600122	0.5392012
H	0.5216872	-0.7659632	1.5569844
H	4.2091836	-0.2133078	-0.6137930
H	4.6125894	-2.6358358	-0.4740095
H	2.9553095	-4.1235854	0.6013102
H	0.8649443	-3.1539041	1.5551138
O	-1.4927048	0.6785454	1.5182289

Transition State TS2a

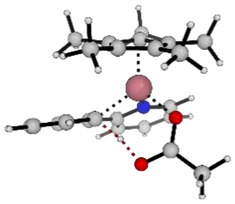

SCF energy:	–2480.510280 hartree
Zero-point correction:	+0.442207 hartree
Enthalpy correction:	+0.467149 hartree
Free energy correction:	+0.380922 hartree
Quasiharmonic free energy correction:	+0.396477 hartree
Imaginary Frequency	1122.70 *i*cm^{-1}

Cartesian Coordinates

Co	-0.6124675	-0.0230263	-0.0513994
O	-0.0740689	-0.4437475	2.8960296
N	0.7562657	1.3364983	0.2409650
C	2.0287792	0.8814089	0.2753129
C	-0.8391249	0.5483688	-2.0782339
C	-0.5047461	-0.8300403	-1.9900318
C	0.6429763	-1.4961708	-2.6684367
C	-0.0485360	1.5800429	-2.8051731
C	-2.0409716	0.7659816	-1.3390341
C	-2.4993044	-0.5155933	-0.8701740
C	-3.7491508	-0.7432895	-0.0965816
C	-2.7749280	2.0501694	-1.1527278
C	-1.5618199	-1.4934988	-1.2619957
C	-1.7102664	-2.9651455	-1.0785043
C	-1.1051987	0.2555451	2.7141146
C	-1.8684829	0.7460738	3.9093848
H	-2.6117635	1.4846545	3.6215434

H	-1.1765554	1.1608264	4.6415000
H	-2.3656802	-0.1059958	4.3773567
H	-2.1041905	-3.2172498	-0.0944261
H	-0.7669723	-3.4865058	-1.2157933
H	-2.4154360	-3.3519962	-1.8197650
H	1.4811800	-0.8146241	-2.8005677
H	0.3392791	-1.8421811	-3.6605530
H	0.9970617	-2.3586491	-2.1081632
H	-0.2518694	2.5845958	-2.4404704
H	-0.3097444	1.5556254	-3.8666055
H	1.0225530	1.3976379	-2.7286092
H	-3.0703639	2.1840812	-0.1113138
H	-3.6867973	2.0585621	-1.7554222
H	-2.1777669	2.9079654	-1.4572514
H	-3.9167689	0.0566274	0.6226205
H	-3.7247573	-1.6897494	0.4403735
H	-4.6044200	-0.7681120	-0.7779976
C	0.4995316	2.6427012	0.3539621
C	1.5110271	3.5787144	0.4601540
C	2.8300146	3.1360255	0.4511316
C	3.0903388	1.7787763	0.3665937
C	2.1345711	-0.5773881	0.2806207
H	3.6455283	3.8426008	0.5300539
H	4.1043825	1.4081772	0.4014689
H	-0.5430272	2.9255124	0.3617950
H	1.2675642	4.6275092	0.5505716
C	3.3036928	-1.2685770	-0.0225870
C	0.9510332	-1.2673314	0.6325386
C	1.0041819	-2.6605837	0.7101951
C	3.3174961	-2.6564987	0.0400906
C	2.1732451	-3.3530213	0.4199117
H	0.3254729	-0.8001152	1.7048450
H	4.1994705	-0.7379539	-0.3185162
H	4.2253325	-3.1956995	-0.1964998
H	2.1994527	-4.4322344	0.4986894
H	0.1328639	-3.2102770	1.0413411
O	-1.5392335	0.5549109	1.5588719

Co-Complex 7a

SCF energy:	–2480.520931 hartree
Zero-point correction:	+0.447403 hartree
Enthalpy correction:	+0.472950 hartree
Free energy correction:	+0.385207 hartree
Quasiharmonic free energy correction:	+0.401528 hartree

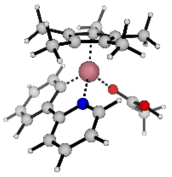

Cartesian Coordinates

Co	-0.4244401	-0.2092268	-0.0460150
C	-2.4532372	-0.8193496	-0.4183748
C	-2.0820500	0.2921080	-1.2441207
C	-2.8587424	1.5589199	-1.3851518
C	-3.5684706	-0.8220208	0.5709016
C	-1.5950071	-1.9067505	-0.6926418
C	-0.6620988	-1.4687318	-1.6936809
C	0.4095416	-2.2935240	-2.3209471
C	-1.7231314	-3.2846753	-0.1297432
C	-0.9843263	-0.1277681	-2.0586955
C	-0.3511188	0.6375054	-3.1699439
H	0.7145939	0.4276571	-3.2458682
H	-0.4669952	1.7105277	-3.0411687
H	-0.8160467	0.3567984	-4.1194836
H	-3.1390153	1.9754832	-0.4191070
H	-3.7792206	1.3709612	-1.9448152
H	-2.2985265	2.3143558	-1.9312374
H	-3.3439430	-1.4518386	1.4315198
H	-4.4763545	-1.2173782	0.1061600
H	-3.7951850	0.1833535	0.9218675

H	-0.8222111	-3.8745722	-0.2890222
H	-2.5432096	-3.8182869	-0.6178617
H	-1.9409608	-3.2727687	0.9395752
H	0.7552088	-3.0857024	-1.6593461
H	1.2697091	-1.6859334	-2.5991468
H	0.0313579	-2.7653212	-3.2321642
C	0.7149389	1.3389496	-0.2791740
C	2.0830524	1.0377462	-0.1491582
C	0.3583415	2.6510021	-0.5640942
C	3.0604105	2.0153779	-0.3581864
C	1.3313298	3.6292734	-0.7622086
H	-0.6824482	2.9326164	-0.6365618
C	2.6831298	3.3102587	-0.6755178
H	4.1118016	1.7748711	-0.2640618
H	1.0318778	4.6447937	-0.9912402
H	3.4350150	4.0700678	-0.8416601
N	1.2592820	-1.0649635	0.5027926
C	2.3709275	-0.3205424	0.2941570
C	1.3606627	-2.2881456	1.0307449
C	3.6310433	-0.8573062	0.5621720
C	2.5794007	-2.8664623	1.3300311
H	0.4341366	-2.8084497	1.2181370
C	3.7369548	-2.1381957	1.0725133
H	4.5151718	-0.2643037	0.3807676
H	2.6158942	-3.8597770	1.7536005
H	4.7100733	-2.5622507	1.2824516
O	-0.7421384	0.1710903	1.8904832
C	-1.2235262	1.1187088	2.4861835
O	-1.8461949	2.0752427	1.8026300
C	-1.1383707	1.2560671	3.9731489
H	-0.6898790	0.3647782	4.4009532
H	-2.1294094	1.4149243	4.4031728
H	-0.5194046	2.1228986	4.2197344
H	-2.1514637	2.7871589	2.3827211

Co-Complex 8a

SCF energy:	–2251.386415 hartree	
Zero-point correction:	+0.383520 hartree	
Enthalpy correction:	+0.403976 hartree	
Free energy correction:	+0.328798 hartree	
Quasiharmonic free energy correction:	+0.339919 hartree	

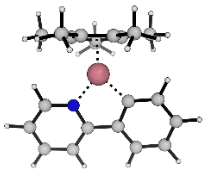

Cartesian Coordinates

Co	0.4372977	0.0931385	-0.2909065
C	2.3934893	-0.2662740	-1.0622914
C	2.2352267	1.0904661	-0.5481953
C	2.4410671	2.3234992	-1.3587638
C	2.7341562	-0.5873702	-2.4763452
C	2.1666637	-1.1641862	-0.0147174
C	1.7715961	-0.3908153	1.1383681
C	1.4131955	-0.9395072	2.4735015
C	2.3274960	-2.6462741	-0.0279117
C	1.9332251	1.0071301	0.8246815
C	1.8677017	2.1094555	1.8238508
H	0.9960437	2.0270223	2.4704860
H	1.8555812	3.0901580	1.3555115
H	2.7581517	2.0605430	2.4568850
H	1.9448094	2.2545139	-2.3276258
H	3.5073968	2.4731158	-1.5513455
H	2.0672850	3.2098050	-0.8512045
H	2.5838175	-1.6406801	-2.7047920
H	3.7836933	-0.3503925	-2.6732487
H	2.1396701	0.0035118	-3.1752454
H	1.5502554	-3.1501750	0.5457212
H	3.2843928	-2.9079972	0.4320327
H	2.3356160	-3.0539034	-1.0376211

H	0.9094425	-1.9017717	2.3916964
H	0.7672682	-0.2574100	3.0237302
H	2.3189436	-1.0922091	3.0694175
C	-0.9968559	1.2741463	0.1782658
C	-2.2433379	0.6185964	0.2668861
C	-0.9690324	2.6499077	0.3801693
C	-3.4025381	1.3155669	0.5988063
C	-2.1316035	3.3544676	0.6916705
H	-0.0449440	3.2024641	0.2902288
C	-3.3427998	2.6864475	0.8196566
H	-4.3554167	0.8075831	0.6716204
H	-2.0881624	4.4263536	0.8376886
H	-4.2412388	3.2320230	1.0756015
N	-0.9960405	-1.2290929	-0.4978341
C	-2.2211587	-0.7950490	-0.1012035
C	-0.8519444	-2.4719312	-0.9736089
C	-3.3111082	-1.6592027	-0.1283795
C	-1.9022194	-3.3675679	-1.0428571
H	0.1349541	-2.7469495	-1.3094986
C	-3.1513091	-2.9539628	-0.5947722
H	-4.2786082	-1.3130825	0.2035656
H	-1.7397636	-4.3603918	-1.4366203
H	-3.9953245	-3.6306087	-0.6205030

Co-Complex 9a

SCF energy:	–3450.216009 hartree
Zero-point correction:	+0.620049 hartree
Enthalpy correction:	+0.656744 hartree
Free energy correction:	+0.538210 hartree
Quasiharmonic free energy correction:	+0.579781 hartree

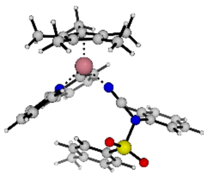

Cartesian Coordinates

Co	1.7215973	0.0093009	-0.9198476
C	2.8470004	1.6959880	-1.4075073
C	3.5818803	0.9119278	-0.4769272
C	4.1442686	1.3590929	0.8272151
C	2.4359702	3.1222170	-1.2520497
C	2.6997513	0.9203331	-2.6238844
C	3.2690673	-0.3407373	-2.4094634
C	3.4298811	-1.4361832	-3.4081755
C	2.0471750	1.4177654	-3.8684863
C	3.7525821	-0.3838659	-1.0456748
C	4.4519045	-1.5341653	-0.4031993
H	4.0860464	-2.4871640	-0.7841181
H	4.3112340	-1.5279290	0.6770339
H	5.5271905	-1.4928876	-0.5979983
H	3.6229902	2.2261526	1.2241695
H	5.1920978	1.6379827	0.6828143
H	4.1080531	0.5718242	1.5776306
H	1.4583284	3.3059258	-1.6977341
H	3.1515553	3.7844825	-1.7470829
H	2.3895086	3.4158289	-0.2047524
H	1.7888986	0.6048162	-4.5447488
H	2.7191668	2.0950025	-4.4027097
H	1.1362662	1.9723095	-3.6434254
H	2.6850945	-1.3837499	-4.2015630
H	3.3751803	-2.4213490	-2.9474266
H	4.4130434	-1.3591772	-3.8812247
C	1.2768946	0.0586565	0.9550253
C	1.0653736	-1.2181402	1.5033851
C	1.1236687	1.1653462	1.7798262
C	0.8018645	-1.3780037	2.8672429
C	0.8350485	1.0085949	3.1338102
H	1.2314215	2.1653070	1.3789087
C	0.6987889	-0.2636829	3.6842976
H	0.6597815	-2.3644665	3.2906324

H	0.7279276	1.8822959	3.7651683
H	0.5015878	-0.3809706	4.7417930
N	1.1254684	-1.8564063	-0.7555280
C	0.9970468	-2.2932788	0.5214730
C	0.8898046	-2.6844805	-1.7764346
C	0.7237873	-3.6376248	0.7725090
C	0.5779808	-4.0185373	-1.5879979
H	0.9490465	-2.2549050	-2.7649427
C	0.5218359	-4.5061222	-0.2863247
H	0.6594491	-3.9907994	1.7914042
H	0.3878166	-4.6535394	-2.4410882
H	0.3054193	-5.5500475	-0.1011105
O	-4.7411753	0.4075829	-1.9394632
H	-5.1131416	0.5203457	0.6516472
H	-4.1818463	3.0618305	-2.1364307
H	-4.9954973	4.9556381	-0.7500178
H	-4.9599012	-0.1513347	3.0266714
C	-4.3745332	-0.1996964	0.9734504
C	-4.2816565	-0.5835554	2.3022633
C	-3.7968506	3.2263652	-1.1405369
C	-4.2485570	4.2796789	-0.3558550
S	-3.4811534	-0.1616002	-1.5911277
C	-3.4954551	-0.7606686	0.0517729
O	-2.7451860	-1.0407287	-2.4465393
N	-2.4001218	1.2459795	-1.4204815
C	-2.8421015	2.3654365	-0.6174674
C	-3.3281713	-1.5133335	2.7202129
H	-4.0591288	-1.5751141	4.7468470
C	-3.7391481	4.4713104	0.9242822
H	-3.0736275	-2.9688939	4.2838829
C	-3.1937526	-1.8908655	4.1660050
H	-4.0920897	5.2981653	1.5264767
C	-1.1215154	0.9339573	-1.3977464
C	-2.5436384	-1.7038980	0.4351391
C	-2.4766212	-2.0758858	1.7644968
C	-2.3302259	2.5365707	0.6643974
N	-0.0063807	0.6447427	-1.3756661
C	-2.7776859	3.6032410	1.4312129
H	-1.8829859	-2.1376114	-0.3015009
H	-2.3078381	-1.4154366	4.5965954
H	-1.7485818	-2.8145295	2.0702784
H	-1.5970092	1.8431971	1.0566464
H	-2.3835976	3.7476433	2.4285219

Transition State TS3a

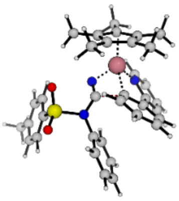

SCF energy:	–3450.180510 hartree
Zero-point correction:	+0.619464 hartree
Enthalpy correction:	+0.654573 hartree
Free energy correction:	+0.540072 hartree
Quasiharmonic free energy correction:	+0.577902 hartree
Imaginary Frequency	413.9 *i*cm^{-1}

Cartesian Coordinates

Co	1.8555440	0.0187405	-0.7271166
C	2.4800149	1.8317415	-1.5501195
C	3.5456886	1.2795708	-0.7517974
C	4.2166250	1.9367079	0.4056297
C	1.8620258	3.1825989	-1.4135665
C	2.1932254	0.9150502	-2.6029910
C	3.0211586	-0.2235896	-2.4364804
C	3.1029360	-1.3820683	-3.3724035
C	1.1508036	1.1077701	-3.6476828
C	3.8676527	0.0130329	-1.2848191
C	4.9175853	-0.9112973	-0.7713806
H	4.6673207	-1.9534192	-0.9595968
H	5.0687323	-0.7879513	0.2993356

H	5.8710615	-0.7089106	-1.2651485
H	3.5783663	2.6831979	0.8698115
H	5.1292826	2.4372913	0.0745203
H	4.4952832	1.2152859	1.1716417
H	0.7764401	3.1406824	-1.4959044
H	2.2234031	3.8318577	-2.2142465
H	2.1232563	3.6541648	-0.4699587
H	0.8228002	0.1569865	-4.0607625
H	1.5495308	1.7124572	-4.4656904
H	0.2802184	1.6214003	-3.2439728
H	2.1152220	-1.7117406	-3.6937037
H	3.6260048	-2.2276582	-2.9311933
H	3.6565931	-1.0986755	-4.2704991
C	1.1684639	0.5784364	1.1530919
C	1.5493369	-0.4449625	2.0433393
C	1.0531183	1.8819978	1.6410994
C	1.8182310	-0.1525685	3.3802019
C	1.3396623	2.1716736	2.9634923
H	0.6928803	2.6647093	0.9902554
C	1.7193883	1.1521985	3.8358461
H	2.1312758	-0.9352959	4.0572962
H	1.2524705	3.1870903	3.3249690
H	1.9350093	1.3779593	4.8707264
N	1.8687196	-1.7756732	0.1262232
C	1.7006503	-1.7763237	1.4662559
C	1.9376163	-2.9372906	-0.5311419
C	1.6382421	-2.9713759	2.1789256
C	1.8965513	-4.1568433	0.1187157
H	2.0187931	-2.8782768	-1.6038970
C	1.7501382	-4.1727988	1.5015538
H	1.4795211	-2.9485315	3.2462500
H	1.9669002	-5.0704362	-0.4524243
H	1.7056571	-5.1098504	2.0386565
O	-3.1506857	2.5842859	0.3965855
H	-4.9673291	0.8038503	0.3771825
H	-1.9951218	2.2870302	2.6741953
H	-2.7389142	1.3986458	4.8668972
H	-6.4512868	-0.9666460	-0.5059088
C	-4.6930723	0.2442703	-0.5045741
C	-5.5196672	-0.7462223	-1.0098299
C	-2.0930237	1.2252272	2.8346481
C	-2.5110099	0.7186463	4.0577430
S	-2.4233494	1.7359457	-0.4969645
C	-3.4993778	0.5237048	-1.1634497
O	-1.5699401	2.2703617	-1.5188901
N	-1.3565568	0.8401209	0.5283770
C	-1.8108196	0.3459162	1.7965703
C	-5.1755562	-1.4569931	-2.1615534
H	-6.7327605	-2.9291390	-1.9524522
C	-2.6300932	-0.6542575	4.2466996
H	-6.7521911	-2.0584602	-3.4804489
C	-6.0972526	-2.4990004	-2.7249812
H	-2.9519658	-1.0416361	5.2035634
C	-0.2753214	0.2330561	-0.0591861
C	-3.1241278	-0.1734694	-2.3086089
C	-3.9677252	-1.1558319	-2.7976679
C	-1.9408936	-1.0286796	1.9727626
N	-0.0127482	-0.3203511	-1.1095014
C	-2.3444928	-1.5278120	3.2030704
H	-2.1881707	0.0528811	-2.7949521
H	-5.5418876	-3.3031907	-3.2063813
H	-3.6871226	-1.6984018	-3.6907494
H	-1.7316244	-1.6942051	1.1462533
H	-2.4489026	-2.5952192	3.3415498

Co-Complex 10a

SCF energy:	–3450.216484 hartree
Zero-point correction:	+0.621363 hartree
Enthalpy correction:	+0.657437 hartree
Free energy correction:	+0.540994 hartree
Quasiharmonic free energy correction:	+0.577472 hartree

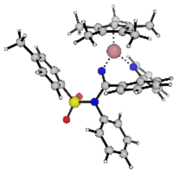

Cartesian Coordinates

Co	1.7507539	-0.8129291	-0.2829225
C	2.6546983	-1.2330470	1.4873574
C	3.2953063	-2.0140276	0.4544032
C	3.3403145	-3.5036995	0.4180722
C	2.0036292	-1.7680127	2.7137985
C	2.8498569	0.1532650	1.1778362
C	3.5405030	0.2172397	-0.0589909
C	3.9144596	1.4536642	-0.7986074
C	2.3564347	1.3056199	1.9839352
C	3.8317315	-1.1287995	-0.5012583
C	4.5926486	-1.4823345	-1.7357933
H	4.3391850	-0.8265143	-2.5695083
H	4.4113687	-2.5114996	-2.0429095
H	5.6678352	-1.3810707	-1.5630541
H	2.4194913	-3.9407587	0.8028998
H	4.1618340	-3.8686001	1.0402001
H	3.4983144	-3.8806742	-0.5907788
H	1.1327851	-1.1792007	2.9978357
H	2.7102807	-1.7403307	3.5488078
H	1.6853763	-2.7999501	2.5861381
H	2.0788445	2.1468365	1.3517349
H	3.1309650	1.6438480	2.6773395
H	1.4823414	1.0276242	2.5710977
H	3.3277000	2.3061873	-0.4680255
H	3.7754062	1.3373943	-1.8739227
H	4.9714459	1.6806901	-0.6294120
C	-1.1703622	-0.8636839	1.1479747
C	-0.9128925	-2.2251440	0.9224257
C	-1.6966941	-0.4500935	2.3679444
C	-1.2076989	-3.1402326	1.9364168
C	-1.9573991	-1.3681034	3.3751588
H	-1.9080011	0.6002133	2.5166620
C	-1.7119219	-2.7186350	3.1576167
H	-1.0240130	-4.1935307	1.7675218
H	-2.3586803	-1.0322834	4.3221268
H	-1.9167703	-3.4429812	3.9345895
N	0.6592284	-2.2807255	-0.9864758
C	-0.4512942	-2.7570312	-0.3815183
C	1.0416690	-2.7936083	-2.1672131
C	-1.1823779	-3.7856444	-0.9797234
C	0.3576564	-3.8097878	-2.8016690
H	1.9272758	-2.3589420	-2.6082453
C	-0.7815831	-4.3183007	-2.1918566
H	-2.0798735	-4.1351960	-0.4910918
H	0.7084886	-4.1815007	-3.7536182
H	-1.3553401	-5.1070244	-2.6599229
O	-0.9808118	1.7541283	-2.5866141
H	-1.7103508	3.4971634	1.1950174
H	-2.6740193	-1.0840190	-1.7474928
H	-4.9316775	-2.1197824	-1.7713134
H	-0.1372375	4.9898908	2.3728877
C	-0.7588907	3.7501919	0.7478570
C	0.1237898	4.5933744	1.3991776
C	-3.4712385	-0.6843765	-1.1342923
C	-4.7365671	-1.2563641	-1.1484594
S	-1.5890033	2.2667278	-1.3922033
C	-0.4174130	3.2419430	-0.5019745
O	-2.8355920	2.9713594	-1.4555526
N	-1.8971571	0.9825361	-0.2916248

C	-3.2264986	0.4254194	-0.3338493
C	1.3380077	4.9627618	0.8090838
H	2.4012078	5.6365694	2.5562330
C	-5.7544677	-0.7059180	-0.3782891
H	3.2443968	5.9439278	1.0310585
C	2.2657853	5.9140664	1.5092417
H	-6.7430182	-1.1457334	-0.3938710
C	-0.8433600	0.1398051	0.0933912
C	0.7834575	3.5802882	-1.1060247
C	1.6485505	4.4463108	-0.4485222
C	-4.2417954	0.9869153	0.4273620
N	0.3333302	0.2288425	-0.3432782
C	-5.5078161	0.4191617	0.4022721
H	1.0231371	3.1746617	-2.0785790
H	1.8577641	6.9279710	1.4951497
H	2.5783084	4.7321153	-0.9251070
H	-4.0383330	1.8661517	1.0224555
H	-6.3041944	0.8559132	0.9903481

Co-Complex 11a

SCF energy:	–3679.334525 hartree	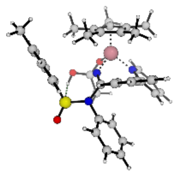
Zero-point correction:	+0.685220 hartree	
Enthalpy correction:	+0.726136 hartree	
Free energy correction:	+0.600350 hartree	
Quasiharmonic free energy correction:	+0.631685 hartree	

Cartesian Coordinates

Co	-1.7455259	-0.8138899	-0.0678357
C	-2.2863996	-1.3220082	1.8337381
C	-3.4463283	-1.5389004	0.9929090
C	-4.7579115	-0.8509901	1.1650823
C	-2.2804332	-0.5175950	3.0853513
C	-1.2504187	-2.2092678	1.3947285
C	-1.7200607	-2.8750329	0.2395193
C	-0.9774086	-3.8684073	-0.5829872
C	0.0882494	-2.3577149	2.0268961
C	-3.1042471	-2.4861312	0.0227466
C	-3.9706974	-3.0212607	-1.0622082
H	-3.4177438	-3.1198232	-1.9937671
H	-4.8361579	-2.3858962	-1.2422972
H	-4.3403387	-4.0119714	-0.7818351
H	-4.6279801	0.1976237	1.4323060
H	-5.3296967	-1.3234135	1.9683356
H	-5.3614826	-0.8980592	0.2604476
H	-1.2813384	-0.1838531	3.3544247
H	-2.6600455	-1.1316845	3.9083678
H	-2.9230668	0.3568014	3.0087914
H	0.8519070	-2.5990206	1.2931215
H	0.0609299	-3.1643103	2.7650868
H	0.3864936	-1.4474446	2.5429599
H	0.0947626	-3.7842197	-0.4271145
H	-1.1763055	-3.7284263	-1.6438157
H	-1.2816413	-4.8832571	-0.3101731
C	0.2377933	1.3604278	1.3980137
C	-0.9901078	2.0299905	1.4786695
C	1.1078265	1.3884078	2.4872644
C	-1.3000606	2.7249987	2.6534920
C	0.7728905	2.0521757	3.6564751
H	2.0639040	0.8913893	2.3985639
C	-0.4379398	2.7301455	3.7376196
H	-2.2434461	3.2506305	2.7216568
H	1.4577966	2.0537142	4.4940185
H	-0.7084131	3.2633888	4.6392027
N	-2.4193367	1.0340867	-0.2820488
C	-1.9618031	2.1281757	0.3657824
C	-3.3866602	1.1751448	-1.2054341

C	-2.4539659	3.3936817	0.0342266
C	-3.9300240	2.3967112	-1.5472443
H	-3.7009447	0.2670052	-1.6949275
C	-3.4408481	3.5360879	-0.9233689
H	-2.0371544	4.2564329	0.5320293
H	-4.7076667	2.4454782	-2.2959923
H	-3.8197003	4.5167372	-1.1791401
O	2.2540427	-0.2413910	-2.5336592
H	4.0178162	-0.4062452	1.2964475
H	0.3919435	2.7364635	-1.5615781
H	0.6419383	5.2002319	-1.6787155
H	4.3954513	-2.4744942	2.5860103
C	3.6815284	-1.3589226	0.9110576
C	3.9003814	-2.5234116	1.6238177
C	1.2285424	3.2265041	-1.0814966
C	1.3708918	4.6065087	-1.1422133
S	2.9058906	0.0565003	-1.2729354
C	3.0485018	-1.4254242	-0.3284789
O	4.1800314	0.7095260	-1.3312510
N	1.9566753	1.0390176	-0.2819149
C	2.1639846	2.4630801	-0.3917253
C	3.5155908	-3.7674431	1.1099447
H	4.7894608	-5.0670315	2.2552842
C	2.4566528	5.2185175	-0.5273294
H	3.1108489	-5.0619758	2.7751997
C	3.7598279	-5.0232205	1.8961509
H	2.5746285	6.2929457	-0.5794417
C	0.6498473	0.6296830	0.1651988
C	2.6589759	-2.6442423	-0.8622440
C	2.9011242	-3.8066786	-0.1404800
C	3.2501894	3.0699660	0.2252818
N	-0.0141308	-0.2607732	-0.4096954
C	3.3966175	4.4478557	0.1501248
H	2.1776654	-2.6884757	-1.8267022
H	3.5684251	-5.9131797	1.2979236
H	2.6110763	-4.7611597	-0.5615612
H	3.9765211	2.4623873	0.7449641
H	4.2452591	4.9224638	0.6247389
O	-1.9596203	-1.1285426	-2.3661957
C	-0.9218593	-0.8088981	-2.9339541
O	0.0543377	-1.6886656	-3.0463644
C	-0.7043498	0.5338750	-3.5644825
H	0.2655805	0.9323138	-3.2694066
H	-1.5010054	1.2176965	-3.2882821
H	-0.6931310	0.4155559	-4.6506771
H	0.9162259	-1.2228692	-3.1380881

Co-Complex 12a

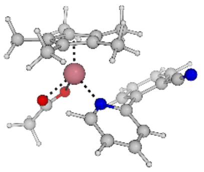

SCF energy:	–2572.782113 hartree
Zero-point correction:	+0.446097 hartree
Enthalpy correction:	+0.473373 hartree
Free energy correction:	+0.381413 hartree
Quasiharmonic free energy correction:	+0.399643 hartree

Cartesian Coordinates

Co	-1.3949204	-0.7729702	0.1851247
O	-1.0776097	-1.4476775	2.0488139
O	-2.5723590	0.0303066	1.5801101
N	-0.1659130	0.8012312	0.3362984
C	1.1843046	0.8325700	0.3527185
C	-1.3190505	-0.7882584	-1.9158340
C	-0.7704226	-2.0021549	-1.3872038
C	0.5487462	-2.6082932	-1.7203622
C	-0.6096236	0.1775372	-2.7983383
C	-2.6521426	-0.6639558	-1.4430737
C	-2.9569246	-1.8368062	-0.6532736

C	-4.2624352	-2.1084284	0.0081122
C	-3.6017676	0.4520205	-1.7088759
C	-1.8008366	-2.6539406	-0.6321480
C	-1.6252867	-3.9395319	0.0947069
C	-1.9946948	-0.6752288	2.4660540
C	-2.3534403	-0.5760422	3.9090792
H	-3.4032846	-0.3117783	4.0201762
H	-1.7541080	0.2164493	4.3646649
H	-2.1322132	-1.5101085	4.4215893
H	-2.3636172	-4.0660002	0.8830028
H	-0.6338200	-4.0039438	0.5412461
H	-1.7310265	-4.7739035	-0.6046524
H	1.1983687	-1.9034155	-2.2302961
H	0.3975743	-3.4593196	-2.3904504
H	1.0646753	-2.9771300	-0.8351059
H	-0.9761274	1.1937779	-2.6651221
H	-0.7798495	-0.0972647	-3.8431872
H	0.4652788	0.1794246	-2.6350603
H	-4.1558731	0.7228117	-0.8095496
H	-4.3332259	0.1484494	-2.4627669
H	-3.0927962	1.3375035	-2.0844578
H	-4.6928312	-1.1987077	0.4235118
H	-4.1640050	-2.8376220	0.8100197
H	-4.9702541	-2.5114059	-0.7214215
C	-0.8391739	1.9677853	0.3019681
C	-0.2177472	3.1989948	0.2616725
C	1.1687747	3.2431740	0.2717674
C	1.8649503	2.0501327	0.3251029
C	1.9879110	-0.4129947	0.4224505
H	1.6979626	4.1862610	0.2462196
H	2.9444126	2.0434892	0.3532677
H	-1.9149789	1.8922499	0.3179695
H	-0.8162099	4.0982717	0.2293910
C	2.9841405	-0.6633103	-0.5428023
C	1.8652697	-1.2937859	1.4919004
C	2.7064623	-2.3943568	1.6053800
C	3.8287885	-1.7688784	-0.4231805
C	3.6907682	-2.6310602	0.6532969
H	1.1116455	-1.1154681	2.2421310
H	4.5797235	-1.9445091	-1.1811557
H	4.3506161	-3.4828658	0.7484965
H	2.5992603	-3.0603127	2.4514958
C	3.0903572	0.1677198	-1.6998499
N	3.1327685	0.8191094	-2.6498805

2-Phenylpyridine (1a)

SCF energy:	–479.300131 hartree
Zero-point correction:	+0.169338 hartree
Enthalpy correction:	+0.177677 hartree
Free energy correction:	+0.133022 hartree
Quasiharmonic free energy correction:	+0.135641 hartree

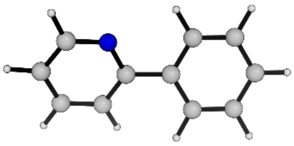

Cartesian Coordinates

C	2.8467963	1.1671023	-0.2177377
C	1.4587239	1.1905461	-0.2245998
C	3.5243589	-0.0250252	0.0092096
C	2.8019101	-1.1947946	0.2205986
C	1.4152651	-1.1728124	0.2087030
C	0.7223080	0.0223469	-0.0068442
C	-0.7594802	0.0243571	-0.0043901
C	-1.4971113	1.1882017	0.2421389
N	-1.3672731	-1.1485217	-0.2337846
C	-2.6954887	-1.1921246	-0.2429002
C	-3.5034535	-0.0826280	-0.0267097
C	-2.8810462	1.1322857	0.2259839
H	0.9495962	2.1233887	-0.4271137

H	3.3996930	2.0805502	-0.3972660
H	4.6068111	-0.0433334	0.0159218
H	3.3219913	-2.1283010	0.3956693
H	0.8459035	-2.0780935	0.3638609
H	-3.4652010	2.0238362	0.4171219
H	-0.9943628	2.1185918	0.4638181
H	-3.1386852	-2.1643095	-0.4346894
H	-4.5811055	-0.1735528	-0.0491907

N-Cyano-4-methyl-*N*-phenylbenzenesulfonamide (2a)

SCF energy:	–1198.775829 hartree
Zero-point correction:	+0.233956 hartree
Enthalpy correction:	+0.250021 hartree
Free energy correction:	+0.184083 hartree
Quasiharmonic free energy correction:	+0.201437 hartree

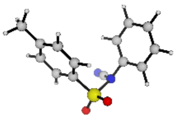

Cartesian Coordinates

N	1.3602715	-1.0159033	-0.4773079
C	1.8575703	0.3334851	-0.3515422
C	1.3355646	1.3441473	-1.1510957
C	1.8124979	2.6399600	-1.0078415
C	2.7910899	2.9223439	-0.0614396
C	3.3017685	1.9036428	0.7360217
C	2.8427289	0.6013049	0.5901647
H	0.5705222	1.1124865	-1.8805240
H	1.4137559	3.4285260	-1.6325504
H	3.1597029	3.9337772	0.0508362
H	4.0681008	2.1195115	1.4689524
H	3.2288894	-0.2014119	1.2004799
C	-1.1379257	-0.6463367	0.5403702
C	-1.2642670	0.5660334	1.2104241
C	-2.0924399	-1.0650154	-0.3831062
C	-3.1885999	-0.2535921	-0.6253145
C	-3.3454699	0.9674270	0.0359510
C	-2.3696710	1.3619953	0.9528489
H	-2.4752441	2.3068994	1.4713169
H	-0.5089235	0.8710845	1.9203223
H	-1.9718561	-2.0088352	-0.8955882
H	-3.9359068	-0.5725053	-1.3414034
C	-4.5548804	1.8223904	-0.2166118
H	-5.3937588	1.4916153	0.4024321
H	-4.8740493	1.7597533	-1.2576543
H	-4.3609912	2.8679440	0.0235411
S	0.3076326	-1.6176027	0.7771363
O	0.0552150	-2.9782390	0.4206791
O	0.9429016	-1.2428891	2.0030405
C	1.0884735	-1.4698459	-1.7086678
N	0.8557779	-1.8522413	-2.7733001

Acetic Acid

SCF energy:	–229.104401 hartree
Zero-point correction:	+0.061502 hartree
Enthalpy correction:	+0.066498 hartree
Free energy correction:	+0.033750 hartree
Quasiharmonic free energy correction:	+0.034724 hartree

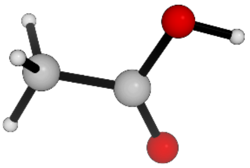

Cartesian Coordinates

C	-1.3930025	-0.1076533	0.0000039
C	0.0903931	0.1246858	0.0000651
H	-1.6760606	-0.6890686	-0.8788890
H	-1.9111177	0.8469029	-0.0004550
H	-1.6761644	-0.6881938	0.8794517
O	0.6399902	1.1944858	0.0005002
O	0.7771299	-1.0447711	-0.0003740

H	1.7203318	-0.8169479	-0.0000830

2-(Pyridin-2-yl)benzonitrile (3a)

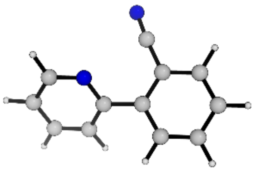

SCF energy:	–571.544021 hartree
Zero-point correction:	+0.167853 hartree
Enthalpy correction:	+0.178016 hartree
Free energy correction:	+0.129099 hartree
Quasiharmonic free energy correction:	+0.132128 hartree

Cartesian Coordinates

C	-2.6326341	-1.7641711	-0.2541769
C	-1.2490443	-1.6621664	-0.2221341
C	-3.4179512	-0.6263783	-0.1131884
C	-2.8122348	0.6069485	0.0644319
C	-1.4180449	0.7153833	0.0941704
C	-0.6155254	-0.4310573	-0.0556742
C	0.8626056	-0.3485807	-0.0514945
C	1.6369012	-1.3002625	0.6168539
N	1.4085255	0.6734877	-0.7169767
C	2.7330831	0.7810000	-0.7394048
C	3.5834858	-0.1204954	-0.1096028
C	3.0177941	-1.1817375	0.5834469
H	-0.6430339	-2.5482806	-0.3578421
H	-3.0968244	-2.7311873	-0.3985311
H	-4.4971523	-0.6981604	-0.1395080
H	-3.4082501	1.5004306	0.1907130
C	-0.8646986	2.0116537	0.3434673
H	3.6412977	-1.9009011	1.0993699
H	1.1625365	-2.0996276	1.1694543
H	3.1329653	1.6277060	-1.2870432
H	4.6558166	0.0127422	-0.1584053
N	-0.5127276	3.0796542	0.5947145

4-Methyl-*N*-phenylbenzenesulfonamide

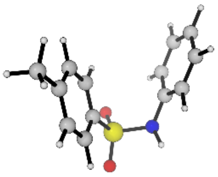

SCF energy:	–1106.557745 hartree
Zero-point correction:	+0.235977 hartree
Enthalpy correction:	+0.250200 hartree
Free energy correction:	+0.188136 hartree
Quasiharmonic free energy correction:	+0.205376 hartree

Cartesian Coordinates

N	1.3504375	1.2413799	-0.8281482
C	1.9120473	-0.0217560	-0.5017076
C	2.5945488	-0.2006591	0.7002219
C	3.1321622	-1.4438861	1.0040456
C	3.0182641	-2.5042427	0.1110006
C	2.3513976	-2.3170531	-1.0936925
C	1.7882653	-1.0842359	-1.3948886
H	2.6913243	0.6311087	1.3822177
H	3.6584018	-1.5790611	1.9404720
H	3.4503681	-3.4671921	0.3502346
H	2.2560221	-3.1346881	-1.7967424
H	1.2437116	-0.9442426	-2.3212894
C	-1.1314002	0.6806559	0.1512993
C	-1.0835462	-0.3262033	1.1099055
C	-2.1307639	0.6945288	-0.8153084
C	-3.0849184	-0.3119330	-0.8189241
C	-3.0521063	-1.3406912	0.1243565
C	-2.0419997	-1.3279384	1.0875860
H	-2.0083103	-2.1108824	1.8354846
H	-0.3118112	-0.3126413	1.8656575
H	-2.1656580	1.4980898	-1.5381088
H	-3.8733293	-0.2947206	-1.5617374
C	-4.0683561	-2.4478454	0.0919281

H	-5.0022495	-2.1175890	-0.3637666
H	-4.2862542	-2.8166906	1.0948996
H	-3.6972746	-3.2937911	-0.4940599
S	0.1384263	1.9138096	0.1114517
O	0.6575498	2.0592301	1.4407509
O	-0.3249005	3.0377950	-0.6535808
H	1.1131514	1.3506657	-1.8061730

5 Optimization with B3LYP/def2-TZVP

Co-Complex 4

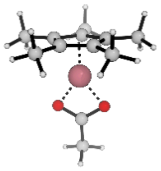

SCF energy:	–2001.119872 hartree
Zero-point correction:	+0.273354 hartree
Enthalpy correction:	+0.291133 hartree
Free energy correction:	+0.221482 hartree
Quasiharmonic free energy correction:	+0.236111 hartree

Cartesian Coordinates

Co	-0.3338847	0.0001070	-0.2138701
O	-1.9163637	-1.0791099	-0.4118014
O	-1.9165845	1.0790830	-0.4106055
C	1.1031016	1.1714012	0.6805374
C	0.7742690	-0.0015050	1.4556238
C	0.2167956	-0.0033696	2.8305588
C	0.8979386	2.5760742	1.1274781
C	1.5577386	0.7298303	-0.5794089
C	1.5582628	-0.7275320	-0.5810907
C	1.9387889	-1.5689932	-1.7464034
C	1.9378719	1.5744536	-1.7425636
C	1.1039342	-1.1723610	0.6778313
C	0.8994958	-2.5781331	1.1217172
C	-2.5943714	-0.0000241	-0.5390256
C	-4.0447254	0.0000574	-0.8380446
H	-4.5128508	0.8982618	-0.4399202
H	-4.5121906	-0.9003413	-0.4441795
H	-4.1702428	0.0027383	-1.9248100
H	0.9202373	-3.2736465	0.2862805
H	-0.0522112	-2.6980107	1.6387483
H	1.6925709	-2.8630296	1.8183321
H	-0.3854159	0.8823064	3.0252488
H	1.0427579	-0.0028469	3.5502994
H	-0.3830911	-0.8909382	3.0237367
H	0.9138182	3.2729014	0.2930132
H	1.6935600	2.8613705	1.8210408
H	-0.0516812	2.6934327	1.6488510
H	1.5771245	1.1539551	-2.6814774
H	3.0286854	1.6275619	-1.8133545
H	1.5588597	2.5895578	-1.6507576
H	1.5741153	-1.1484509	-2.6838129
H	1.5638361	-2.5856422	-1.6552601
H	3.0296107	-1.6177286	-1.8200212

Co-Complex 5a

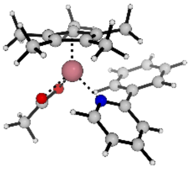

SCF energy:	–2480.401987 hartree
Zero-point correction:	+0.447794 hartree
Enthalpy correction:	+0.473175 hartree
Free energy correction:	+0.385808 hartree
Quasiharmonic free energy correction:	+0.400275 hartree

Cartesian Coordinates

Co	-0.9207622	-0.0130385	0.0337592
O	-0.8499233	-0.5475690	1.9648005
O	-2.1186294	1.0268761	1.2345390
N	0.4772368	1.4386753	0.1666429
C	1.8239589	1.3478776	0.2802088
C	-1.0752400	-0.0055620	-2.0420413
C	-0.2420275	-1.1131112	-1.6317965
C	1.1199575	-1.3989926	-2.1563870
C	-0.6536473	1.0839248	-2.9667847
C	-2.3574627	-0.1850185	-1.4650570

C	-2.3112704	-1.3560772	-0.6350703
C	-3.4371925	-1.9006233	0.1748719
C	-3.5318333	0.7188060	-1.5933689
C	-1.0124427	-1.9442766	-0.7816576
C	-0.5510960	-3.1756770	-0.0871368
C	-1.7016639	0.3545904	2.2305294
C	-2.1607327	0.6304107	3.6199922
H	-3.1347258	1.1148808	3.6105646
H	-1.4432035	1.3032970	4.0963076
H	-2.1914870	-0.2917860	4.1977655
H	-0.8395184	-3.1619720	0.9642212
H	0.5280480	-3.2902010	-0.1454069
H	-1.0150119	-4.0529088	-0.5463596
H	1.6703705	-0.4872030	-2.3739869
H	1.0282078	-1.9577724	-3.0930494
H	1.7112106	-1.9951980	-1.4670491
H	-1.2569745	1.9806963	-2.8395326
H	-0.7612271	0.7561572	-4.0043252
H	0.3905952	1.3531174	-2.8147393
H	-3.9614488	0.9449080	-0.6174457
H	-4.3054464	0.2305759	-2.1919646
H	-3.2737223	1.6553080	-2.0830342
H	-4.0952628	-1.1064227	0.5245561
H	-3.0725517	-2.4518418	1.0404909
H	-4.0386144	-2.5884761	-0.4258265
C	-0.0815237	2.6599947	0.0424517
C	0.6444076	3.8315216	0.0166603
C	2.0248466	3.7549320	0.1412822
C	2.6050463	2.5091484	0.2765890
C	2.5159748	0.0458755	0.3945457
H	2.6349518	4.6484675	0.1440310
H	3.6725667	2.4112106	0.4057847
H	-1.1577547	2.6813613	-0.0273954
H	0.1311080	4.7766025	-0.0881555
C	3.6724987	-0.1648546	-0.3665296
C	2.0960276	-0.9529089	1.2732074
C	2.8068987	-2.1428268	1.3702204
C	4.3736463	-1.3570539	-0.2750526
C	3.9401071	-2.3534392	0.5937461
H	1.2319698	-0.7954456	1.8978322
H	4.0078757	0.5980948	-1.0572565
H	5.2572234	-1.5090548	-0.8809186
H	4.4900150	-3.2820665	0.6730282
H	2.4794377	-2.9022523	2.0685794

Transition State TS1a

SCF energy:	–2480.385691 hartree
Zero-point correction:	+0.446624 hartree
Enthalpy correction:	+0.471625 hartree
Free energy correction:	+0.385169 hartree
Quasiharmonic free energy correction:	+0.398912 hartree
Imaginary Frequency	73.0 *i*cm^{-1}

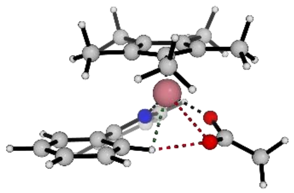

Cartesian Coordinates

Co	-0.6605453	0.0425209	-0.0096527
O	-1.3189155	-1.3367883	2.2572650
N	0.6245071	1.5006373	0.2597780
C	1.9519038	1.2597258	0.3009209
C	-0.6948075	0.2556863	-2.0904180
C	-0.2838638	-1.0567857	-1.7885041
C	0.9468569	-1.7352245	-2.2782421
C	0.0656612	1.2571042	-2.8865205
C	-1.9768574	0.4751202	-1.4643019
C	-2.3890688	-0.7671017	-0.8561388
C	-3.6810231	-0.9992826	-0.1573360
C	-2.7996304	1.7124079	-1.5231045

C	-1.3394243	-1.6892754	-1.0171351
C	-1.3060885	-3.0790756	-0.4883087
C	-1.7958963	-0.2084491	2.3888840
C	-2.6285030	0.1849470	3.5802353
H	-2.5175984	1.2434343	3.8084598
H	-2.3577418	-0.4251795	4.4389008
H	-3.6799920	-0.0012381	3.3466977
H	-1.7151914	-3.1223100	0.5193193
H	-0.2925842	-3.4724486	-0.4656020
H	-1.9041767	-3.7293230	-1.1334638
H	1.7101225	-1.0220841	-2.5788676
H	0.6991917	-2.3456279	-3.1515955
H	1.3753904	-2.3941425	-1.5273434
H	-0.1475825	2.2757878	-2.5682503
H	-0.2169943	1.1760644	-3.9398724
H	1.1397594	1.0942693	-2.8190389
H	-3.2735768	1.9121082	-0.5621353
H	-3.5949100	1.5937043	-2.2647324
H	-2.2107601	2.5811369	-1.8110905
H	-4.0097604	-0.1069312	0.3728120
H	-3.6066042	-1.8189411	0.5538137
H	-4.4523795	-1.2544293	-0.8893599
C	0.1674075	2.7587690	0.3240200
C	1.0153039	3.8479405	0.3638361
C	2.3873715	3.6227650	0.3542341
C	2.8555497	2.3205318	0.3361893
C	2.3764858	-0.1458782	0.3737660
H	3.0817992	4.4517642	0.3885107
H	3.9141661	2.1117559	0.3876918
H	-0.9060637	2.8706809	0.3531615
H	0.6058587	4.8468154	0.4103761
C	3.5611608	-0.5907992	-0.2170840
C	1.5825198	-1.0584863	1.0768269
C	1.9567889	-2.3921094	1.1785023
C	3.9360731	-1.9225931	-0.1099956
C	3.1372989	-2.8258333	0.5880492
H	0.7121227	-0.7350494	1.6417838
H	4.1799220	0.0971607	-0.7785218
H	4.8529366	-2.2596966	-0.5751691
H	3.4426355	-3.8601285	0.6773765
H	1.3345176	-3.0733269	1.7432483
O	-1.6301836	0.7177331	1.4891089

Co-Complex 6a

SCF energy:	–2480.386355 hartree
Zero-point correction:	+0.446875 hartree
Enthalpy correction:	+0.472504 hartree
Free energy correction:	+0.385054 hartree
Quasiharmonic free energy correction:	+0.397771 hartree

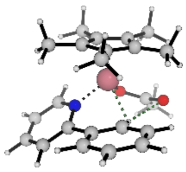

Cartesian Coordinates

Co	-0.5964977	0.0851133	-0.0509731
O	-1.5303825	-1.4324170	2.2364356
N	0.7296693	1.4878000	0.2773219
C	2.0298418	1.1378282	0.3631801
C	-0.6492988	0.4075807	-2.1195544
C	-0.3113943	-0.9391471	-1.8893355
C	0.8806301	-1.6552954	-2.4196852
C	0.1573027	1.4024522	-2.8783465
C	-1.9066755	0.6683045	-1.4594643
C	-2.3829671	-0.5792192	-0.9124272
C	-3.6815956	-0.7799159	-0.2171545
C	-2.6618244	1.9497991	-1.4494326
C	-1.3916656	-1.5510499	-1.1335298
C	-1.4516836	-2.9692679	-0.6859162
C	-1.8275671	-0.2496907	2.3762308

C	-2.6316896	0.2508736	3.5503113
H	-2.2801926	1.2279434	3.8783846
H	-2.5854074	-0.4678389	4.3647187
H	-3.6740132	0.3602353	3.2403688
H	-1.7519669	-3.0288299	0.3596699
H	-0.4921082	-3.4668009	-0.8021070
H	-2.1874335	-3.5125587	-1.2852258
H	1.6859615	-0.9697532	-2.6726125
H	0.6039149	-2.1929072	-3.3312881
H	1.2647598	-2.3851048	-1.7114062
H	0.0014692	2.4157884	-2.5130456
H	-0.1368752	1.3846278	-3.9314551
H	1.2220150	1.1808948	-2.8291770
H	-3.1129075	2.1303065	-0.4737874
H	-3.4708601	1.9102838	-2.1844579
H	-2.0299304	2.7979930	-1.7058808
H	-3.9815629	0.1121120	0.3299255
H	-3.6346812	-1.6155593	0.4771763
H	-4.4590429	-0.9929713	-0.9565918
C	0.3688753	2.7721949	0.3755519
C	1.2996585	3.7865880	0.4954802
C	2.6484775	3.4510174	0.5295874
C	3.0143838	2.1163837	0.4758319
C	2.3125447	-0.3044734	0.4007206
H	3.4038749	4.2196944	0.6248040
H	4.0504375	1.8217721	0.5584098
H	-0.6928035	2.9691393	0.3656438
H	0.9707394	4.8132736	0.5684309
C	3.4819904	-0.8514317	-0.1288457
C	1.3750128	-1.1561662	1.0008140
C	1.5965772	-2.5262734	1.0652889
C	3.7041376	-2.2202560	-0.0581766
C	2.7660651	-3.0600677	0.5392125
H	0.5216815	-0.7658467	1.5569761
H	4.2092287	-0.2134789	-0.6137877
H	4.6124516	-2.6360214	-0.4739927
H	2.9550498	-4.1236558	0.6013319
H	0.8647607	-3.1538270	1.5551060
O	-1.4925695	0.6785374	1.5183331

Transition State TS2a

SCF energy:	–2480.369915 hartree
Zero-point correction:	+0.442210 hartree
Enthalpy correction:	+0.467153 hartree
Free energy correction:	+0.380952 hartree
Quasiharmonic free energy correction:	+0.396411 hartree
Imaginary Frequency	1122.9 *i*cm^{-1}

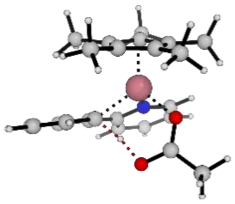

Cartesian Coordinates

Co	-0.6124703	-0.0230236	-0.0513830
O	-0.0740670	-0.4437346	2.8960294
N	0.7562553	1.3365022	0.2409869
C	2.0287705	0.8814170	0.2753085
C	-0.8391063	0.5483620	-2.0782236
C	-0.5047328	-0.8300488	-1.9900138
C	0.6429898	-1.4961954	-2.6684024
C	-0.0485015	1.5800258	-2.8051605
C	-2.0409622	0.7659778	-1.3390394
C	-2.4992996	-0.5155940	-0.8701778
C	-3.7491530	-0.7432756	-0.0965939
C	-2.7749124	2.0501705	-1.1527462
C	-1.5618130	-1.4935024	-1.2619852
C	-1.7102736	-2.9651471	-1.0784894
C	-1.1052110	0.2555451	2.7141221
C	-1.8684921	0.7460625	3.9093984
H	-2.6117810	1.4846385	3.6215652

H	-1.1765620	1.1608170	4.6415109
H	-2.3656787	-0.1060137	4.3773701
H	-2.1042169	-3.2172435	-0.0944180
H	-0.7669823	-3.4865173	-1.2157609
H	-2.4154356	-3.3519937	-1.8197598
H	1.4812043	-0.8146589	-2.8005318
H	0.3392992	-1.8422121	-3.6605192
H	0.9970596	-2.3586734	-2.1081175
H	-0.2518292	2.5845832	-2.4404662
H	-0.3096979	1.5556037	-3.8665962
H	1.0225857	1.3976103	-2.7285834
H	-3.0703591	2.1840888	-0.1113371
H	-3.6867735	2.0585682	-1.7554528
H	-2.1777405	2.9079607	-1.4572651
H	-3.9167691	0.0566475	0.6226009
H	-3.7247729	-1.6897321	0.4403679
H	-4.6044181	-0.7680936	-0.7780149
C	0.4995172	2.6427028	0.3540012
C	1.5110094	3.5787208	0.4601746
C	2.8299987	3.1360384	0.4511157
C	3.0903282	1.7787897	0.3665670
C	2.1345615	-0.5773801	0.2806081
H	3.6455109	3.8426178	0.5300164
H	4.1043755	1.4081953	0.4014124
H	-0.5430425	2.9255065	0.3618623
H	1.2675429	4.6275132	0.5506058
C	3.3036798	-1.2685640	-0.0226252
C	0.9510292	-1.2673265	0.6325408
C	1.0041808	-2.6605773	0.7101884
C	3.3174831	-2.6564874	0.0400406
C	2.1732382	-3.3530123	0.4198789
H	0.3254778	-0.8001076	1.7048594
H	4.1994531	-0.7379372	-0.3185666
H	4.2253155	-3.1956878	-0.1965737
H	2.1994486	-4.4322263	0.4986486
H	0.1328708	-3.2102739	1.0413437
O	-1.5392493	0.5549125	1.5588814

Co-Complex 7a

SCF energy:	–2480.383317 hartree
Zero-point correction:	+0.447289 hartree
Enthalpy correction:	+0.472813 hartree
Free energy correction:	+0.385321 hartree
Quasiharmonic free energy correction:	+0.401181 hartree

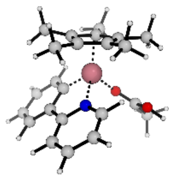

Cartesian Coordinates

Co	-0.4237080	0.1746053	-0.0862813
C	-2.4913748	0.3059180	-0.7068427
C	-1.8209968	1.5889883	-0.7316397
C	-2.2879541	2.8026155	0.0009875
C	-3.7002580	0.0014140	0.1110511
C	-1.8252310	-0.5638465	-1.5778837
C	-0.6980904	0.1551428	-2.1320854
C	0.2737723	-0.3776251	-3.1300221
C	-2.2477881	-1.9453936	-1.9511503
C	-0.7685230	1.5086619	-1.6824760
C	0.0743622	2.6248747	-2.1942411
H	1.0991528	2.3081073	-2.3784351
H	0.1046140	3.4662521	-1.5074297
H	-0.3436997	2.9784261	-3.1413126
H	-2.4542327	2.5941867	1.0589093
H	-3.2328365	3.1622724	-0.4152107
H	-1.5694465	3.6161488	-0.0715115
H	-3.8169118	-1.0675515	0.2813514
H	-4.5993770	0.3558019	-0.4011276
H	-3.6659843	0.5071439	1.0765122

H	-1.4138920	-2.5448543	-2.3117182
H	-2.9823313	-1.9009854	-2.7602190
H	-2.7174986	-2.4712162	-1.1192522
H	0.4359018	-1.4470796	-3.0017437
H	1.2384465	0.1211738	-3.0467381
H	-0.0943862	-0.2191770	-4.1476387
C	1.1756258	1.1541284	0.3576819
C	2.3441924	0.3797050	0.2805177
C	1.2804511	2.4741192	0.7719467
C	3.5973616	0.9445217	0.5396376
C	2.5265341	3.0341511	1.0450440
H	0.3954607	3.0848502	0.8919798
C	3.6870862	2.2766463	0.9095281
H	4.4979938	0.3472756	0.4739186
H	2.5924106	4.0673592	1.3632516
H	4.6530173	2.7197652	1.1114863
N	0.8052319	-1.3709758	-0.0383134
C	2.1187526	-1.0356762	0.0237102
C	0.4526799	-2.6541183	-0.1713046
C	3.1005430	-2.0193682	-0.1041412
C	1.3795794	-3.6713786	-0.2935296
H	-0.6052383	-2.8598279	-0.1723893
C	2.7315202	-3.3409036	-0.2724977
H	4.1432702	-1.7406798	-0.0677263
H	1.0472858	-4.6937097	-0.4035607
H	3.4866575	-4.1087958	-0.3775930
O	-1.5697497	-1.9064383	2.1318304
C	-1.2973829	-0.6921686	2.6171949
O	-0.8948458	0.1754596	1.8655464
C	-1.5037231	-0.4575978	4.0802288
H	-2.5311596	-0.6972903	4.3637249
H	-1.2891804	0.5796073	4.3185688
H	-0.8355844	-1.1047305	4.6541186
H	-1.8677384	-2.5076736	2.8290781

Co-Complex 8a

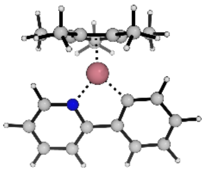

SCF energy:	–2251.272807 hartree
Zero-point correction:	+0.383521 hartree
Enthalpy correction:	+0.403980 hartree
Free energy correction:	+0.328806 hartree
Quasiharmonic free energy correction:	+0.339913 hartree

Cartesian Coordinates

Co	0.4372416	0.0928863	-0.2909154
C	2.3934093	-0.2657804	-1.0625976
C	2.2348889	1.0907585	-0.5480439
C	2.4404300	2.3241007	-1.3582256
C	2.7339359	-0.5863180	-2.4768049
C	2.1669585	-1.1641298	-0.0153042
C	1.7718156	-0.3912739	1.1380503
C	1.4136840	-0.9405295	2.4730246
C	2.3283577	-2.6461556	-0.0291343
C	1.9330058	1.0068473	0.8248179
C	1.8670832	2.1087648	1.8244162
H	0.9952726	2.0259442	2.4707992
H	1.8548977	3.0896665	1.3564983
H	2.7573988	2.0597396	2.4576337
H	1.9434418	2.2556788	-2.3267528
H	3.5066558	2.4735330	-1.5515183
H	2.0672478	3.2102759	-0.8500051
H	2.5829425	-1.6393969	-2.7058913
H	3.7836626	-0.3499311	-2.6734436
H	2.1399305	0.0053775	-3.1754131
H	1.5519171	-3.1505859	0.5451097
H	3.2858122	-2.9076512	0.4297836
H	2.3356520	-3.0534306	-1.0389973

H	0.9104023	-1.9030154	2.3909456
H	0.7674110	-0.2589124	3.0234509
H	2.3195012	-1.0929429	3.0689085
C	-0.9968041	1.2741777	0.1778112
C	-2.2433105	0.6187488	0.2667389
C	-0.9688509	2.6500204	0.3791660
C	-3.4024577	1.3159266	0.5984419
C	-2.1313690	3.3547862	0.6903787
H	-0.0447121	3.2024467	0.2889561
C	-3.3426114	2.6868953	0.8186737
H	-4.3553576	0.8080252	0.6715307
H	-2.0879009	4.4267372	0.8359201
H	-4.2409849	3.2326806	1.0744039
N	-0.9960920	-1.2293715	-0.4970384
C	-2.2212040	-0.7950792	-0.1006510
C	-0.8520457	-2.4724561	-0.9721845
C	-3.3111928	-1.6591967	-0.1274205
C	-1.9023590	-3.3680862	-1.0409898
H	0.1348353	-2.7476977	-1.3079362
C	-3.1514395	-2.9542011	-0.5931487
H	-4.2786720	-1.3128363	0.2043295
H	-1.7399122	-4.3611070	-1.4342573
H	-3.9954852	-3.6308219	-0.6185655

Co-Complex 9a

SCF energy: –3449.991874 hartree
Zero-point correction: +0.620054 hartree
Enthalpy correction: +0.656747 hartree
Free energy correction: +0.538244 hartree
Quasiharmonic free energy correction: +0.579720 hartree

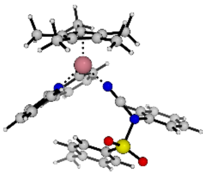

Cartesian Coordinates

Co	1.7190077	0.0058243	-0.9243368
C	2.8463372	1.6902882	-1.4159307
C	3.5820661	0.9054045	-0.4867086
C	4.1485380	1.3524369	0.8157017
C	2.4384497	3.1173903	-1.2602093
C	2.6944073	0.9140890	-2.6313510
C	3.2617940	-0.3479653	-2.4175238
C	3.4177848	-1.4442539	-3.4160905
C	2.0395322	1.4117375	-3.8746565
C	3.7488655	-0.3910844	-1.0550129
C	4.4477385	-1.5422398	-0.4135790
H	4.0799021	-2.4947884	-0.7936995
H	4.3089931	-1.5356526	0.6669096
H	5.5227323	-1.5024663	-0.6102618
H	3.6290455	2.2199762	1.2139349
H	5.1961739	1.6304746	0.6682943
H	4.1138106	0.5653411	1.5663759
H	1.4603069	3.3029047	-1.7040312
H	3.1544033	3.7780269	-1.7568892
H	2.3946765	3.4115117	-0.2129356
H	1.7764317	0.5985723	-4.5488046
H	2.7120983	2.0858549	-4.4121000
H	1.1312043	1.9696635	-3.6475907
H	2.6696690	-1.3920104	-4.2063544
H	3.3644947	-2.4290313	-2.9543676
H	4.3989577	-1.3681161	-3.8933900
C	1.2789568	0.0575856	0.9515778
C	1.0668608	-1.2183840	1.5016466
C	1.1295688	1.1652415	1.7757930
C	0.8066331	-1.3766236	2.8663128
C	0.8441524	1.0101589	3.1306524
H	1.2378342	2.1646685	1.3736761
C	0.7073558	-0.2614141	3.6826306
H	0.6641282	-2.3624790	3.2909683

H	0.7399843	1.8845944	3.7614912
H	0.5127010	-0.3774518	4.7407330
N	1.1202101	-1.8587377	-0.7568465
C	0.9943332	-2.2942759	0.5208627
C	0.8806444	-2.6873407	-1.7764116
C	0.7195130	-3.6379492	0.7737881
C	0.5671826	-4.0207291	-1.5860036
H	0.9380552	-2.2587462	-2.7654486
C	0.5135036	-4.5070655	-0.2837560
H	0.6571747	-3.9901326	1.7931511
H	0.3738945	-4.6562045	-2.4380354
H	0.2958708	-5.5504745	-0.0970658
O	-4.7457636	0.4133624	-1.9272738
H	-5.1105315	0.5290747	0.6648215
H	-4.1824125	3.0667204	-2.1283575
H	-4.9894024	4.9630545	-0.7415161
H	-4.9518603	-0.1407070	3.0400080
C	-4.3722101	-0.1918785	0.9852474
C	-4.2763054	-0.5746963	2.3141431
C	-3.7946511	3.2314373	-1.1335669
C	-4.2426211	4.2861687	-0.3486498
S	-3.4857161	-0.1575392	-1.5818209
C	-3.4965893	-0.7551259	0.0616643
O	-2.7534845	-1.0386181	-2.4384284
N	-2.4020097	1.2484231	-1.4153117
C	-2.8400758	2.3693257	-0.6121476
C	-3.3231883	-1.5056485	2.7303171
H	-4.0492111	-1.5654390	4.7587773
C	-3.7297230	4.4779823	0.9300678
H	-3.0652805	-2.9599396	4.2945794
C	-3.1853635	-1.8819805	4.1760991
H	-4.0797645	5.3059250	1.5324612
C	-1.1238126	0.9344954	-1.3958529
C	-2.5452875	-1.6995778	0.4432684
C	-2.4752280	-2.0704885	1.7727705
C	-2.3247288	2.5406376	0.6683013
N	-0.0090414	0.6436737	-1.3765681
C	-2.7684914	3.6086890	1.4353410
H	-1.8873844	-2.1350389	-0.2948012
H	-2.2982608	-1.4064619	4.6041325
H	-1.7475941	-2.8100765	2.0772080
H	-1.5917066	1.8463557	1.0592977
H	-2.3717082	3.7532319	2.4315606

Transition State TS3a

SCF energy:	–3449.954368 hartree
Zero-point correction:	+0.618917 hartree
Enthalpy correction:	+0.654885 hartree
Free energy correction:	+0.538598 hartree
Quasiharmonic free energy correction:	+0.576600 hartree
Imaginary Frequency	414.5 $i$cm^{-1}

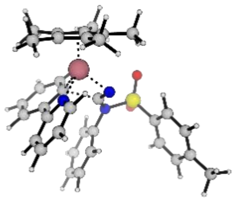

Cartesian Coordinates

Co	-1.8436200	-0.7005196	0.0619822
C	-2.4612148	-1.4097175	1.9265998
C	-3.5287478	-0.6467088	1.3286638
C	-4.2010486	0.5520753	1.9081306
C	-1.8325894	-1.1920185	3.2622846
C	-2.1807348	-2.5206286	1.0776204
C	-3.0129105	-2.4268684	-0.0655639
C	-3.1066686	-3.4410811	-1.1562677
C	-1.1439004	-3.5566203	1.3385202
C	-3.8557071	-1.2596756	0.0994154
C	-4.9124449	-0.8058114	-0.8483098
H	-4.6622439	-1.0435179	-1.8813055
H	-5.0757738	0.2687436	-0.7774362

H	-5.8603174	-1.3006542	-0.6185347
H	-3.5979203	1.0145220	2.6852581
H	-5.1583936	0.2676889	2.3528601
H	-4.4003020	1.3081955	1.1490117
H	-0.7454355	-1.2620354	3.2139098
H	-2.1756075	-1.9627862	3.9578731
H	-2.1037985	-0.2277989	3.6857655
H	-0.8406501	-4.0563436	0.4205279
H	-1.5389482	-4.3146820	2.0209489
H	-0.2585356	-3.1196716	1.7990244
H	-2.1219895	-3.7654441	-1.4955217
H	-3.6663196	-3.0690354	-2.0129789
H	-3.6311308	-4.3296626	-0.7946661
C	-1.1526442	1.2100280	0.4978483
C	-1.5054892	2.0332767	-0.5907565
C	-1.0500241	1.7863671	1.7665912
C	-1.7490530	3.3926253	-0.3964933
C	-1.3176387	3.1301329	1.9594818
H	-0.7132463	1.1857057	2.5992156
C	-1.6606972	3.9365881	0.8749331
H	-2.0366397	4.0200475	-1.2298688
H	-1.2413770	3.5596570	2.9499767
H	-1.8579703	4.9898477	1.0242108
N	-1.8550548	0.0332383	-1.7856450
C	-1.6624141	1.3666835	-1.8793093
C	-1.9379573	-0.7019242	-2.8985801
C	-1.5869345	1.9923310	-3.1219685
C	-1.8842782	-0.1403263	-4.1609315
H	-2.0402335	-1.7668467	-2.7636463
C	-1.7112457	1.2349269	-4.2733304
H	-1.4100679	3.0565539	-3.1744677
H	-1.9656718	-0.7729107	-5.0331583
H	-1.6559010	1.7049425	-5.2461924
O	3.1874376	0.4998433	2.5531410
H	4.9406736	0.4136009	0.6951069
H	1.9935017	2.7819211	2.2052911
H	2.7651925	4.9332818	1.2399928
H	6.3739969	-0.5630681	-1.0674138
C	4.6543987	-0.5013704	0.1963931
C	5.4521760	-1.0583584	-0.7886338
C	2.1084097	2.8989836	1.1384538
C	2.5430107	4.0976770	0.5894810
S	2.4329724	-0.4193723	1.7575689
C	3.4746489	-1.1506728	0.5526399
O	1.5780852	-1.4070443	2.3510752
N	1.3637327	0.5843165	0.8385904
C	1.8327725	1.8279031	0.2973857
C	5.0942224	-2.2528387	-1.4202070
H	6.4120178	-2.1023557	-3.1162121
C	2.6875714	4.2286283	-0.7878264
H	6.8287913	-3.3791464	-1.9813375
C	5.9906628	-2.8636393	-2.4581721
H	3.0233709	5.1672614	-1.2089702
C	0.2844867	-0.0235533	0.2467703
C	3.0868601	-2.3361618	-0.0634204
C	3.9029465	-2.8770876	-1.0436734
C	1.9875981	1.9456800	-1.0807914
N	0.0247582	-1.1025353	-0.2510070
C	2.4092393	3.1516257	-1.6227087
H	2.1620707	-2.8148727	0.2212161
H	5.4578887	-3.5927906	-3.0679441
H	3.6130897	-3.8036901	-1.5231479
H	1.7827466	1.0937859	-1.7164666
H	2.5341552	3.2452962	-2.6936345

Co-Complex 10a

SCF energy:	–3449.994071 hartree	
Zero-point correction:	+0.621465 hartree	
Enthalpy correction:	+0.657482 hartree	
Free energy correction:	+0.540655 hartree	
Quasiharmonic free energy correction:	+0.578870 hartree	

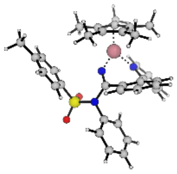

Cartesian Coordinates

Co	1.9341228	-0.6531344	-0.1103320
C	2.7838194	-0.4555877	-1.9881679
C	3.5430548	0.2253671	-0.9783522
C	3.8743575	1.6756794	-0.9647530
C	2.1051934	0.1682476	-3.1584113
C	2.7184944	-1.8197906	-1.6179522
C	3.5177751	-2.0130817	-0.4191402
C	3.7318887	-3.3250312	0.2582613
C	1.9587235	-2.8825584	-2.3345541
C	4.0282008	-0.7615321	-0.0365319
C	4.8928452	-0.4598214	1.1407403
H	4.8425055	-1.2439094	1.8942838
H	4.6113428	0.4812127	1.6128815
H	5.9365230	-0.3694417	0.8288722
H	3.0705097	2.2770127	-1.3850775
H	4.7720337	1.8547043	-1.5644374
H	4.0757447	2.0349737	0.0426319
H	1.1222038	-0.2701329	-3.3273724
H	2.7033773	0.0180957	-4.0609521
H	1.9796228	1.2405858	-3.0186033
H	1.6476077	-3.6786416	-1.6584675
H	2.5886379	-3.3382888	-3.1041246
H	1.0732145	-2.4767822	-2.8214992
H	2.8089622	-3.9034265	0.3189294
H	4.1272094	-3.2034233	1.2655621
H	4.4503259	-3.9277476	-0.3044427
C	0.1804033	2.0945668	-0.4002335
C	0.9429824	2.3232011	0.7585399
C	0.0028941	3.1177455	-1.3275757
C	1.5057239	3.5889849	0.9476900
C	0.5937614	4.3584878	-1.1381675
H	-0.6071585	2.9266081	-2.1999768
C	1.3485738	4.5937177	0.0046809
H	2.0868082	3.7808112	1.8404021
H	0.4592870	5.1397329	-1.8743887
H	1.8094038	5.5592216	0.1661052
N	1.6008704	0.1026825	1.6623987
C	1.1015174	1.3434728	1.8638270
C	1.7737995	-0.7216606	2.7085006
C	0.7620790	1.7501758	3.1565480
C	1.4622649	-0.3675301	4.0048493
H	2.1724723	-1.6996891	2.4775505
C	0.9386296	0.8983953	4.2322370
H	0.3393945	2.7342927	3.2948114
H	1.6207518	-1.0720267	4.8086616
H	0.6656049	1.2141389	5.2303668
O	-3.6217227	-0.1139421	-2.4888008
H	-4.6348323	-0.9703081	-0.2663249
H	-1.9983493	0.6632723	1.5493494
H	-3.5210771	2.0214382	2.9577415
H	-5.0604758	-2.5826967	1.5566624
C	-3.8987120	-1.7199816	-0.0138250
C	-4.1308275	-2.6317185	1.0032665
C	-2.6725423	1.3792558	1.0971423
C	-3.5316855	2.1365715	1.8814738
S	-2.3748828	-0.5750274	-1.9563614
C	-2.7019122	-1.7881096	-0.7225512
O	-1.3129205	-1.0130530	-2.8159862
N	-1.7637923	0.7599679	-1.0768588

C	-2.6869004	1.5267296	-0.2872278
C	-3.1942333	-3.6194055	1.3173672
H	-4.0352715	-4.1845390	3.2185463
C	-4.4156310	3.0260614	1.2811180
H	-4.0884407	-5.4465704	1.9950217
C	-3.4799500	-4.6292823	2.3919098
H	-5.0905280	3.6135794	1.8896147
C	-0.4167336	0.7542339	-0.6504162
C	-1.7517290	-2.7591370	-0.4306622
C	-2.0068565	-3.6686845	0.5835637
C	-3.5687071	2.4135630	-0.8931854
N	0.2701863	-0.2878566	-0.5305134
C	-4.4352839	3.1576341	-0.1042102
H	-0.8346293	-2.7934135	-0.9978336
H	-2.5619074	-5.0657881	2.7859917
H	-1.2769488	-4.4378880	0.8061365
H	-3.5778048	2.5016419	-1.9696953
H	-5.1255588	3.8466168	-0.5728707

Co-Complex 11a

SCF energy:	–3679.076707 hartree	
Zero-point correction:	+0.685219 hartree	
Enthalpy correction:	+0.726140 hartree	
Free energy correction:	+0.600335 hartree	
Quasiharmonic free energy correction:	+0.631714 hartree	

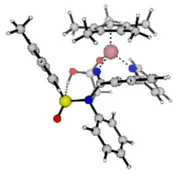

Cartesian Coordinates

Co	-1.7456155	-0.8138846	-0.0677341
C	-2.2863489	-1.3218769	1.8338224
C	-3.4463486	-1.5388182	0.9931027
C	-4.7579168	-0.8508862	1.1653478
C	-2.2802638	-0.5174003	3.0854054
C	-1.2504054	-2.2091816	1.3947977
C	-1.7201236	-2.8749689	0.2396388
C	-0.9775032	-3.8683250	-0.5829298
C	0.0883148	-2.3575845	2.0268619
C	-3.1043140	-2.4860446	0.0229290
C	-3.9708047	-3.0211741	-1.0620087
H	-3.4178519	-3.1196481	-1.9935724
H	-4.8362628	-2.3857885	-1.2420220
H	-4.3404129	-4.0118951	-0.7816532
H	-4.6279228	0.1977053	1.4326154
H	-5.3296556	-1.3233751	1.9685889
H	-5.3614982	-0.8978993	0.2607201
H	-1.2811401	-0.1836033	3.3543001
H	-2.6597343	-1.1314897	3.9084801
H	-2.9229528	0.3569548	3.0088713
H	0.8519076	-2.5988620	1.2930145
H	0.0610355	-3.1641940	2.7650369
H	0.3865566	-1.4473017	2.5429012
H	0.0946671	-3.7841839	-0.4270258
H	-1.1763905	-3.7282403	-1.6437433
H	-1.2818075	-4.8831628	-0.3101638
C	0.2376017	1.3605897	1.3979451
C	-0.9903253	2.0301144	1.4785767
C	1.1076295	1.3886247	2.4871955
C	-1.3002695	2.7251849	2.6533643
C	0.7726378	2.0523417	3.6564148
H	2.0638123	0.8918217	2.3984443
C	-0.4382113	2.7302762	3.7375398
H	-2.2435361	3.2510413	2.7214354
H	1.4575021	2.0538369	4.4939901
H	-0.7087979	3.2633657	4.6391784
N	-2.4195118	1.0340660	-0.2820783
C	-1.9620488	2.1282096	0.3657010
C	-3.3868494	1.1750114	-1.2054659

C	-2.4542888	3.3936665	0.0340460
C	-3.9302648	2.3965179	-1.5473763
H	-3.7011454	0.2668240	-1.6948634
C	-3.4411070	3.5359714	-0.9236248
H	-2.0376761	4.2564610	0.5319377
H	-4.7079638	2.4451754	-2.2960700
H	-3.8198549	4.5166029	-1.1796154
O	2.2537493	-0.2414734	-2.5336338
H	4.0172117	-0.4062269	1.2966186
H	0.3924336	2.7367174	-1.5619632
H	0.6426453	5.2004634	-1.6785743
H	4.3951570	-2.4744558	2.5861043
C	3.6811894	-1.3589567	0.9111260
C	3.9002553	-2.5234399	1.6238263
C	1.2289198	3.2265924	-1.0815106
C	1.3714477	4.6065918	-1.1420335
S	2.9056695	0.0564087	-1.2729378
C	3.0483405	-1.4255231	-0.3285026
O	4.1798192	0.7093997	-1.3313380
N	1.9565532	1.0389429	-0.2819086
C	2.1640764	2.4629942	-0.3915537
C	3.5159068	-3.7675395	1.1097833
H	4.7900748	-5.0668232	2.2550958
C	2.4571640	5.2183949	-0.5268758
H	3.1113894	-5.0623608	2.7748510
C	3.7604599	-5.0233295	1.8958876
H	2.5752974	6.2928093	-0.5788668
C	0.6496370	0.6297741	0.1651563
C	2.6591959	-2.6443980	-0.8624037
C	2.9015847	-3.8068347	-0.1407086
C	3.2502502	3.0696741	0.2257067
N	-0.0143928	-0.2607236	-0.4096116
C	3.3968851	4.4475443	0.1506984
H	2.1780653	-2.6887139	-1.8269520
H	3.5694009	-5.9132666	1.2975237
H	2.6118371	-4.7613533	-0.5619113
H	3.9763675	2.4619456	0.7455160
H	4.2455254	4.9219842	0.6254816
O	-1.9599938	-1.1284868	-2.3666637
C	-0.9221504	-0.8090372	-2.9343422
O	0.0541096	-1.6888067	-3.0462449
C	-0.7044108	0.5337073	-3.5648680
H	0.2654012	0.9321680	-3.2694517
H	-1.5012121	1.2174855	-3.2889713
H	-0.6927889	0.4153107	-4.6510496
H	0.9160203	-1.2230286	-3.1378454

Co-Complex 12a

SCF energy:	–2572.638675 hartree
Zero-point correction:	+0.444800 hartree
Enthalpy correction:	+0.472257 hartree
Free energy correction:	+0.379848 hartree
Quasiharmonic free energy correction:	+0.398169 hartree

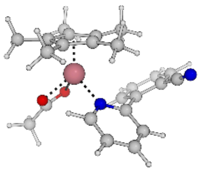

Cartesian Coordinates

Co	-0.7996000	-0.0020234	0.3914392
O	0.4185421	-0.9022492	1.6722451
O	-0.9085237	0.6951044	2.2270736
N	0.5369820	1.4732852	-0.0614384
C	1.8091202	1.3873197	-0.5141074
C	-2.3831961	0.4241698	-0.9322182
C	-1.4766615	-0.5033627	-1.5722136
C	-0.8645444	-0.2844040	-2.9154428
C	-2.8248995	1.7302489	-1.5100630
C	-2.9011385	-0.2085193	0.2321424
C	-2.2504727	-1.4728037	0.3770830

C	-2.4950645	-2.4736666	1.4580658
C	-3.8942244	0.3624445	1.1858534
C	-1.3927319	-1.6643194	-0.7688305
C	-0.5714950	-2.8852543	-1.0140814
C	-0.0287887	-0.1512070	2.5964953
C	0.4429964	-0.2391890	4.0048464
H	-0.2904158	0.1980875	4.6792393
H	1.3827310	0.3121581	4.0895593
H	0.6426537	-1.2769752	4.2690110
H	-0.0266525	-3.1792613	-0.1160368
H	0.1497927	-2.7397106	-1.8151375
H	-1.2207019	-3.7205353	-1.2927040
H	-0.4358780	0.7132219	-3.0157880
H	-1.6408924	-0.3764756	-3.6812117
H	-0.0957101	-1.0190506	-3.1388367
H	-3.1924433	2.4144462	-0.7464318
H	-3.6434145	1.5675082	-2.2180291
H	-2.0228373	2.2277146	-2.0546161
H	-3.5937612	0.2030703	2.2209402
H	-4.8610463	-0.1289581	1.0411041
H	-4.0405828	1.4302227	1.0359264
H	-2.8331302	-1.9984987	2.3782668
H	-1.5986595	-3.0512323	1.6804639
H	-3.2738086	-3.1776608	1.1492411
C	0.0263333	2.6976921	0.1720241
C	0.7166241	3.8713315	-0.0604244
C	2.0091075	3.7919077	-0.5551868
C	2.5528360	2.5374352	-0.7751549
C	2.4856057	0.0701817	-0.7227269
H	2.5883468	4.6847855	-0.7514906
H	3.5664194	2.4288038	-1.1352306
H	-0.9732328	2.7232376	0.5791424
H	0.2451141	4.8204044	0.1533261
C	3.2305187	-0.5194542	0.3157441
C	2.5297455	-0.5162213	-1.9840252
C	3.2488237	-1.6868220	-2.2047985
C	3.9466826	-1.6988853	0.0927673
C	3.9475022	-2.2854235	-1.1637054
H	2.0192123	-0.0380820	-2.8086194
H	4.5108249	-2.1351110	0.9060221
H	4.5112637	-3.1931482	-1.3339358
H	3.2761785	-2.1203505	-3.1964730
C	3.3103380	0.1149361	1.5949582
N	3.3939029	0.6464379	2.6139983

2-Phenylpyridine (1a)

SCF energy: –479.257226 hartree
Zero-point correction: +0.169338 hartree
Enthalpy correction: +0.177677 hartree
Free energy correction: +0.133022 hartree
Quasiharmonic free energy correction: +0.135641 hartree

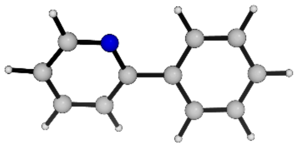

Cartesian Coordinates

C	2.8467963	1.1671023	-0.2177376
C	1.4587239	1.1905461	-0.2245997
C	3.5243589	-0.0250252	0.0092096
C	2.8019101	-1.1947946	0.2205986
C	1.4152651	-1.1728124	0.2087030
C	0.7223080	0.0223469	-0.0068442
C	-0.7594802	0.0243571	-0.0043900
C	-1.4971113	1.1882017	0.2421389
N	-1.3672731	-1.1485217	-0.2337845
C	-2.6954887	-1.1921246	-0.2429001
C	-3.5034534	-0.0826280	-0.0267097
C	-2.8810462	1.1322857	0.2259839

H	0.9495961	2.1233886	-0.4271137
H	3.3996930	2.0805502	-0.3972660
H	4.6068111	-0.0433334	0.0159218
H	3.3219913	-2.1283010	0.3956693
H	0.8459035	-2.0780935	0.3638608
H	-3.4652010	2.0238362	0.4171218
H	-0.9943628	2.1185918	0.4638181
H	-3.1386852	-2.1643095	-0.4346893
H	-4.5811055	-0.1735528	-0.0491907

***N*-Cyano-4-methyl-*N*-phenylbenzenesulfonamide (2a)**

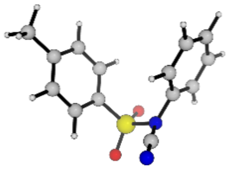

SCF energy:	–1198.697514 hartree
Zero-point correction:	+0.233971 hartree
Enthalpy correction:	+0.250032 hartree
Free energy correction:	+0.184152 hartree
Quasiharmonic free energy correction:	+0.201317 hartree

Cartesian Coordinates

N	-1.2722770	-1.3454970	-0.4670035
C	-1.8972562	-0.0450972	-0.4197056
C	-2.8772446	0.1950418	0.5347658
C	-3.4593900	1.4538164	0.6034572
C	-3.0754658	2.4550941	-0.2822785
C	-2.1006764	2.1994948	-1.2401371
C	-1.5012586	0.9491826	-1.3072845
H	-3.1642830	-0.5937101	1.2140419
H	-4.2224461	1.6481265	1.3457952
H	-3.5397147	3.4313980	-0.2295182
H	-1.8002765	2.9743064	-1.9333530
H	-0.7379520	0.7392725	-2.0450842
C	1.2035152	-0.6642520	0.4391571
C	2.1705108	-1.0550010	-0.4837116
C	1.2272328	0.5998989	1.0185723
C	2.2420260	1.4778393	0.6700529
C	3.2277400	1.1136765	-0.2485907
C	3.1747952	-0.1616102	-0.8176449
H	3.9312265	-0.4582125	-1.5337707
H	2.1302065	-2.0408118	-0.9248140
H	0.4643273	0.8814870	1.7300991
H	2.2676288	2.4635458	1.1178091
C	4.3400116	2.0603518	-0.6007687
H	4.0604960	3.0951654	-0.4025405
H	4.6171608	1.9721305	-1.6520671
H	5.2328033	1.8397887	-0.0087238
S	-0.1329332	-1.7503331	0.7905493
O	-0.7695583	-1.3505980	2.0078405
O	0.2424843	-3.1026247	0.5212500
C	-0.9903678	-1.8603465	-1.6718595
N	-0.7500749	-2.2957428	-2.7142142

Acetic Acid

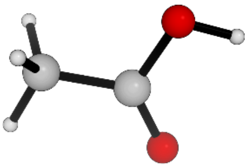

SCF energy:	–229.097386 hartree
Zero-point correction:	+0.061502 hartree
Enthalpy correction:	+0.066498 hartree
Free energy correction:	+0.033750 hartree
Quasiharmonic free energy correction:	+0.034724 hartree

Cartesian Coordinates

C	-1.3930025	-0.1076533	0.0000039
C	0.0903931	0.1246858	0.0000651
H	-1.6760606	-0.6890686	-0.8788890
H	-1.9111177	0.8469029	-0.0004550
H	-1.6761644	-0.6881938	0.8794517
O	0.6399902	1.1944858	0.0005002
O	0.7771299	-1.0447711	-0.0003740
H	1.7203318	-0.8169479	-0.0000830

2-(Pyridin-2-yl)benzonitrile (3a)

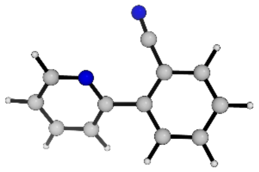

SCF energy:	–571.494940 hartree
Zero-point correction:	+0.167853 hartree
Enthalpy correction:	+0.178016 hartree
Free energy correction:	+0.129099 hartree
Quasiharmonic free energy correction:	+0.132128 hartree

Cartesian Coordinates

C	-2.6326341	-1.7641711	-0.2541770
C	-1.2490443	-1.6621665	-0.2221343
C	-3.4179512	-0.6263783	-0.1131883
C	-2.8122348	0.6069485	0.0644320
C	-1.4180449	0.7153833	0.0941704
C	-0.6155254	-0.4310574	-0.0556743
C	0.8626056	-0.3485807	-0.0514946
C	1.6369011	-1.3002624	0.6168540
N	1.4085255	0.6734876	-0.7169769
C	2.7330831	0.7810000	-0.7394049
C	3.5834857	-0.1204953	-0.1096027
C	3.0177940	-1.1817374	0.5834471
H	-0.6430340	-2.5482806	-0.3578425
H	-3.0968244	-2.7311872	-0.3985312
H	-4.4971523	-0.6981605	-0.1395077
H	-3.4082500	1.5004306	0.1907133
C	-0.8646984	2.0116536	0.3434673
H	3.6412976	-1.9009009	1.0993702
H	1.1625364	-2.0996274	1.1694544
H	3.1329653	1.6277060	-1.2870433
H	4.6558166	0.0127422	-0.1584052
N	-0.5127272	3.0796540	0.5947144

4-Methyl-*N*-phenylbenzenesulfonamide

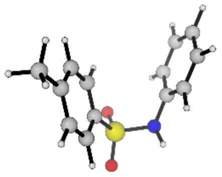

SCF energy:	–1106.487057 hartree
Zero-point correction:	+0.235984 hartree
Enthalpy correction:	+0.250196 hartree
Free energy correction:	+0.188284 hartree
Quasiharmonic free energy correction:	+0.205002 hartree

Cartesian Coordinates

N	1.3517381	1.2395559	-0.8297273
C	1.9139523	-0.0238908	-0.5046228
C	2.6077963	-0.1994392	0.6912539
C	3.1456327	-1.4426235	0.9948727
C	3.0205273	-2.5062624	0.1071902
C	2.3420543	-2.3225206	-1.0915510
C	1.7787524	-1.0896292	-1.3921821
H	2.7126553	0.6348537	1.3689698
H	3.6807561	-1.5752747	1.9266103

H	3.4529799	-3.4691875	0.3459821
H	2.2374489	-3.1429167	-1.7900461
H	1.2249142	-0.9522088	-2.3134325
C	-1.1305532	0.6808666	0.1527499
C	-1.0762606	-0.3364690	1.0993381
C	-2.1344912	0.7035376	-0.8095762
C	-3.0887342	-0.3021501	-0.8178575
C	-3.0540397	-1.3376242	0.1186803
C	-2.0353502	-1.3380964	1.0722016
H	-1.9924183	-2.1335379	1.8062352
H	-0.2957122	-0.3353003	1.8461358
H	-2.1685288	1.5102850	-1.5288538
H	-3.8764902	-0.2820200	-1.5614437
C	-4.1098481	-2.4075764	0.1189327
H	-4.4598620	-2.6206695	-0.8919378
H	-4.9775819	-2.0924359	0.7056748
H	-3.7368592	-3.3338824	0.5568628
S	0.1425078	1.9107818	0.1143937
O	0.6647345	2.0501347	1.4431329
O	-0.3206221	3.0383892	-0.6454173
H	1.1106215	1.3482648	-1.8069059

6 Optimization with M06-L/def2-TZVPP

Co-Complex 4

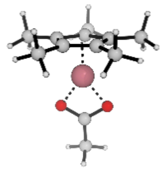

SCF energy:	–2001.485066 hartree
Zero-point correction:	+0.274985 hartree
Enthalpy correction:	+0.292417 hartree
Free energy correction:	+0.224738 hartree
Quasiharmonic free energy correction:	+0.235195 hartree

Cartesian Coordinates

Co	-0.2778427	0.0000770	-0.2045208
O	-1.8634705	-1.0804443	-0.3747175
O	-1.8636561	1.0804262	-0.3736533
C	1.1013894	1.1705383	0.6779648
C	0.7704765	-0.0015187	1.4525995
C	0.1911427	-0.0033273	2.8102137
C	0.8689573	2.5669713	1.1077560
C	1.5630064	0.7281438	-0.5809671
C	1.5635308	-0.7259002	-0.5826273
C	1.9326576	-1.5634655	-1.7441854
C	1.9319369	1.5689225	-1.7402777
C	1.1022352	-1.1715387	0.6752720
C	0.8705650	-2.5690781	1.1019433
C	-2.5371842	-0.0000170	-0.5021043
C	-3.9795302	0.0000370	-0.7996667
H	-4.4522167	0.8944379	-0.4084596
H	-4.4516966	-0.8960052	-0.4116721
H	-4.1118408	0.0020595	-1.8813624
H	0.8890747	-3.2593553	0.2647354
H	-0.0863254	-2.6801947	1.6070188
H	1.6438732	-2.8789129	1.8054906
H	-0.4141804	0.8793946	2.9979885
H	0.9954985	-0.0026639	3.5494658
H	-0.4117002	-0.8880046	2.9967116
H	0.8823109	3.2585702	0.2715070
H	1.6450179	2.8772671	1.8080919
H	-0.0857370	2.6755300	1.6174882
H	1.5741475	1.1497023	-2.6781190
H	3.0186163	1.6294244	-1.8219703
H	1.5529731	2.5820192	-1.6515287
H	1.5704612	-1.1441947	-2.6803535
H	1.5579470	-2.5781616	-1.6560213
H	3.0193224	-1.6193087	-1.8291499

Co-Complex 5a

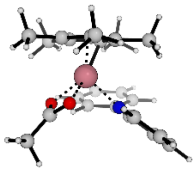

SCF energy:	–2481.025551 hartree
Zero-point correction:	+0.449939 hartree
Enthalpy correction:	+0.474732 hartree
Free energy correction:	+0.388090 hartree
Quasiharmonic free energy correction:	+0.406696 hartree

Cartesian Coordinates

Co	-0.9301795	-0.0594515	0.0271061
O	-0.8660000	-0.6149677	1.9577917
O	-2.1239974	0.9824954	1.2480282
N	0.4694825	1.4227511	0.1822967
C	1.8220748	1.3596689	0.2450381
C	-1.0359735	-0.0097407	-2.0085519
C	-0.2320001	-1.1321563	-1.5884711
C	1.1249090	-1.4426940	-2.0936734
C	-0.5746999	1.0739036	-2.9089778
C	-2.3279418	-0.1591407	-1.4456238

C	-2.3246695	-1.3388201	-0.6235441
C	-3.4624995	-1.8522780	0.1753683
C	-3.4613801	0.7828732	-1.5737872
C	-1.0344285	-1.9489127	-0.7515763
C	-0.5982388	-3.1742496	-0.0468339
C	-1.7079285	0.2929372	2.2317138
C	-2.1633701	0.5528387	3.6174177
H	-3.1859157	0.9157458	3.6254850
H	-1.5365728	1.3277533	4.0560677
H	-2.0687314	-0.3377898	4.2288542
H	-0.9224173	-3.1723022	0.9918173
H	0.4816168	-3.2896520	-0.0663727
H	-1.0385538	-4.0524919	-0.5207884
H	1.6930983	-0.5448689	-2.3210442
H	1.0388957	-2.0095730	-3.0227573
H	1.7066857	-2.0407734	-1.3966054
H	-1.1850718	1.9688509	-2.8228037
H	-0.6244282	0.7471282	-3.9483183
H	0.4592758	1.3500108	-2.7115501
H	-3.9637820	0.9363529	-0.6215498
H	-4.1996620	0.3801411	-2.2681278
H	-3.1495799	1.7510767	-1.9547923
H	-4.0924522	-1.0452506	0.5413176
H	-3.1221634	-2.4311619	1.0301727
H	-4.0931879	-2.5065395	-0.4277543
C	-0.1116983	2.6315229	0.0540017
C	0.5895974	3.8134692	-0.0195713
C	1.9717351	3.7624482	0.0504768
C	2.5785652	2.5330486	0.1848922
C	2.5247633	0.0728909	0.3659248
H	2.5642893	4.6649230	0.0095474
H	3.6515028	2.4565445	0.2721113
H	-1.1909691	2.6288055	0.0112651
H	0.0565657	4.7456601	-0.1240035
C	3.7028844	-0.1229090	-0.3616405
C	2.0777927	-0.9469113	1.2041548
C	2.7842567	-2.1360494	1.2970432
C	4.3972517	-1.3160595	-0.2772182
C	3.9379304	-2.3295543	0.5523653
H	1.2004313	-0.7985661	1.8130211
H	4.0574555	0.6542303	-1.0260322
H	5.2971366	-1.4555387	-0.8587697
H	4.4849890	-3.2583151	0.6279344
H	2.4376570	-2.9080937	1.9700461

Transition State TS1a

SCF energy:	–2481.011537 hartree
Zero-point correction:	+0.448620 hartree
Enthalpy correction:	+0.472939 hartree
Free energy correction:	+0.389598 hartree
Quasiharmonic free energy correction:	+0.398345 hartree
Imaginary Frequency	68.8 *i*cm^{-1}

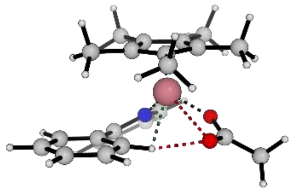

Cartesian Coordinates

Co	-0.6652789	0.0337311	-0.0575626
O	-1.3840662	-1.2992418	2.1736137
N	0.6706393	1.4846400	0.2113736
C	1.9939726	1.2186249	0.2658933
C	-0.7531212	0.3186028	-2.0729301
C	-0.2623218	-0.9811443	-1.8012639
C	0.9971111	-1.5723178	-2.3125430
C	-0.0530257	1.3708190	-2.8436990
C	-2.0308463	0.4611946	-1.4273994
C	-2.3603308	-0.8049274	-0.8215866
C	-3.6096690	-1.1099229	-0.0898754
C	-2.8964674	1.6610226	-1.4159710

C	-1.2715660	-1.6764259	-1.0336609
C	-1.1707421	-3.0635836	-0.5303880
C	-1.8252578	-0.1568551	2.3262064
C	-2.6587147	0.2268190	3.5089753
H	-2.6304365	1.2941442	3.6996596
H	-2.3417891	-0.3254452	4.3869685
H	-3.6934870	-0.0493329	3.3083318
H	-1.5135696	-3.1337618	0.4992328
H	-0.1547080	-3.4430607	-0.5845729
H	-1.8007849	-3.7174320	-1.1357363
H	1.7318403	-0.8118671	-2.5626340
H	0.8000155	-2.1431887	-3.2213237
H	1.4510471	-2.2535536	-1.5960805
H	-0.3443932	2.3708459	-2.5327171
H	-0.3029888	1.2782412	-3.9012518
H	1.0282517	1.2882003	-2.7606027
H	-3.2968770	1.8456466	-0.4208350
H	-3.7472174	1.5196723	-2.0837791
H	-2.3698434	2.5515911	-1.7479275
H	-3.9771377	-0.2411607	0.4512462
H	-3.4719979	-1.9221162	0.6173290
H	-4.3890328	-1.4047885	-0.7940680
C	0.2433973	2.7526698	0.2375242
C	1.1115742	3.8239624	0.2569590
C	2.4747998	3.5693745	0.2577438
C	2.9160738	2.2611564	0.2743764
C	2.3751988	-0.1885290	0.3803647
H	3.1860651	4.3825675	0.2719115
H	3.9699079	2.0337368	0.3315011
H	-0.8299209	2.8854308	0.2502456
H	0.7238014	4.8305529	0.2759668
C	3.5856269	-0.6792183	-0.1066050
C	1.5020633	-1.0682380	1.0264915
C	1.8184101	-2.4091441	1.1712947
C	3.9011242	-2.0202292	0.0368923
C	3.0219163	-2.8876297	0.6748696
H	0.6150884	-0.7013640	1.5427582
H	4.2711502	-0.0176288	-0.6184570
H	4.8384435	-2.3924796	-0.3507217
H	3.2841576	-3.9286733	0.7961130
H	1.1352929	-3.0581372	1.7013930
O	-1.6187894	0.7801832	1.4509394

Co-Complex 6a

SCF energy:	–2481.004918 hartree
Zero-point correction:	+0.447490 hartree
Enthalpy correction:	+0.472470 hartree
Free energy correction:	+0.387298 hartree
Quasiharmonic free energy correction:	+0.398077 hartree

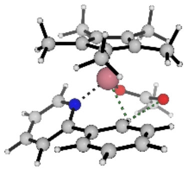

Cartesian Coordinates

Co	-0.5906362	0.0620276	-0.1254027
O	-1.2645900	-1.2620404	2.3098536
N	0.7517111	1.4639938	0.2048391
C	2.0373765	1.0891063	0.3643444
C	-0.7092135	0.4458955	-2.1330551
C	-0.3158597	-0.8976553	-1.9262159
C	0.8914083	-1.5524535	-2.4844042
C	0.0574803	1.4712454	-2.8775403
C	-1.9528051	0.6630310	-1.4452449
C	-2.3668274	-0.6002547	-0.8870689
C	-3.6118681	-0.8397838	-0.1235872
C	-2.7340162	1.9190124	-1.3729966
C	-1.3603752	-1.5470315	-1.1613186
C	-1.3898066	-2.9650899	-0.7333691
C	-1.7254892	-0.1197031	2.3559765

C	-2.5950336	0.3378090	3.4876095
H	-2.6599649	1.4184847	3.5462332
H	-2.2331198	-0.0731820	4.4243914
H	-3.5985663	-0.0562159	3.3321745
H	-1.5943730	-3.0505850	0.3328162
H	-0.4565292	-3.4759425	-0.9485988
H	-2.1865938	-3.4929039	-1.2585258
H	1.7052159	-0.8474508	-2.6335026
H	0.6647546	-1.9932833	-3.4560604
H	1.2552711	-2.3513424	-1.8431565
H	-0.1688287	2.4790253	-2.5394459
H	-0.1972643	1.4234435	-3.9368671
H	1.1312726	1.3180700	-2.7990547
H	-3.0771145	2.1079268	-0.3571703
H	-3.6207384	1.8513722	-2.0044753
H	-2.1633355	2.7795071	-1.7108921
H	-3.9259064	0.0483046	0.4184351
H	-3.4972444	-1.6545111	0.5861124
H	-4.4192478	-1.1070964	-0.8068625
C	0.4166683	2.7566411	0.2610995
C	1.3570621	3.7520023	0.4242309
C	2.6908325	3.3891938	0.5430756
C	3.0306203	2.0511969	0.5232883
C	2.2810448	-0.3497256	0.4142392
H	3.4543349	4.1420875	0.6736690
H	4.0542463	1.7384838	0.6622707
H	-0.6395337	2.9743292	0.1833061
H	1.0458067	4.7841139	0.4638344
C	3.5166020	-0.8984233	0.0723142
C	1.2504881	-1.2087553	0.8192298
C	1.4710037	-2.5758732	0.9074307
C	3.7248406	-2.2649045	0.1439140
C	2.7070439	-3.1065863	0.5729449
H	0.2365241	-0.8897621	1.3656009
H	4.3146534	-0.2534212	-0.2689123
H	4.6868085	-2.6740262	-0.1282404
H	2.8818188	-4.1693236	0.6591582
H	0.6818348	-3.2115871	1.2821557
O	-1.5159283	0.7613222	1.4250096

Transition State TS2a

SCF energy:	–2480.990388 hartree
Zero-point correction:	+0.443328 hartree
Enthalpy correction:	+0.468067 hartree
Free energy correction:	+0.382342 hartree
Quasiharmonic free energy correction:	+0.397603 hartree
Imaginary Frequency	1397.4 *i*cm^{-1}

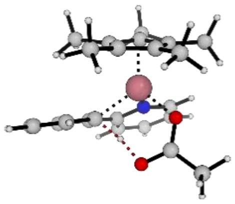

Cartesian Coordinates

Co	-0.6425566	-0.0343987	-0.0974565
O	-0.0775002	-0.3909872	2.8553395
N	0.7651918	1.3146841	0.2115978
C	2.0309421	0.8395073	0.2554444
C	-0.8743599	0.5852662	-2.0627909
C	-0.4941409	-0.7858102	-1.9969462
C	0.6812030	-1.4023599	-2.6600170
C	-0.1138982	1.6496903	-2.7586991
C	-2.0758352	0.7574345	-1.3178003
C	-2.4890392	-0.5438686	-0.8617324
C	-3.7154274	-0.8192256	-0.0803517
C	-2.8301574	2.0133499	-1.0884637
C	-1.5303877	-1.4904007	-1.2837642
C	-1.6402363	-2.9608538	-1.1326621
C	-1.1068245	0.3100846	2.6741023
C	-1.8450589	0.8193706	3.8671219
H	-2.5918245	1.5534262	3.5887068

H	-1.1479871	1.2419314	4.5842134
H	-2.3366860	-0.0170127	4.3601577
H	-2.0242931	-3.2521950	-0.1582395
H	-0.6897563	-3.4618024	-1.2888836
H	-2.3387476	-3.3477357	-1.8764528
H	1.5008687	-0.6961232	-2.7707213
H	0.4191400	-1.7512456	-3.6596399
H	1.0542988	-2.2583840	-2.1021041
H	-0.3469646	2.6409090	-2.3797528
H	-0.3669416	1.6429247	-3.8195236
H	0.9615104	1.5012645	-2.6887499
H	-3.1103918	2.1172453	-0.0413989
H	-3.7531436	2.0213197	-1.6688800
H	-2.2621707	2.8924991	-1.3805613
H	-3.9062205	-0.0383609	0.6516224
H	-3.6626217	-1.7708500	0.4409122
H	-4.5787512	-0.8588759	-0.7460921
C	0.5351634	2.6266456	0.3072209
C	1.5603747	3.5448855	0.4041849
C	2.8681653	3.0797460	0.4001771
C	3.1048612	1.7210573	0.3336164
C	2.1111677	-0.6118620	0.2818291
H	3.6942967	3.7725682	0.4689741
H	4.1117619	1.3333913	0.3700312
H	-0.5040239	2.9278615	0.3074970
H	1.3355047	4.5970548	0.4821398
C	3.2817089	-1.3205813	0.0359721
C	0.9058801	-1.2810474	0.6063641
C	0.9496121	-2.6729619	0.7092913
C	3.2796418	-2.7032383	0.1176788
C	2.1175189	-3.3796595	0.4674664
H	0.2949064	-0.7806134	1.6647172
H	4.1931142	-0.8004895	-0.2272470
H	4.1883127	-3.2545140	-0.0768905
H	2.1297609	-4.4560294	0.5656467
H	0.0668345	-3.2120741	1.0266579
O	-1.5569121	0.5937807	1.5223402

Co-Complex 7a

SCF energy:	–2481.001051 hartree
Zero-point correction:	+0.449422 hartree
Enthalpy correction:	+0.474550 hartree
Free energy correction:	+0.388772 hartree
Quasiharmonic free energy correction:	+0.402011 hartree

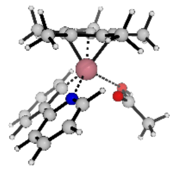

Cartesian Coordinates

Co	-0.4491268	-0.2223802	-0.0923021
C	-2.4399490	-0.7644760	-0.4081678
C	-2.0514036	0.3164364	-1.2597553
C	-2.7963542	1.5904418	-1.4282816
C	-3.5420112	-0.7223444	0.5832553
C	-1.6074506	-1.8827613	-0.6512947
C	-0.6772892	-1.4939984	-1.6704379
C	0.3804668	-2.3452323	-2.2663920
C	-1.7678181	-3.2265522	-0.0362933
C	-0.9598112	-0.1492951	-2.0611399
C	-0.2947169	0.5815931	-3.1674647
H	0.7655244	0.3478262	-3.2291454
H	-0.3838860	1.6584077	-3.0569126
H	-0.7455086	0.3054856	-4.1214872
H	-3.0190963	2.0707609	-0.4781963
H	-3.7493387	1.4051954	-1.9261048
H	-2.2495829	2.2991644	-2.0431023
H	-3.3121686	-1.2999228	1.4766753
H	-4.4444581	-1.1580494	0.1515444
H	-3.7899163	0.2944233	0.8769300

H	-0.9185304	-3.8746824	-0.2371180
H	-2.6488324	-3.7318281	-0.4339350
H	-1.9013720	-3.1708767	1.0439889
H	0.6947575	-3.1438407	-1.5989255
H	1.2609509	-1.7655596	-2.5353825
H	0.0133759	-2.8124725	-3.1810419
C	0.6900036	1.3495753	-0.2930027
C	2.0604339	1.0601345	-0.1553912
C	0.3342005	2.6613056	-0.5808347
C	3.0300159	2.0465549	-0.3468537
C	1.3001388	3.6452222	-0.7620261
H	-0.7065227	2.9413460	-0.6650452
C	2.6499418	3.3370424	-0.6599041
H	4.0806187	1.8085984	-0.2430102
H	0.9960534	4.6579721	-0.9906645
H	3.3972203	4.1012179	-0.8139150
N	1.2693561	-1.0604966	0.4559502
C	2.3677014	-0.2911640	0.2678584
C	1.3982131	-2.2914097	0.9572683
C	3.6345798	-0.8076793	0.5323333
C	2.6250973	-2.8493799	1.2504760
H	0.4804824	-2.8356237	1.1281069
C	3.7651138	-2.0933985	1.0137932
H	4.5052448	-0.1915182	0.3667845
H	2.6806470	-3.8497489	1.6503849
H	4.7445925	-2.5016350	1.2172107
O	-0.7401043	0.0882365	1.8865152
C	-1.2234479	1.0302888	2.4904599
O	-1.7909863	2.0178303	1.8043257
C	-1.2131379	1.1275271	3.9736755
H	-0.7389275	0.2572781	4.4085902
H	-2.2284067	1.2146952	4.3570100
H	-0.6718050	2.0201638	4.2834847
H	-2.1060414	2.7108524	2.3929263

Co-Complex 8a

SCF energy:	–2251.791580 hartree
Zero-point correction:	+0.384452 hartree
Enthalpy correction:	+0.405016 hartree
Free energy correction:	+0.328353 hartree
Quasiharmonic free energy correction:	+0.343810 hartree

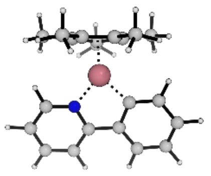

Cartesian Coordinates

Co	0.4938901	0.0102994	-0.1133254
C	2.2757277	-0.0608807	-1.0841752
C	2.1460877	1.1605942	-0.3344192
C	2.3220892	2.5178338	-0.9090725
C	2.4299326	-0.1317676	-2.5540484
C	2.2265943	-1.1689926	-0.1990584
C	1.9551983	-0.6513160	1.0946124
C	1.7373593	-1.4264973	2.3373523
C	2.4678656	-2.5835211	-0.5836034
C	1.9360965	0.7979929	1.0190552
C	1.7671922	1.6979769	2.1817482
H	1.0044088	1.3343950	2.8661752
H	1.4968860	2.7072162	1.8861011
H	2.7006468	1.7573736	2.7438314
H	1.6518947	2.7045460	-1.7458274
H	3.3399988	2.6294308	-1.2832759
H	2.1661193	3.2996843	-0.1725231
H	2.1042118	-1.0862553	-2.9581510
H	3.4820639	-0.0113967	-2.8214901
H	1.8867854	0.6636999	-3.0591923
H	2.2138533	-3.2791747	0.2117229
H	3.5253427	-2.7337405	-0.8027632
H	1.9203371	-2.8736057	-1.4788869

H	1.5247264	-2.4731017	2.1391488
H	0.9177389	-1.0197733	2.9265229
H	2.6269243	-1.3865722	2.9681035
C	-0.9293772	1.3033167	-0.0043677
C	-2.2114631	0.7162210	-0.0819581
C	-0.8658913	2.6920322	0.0717534
C	-3.3610426	1.4970306	-0.1112792
C	-2.0141561	3.4745557	0.0509551
H	0.0881644	3.1947258	0.1469957
C	-3.2614376	2.8778489	-0.0457134
H	-4.3380155	1.0375937	-0.1813905
H	-1.9337998	4.5508988	0.1110952
H	-4.1549970	3.4842379	-0.0647115
N	-1.0015173	-1.3034069	-0.0717889
C	-2.2357500	-0.7378512	-0.1062517
C	-0.8917018	-2.6359235	-0.0516319
C	-3.3719790	-1.5347760	-0.1396396
C	-1.9851233	-3.4774934	-0.0718358
H	0.1107327	-3.0338752	-0.0188040
C	-3.2487874	-2.9112955	-0.1226820
H	-4.3466352	-1.0725361	-0.1724802
H	-1.8423675	-4.5463132	-0.0501817
H	-4.1297971	-3.5363274	-0.1460947

Co-Complex 9a

SCF energy:	–3450.942427 hartree
Zero-point correction:	+0.622902 hartree
Enthalpy correction:	+0.658918 hartree
Free energy correction:	+0.544788 hartree
Quasiharmonic free energy correction:	+0.575808 hartree

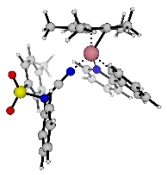

Cartesian Coordinates

Co	-1.6441595	-0.2195573	-0.6289768
C	-1.9144853	-0.8130715	-2.5654330
C	-3.1654654	-0.8513239	-1.8888180
C	-4.0866399	-2.0070618	-1.7765296
C	-1.2383116	-1.9298612	-3.2725859
C	-1.4627733	0.5601848	-2.5767525
C	-2.3800991	1.3358976	-1.8536739
C	-2.3633604	2.8112375	-1.6853555
C	-0.2315506	1.0339372	-3.2529440
C	-3.4135538	0.4505347	-1.3656168
C	-4.5960585	0.8531345	-0.5638786
H	-4.3647064	1.6627496	0.1261270
H	-4.9849220	0.0220533	0.0198713
H	-5.4044737	1.2012841	-1.2083667
H	-3.5852262	-2.9549786	-1.9465423
H	-4.8724645	-1.9126734	-2.5269553
H	-4.5681614	-2.0572744	-0.8031344
H	-0.1715791	-1.9567833	-3.0554046
H	-1.3400414	-1.8238343	-4.3534815
H	-1.6594357	-2.8946731	-3.0039985
H	0.0379055	2.0401837	-2.9419759
H	-0.3786055	1.0529788	-4.3336410
H	0.6191902	0.3815294	-3.0621579
H	-1.3524365	3.2165833	-1.6600871
H	-2.8776901	3.1269113	-0.7808920
H	-2.8772120	3.2890934	-2.5209266
C	-2.0021491	-1.7529537	0.5050703
C	-2.3995905	-1.4011388	1.8057138
C	-1.9000510	-3.0987936	0.1878652
C	-2.7742264	-2.3820106	2.7268380
C	-2.2534358	-4.0740813	1.1130475
H	-1.5446867	-3.4061778	-0.7883930
C	-2.7138829	-3.7162007	2.3741856
H	-3.0915398	-2.1031852	3.7234509

H	-2.1718427	-5.1194628	0.8475536
H	-3.0014251	-4.4777174	3.0840230
N	-1.7544250	0.7407984	1.1085051
C	-2.2604527	0.0037434	2.1281391
C	-1.4073022	2.0125946	1.3149020
C	-2.5101042	0.6017470	3.3619205
C	-1.6133848	2.6503988	2.5209535
H	-0.9398466	2.5194503	0.4805382
C	-2.2005742	1.9319859	3.5542617
H	-2.9302284	0.0125673	4.1628609
H	-1.3221904	3.6820169	2.6436862
H	-2.3954467	2.4034907	4.5067642
O	4.8873137	0.4641689	0.1210832
H	3.1146691	1.7701612	1.9528560
H	2.3610691	-2.9369330	-1.2016059
H	3.3744362	-5.1179686	-0.5997734
H	1.8728780	3.8155883	2.5551014
C	2.6620990	2.3832171	1.1856092
C	1.9658497	3.5310265	1.5157228
C	2.9866898	-3.0342902	-0.3242362
C	3.5585773	-4.2489363	0.0147927
S	3.6692812	0.5889177	-0.6033493
C	2.7803492	2.0284238	-0.1522330
O	3.5907066	0.4120622	-2.0173530
N	2.6309230	-0.6556756	0.1437886
C	3.2292353	-1.9295085	0.4781616
C	1.3939867	4.3381109	0.5312977
H	0.6001989	5.7355898	1.9603218
C	4.3773149	-4.3436698	1.1299651
H	1.1153468	6.4591742	0.4458799
C	0.6468983	5.5802743	0.8863428
H	4.8305645	-5.2901705	1.3853917
C	1.3606798	-0.5941952	-0.1705076
C	2.2232557	2.8123575	-1.1545468
C	1.5381829	3.9608160	-0.8047773
C	4.0410483	-2.0100832	1.5979634
N	0.2362493	-0.4766747	-0.4300759
C	4.6201436	-3.2267300	1.9159231
H	2.3476122	2.5235457	-2.1875391
H	-0.3731799	5.5521152	0.5021249
H	1.1194776	4.5866106	-1.5824621
H	4.2186764	-1.1306078	2.1987533
H	5.2590995	-3.3026484	2.7833502

Transition State TS3a

SCF energy:	–3450.916519 hartree
Zero-point correction:	+0.621397 hartree
Enthalpy correction:	+0.656763 hartree
Free energy correction:	+0.543801 hartree
Quasiharmonic free energy correction:	+0.574738 hartree
Imaginary Frequency	241.2 $i$cm^{-1}

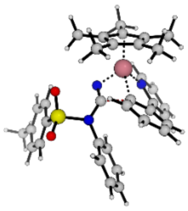

Cartesian Coordinates

Co	1.8570666	0.0894177	-0.6840433
C	2.4720556	1.9308437	-1.3292119
C	3.5126044	1.3131982	-0.5507317
C	4.1578836	1.8652949	0.6662646
C	1.8478895	3.2605860	-1.1150577
C	2.2067996	1.0943979	-2.4549172
C	3.0290774	-0.0550612	-2.3620138
C	3.1251065	-1.1266467	-3.3852444
C	1.1963986	1.3734259	-3.5003222
C	3.8417325	0.0867004	-1.1741916
C	4.8605943	-0.8821341	-0.7025347

H	4.6045061	-1.9058441	-0.9674907
H	4.9945896	-0.8364157	0.3758054
H	5.8279753	-0.6658761	-1.1573680
H	3.5735140	2.6671172	1.1081773
H	5.1397439	2.2718280	0.4230849
H	4.3034525	1.1044207	1.4312507
H	0.7611517	3.2146410	-1.1837687
H	2.1791956	3.9541350	-1.8891507
H	2.1191435	3.6957808	-0.1576970
H	0.9025682	0.4716152	-4.0301243
H	1.6025646	2.0719823	-4.2334034
H	0.3007920	1.8242878	-3.0774557
H	2.1437469	-1.4698303	-3.7097113
H	3.6933839	-1.9845649	-3.0357413
H	3.6360942	-0.7536277	-4.2736458
C	1.1956595	0.4573888	1.2100269
C	1.4451209	-0.6678893	2.0250728
C	1.0868762	1.7054183	1.8324477
C	1.5778805	-0.5276645	3.4052310
C	1.2560927	1.8413181	3.1967346
H	0.8248339	2.5728664	1.2426104
C	1.4941573	0.7219797	3.9883703
H	1.7763626	-1.3964606	4.0183860
H	1.1824574	2.8191619	3.6518775
H	1.6109678	0.8285058	5.0568032
N	1.8313370	-1.8073040	0.0127386
C	1.6016023	-1.9337796	1.3377469
C	1.9246099	-2.9052929	-0.7421109
C	1.4972467	-3.1900622	1.9283778
C	1.8443248	-4.1778244	-0.2145705
H	2.0590432	-2.7466174	-1.8014095
C	1.6325367	-4.3202238	1.1493069
H	1.2961077	-3.2658992	2.9864662
H	1.9388494	-5.0346951	-0.8631567
H	1.5588702	-5.3024338	1.5934218
O	-3.1099806	2.6178052	0.2701840
H	-4.7709175	0.7003697	0.4151396
H	-2.0274422	2.4226655	2.5756756
H	-2.9604968	1.5703506	4.7050214
H	-6.2334546	-1.1077090	-0.4179495
C	-4.5218818	0.1597010	-0.4871169
C	-5.3348367	-0.8505909	-0.9620387
C	-2.1453424	1.3625703	2.7424274
C	-2.6674237	0.8770102	3.9299333
S	-2.3509350	1.7680301	-0.5870392
C	-3.3793460	0.5002614	-1.2045951
O	-1.5078957	2.2806751	-1.6215961
N	-1.2619422	0.9510131	0.4984248
C	-1.7821230	0.4683012	1.7443402
C	-5.0290170	-1.5302701	-2.1418357
H	-5.6810494	-3.5666777	-2.1383155
C	-2.8086521	-0.4890440	4.1287666
H	-6.9608208	-2.4161532	-2.4420799
C	-5.9122812	-2.6256419	-2.6382276
H	-3.2132487	-0.8603204	5.0592478
C	-0.2138363	0.2826190	-0.0883648
C	-3.0452523	-0.1622024	-2.3793862
C	-3.8762776	-1.1673957	-2.8381037
C	-1.9324828	-0.9014444	1.9321620
N	0.0010985	-0.2599379	-1.1637165

C	-2.4372051	-1.3781536	3.1303903
H	-2.1554926	0.1211955	-2.9222267
H	-5.7905882	-2.7909416	-3.7048965
H	-3.6354104	-1.6747533	-3.7624933
H	-1.6630919	-1.5804205	1.1317846
H	-2.5582883	-2.4421630	3.2763692

Co-Complex 10a

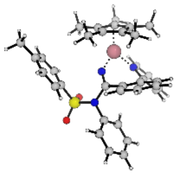

SCF energy:	–3450.946732 hartree
Zero-point correction:	+0.623874 hartree
Enthalpy correction:	+0.659520 hartree
Free energy correction:	+0.544742 hartree
Quasiharmonic free energy correction:	+0.578440 hartree

Cartesian Coordinates

Co	1.9717815	-0.6572241	-0.1488875
C	2.7779771	-0.4110570	-1.9900027
C	3.5482628	0.2381742	-0.9691424
C	3.8725650	1.6813833	-0.9043752
C	2.0898428	0.2414731	-3.1292365
C	2.6986256	-1.7845392	-1.6488285
C	3.4890488	-2.0124538	-0.4537076
C	3.6634775	-3.3303843	0.2054760
C	1.9187406	-2.8116842	-2.3788854
C	4.0191903	-0.7740641	-0.0489730
C	4.8872781	-0.5078753	1.1245835
H	4.8599911	-1.3155703	1.8516106
H	4.6129836	0.4131073	1.6352318
H	5.9244605	-0.3952361	0.8080284
H	3.1015844	2.2911403	-1.3703847
H	4.8091583	1.8843985	-1.4275107
H	3.9999920	2.0244003	0.1202253
H	1.1157674	-0.2070344	-3.3151946
H	2.6803541	0.1392115	-4.0400807
H	1.9423279	1.3045288	-2.9540635
H	1.6564034	-3.6524465	-1.7409086
H	2.5056738	-3.2114850	-3.2072778
H	1.0027437	-2.3984371	-2.7960254
H	2.7176620	-3.8613453	0.3069562
H	4.1061471	-3.2382916	1.1937418
H	4.3189470	-3.9728795	-0.3840097
C	0.1737389	2.0764445	-0.3700942
C	0.9328837	2.2924504	0.7938289
C	-0.0118892	3.1147225	-1.2775961
C	1.4683605	3.5651024	1.0063976
C	0.5611573	4.3565660	-1.0658652
H	-0.6170850	2.9313910	-2.1556241
C	1.3030831	4.5823358	0.0836391
H	2.0404988	3.7485120	1.9064662
H	0.4188423	5.1473989	-1.7877533
H	1.7481116	5.5493652	0.2661477
N	1.6282989	0.0689521	1.6598028
C	1.1210224	1.3044140	1.8819016
C	1.8306259	-0.7565312	2.6992175
C	0.8151852	1.6990277	3.1858555
C	1.5433358	-0.4154918	4.0021198
H	2.2355675	-1.7289496	2.4531862
C	1.0188274	0.8429361	4.2486102
H	0.3945736	2.6813429	3.3410999
H	1.7230750	-1.1234342	4.7959175
H	0.7656779	1.1498367	5.2531368
O	-3.6162852	-0.1953645	-2.4328984
H	-4.5803076	-0.9802289	-0.1804169
H	-2.0109730	0.8274956	1.5441440
H	-3.5695850	2.2640222	2.8334396

H	-5.0096280	-2.5944395	1.6374093
C	-3.8491168	-1.7369998	0.0677283
C	-4.0847282	-2.6489939	1.0792253
C	-2.6910210	1.5017482	1.0373545
C	-3.5685191	2.2994859	1.7531930
S	-2.3610101	-0.6314597	-1.9128373
C	-2.6617091	-1.8129589	-0.6526678
O	-1.3051143	-1.0817832	-2.7653072
N	-1.7555577	0.7372918	-1.0762229
C	-2.6907753	1.5468482	-0.3532170
C	-3.1602602	-3.6490186	1.3811266
H	-3.9977398	-4.2215346	3.2747035
C	-4.4562869	3.1266294	1.0813783
H	-4.0628964	-5.4665283	2.0509150
C	-3.4478673	-4.6566477	2.4437059
H	-5.1444106	3.7458092	1.6383440
C	-0.4157784	0.7411704	-0.6384809
C	-1.7231958	-2.7955616	-0.3704915
C	-1.9805526	-3.7050894	0.6394489
C	-3.5773944	2.3743593	-1.0293852
N	0.2927835	-0.2941405	-0.5366334
C	-4.4623861	3.1566037	-0.3061898
H	-0.8136541	-2.8402962	-0.9504606
H	-2.5384844	-5.1072637	2.8324219
H	-1.2580981	-4.4820095	0.8540107
H	-3.5711555	2.3898977	-2.1088707
H	-5.1554703	3.7987962	-0.8298224

Co-Complex 11a

SCF energy:	–3680.140137 hartree
Zero-point correction:	+0.688856 hartree
Enthalpy correction:	+0.728836 hartree
Free energy correction:	+0.606662 hartree
Quasiharmonic free energy correction:	+0.633143 hartree

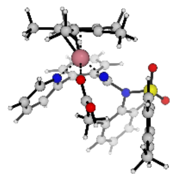

Cartesian Coordinates

Co	1.8291658	-0.7659497	-0.2095377
C	2.3291983	-0.4390705	-2.1765896
C	3.3113867	0.0850719	-1.2867412
C	3.8286464	1.4730165	-1.2615990
C	1.5699238	0.3176981	-3.1961601
C	2.1883767	-1.8251782	-1.8922804
C	3.1958907	-2.1844015	-0.9165729
C	3.4193116	-3.5519622	-0.3958847
C	1.2247243	-2.7520637	-2.5316896
C	3.8663115	-1.0169850	-0.5263533
C	4.9692716	-0.8988593	0.4590993
H	5.0177287	-1.7562375	1.1258956
H	4.8770129	-0.0013389	1.0687469
H	5.9320865	-0.8345302	-0.0493022
H	3.1038499	2.1917369	-1.6387513
H	4.7197646	1.5448960	-1.8882788
H	4.1197329	1.7821725	-0.2597798
H	0.5090332	0.0760766	-3.1824311
H	1.9490221	0.0679363	-4.1879450
H	1.6794799	1.3909124	-3.0677092
H	0.8307312	-3.4728885	-1.8161683
H	1.6986523	-3.3191987	-3.3342652
H	0.3872662	-2.2066824	-2.9606649
H	2.5041835	-4.1363123	-0.3939650
H	3.8179741	-3.5484548	0.6158260
H	4.1401100	-4.0727279	-1.0277073
C	0.4561274	2.2106894	-0.4599120

C	1.3495179	2.4517384	0.5928609
C	0.2952904	3.1812782	-1.4489180
C	2.0306735	3.6735745	0.6263785
C	1.0150111	4.3610722	-1.4276120
H	-0.4160586	2.9898653	-2.2411787
C	1.8819057	4.6139163	-0.3749801
H	2.7119358	3.8696235	1.4436725
H	0.8856252	5.0878448	-2.2163190
H	2.4375603	5.5390368	-0.3312599
N	1.9735519	0.2508009	1.5209079
C	1.6088137	1.5372433	1.7220377
C	2.3689347	-0.4829167	2.5760403
C	1.5696320	2.0565234	3.0188136
C	2.3770136	-0.0116394	3.8702938
H	2.6778729	-1.4937390	2.3490048
C	1.9425157	1.2843678	4.0987271
H	1.2354735	3.0743105	3.1544778
H	2.7088556	-0.6511555	4.6735208
H	1.9057694	1.6885956	5.1001603
O	-3.8033481	0.9217589	-2.1892660
H	-4.5490750	-0.0929826	0.2030361
H	-1.6267786	1.2499888	1.7631866
H	-2.7319032	2.8407925	3.3050958
H	-5.1585491	-2.0368638	1.5945572
C	-4.0414488	-1.0393894	0.0742522
C	-4.3842728	-2.1356263	0.8451502
C	-2.2526953	2.0455734	1.3776687
C	-2.8730977	2.9368606	2.2376235
S	-2.5985206	0.2228497	-1.8709815
C	-3.0443976	-1.1690066	-0.8876856
O	-1.6898699	-0.2114623	-2.8872714
N	-1.7350496	1.2600461	-0.8636207
C	-2.4281022	2.1625325	0.0013752
C	-3.7663409	-3.3734529	0.6568898
H	-4.2063569	-4.3177181	2.5401618
C	-3.6855634	3.9384634	1.7285172
H	-5.1627470	-4.9046958	1.2076471
C	-4.1660717	-4.5568297	1.4777748
H	-4.1754487	4.6310774	2.3971185
C	-0.3569857	0.9701909	-0.5511151
C	-2.4148595	-2.3867285	-1.0921316
C	-2.7874137	-3.4807130	-0.3297101
C	-3.2297139	3.1768476	-0.5105924
N	0.0489247	-0.2148939	-0.4480308
C	-3.8628310	4.0508873	0.3569494
H	-1.6495203	-2.4631430	-1.8491911
H	-3.4878708	-5.3955939	1.3389427
H	-2.3104816	-4.4367422	-0.5046311
H	-3.3559774	3.2666305	-1.5780347
H	-4.4900672	4.8340683	-0.0438018
O	1.0060772	-2.4359444	0.8351728
C	-0.0065280	-2.6648077	1.4718531
O	-0.3150807	-3.9483697	1.6582814
H	-1.1466698	-4.0273165	2.1361376
C	-0.9162242	-1.6473580	2.0633430
H	-1.6583225	-1.3752918	1.3124856
H	-0.3662704	-0.7462461	2.3121557
H	-1.4334450	-2.0229482	2.9436203

Co-Complex 12a

SCF energy:	–2573.299045 hartree
Zero-point correction:	+0.447767 hartree
Enthalpy correction:	+0.473895 hartree
Free energy correction:	+0.385721 hartree
Quasiharmonic free energy correction:	+0.399681 hartree

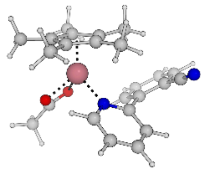

Cartesian Coordinates

Co	-0.8167516	0.1073547	0.4337291
O	0.0335241	-0.4607699	2.1692257
O	-1.1736613	1.3171711	1.9852327
N	0.6703100	1.3595179	-0.1508260
C	1.8345890	1.0539527	-0.7674024
C	-2.5119445	0.4194654	-0.6075798
C	-1.5203204	-0.0857543	-1.4882513
C	-1.0052018	0.5869228	-2.7018057
C	-3.2213932	1.7157265	-0.7029668
C	-2.7192974	-0.5599128	0.4352638
C	-1.8344409	-1.6388405	0.1925598
C	-1.6298503	-2.8328851	1.0399243
C	-3.6667212	-0.4215068	1.5637880
C	-1.0716903	-1.3490708	-0.9847366
C	-0.1594187	-2.3042153	-1.6572210
C	-0.4686205	0.5629218	2.7261756
C	-0.2260210	0.8779699	4.1541144
H	-1.0769665	1.3955281	4.5840418
H	0.6338771	1.5415865	4.2291172
H	-0.0023743	-0.0223824	4.7158010
H	0.6107202	-2.6894460	-0.9909550
H	0.3263571	-1.8637276	-2.5223812
H	-0.7303116	-3.1633063	-2.0110372
H	-1.0608315	1.6700010	-2.6274265
H	-1.6030141	0.2942361	-3.5654838
H	0.0237308	0.3143538	-2.9241790
H	-3.2886200	2.2047607	0.2673210
H	-4.2431945	1.5639564	-1.0517123
H	-2.7402167	2.3964537	-1.3996229
H	-3.3847929	-1.0462088	2.4072048
H	-4.6675269	-0.7242516	1.2550920
H	-3.7282239	0.6069395	1.9096628
H	-2.0370763	-2.7050388	2.0376079
H	-0.5718743	-3.0693366	1.1322368
H	-2.1177532	-3.6975399	0.5883386
C	0.2761495	2.6459746	-0.1248218
C	0.9838671	3.6673210	-0.7193879
C	2.1624050	3.3597420	-1.3752715
C	2.5888878	2.0483640	-1.3854672
C	2.3489549	-0.3276286	-0.7425503
H	2.7474049	4.1283072	-1.8583894
H	3.5213324	1.7796673	-1.8570605
H	-0.6457383	2.8410465	0.4011588
H	0.6104151	4.6781935	-0.6644135
C	2.4467829	-1.0186903	0.4594542
C	2.8455821	-0.9413804	-1.9095752
C	3.4259942	-2.2079985	-1.8470277
C	3.0219173	-2.2780101	0.5133781
C	3.5175144	-2.8713702	-0.6376454
H	2.0848056	-0.5546423	1.3624800
H	3.0978988	-2.7880650	1.4629185
H	3.9745665	-3.8489176	-0.5954663
H	3.7916774	-2.6640105	-2.7548954
C	2.6670097	-0.3257001	-3.1788719
N	2.4722843	0.1655526	-4.2085052

2-Phenylpyridine (1a)

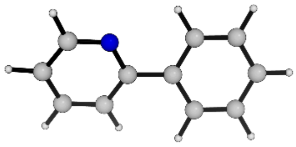

SCF energy:	–479.486942 hartree
Zero-point correction:	+0.170364 hartree
Enthalpy correction:	+0.178644 hartree
Free energy correction:	+0.134150 hartree
Quasiharmonic free energy correction:	+0.136454 hartree

Cartesian Coordinates

C	2.8400514	1.1693371	-0.1834346
C	1.4559796	1.1936247	-0.1875708
C	3.5154167	-0.0264168	0.0084624
C	2.7945522	-1.1985300	0.1878007
C	1.4117237	-1.1757663	0.1776874
C	0.7188547	0.0226497	-0.0031884
C	-0.7557603	0.0248685	-0.0004468
C	-1.4939671	1.1931891	0.2039686
N	-1.3606450	-1.1565136	-0.1892898
C	-2.6860982	-1.1967622	-0.1975515
C	-3.4941240	-0.0846498	-0.0198584
C	-2.8741094	1.1354351	0.1905379
H	0.9462809	2.1313410	-0.3606837
H	3.3929735	2.0855219	-0.3380024
H	4.5962865	-0.0452065	0.0139310
H	3.3146223	-2.1346535	0.3380954
H	0.8394794	-2.0823150	0.3104760
H	-3.4582954	2.0313916	0.3506308
H	-0.9926945	2.1313476	0.3914438
H	-3.1302355	-2.1739685	-0.3560016
H	-4.5701415	-0.1762142	-0.0392061

N-Cyano-4-methyl-*N*-phenylbenzenesulfonamide (2a)

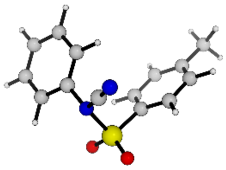

SCF energy:	–1199.107140 hartree
Zero-point correction:	+0.235115 hartree
Enthalpy correction:	+0.251110 hartree
Free energy correction:	+0.185759 hartree
Quasiharmonic free energy correction:	+0.201157 hartree

Cartesian Coordinates

N	1.3757688	-1.0369984	-0.4904390
C	1.8350160	0.3211601	-0.3614493
C	1.3577638	1.3005304	-1.2227714
C	1.7979430	2.6052960	-1.0797890
C	2.6931595	2.9320070	-0.0722217
C	3.1595744	1.9466547	0.7858473
C	2.7413130	0.6340705	0.6418842
H	0.6538071	1.0318826	-1.9993551
H	1.4350211	3.3676522	-1.7543643
H	3.0334540	3.9515098	0.0389494
H	3.8653455	2.1963077	1.5650024
H	3.1025836	-0.1441572	1.2961259
C	-1.1081089	-0.6634934	0.5364589
C	-1.2175019	0.5417978	1.2183555
C	-2.0675100	-1.0526894	-0.3918210
C	-3.1509504	-0.2261230	-0.6205925
C	-3.2927041	0.9851221	0.0568264
C	-2.3102767	1.3533714	0.9745131
H	-2.4043547	2.2917705	1.5046817
H	-0.4551924	0.8259706	1.9302976

H	-1.9566682	-1.9902872	-0.9180635
H	-3.9022406	-0.5237794	-1.3398934
C	-4.4852322	1.8522284	-0.1767965
H	-5.3258327	1.5272512	0.4370970
H	-4.8170861	1.8104228	-1.2114587
H	-4.2860754	2.8899351	0.0773546
S	0.3166443	-1.6542274	0.7607462
O	0.0515179	-2.9977721	0.3710220
O	0.9555329	-1.3010033	1.9855004
C	1.0849915	-1.4794031	-1.7178325
N	0.8387778	-1.8650972	-2.7832448

Acetic Acid

SCF energy: –229.177298 hartree
Zero-point correction: +0.062132 hartree
Enthalpy correction: +0.067050 hartree
Free energy correction: +0.034660 hartree
Quasiharmonic free energy correction: +0.034660 hartree

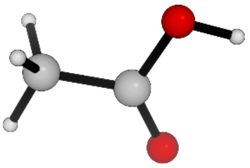

Cartesian Coordinates

C	-1.3836747	-0.1063986	0.0000171
C	0.0907411	0.1267888	0.0000204
H	-1.6710837	-0.6882789	-0.8723122
H	-1.9104458	0.8392135	-0.0003112
H	-1.6711260	-0.6876390	0.8727625
O	0.6426988	1.1940970	0.0003104
O	0.7693975	-1.0448439	-0.0003380
H	1.7049926	-0.8134990	0.0000709

2-(Pyridin-2-yl)benzonitrile (3a)

SCF energy: –571.762209 hartree
Zero-point correction: +0.168805 hartree
Enthalpy correction: +0.178854 hartree
Free energy correction: +0.130345 hartree
Quasiharmonic free energy correction: +0.132738 hartree

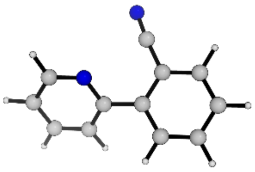

Cartesian Coordinates

C	-2.6223688	-1.7647158	-0.1926483
C	-1.2434267	-1.6577056	-0.1573359
C	-3.4079642	-0.6260514	-0.1039608
C	-2.8052912	0.6105822	0.0249247
C	-1.4134643	0.7275277	0.0572105
C	-0.6089371	-0.4235579	-0.0397377
C	0.8622746	-0.3453779	-0.0343322
C	1.6388883	-1.3470899	0.5490722
N	1.4071963	0.7247950	-0.6198283
C	2.7292838	0.8261418	-0.6423207
C	3.5799392	-0.1228037	-0.0956309
C	3.0158379	-1.2305594	0.5142166
H	-0.6364312	-2.5470762	-0.2551741
H	-3.0829192	-2.7366355	-0.2992426
H	-4.4852875	-0.6997662	-0.1327653
H	-3.4029006	1.5059739	0.1129491
C	-0.8816265	2.0341151	0.2572783
H	3.6395252	-1.9898301	0.9650472
H	1.1689127	-2.1866830	1.0415942
H	3.1297378	1.7108938	-1.1245030
H	4.6510140	0.0081750	-0.1431588

N	-0.5651027	3.1256482	0.4709859

4-Methyl-*N*-phenylbenzenesulfonamide

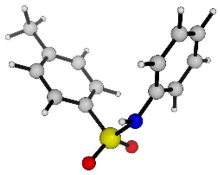

SCF energy:	–1106.854665 hartree
Zero-point correction:	+0.237709 hartree
Enthalpy correction:	+0.251659 hartree
Free energy correction:	+0.191697 hartree
Quasiharmonic free energy correction:	+0.202340 hartree

Cartesian Coordinates

N	1.4294035	1.4713544	-0.2313195
C	1.9928537	0.1906673	-0.0553806
C	2.4255445	-0.5136725	-1.1765856
C	3.0159750	-1.7574954	-1.0346350
C	3.1616759	-2.3235416	0.2225992
C	2.7259980	-1.6225856	1.3369641
C	2.1540559	-0.3676076	1.2106768
H	2.2989034	-0.0792979	-2.1601567
H	3.3533521	-2.2892158	-1.9130773
H	3.6145768	-3.2980560	0.3325718
H	2.8435291	-2.0487685	2.3233991
H	1.8400255	0.1891195	2.0800586
C	-1.1743601	0.7843987	0.1562712
C	-1.8864349	0.8884267	-1.0332850
C	-1.3879554	-0.2852937	1.0129158
C	-2.3195027	-1.2523111	0.6720581
C	-3.0458365	-1.1694806	-0.5117547
C	-2.8143672	-0.0820930	-1.3560939
H	-3.3771620	0.0048441	-2.2765206
H	-1.7223364	1.7389153	-1.6806585
H	-0.8391242	-0.3416363	1.9418847
H	-2.4905623	-2.0852857	1.3409368
C	-4.0557516	-2.2086065	-0.8744175
H	-4.1063163	-2.9979795	-0.1296522
H	-3.8240487	-2.6697179	-1.8335485
H	-5.0515665	-1.7770870	-0.9697853
S	0.0466477	1.9942098	0.5436427
O	0.2894508	1.9349378	1.9508996
O	-0.2816459	3.2135503	-0.1289263
H	1.4231986	1.7945082	-1.1868313

7 Optimization with B3LYP-D3BJ/def2-SVP for 1a (R = H)

Co-Complex 4

SCF energy:	–2001.181324 hartree
Zero-point correction:	+0.272562 hartree
Enthalpy correction:	+0.290387 hartree
Free energy correction:	+0.221188 hartree
Quasiharmonic free energy correction:	+0.234055 hartree

Cartesian Coordinates

Co	-0.3171349	0.0002732	-0.2638762
O	-1.9088456	-1.0778642	-0.3970817
O	-1.9095156	1.0776636	-0.3950074
C	1.0990361	1.1772280	0.6628234
C	0.7449629	-0.0013350	1.4319245
C	0.1555360	-0.0031928	2.7981257
C	0.8763224	2.5843287	1.1056565
C	1.5865148	0.7329114	-0.5911908
C	1.5870359	-0.7303059	-0.5928753
C	1.9976849	-1.5708856	-1.7540206
C	1.9966536	1.5764870	-1.7503461
C	1.0998730	-1.1778578	0.6601091
C	0.8780653	-2.5861039	1.0997641
C	-2.5954311	-0.0002218	-0.4942365
C	-4.0604460	-0.0004238	-0.7383462
H	-4.5170237	0.9066869	-0.3205835
H	-4.5163216	-0.9092495	-0.3235756
H	-4.2284194	0.0013704	-1.8291063
H	0.9509469	-3.2922679	0.2633515
H	-0.1111412	-2.7092435	1.5640996
H	1.6362599	-2.8662150	1.8503522
H	-0.4554425	0.8909757	2.9814187
H	0.9718178	-0.0042434	3.5433380
H	-0.4554512	-0.8978641	2.9789430
H	0.9472710	3.2922625	0.2705707
H	1.6352964	2.8637379	1.8557255
H	-0.1123415	2.7054745	1.5716455
H	1.6158309	1.1741287	-2.7010331
H	3.0980090	1.5904555	-1.8259427
H	1.6514441	2.6131024	-1.6490693
H	1.6157559	-1.1670550	-2.7036459
H	1.6539454	-2.6081748	-1.6547293
H	3.0990120	-1.5831522	-1.8302916

Co-Complex 5a

SCF energy:	–2480.535482 hartree
Zero-point correction:	+0.447497 hartree
Enthalpy correction:	+0.472782 hartree
Free energy correction:	+0.386060 hartree
Quasiharmonic free energy correction:	+0.399160 hartree

Cartesian Coordinates

Co	-0.9177851	-0.0062165	0.0225290
O	-0.8540738	-0.5341200	1.9550726
O	-2.1208342	1.0348193	1.2108645
N	0.4688285	1.4395462	0.1586421
C	1.8181903	1.3409995	0.2890445
C	-1.0791576	0.0056759	-2.0497876

C	-0.2262106	-1.0974655	-1.6448574
C	1.1422834	-1.3596381	-2.1753000
C	-0.6734055	1.1074106	-2.9733265
C	-2.3643458	-0.1945660	-1.4677159
C	-2.2960658	-1.3677704	-0.6333004
C	-3.4077341	-1.9260633	0.1934735
C	-3.5539046	0.6975716	-1.5840564
C	-0.9854927	-1.9439242	-0.7889375
C	-0.5098097	-3.1711366	-0.0879323
C	-1.7052551	0.3721913	2.2163571
C	-2.1556283	0.6704188	3.6082721
H	-3.1546254	1.1257456	3.6008223
H	-1.4501062	1.3891506	4.0573605
H	-2.1436476	-0.2432553	4.2176086
H	-0.7245031	-3.1107679	0.9897902
H	0.5683970	-3.3230229	-0.2154128
H	-1.0363990	-4.0555359	-0.4835559
H	1.6956049	-0.4308454	-2.3584138
H	1.0559868	-1.8879056	-3.1409590
H	1.7391647	-1.9833690	-1.5001877
H	-1.2911568	2.0050514	-2.8369573
H	-0.7821988	0.7830582	-4.0220823
H	0.3773498	1.3910249	-2.8232496
H	-3.9511048	0.9531036	-0.5905496
H	-4.3525267	0.1814005	-2.1424054
H	-3.3199237	1.6293069	-2.1149810
H	-4.0895551	-1.1348633	0.5341872
H	-3.0212325	-2.4526641	1.0770173
H	-4.0004148	-2.6485218	-0.3928790
C	-0.0901300	2.6644594	0.0280353
C	0.6420322	3.8425108	0.0126661
C	2.0285272	3.7617115	0.1544853
C	2.6078108	2.5069071	0.2959628
C	2.5038415	0.0294050	0.4041878
H	2.6466205	4.6623452	0.1653391
H	3.6836829	2.4062758	0.4377732
H	-1.1759716	2.6868769	-0.0548376
H	0.1276399	4.7981529	-0.0984365
C	3.6783292	-0.1837535	-0.3438910
C	2.0565281	-0.9837368	1.2664397
C	2.7577902	-2.1886347	1.3582635
C	4.3692611	-1.3915630	-0.2574904
C	3.9082384	-2.4016314	0.5936665
H	1.1747167	-0.8263308	1.8829884
H	4.0367636	0.5908303	-1.0256629
H	5.2689461	-1.5460549	-0.8572502
H	4.4520779	-3.3461637	0.6688076
H	2.4063777	-2.9627499	2.0446396

Transition State TS1a

SCF energy:	–2480.522267 hartree
Zero-point correction:	+0.446304 hartree
Enthalpy correction:	+0.471145 hartree
Free energy correction:	+0.385462 hartree
Quasiharmonic free energy correction:	+0.398240 hartree
Imaginary Frequency	92.6 $i$cm^{-1}

Cartesian Coordinates

Co	-0.6477628	0.0752849	-0.0586464
O	-1.5087297	-1.2317639	2.1162486
N	0.6763138	1.4654674	0.3205794
C	1.9894999	1.1649778	0.4643830
C	-0.5189968	0.3256830	-2.1303943
C	-0.1593592	-1.0099308	-1.8171020
C	1.0930059	-1.7074157	-2.2317326
C	0.3299356	1.3207594	-2.8482119

C	-1.8448046	0.5683560	-1.6010580
C	-2.3311094	-0.6747149	-1.0389135
C	-3.6753645	-0.8874378	-0.4293538
C	-2.6314805	1.8322689	-1.6873483
C	-1.2906729	-1.6279734	-1.1396440
C	-1.3402486	-3.0212120	-0.6080717
C	-1.9804759	-0.0937325	2.2155992
C	-2.9040967	0.3128247	3.3369791
H	-2.7152444	1.3501691	3.6462261
H	-2.7902602	-0.3737331	4.1852892
H	-3.9447845	0.2566586	2.9770836
H	-1.6788371	-3.0186517	0.4381419
H	-0.3589043	-3.5083558	-0.6559454
H	-2.0511668	-3.6221680	-1.1995450
H	1.9129910	-1.0016082	-2.4122343
H	0.9123609	-2.2580789	-3.1708731
H	1.4290574	-2.4317526	-1.4803606
H	0.1032749	2.3509651	-2.5431406
H	0.1445687	1.2483557	-3.9333845
H	1.4000088	1.1384132	-2.6811460
H	-3.1340394	2.0436209	-0.7321566
H	-3.4116853	1.7389175	-2.4618634
H	-2.0030882	2.6926416	-1.9524047
H	-4.0392141	0.0260832	0.0594087
H	-3.6525602	-1.6939114	0.3144065
H	-4.4021529	-1.1629735	-1.2123434
C	0.2627413	2.7440454	0.3599067
C	1.1526517	3.8047649	0.4770797
C	2.5186342	3.5214705	0.5745219
C	2.9364230	2.1934569	0.5812113
C	2.3486183	-0.2638736	0.5483599
H	3.2482761	4.3286113	0.6710747
H	3.9883150	1.9396661	0.7142682
H	-0.8148329	2.8993088	0.3052500
H	0.7782125	4.8292274	0.4996768
C	3.5757701	-0.7528938	0.0702788
C	1.4412849	-1.1602430	1.1430063
C	1.7474357	-2.5183279	1.2494072
C	3.8814026	-2.1104065	0.1811825
C	2.9706855	-2.9963869	0.7711607
H	0.5211689	-0.8154892	1.6309909
H	4.2863279	-0.0770217	-0.4104434
H	4.8358162	-2.4819044	-0.1984928
H	3.2217919	-4.0553768	0.8648079
H	1.0329194	-3.1881908	1.7320595
O	-1.7260304	0.8237703	1.3286517

Co-Complex 6a

SCF energy:	–2480.523843 hartree
Zero-point correction:	+0.446614 hartree
Enthalpy correction:	+0.472059 hartree
Free energy correction:	+0.385435 hartree
Quasiharmonic free energy correction:	+0.397128 hartree

Cartesian Coordinates

Co	-0.5814373	0.0809192	-0.0512958
O	-1.5117952	-1.4336525	2.2355637
N	0.7279296	1.4920476	0.2643302
C	2.0328274	1.1413208	0.3523118
C	-0.6455766	0.4151303	-2.1193056
C	-0.2995339	-0.9388408	-1.8916849
C	0.8993515	-1.6468324	-2.4287576
C	0.1600608	1.4166635	-2.8780679
C	-1.9047891	0.6715309	-1.4491368
C	-2.3763802	-0.5834747	-0.9009101
C	-3.6704250	-0.7888782	-0.1901375

C	-2.6669642	1.9538994	-1.4221525
C	-1.3798614	-1.5584256	-1.1313835
C	-1.4415542	-2.9782170	-0.6748280
C	-1.8220893	-0.2514178	2.3682844
C	-2.6489602	0.2496438	3.5313601
H	-2.2706170	1.2148729	3.8962962
H	-2.6531382	-0.4955383	4.3365244
H	-3.6849950	0.4090236	3.1897483
H	-1.6682179	-3.0200526	0.4005919
H	-0.4975599	-3.5056929	-0.8575155
H	-2.2407122	-3.5113467	-1.2162260
H	1.7211965	-0.9528731	-2.6455013
H	0.6342139	-2.1560162	-3.3712393
H	1.2714813	-2.4087032	-1.7331808
H	-0.0038673	2.4389193	-2.5118674
H	-0.1306096	1.3962703	-3.9420673
H	1.2354891	1.1998973	-2.8236890
H	-3.0728998	2.1463571	-0.4181296
H	-3.5193436	1.9026330	-2.1207947
H	-2.0472311	2.8093141	-1.7216609
H	-3.9774299	0.1169130	0.3488860
H	-3.6052678	-1.6157764	0.5276557
H	-4.4599310	-1.0278732	-0.9231056
C	0.3579096	2.7777136	0.3608452
C	1.2906517	3.8025766	0.4792014
C	2.6483713	3.4694380	0.5130601
C	3.0217711	2.1279910	0.4618885
C	2.3119919	-0.3072521	0.3908059
H	3.4084516	4.2484183	0.6055318
H	4.0688120	1.8350992	0.5442082
H	-0.7148306	2.9715975	0.3489872
H	0.9554345	4.8383347	0.5503892
C	3.5019694	-0.8630816	-0.1036551
C	1.3430211	-1.1625104	0.9551683
C	1.5571040	-2.5408592	1.0270593
C	3.7144378	-2.2417656	-0.0297289
C	2.7481413	-3.0832839	0.5375444
H	0.4666464	-0.7778081	1.5018064
H	4.2570965	-0.2233187	-0.5652211
H	4.6427493	-2.6650632	-0.4199456
H	2.9318979	-4.1578715	0.6051646
H	0.7976402	-3.1679606	1.4969202
O	-1.4847155	0.6765740	1.5086441

Transition State TS2a

SCF energy:	–2480.507280 hartree
Zero-point correction:	+0.442221 hartree
Enthalpy correction:	+0.466955 hartree
Free energy correction:	+0.382144 hartree
Quasiharmonic free energy correction:	+0.394438 hartree
Imaginary Frequency	940.5 $i$cm^{-1}

Cartesian Coordinates

Co	-0.5826982	-0.0548688	-0.0052540
O	0.1334568	0.4138014	2.8939363
N	0.6777424	1.4128196	-0.2132854
C	1.9872076	1.0623761	-0.1598261
C	-0.9821570	-0.0786140	-2.0857519
C	-0.5021175	-1.3527282	-1.6532458
C	0.6755525	-2.0829040	-2.2119334
C	-0.3416437	0.7782275	-3.1273825
C	-2.1571363	0.2376970	-1.3278872
C	-2.4599443	-0.9039472	-0.4933881
C	-3.6360945	-0.9994576	0.4183709
C	-3.0033629	1.4658676	-1.4181954
C	-1.4507492	-1.8784494	-0.6887496

C	-1.4405262	-3.2541881	-0.1057430
C	-0.9558443	0.9651981	2.5966895
C	-1.7161546	1.7145203	3.6581237
H	-2.4352561	2.4109168	3.2094896
H	-1.0152741	2.2417447	4.3192688
H	-2.2652052	0.9824965	4.2731205
H	-1.7365740	-3.2577487	0.9528657
H	-0.4564183	-3.7287828	-0.1925736
H	-2.1654794	-3.8839674	-0.6492992
H	1.4403868	-1.3899448	-2.5867461
H	0.3614527	-2.7205267	-3.0559032
H	1.1478978	-2.7283641	-1.4602642
H	-0.6093088	1.8370290	-3.0161886
H	-0.6794707	0.4547622	-4.1265140
H	0.7538790	0.6954197	-3.1086656
H	-3.2337882	1.8557174	-0.4153260
H	-3.9624849	1.2382960	-1.9127529
H	-2.5166022	2.2603795	-1.9997407
H	-3.7510836	-0.0767312	1.0042348
H	-3.5479164	-1.8444542	1.1139472
H	-4.5571121	-1.1418091	-0.1718327
C	0.3162193	2.6821780	-0.4459813
C	1.2563203	3.6768162	-0.6946603
C	2.6119972	3.3298869	-0.6942585
C	2.9813016	2.0155119	-0.4176261
C	2.2120131	-0.3303424	0.2471050
H	3.3762263	4.0855197	-0.8892989
H	4.0321779	1.7293430	-0.3688649
H	-0.7542038	2.8910084	-0.4281934
H	0.9299571	4.7009493	-0.8810990
C	3.4162121	-1.0164636	0.0535868
C	1.1053076	-0.9575461	0.8836733
C	1.2719270	-2.2708337	1.3517291
C	3.5407263	-2.3327787	0.5065417
C	2.4751383	-2.9569835	1.1670683
H	0.5074013	-0.2499233	1.8088982
H	4.2551264	-0.5403946	-0.4589635
H	4.4787582	-2.8713439	0.3539696
H	2.5913273	-3.9744319	1.5480206
H	0.4627316	-2.7492244	1.9069763
O	-1.4676678	0.8978598	1.4312490

Co-Complex 7a

SCF energy:	–2480.517729 hartree
Zero-point correction:	+0.447267 hartree
Enthalpy correction:	+0.472756 hartree
Free energy correction:	+0.385618 hartree
Quasiharmonic free energy correction:	+0.400035 hartree

Cartesian Coordinates

Co	-0.4290891	-0.2293153	-0.0646727
C	-2.4646266	-0.8079026	-0.4290428
C	-2.0763038	0.2902097	-1.2710705
C	-2.8363611	1.5707852	-1.4201640
C	-3.5807632	-0.7834061	0.5643582
C	-1.6187527	-1.9195529	-0.6906664
C	-0.6771845	-1.5075793	-1.7013486
C	0.3863002	-2.3557339	-2.3213551
C	-1.7734651	-3.2871057	-0.0988191
C	-0.9731978	-0.1562851	-2.0783752
C	-0.3109751	0.5950308	-3.1877390
H	0.7612582	0.3636041	-3.2475791
H	-0.4068186	1.6803101	-3.0611873
H	-0.7694532	0.3191468	-4.1528236
H	-3.0382115	2.0425089	-0.4489345
H	-3.8072959	1.3789057	-1.9072835

H	-2.2962836	2.2925795	-2.0454901
H	-3.3583658	-1.4064343	1.4427257
H	-4.5013133	-1.1827684	0.1050788
H	-3.7994436	0.2380244	0.9031561
H	-0.9078084	-3.9285546	-0.3118342
H	-2.6574309	-3.7923764	-0.5233397
H	-1.9172073	-3.2533669	0.9925431
H	0.7011756	-3.1743446	-1.6605375
H	1.2763812	-1.7636732	-2.5762036
H	0.0108545	-2.8072441	-3.2553316
C	0.6836301	1.3427798	-0.2744752
C	2.0614003	1.0649422	-0.1148173
C	0.3075452	2.6602668	-0.5509101
C	3.0305518	2.0690197	-0.2828109
C	1.2717886	3.6646885	-0.7097445
H	-0.7445793	2.9265408	-0.6482627
C	2.6344949	3.3689793	-0.5903637
H	4.0929343	1.8435697	-0.1648630
H	0.9558123	4.6872468	-0.9337952
H	3.3828803	4.1526341	-0.7257509
N	1.2605434	-1.0776155	0.4691427
C	2.3645737	-0.3025636	0.3066961
C	1.3729674	-2.3177804	0.9652658
C	3.6364761	-0.8210263	0.5950578
C	2.6028167	-2.8835776	1.2828141
H	0.4454528	-2.8698061	1.1106743
C	3.7575853	-2.1202477	1.0771357
H	4.5192604	-0.1984023	0.4505219
H	2.6496692	-3.8980754	1.6809016
H	4.7431623	-2.5331431	1.3039486
O	-0.7291629	0.1403239	1.8663755
C	-1.2035626	1.0953975	2.4634746
O	-1.8627754	2.0296278	1.7890788
C	-1.0503087	1.2645170	3.9461761
H	-0.6412280	0.3471431	4.3844080
H	-2.0130501	1.5113638	4.4206224
H	-0.3498149	2.0949802	4.1398126
H	-2.1579526	2.7520060	2.3656431

Co-Complex 8a

SCF energy:	–2251.383557 hartree
Zero-point correction:	+0.383256 hartree
Enthalpy correction:	+0.403687 hartree
Free energy correction:	+0.328737 hartree
Quasiharmonic free energy correction:	+0.339195 hartree

Cartesian Coordinates

Co	0.4396784	0.0821484	-0.3454614
C	2.4233551	-0.2310271	-1.0784243
C	2.2328875	1.1103790	-0.5255315
C	2.4603856	2.3723073	-1.2934911
C	2.8052565	-0.4998765	-2.4966119
C	2.1772354	-1.1733995	-0.0637547
C	1.7350599	-0.4396043	1.1053362
C	1.3376531	-1.0355602	2.4138826
C	2.3708277	-2.6542935	-0.1277760
C	1.8895525	0.9770542	0.8422971
C	1.7662835	2.0467762	1.8766207
H	0.8675127	1.9220091	2.4951151
H	1.7430067	3.0510089	1.4375507
H	2.6433848	1.9966872	2.5441484
H	1.9273994	2.3631797	-2.2564903
H	3.5338812	2.4942209	-1.5168486
H	2.1338456	3.2581898	-0.7341599
H	2.6743524	-1.5554841	-2.7694705
H	3.8652789	-0.2393011	-2.6600195

H	2.2167539	0.1142466	-3.1958527
H	1.6317247	-3.1983118	0.4763729
H	3.3653345	-2.9089326	0.2769404
H	2.3360475	-3.0384776	-1.1570317
H	0.8506179	-2.0121757	2.2852030
H	0.6515617	-0.3768306	2.9633929
H	2.2302823	-1.1902209	3.0457637
C	-0.9759651	1.2889288	0.1059763
C	-2.2270729	0.6393136	0.2588947
C	-0.9358478	2.6802460	0.2451282
C	-3.3807588	1.3581623	0.5958639
C	-2.0936897	3.4058370	0.5599214
H	-0.0047249	3.2287201	0.1012416
C	-3.3093914	2.7449409	0.7544137
H	-4.3410805	0.8527365	0.7192490
H	-2.0427339	4.4931222	0.6576113
H	-4.2066179	3.3103203	1.0149740
N	-0.9962851	-1.2453271	-0.4702834
C	-2.2129217	-0.7937075	-0.0504460
C	-0.8633310	-2.5098009	-0.9027444
C	-3.3093816	-1.6618673	-0.0064265
C	-1.9199175	-3.4144957	-0.9018059
H	0.1211140	-2.8013929	-1.2640723
C	-3.1618429	-2.9821821	-0.4275403
H	-4.2742645	-1.2971950	0.3453078
H	-1.7663977	-4.4316952	-1.2641726
H	-4.0130181	-3.6662657	-0.3982398

Co-Complex 9a

SCF energy:	–3450.204953 hartree
Zero-point correction:	+0.619639 hartree
Enthalpy correction:	+0.656340 hartree
Free energy correction:	+0.538861 hartree
Quasiharmonic free energy correction:	+0.576492 hartree

Cartesian Coordinates

Co	-1.8370392	-0.4693050	-0.9241376
C	-2.5223005	-0.7700514	-2.8703691
C	-3.6429563	-0.7977840	-1.9851010
C	-4.6689745	-1.8745231	-1.8534943
C	-2.0856783	-1.8387542	-3.8214659
C	-1.9428993	0.5636252	-2.8169170
C	-2.6488773	1.3102544	-1.8527289
C	-2.4671375	2.7581745	-1.5253333
C	-0.8049336	1.0234537	-3.6719948
C	-3.6586789	0.4429382	-1.2693568
C	-4.6468789	0.8319619	-0.2163046
H	-4.2209894	1.5622882	0.4864612
H	-4.9720266	-0.0423111	0.3647743
H	-5.5453044	1.2879481	-0.6663957
H	-4.3228713	-2.8323316	-2.2588084
H	-5.5690836	-1.5782016	-2.4191572
H	-4.9660443	-2.0360942	-0.8088828
H	-0.9896929	-1.9261719	-3.8473444
H	-2.4235790	-1.6087956	-4.8463193
H	-2.4979111	-2.8203480	-3.5519293
H	-0.4931034	2.0453389	-3.4167434
H	-1.1066106	1.0276615	-4.7324925
H	0.0741644	0.3678557	-3.5862647
H	-1.4405047	3.1028107	-1.7152619
H	-2.7205154	2.9807402	-0.4799684
H	-3.1367387	3.3688342	-2.1550903
C	-2.1631385	-2.0705429	0.1031169
C	-2.1647029	-1.8447852	1.4985568
C	-2.3473561	-3.3751435	-0.3585586
C	-2.4403639	-2.8889606	2.4000445

C	-2.6067424	-4.4181249	0.5386204
H	-2.2886858	-3.5988941	-1.4251368
C	-2.6764303	-4.1731753	1.9163560
H	-2.4553873	-2.7066805	3.4772620
H	-2.7553568	-5.4327910	0.1597574
H	-2.8924406	-4.9888188	2.6094684
N	-1.4506011	0.3070278	0.8427980
C	-1.7334142	-0.5074770	1.8949045
C	-0.8739885	1.4998700	1.0478729
C	-1.5011966	-0.0628659	3.2077404
C	-0.5967606	1.9853350	2.3205492
H	-0.6101836	2.0723672	0.1610095
C	-0.9394468	1.1911162	3.4220700
H	-1.7430310	-0.7143270	4.0473839
H	-0.1220111	2.9593386	2.4380585
H	-0.7518901	1.5425248	4.4393382
O	4.7301211	-0.0472905	-1.4032763
H	4.3575499	1.3314087	0.8940170
H	4.5877338	-2.4521950	-0.2503184
H	5.4828101	-3.1248996	1.9880788
H	3.6720064	3.3047752	2.2479666
C	3.5661295	1.9963653	0.5460862
C	3.1785273	3.1065721	1.2933071
C	4.0786695	-2.1603125	0.6680573
C	4.5716386	-2.5265911	1.9220083
S	3.3368750	0.3060701	-1.6010626
C	2.9199729	1.7538414	-0.6713893
O	2.6694416	0.3365913	-2.8948474
N	2.4276100	-0.9775605	-0.6932620
C	2.9145076	-1.3903588	0.5988657
C	2.1773414	3.9827158	0.8362466
H	1.8919263	5.0194108	2.7177077
C	3.8981729	-2.1421823	3.0864411
H	2.4712967	6.0424278	1.3922708
C	1.8003692	5.2005752	1.6361785
H	4.2855875	-2.4414965	4.0628560
C	1.1306292	-0.9744842	-0.9242663
C	1.9219249	2.6026870	-1.1588277
C	1.5633289	3.7155848	-0.4001063
C	2.2372741	-0.9877156	1.7576979
N	-0.0138594	-0.9261686	-1.1122301
C	2.7290513	-1.3784391	3.0032706
H	1.4526957	2.3919235	-2.1201152
H	0.7735077	5.5299141	1.4183605
H	0.7971176	4.3977794	-0.7775992
H	1.3358442	-0.3766521	1.6820057
H	2.2010231	-1.0781627	3.9110188

Transition State TS3a

SCF energy:	–3450.167677 hartree
Zero-point correction:	+0.618415 hartree
Enthalpy correction:	+0.654195 hartree
Free energy correction:	+0.540895 hartree
Quasiharmonic free energy correction:	+0.569412 hartree
Imaginary Frequency	335.6 $i\text{cm}^{-1}$

Cartesian Coordinates

Co	0.6639404	1.5922068	-0.3210927
C	0.3046914	3.2447036	-1.5384838
C	0.3384807	3.6964059	-0.1701076
C	-0.7448376	4.4144182	0.5686292
C	-0.8042991	3.4083616	-2.5291905
C	1.6040200	2.7131600	-1.8654793
C	2.3970081	2.7379901	-0.6926115
C	3.8416326	2.3637661	-0.5928903
C	1.9908838	2.1875940	-3.2072664

C	1.5966686	3.3307615	0.3743356
C	2.0558815	3.5823038	1.7747798
H	2.7562120	2.8078331	2.1189324
H	1.2094527	3.6086931	2.4756254
H	2.5763932	4.5525796	1.8464849
H	-1.7008235	4.3794331	0.0325794
H	-0.4700448	5.4750862	0.6944933
H	-0.9059732	3.9918582	1.5709829
H	-1.0149785	2.4610030	-3.0486598
H	-0.5287480	4.1479225	-3.2993890
H	-1.7314131	3.7578077	-2.0562962
H	2.9444930	1.6441890	-3.1740022
H	2.0991795	3.0192470	-3.9234974
H	1.2244384	1.5013140	-3.5946442
H	4.0884023	1.4971828	-1.2239834
H	4.1406456	2.1411091	0.4401767
H	4.4709906	3.2031127	-0.9353243
C	-1.1463341	1.1151781	0.4661900
C	-0.9236850	0.7262662	1.8161630
C	-2.3898016	1.6640085	0.1127354
C	-1.9186196	0.9223326	2.7772034
C	-3.3838220	1.8528117	1.0752710
H	-2.5855352	1.9281482	-0.9270971
C	-3.1448801	1.4854156	2.4025452
H	-1.7442994	0.6591072	3.8227946
H	-4.3502150	2.2727129	0.7891908
H	-3.9219197	1.6328696	3.1557493
N	1.3145816	0.4002198	1.1112610
C	0.4049847	0.1680678	2.0940715
C	2.5348150	-0.1441866	1.1882115
C	0.7527178	-0.5764903	3.2281034
C	2.9433349	-0.8933829	2.2880314
H	3.1957681	0.0212937	0.3394852
C	2.0396193	-1.0985538	3.3333969
H	0.0129502	-0.7539666	4.0091207
H	3.9487738	-1.3138994	2.3121264
H	2.3283876	-1.6790166	4.2123035
O	-1.7267866	-3.8260093	-0.8552327
H	0.1142403	-3.9721269	0.9789516
H	-3.3612953	-0.5786027	-2.2727309
H	-5.8099981	-0.5037516	-1.7469594
H	2.4389888	-4.3450455	1.8072362
C	0.9749179	-3.6240824	0.4057203
C	2.2769040	-3.8350527	0.8544025
C	-3.7317696	-0.8604281	-1.2846525
C	-5.0943869	-0.8245535	-0.9865739
S	-0.8698929	-2.8010126	-1.4353986
C	0.7853744	-2.9812892	-0.8204354
O	-0.8409002	-2.5205741	-2.8647817
N	-1.4198484	-1.3058553	-0.5993725
C	-2.8228135	-1.2729258	-0.3032024
C	3.3843932	-3.4213759	0.0936915
H	4.8288176	-3.8623189	1.6515715
C	-5.5416405	-1.2131051	0.2809789
H	5.1914981	-4.5960821	0.0786208
C	4.7883582	-3.6915130	0.5655823
H	-6.6091316	-1.1892877	0.5122158
C	-0.6743385	-0.1931641	-0.8612583
C	1.8611062	-2.5544051	-1.6031804
C	3.1548822	-2.7756504	-1.1332233
C	-3.2655628	-1.6895982	0.9529606
N	0.2862896	0.1202065	-1.5472233
C	-4.6291607	-1.6522792	1.2461936
H	1.6738955	-2.0683880	-2.5602113
H	5.4682006	-2.8630053	0.3128053
H	4.0059582	-2.4554632	-1.7402681
H	-2.5366748	-2.0361551	1.6860900
H	-4.9836023	-1.9737836	2.2281058

Co-Complex 10a

SCF energy:	–3450.209558 hartree
Zero-point correction:	+0.621272 hartree
Enthalpy correction:	+0.657166 hartree
Free energy correction:	+0.541573 hartree
Quasiharmonic free energy correction:	+0.576662 hartree

Cartesian Coordinates

Co	1.9605439	-0.6405207	-0.0280584
C	2.7243402	-0.3970531	-1.9426873
C	3.5442987	0.2401212	-0.9417967
C	3.9046470	1.6869457	-0.8953544
C	2.0069723	0.2738849	-3.0674966
C	2.6429672	-1.7761786	-1.6069815
C	3.4967847	-2.0214665	-0.4480640
C	3.7003119	-3.3580426	0.1904940
C	1.8248636	-2.7996717	-2.3255210
C	4.0563056	-0.7875359	-0.0504285
C	4.9880016	-0.5389891	1.0923256
H	4.9592807	-1.3512712	1.8313569
H	4.7505391	0.4009322	1.6108640
H	6.0262310	-0.4598139	0.7287442
H	3.1136548	2.3171820	-1.3217689
H	4.8255771	1.8612693	-1.4790708
H	4.0948564	2.0274587	0.1310505
H	0.9984983	-0.1422970	-3.2033149
H	2.5659985	0.1324291	-4.0077589
H	1.9118214	1.3539707	-2.8942165
H	1.5676641	-3.6442207	-1.6705348
H	2.3930457	-3.2095376	-3.1783125
H	0.8936097	-2.3632117	-2.7126789
H	2.7542232	-3.9120836	0.2867799
H	4.1538935	-3.2738362	1.1876671
H	4.3729937	-3.9775979	-0.4269676
C	0.2108800	2.0976700	-0.3451713
C	1.0140242	2.3631903	0.7890804
C	0.0087534	3.0956504	-1.3071068
C	1.5871551	3.6409956	0.9186995
C	0.6129601	4.3462441	-1.1776538
H	-0.6325394	2.8736640	-2.1618677
C	1.4045603	4.6193892	-0.0588840
H	2.2032613	3.8636236	1.7927398
H	0.4581429	5.1096232	-1.9433435
H	1.8777128	5.5968479	0.0569055
N	1.7189507	0.1633275	1.7338106
C	1.2176626	1.4118410	1.9201356
C	1.9467212	-0.6342279	2.7954728
C	0.9288504	1.8558671	3.2222638
C	1.6855352	-0.2454446	4.1030101
H	2.3510004	-1.6238522	2.5737193
C	1.1578184	1.0292115	4.3186812
H	0.5041417	2.8511864	3.3543138
H	1.8858753	-0.9324355	4.9264431
H	0.9227411	1.3733892	5.3284215
O	-3.5618746	-0.1584312	-2.4988907
H	-4.6536411	-1.0521351	-0.3374813
H	-2.0469779	0.8048693	1.6500083
H	-3.6760024	2.2071942	2.9260716
H	-5.1822318	-2.7367102	1.4218804
C	-3.9245416	-1.8137211	-0.0582344
C	-4.2121514	-2.7643183	0.9189391
C	-2.7374878	1.4702037	1.1264549
C	-3.6533663	2.2505742	1.8344964
S	-2.3098068	-0.6183595	-1.9107141
C	-2.6799332	-1.8594188	-0.6959208
O	-1.1871748	-1.0444066	-2.7461138
N	-1.7407586	0.7455890	-0.9828810

C	-2.7054368	1.5309211	-0.2739306
C	-3.2849208	-3.7654514	1.2575992
H	-4.1776800	-4.3742717	3.1343400
C	-4.5506036	3.0721445	1.1452871
H	-4.2835229	-5.5839293	1.8434393
C	-3.6308618	-4.8108227	2.2844761
H	-5.2731769	3.6770805	1.6977817
C	-0.4039424	0.7508344	-0.5382432
C	-1.7345305	-2.8377538	-0.3825539
C	-2.0480273	-3.7855636	0.5909548
C	-3.5998143	2.3548746	-0.9675912
N	0.2761241	-0.3009997	-0.3759537
C	-4.5252338	3.1167649	-0.2531727
H	-0.7773397	-2.8495614	-0.9006765
H	-2.7336240	-5.3167071	2.6698958
H	-1.3214626	-4.5649725	0.8354986
H	-3.5708068	2.3758722	-2.0565558
H	-5.2276233	3.7560254	-0.7927488

Co-Complex 11a

SCF energy:	–3679.326360 hartree
Zero-point correction:	+0.685313 hartree
Enthalpy correction:	+0.725858 hartree
Free energy correction:	+0.601832 hartree
Quasiharmonic free energy correction:	+0.630621 hartree

Cartesian Coordinates

Co	-1.7479979	-0.8206766	-0.0857713
C	-2.2671763	-1.3130588	1.8316999
C	-3.4340427	-1.5441793	0.9939394
C	-4.7541590	-0.8638125	1.1630768
C	-2.2600597	-0.5043708	3.0851280
C	-1.2222545	-2.1989519	1.3924646
C	-1.6942985	-2.8799549	0.2382124
C	-0.9539610	-3.8743701	-0.5927986
C	0.1240807	-2.3324240	2.0206012
C	-3.0859451	-2.5012902	0.0238255
C	-3.9436752	-3.0500067	-1.0661334
H	-3.3964006	-3.0781806	-2.0176085
H	-4.8586328	-2.4590012	-1.2081507
H	-4.2473905	-4.0813596	-0.8177497
H	-4.6323280	0.1860140	1.4655559
H	-5.3454592	-1.3651176	1.9478739
H	-5.3468233	-0.8850049	0.2383589
H	-1.2540148	-0.1567095	3.3484374
H	-2.6295129	-1.1261573	3.9197025
H	-2.9168756	0.3712552	3.0135180
H	0.8907995	-2.5779531	1.2784649
H	0.1081469	-3.1377469	2.7742286
H	0.4245240	-1.4061714	2.5268815
H	0.0993930	-3.9341798	-0.2959103
H	-0.9915965	-3.6026103	-1.6567522
H	-1.4025362	-4.8754316	-0.4779102
C	0.2057593	1.3779206	1.4038872
C	-1.0390612	2.0339400	1.4798259
C	1.0731345	1.4111258	2.5058134
C	-1.3691785	2.7204956	2.6635477
C	0.7160500	2.0628985	3.6850892
H	2.0449792	0.9227842	2.4184292
C	-0.5114812	2.7272698	3.7616311
H	-2.3294752	3.2363485	2.7304346
H	1.3994139	2.0665161	4.5372038
H	-0.8005227	3.2518219	4.6747399
N	-2.4419232	1.0179408	-0.3024901
C	-2.0043378	2.1212672	0.3530671
C	-3.4042345	1.1438779	-1.2397824

C	-2.5115088	3.3887237	0.0135974
C	-3.9627683	2.3653724	-1.5940456
H	-3.7000124	0.2227231	-1.7392849
C	-3.4929010	3.5185799	-0.9634340
H	-2.1101252	4.2657007	0.5213403
H	-4.7386001	2.4035558	-2.3602120
H	-3.8840434	4.5033576	-1.2289739
O	2.2404772	-0.2436448	-2.5661524
H	3.9893324	-0.4171538	1.3212560
H	0.3597509	2.7858936	-1.5213661
H	0.6671540	5.2579589	-1.6768818
H	4.3499902	-2.5080413	2.6168075
C	3.6553422	-1.3774230	0.9247527
C	3.8635399	-2.5531744	1.6390905
C	1.2295142	3.2645865	-1.0677126
C	1.4052162	4.6469857	-1.1517088
S	2.8998217	0.0517952	-1.2730361
C	3.0247902	-1.4441612	-0.3228769
O	4.2028957	0.7020559	-1.3276448
N	1.9454203	1.0643291	-0.2613312
C	2.1725860	2.4804466	-0.3907882
C	3.4784198	-3.8016817	1.1141468
H	4.8180475	-5.3298437	1.8304909
C	2.5319835	5.2432349	-0.5778631
H	3.4956551	-4.9512914	2.9477946
C	3.7475127	-5.0675087	1.8821713
H	2.6758947	6.3236614	-0.6502160
C	0.6376190	0.6505993	0.1706233
C	2.6285808	-2.6623822	-0.8707683
C	2.8633782	-3.8334480	-0.1466653
C	3.2989412	3.0745852	0.1869274
N	-0.0098587	-0.2554281	-0.4148027
C	3.4789325	4.4544996	0.0843892
H	2.1372043	-2.7001111	-1.8412884
H	3.1774294	-5.9169365	1.4797363
H	2.5629521	-4.7933207	-0.5737907
H	4.0307090	2.4510635	0.7000145
H	4.3625127	4.9181857	0.5286318
O	-1.9255131	-1.1205900	-2.3010076
C	-0.8838815	-0.7863669	-2.8703068
O	0.0859790	-1.6570470	-3.0221434
C	-0.6761414	0.5814824	-3.4594322
H	0.3146654	0.9622904	-3.1762470
H	-1.4658905	1.2672553	-3.1350917
H	-0.6956368	0.5016669	-4.5590245
H	0.9553388	-1.1790857	-3.0873456

Co-Complex 12a

SCF energy:	–2572.778405 hartree
Zero-point correction:	+0.445779 hartree
Enthalpy correction:	+0.473053 hartree
Free energy correction:	+0.381409 hartree
Quasiharmonic free energy correction:	+0.398402 hartree

Cartesian Coordinates

Co	-0.8108629	0.1663527	0.4013087
O	0.1110331	-0.3098450	2.1160456
O	-1.1376288	1.4315051	1.9029085
N	0.6444002	1.3578202	-0.2513987
C	1.8781461	1.0230905	-0.6993884
C	-2.6049958	0.3851717	-0.5902490
C	-1.6322973	-0.1032504	-1.5116841
C	-1.2038578	0.5626764	-2.7767913
C	-3.3739208	1.6619924	-0.6770616
C	-2.7324304	-0.5831496	0.4844505
C	-1.8303568	-1.6509257	0.2138755

C	-1.5742984	-2.8489992	1.0638844
C	-3.6549965	-0.4543946	1.6508513
C	-1.1240703	-1.3525527	-1.0033414
C	-0.1970108	-2.2929863	-1.6993869
C	-0.3854782	0.7298956	2.6531251
C	-0.0720913	1.1279137	4.0586314
H	-0.8903348	1.7265105	4.4800253
H	0.8404014	1.7471472	4.0528037
H	0.1221394	0.2392633	4.6739892
H	0.5677165	-2.6957468	-1.0220379
H	0.3046689	-1.8170942	-2.5486073
H	-0.7733863	-3.1480855	-2.0923827
H	-1.3251586	1.6529760	-2.7264240
H	-1.8283928	0.1995573	-3.6105932
H	-0.1591915	0.3463285	-3.0339433
H	-3.4060507	2.1734605	0.2965621
H	-4.4161225	1.4557687	-0.9743258
H	-2.9478400	2.3486695	-1.4202752
H	-3.3380160	-1.0869126	2.4910385
H	-4.6731355	-0.7670328	1.3635336
H	-3.7082098	0.5843071	2.0041426
H	-1.9348437	-2.7096614	2.0907652
H	-0.5008461	-3.0809190	1.1049786
H	-2.0905547	-3.7253379	0.6362172
C	0.2561837	2.6509707	-0.3046460
C	1.0575749	3.6639430	-0.8135858
C	2.3307406	3.3347899	-1.2798179
C	2.7376549	2.0065556	-1.2140863
C	2.3680478	-0.3831213	-0.6606578
H	2.9981552	4.0992722	-1.6833756
H	3.7299085	1.7113022	-1.5554510
H	-0.7352891	2.8684251	0.0903202
H	0.6847120	4.6891140	-0.8341634
C	2.5111936	-1.0808375	0.5435299
C	2.8067389	-1.0027107	-1.8584071
C	3.3794538	-2.2847622	-1.8321290
C	3.0754251	-2.3596878	0.5628499
C	3.5154534	-2.9602843	-0.6200981
H	2.1789896	-0.6217339	1.4721767
H	3.1836127	-2.8835522	1.5152800
H	3.9650268	-3.9552245	-0.6000563
H	3.7009831	-2.7454142	-2.7677523
C	2.5797652	-0.3578552	-3.1198014
N	2.3282532	0.1596780	-4.1284850

2-Phenylpyridine (1a)

SCF energy:	–479.298253 hartree
Zero-point correction:	+0.169948 hartree
Enthalpy correction:	+0.178267 hartree
Free energy correction:	+0.132939 hartree
Quasiharmonic free energy correction:	+0.138274 hartree

Cartesian Coordinates

C	2.8750358	1.1830879	-0.0818339
C	1.4797113	1.2204854	-0.0826925
C	3.5467680	-0.0394225	0.0070922
C	2.8090827	-1.2247594	0.0909654
C	1.4153684	-1.1877671	0.0880323
C	0.7265210	0.0361353	0.0054818
C	-0.7630233	0.0360339	0.0103114
C	-1.5177376	1.2224671	0.0910877
N	-1.3672336	-1.1627199	-0.0593549
C	-2.6957853	-1.2269836	-0.0604716
C	-3.5231258	-0.1017261	0.0073054
C	-2.9091879	1.1494652	0.0872909
H	0.9819822	2.1880284	-0.1649326

H	3.4405852	2.1155563	-0.1545127
H	4.6393707	-0.0678558	0.0085374
H	3.3247830	-2.1862166	0.1591275
H	0.8256457	-2.1028236	0.1485365
H	-3.5073108	2.0622750	0.1500621
H	-1.0267723	2.1926576	0.1656101
H	-3.1344941	-2.2306757	-0.1190027
H	-4.6100334	-0.2075318	0.0011601

N-Cyano-4-methyl-*N*-phenylbenzenesulfonamide (2a)

SCF energy:	–1198.771452 hartree
Zero-point correction:	+0.233701 hartree
Enthalpy correction:	+0.249895 hartree
Free energy correction:	+0.183165 hartree
Quasiharmonic free energy correction:	+0.202490 hartree

Cartesian Coordinates

N	-1.2861143	-1.3326956	-0.5105038
C	-1.9184536	-0.0436133	-0.4407827
C	-2.8950126	0.1793415	0.5358862
C	-3.4838472	1.4409972	0.6353349
C	-3.1142475	2.4652430	-0.2427673
C	-2.1443123	2.2302472	-1.2223029
C	-1.5347165	0.9782773	-1.3180645
H	-3.1698054	-0.6311891	1.2100081
H	-4.2449038	1.6209147	1.3982124
H	-3.5864052	3.4475849	-0.1659380
H	-1.8541972	3.0266274	-1.9117245
H	-0.7694292	0.7832149	-2.0723853
C	1.2114002	-0.6637421	0.4365977
C	2.2089766	-1.0890303	-0.4469709
C	1.2100559	0.6228528	0.9816294
C	2.2367461	1.4980922	0.6290248
C	3.2554400	1.1061494	-0.2549508
C	3.2235621	-0.1962436	-0.7844701
H	4.0087045	-0.5160989	-1.4744872
H	2.1809742	-2.0997194	-0.8566390
H	0.4185808	0.9226769	1.6695514
H	2.2468627	2.5073295	1.0484307
C	4.3760876	2.0474213	-0.6108463
H	4.1018525	3.0951907	-0.4195271
H	4.6610759	1.9499225	-1.6697154
H	5.2752768	1.8245296	-0.0104416
S	-0.1426342	-1.7529311	0.7921467
O	-0.8021233	-1.3275732	2.0196190
O	0.2366808	-3.1310859	0.5235630
C	-0.9748643	-1.8474106	-1.7094915
N	-0.6902200	-2.2894997	-2.7476749

Acetic Acid

SCF energy:	–229.104129 hartree
Zero-point correction:	+0.061671 hartree
Enthalpy correction:	+0.066643 hartree
Free energy correction:	+0.034005 hartree
Quasiharmonic free energy correction:	+0.034629 hartree

Cartesian Coordinates

C	-1.3937391	-0.1072916	0.0000014
C	0.0956072	0.1223669	0.0000538
H	-1.6806446	-0.6951749	-0.8857812
H	-1.9155498	0.8567814	-0.0003226
H	-1.6807133	-0.6945219	0.8862049
O	0.6469215	1.1937455	0.0003206
O	0.7775139	-1.0446777	-0.0001879

H	1.7221040	-0.8117878	-0.0000691

2-(Pyridin-2-yl)benzonitrile (3a)

SCF energy:	–571.542217 hartree
Zero-point correction:	+0.168463 hartree
Enthalpy correction:	+0.178560 hartree
Free energy correction:	+0.129770 hartree
Quasiharmonic free energy correction:	+0.132746 hartree

Cartesian Coordinates

C	-2.6508850	-1.7746851	-0.2069952
C	-1.2595540	-1.6749111	-0.1772826
C	-3.4364185	-0.6240495	-0.1007915
C	-2.8218986	0.6188558	0.0372557
C	-1.4192267	0.7272428	0.0648775
C	-0.6159045	-0.4351880	-0.0460978
C	0.8677406	-0.3544805	-0.0388800
C	1.6538571	-1.3522941	0.5657460
N	1.4139842	0.7125205	-0.6361390
C	2.7392784	0.8324332	-0.6591221
C	3.6038587	-0.1113369	-0.0944500
C	3.0420763	-1.2253972	0.5321537
H	-0.6536516	-2.5764199	-0.2882228
H	-3.1221284	-2.7536261	-0.3229036
H	-4.5262253	-0.6931381	-0.1250039
H	-3.4214226	1.5259345	0.1330638
C	-0.8681555	2.0410166	0.2639828
H	3.6768147	-1.9824061	0.9996948
H	1.1841877	-2.1964436	1.0732437
H	3.1379958	1.7260027	-1.1531890
H	4.6857431	0.0304618	-0.1406623
N	-0.5431762	3.1359083	0.4723616

4-Methyl-*N*-phenylbenzenesulfonamide

SCF energy:	–1106.553696 hartree
Zero-point correction:	+0.235745 hartree
Enthalpy correction:	+0.250029 hartree
Free energy correction:	+0.187701 hartree
Quasiharmonic free energy correction:	+0.205476 hartree

Cartesian Coordinates

N	1.4654417	1.5116850	-0.2321240
C	2.0030378	0.2177595	-0.0236004
C	2.1212160	-0.6769469	-1.0978144
C	2.6690603	-1.9454334	-0.8977920
C	3.0757608	-2.3425506	0.3789505
C	2.9459260	-1.4534100	1.4512417
C	2.4259181	-0.1732349	1.2569512
H	1.7777464	-0.3742809	-2.0909050
H	2.7653186	-2.6306337	-1.7437771
H	3.4954664	-3.3387108	0.5373473
H	3.2692454	-1.7529779	2.4514232
H	2.3340269	0.5326865	2.0819072
C	-1.1715294	0.7912710	0.1486526
C	-1.9833955	1.0087432	-0.9692012
C	-1.2630350	-0.3869525	0.8918968
C	-2.1772092	-1.3650056	0.4960163
C	-3.0024598	-1.1794114	-0.6232527
C	-2.8933431	0.0232834	-1.3445658
H	-3.5380584	0.1900858	-2.2121168
H	-1.9035482	1.9487998	-1.5182016
H	-0.6316835	-0.5265925	1.7699344
H	-2.2533504	-2.2906232	1.0724532
C	-3.9942843	-2.2319741	-1.0465256

H	-3.9378751	-3.1237702	-0.4058168
H	-3.8167604	-2.5480133	-2.0878331
H	-5.0257954	-1.8445394	-0.9992989
S	0.0563782	2.0142054	0.5815307
O	0.3192264	1.9012580	2.0140109
O	-0.2946185	3.2790265	-0.0611181
H	1.4013971	1.8014569	-1.2061227

8 Optimization with B3LYP-D3BJ/def2-SVP for 1b (R = Me)

Co-Complex 5b

SCF energy:	–2519.847194 hartree
Zero-point correction:	+0.474494 hartree
Enthalpy correction:	+0.501485 hartree
Free energy correction:	+0.409686 hartree
Quasiharmonic free energy correction:	+0.427501 hartree

Cartesian Coordinates

Co	-0.9362350	0.0140484	0.0242060
O	-0.8705147	-0.5152016	1.9567032
O	-2.1523891	1.0412936	1.2119688
N	0.4342321	1.4738171	0.1609821
C	1.7859071	1.3877152	0.2818652
C	-1.0851964	0.0226804	-2.0495172
C	-0.2308295	-1.0767763	-1.6381199
C	1.1411418	-1.3372317	-2.1605849
C	-0.6775598	1.1253320	-2.9711143
C	-2.3727236	-0.1803496	-1.4737837
C	-2.3058175	-1.3533132	-0.6383773
C	-3.4202705	-1.9144713	0.1827831
C	-3.5641019	0.7085216	-1.5972194
C	-0.9927441	-1.9247926	-0.7856978
C	-0.5159228	-3.1490120	-0.0801085
C	-1.7305085	0.3827071	2.2174337
C	-2.1840663	0.6770313	3.6092496
H	-3.1911834	1.1141992	3.6018435
H	-1.4913403	1.4104852	4.0544307
H	-2.1545260	-0.2341542	4.2216293
H	-0.7393770	-3.0889313	0.9958242
H	0.5642972	-3.2938513	-0.1985506
H	-1.0341595	-4.0366988	-0.4794073
H	1.6883821	-0.4073959	-2.3556337
H	1.0615266	-1.8808981	-3.1182668
H	1.7404591	-1.9455342	-1.4736307
H	-1.2985686	2.0212281	-2.8382293
H	-0.7795863	0.8003382	-4.0203435
H	0.3714447	1.4120658	-2.8149423
H	-3.9706649	0.9592759	-0.6063048
H	-4.3563248	0.1921736	-2.1644836
H	-3.3282836	1.6429031	-2.1226870
H	-4.1079922	-1.1255581	0.5167858
H	-3.0368880	-2.4364078	1.0704993
H	-4.0056004	-2.6417587	-0.4049737
C	-0.1377935	2.6929894	0.0309500
C	0.5823414	3.8778176	0.0035483
C	1.9712165	3.8105397	0.1324828
C	2.5635596	2.5626101	0.2758000
C	2.4834477	0.0852987	0.3994720
H	2.5807337	4.7170819	0.1332986
H	3.6413326	2.4735571	0.4103155
H	-1.2245225	2.7042934	-0.0422489
H	0.0578110	4.8279872	-0.1068862
C	3.6654818	-0.1212960	-0.3387416
C	2.0442282	-0.9399413	1.2500913
C	2.7552094	-2.1377857	1.3380657
C	4.3614597	-1.3234240	-0.2536606
C	3.9204672	-2.3595017	0.5881964
H	1.1571094	-0.7996952	1.8630551
H	4.0251136	0.6544640	-1.0187124

H	5.2636435	-1.4646763	-0.8544943
C	4.6967428	-3.6436293	0.7060142
H	2.3993644	-2.9170696	2.0174450
H	5.1093640	-3.9566798	-0.2652494
H	4.0746223	-4.4602775	1.1003692
H	5.5503310	-3.5172118	1.3943109

Transition State TS1b

SCF energy:	–2519.834373 hartree
Zero-point correction:	+0.473384 hartree
Enthalpy correction:	+0.499897 hartree
Free energy correction:	+0.409644 hartree
Quasiharmonic free energy correction:	+0.425590 hartree
Imaginary Frequency	94.8 $i\text{cm}^{-1}$

Cartesian Coordinates

Co	-0.7979666	-0.1569058	-0.0504572
O	-0.7888657	-1.8238732	2.0315658
N	-0.2909386	1.6769210	0.4040955
C	1.0105260	2.0464553	0.4854940
C	-0.9230153	0.2421109	-2.0989775
C	0.0576053	-0.7627745	-1.8971566
C	1.4651622	-0.7349787	-2.3918444
C	-0.7051330	1.5642173	-2.7554103
C	-2.1675144	-0.2248901	-1.5254066
C	-1.9557745	-1.5766619	-1.0502565
C	-2.9901535	-2.4533484	-0.4294122
C	-3.4726866	0.4958433	-1.4944783
C	-0.5905662	-1.8919484	-1.2457297
C	0.0741458	-3.1580370	-0.8195241
C	-1.7528854	-1.0749612	2.2281897
C	-2.6914584	-1.2445645	3.3969603
H	-3.0744086	-0.2747368	3.7425855
H	-2.1837952	-1.7770720	4.2113078
H	-3.5534916	-1.8503167	3.0713834
H	-0.1560077	-3.3728258	0.2340886
H	1.1633647	-3.1013330	-0.9338159
H	-0.2930178	-3.9986945	-1.4317066
H	1.8268178	0.2902607	-2.5363905
H	1.5224389	-1.2514958	-3.3653790
H	2.1523438	-1.2412885	-1.7035699
H	-1.3918763	2.3313006	-2.3736759
H	-0.8824425	1.4705617	-3.8402840
H	0.3238394	1.9223754	-2.6166233
H	-3.9523969	0.3875435	-0.5107060
H	-4.1568982	0.0709030	-2.2485017
H	-3.3593282	1.5654191	-1.7148061
H	-3.7213540	-1.8635378	0.1390888
H	-2.5355759	-3.1883482	0.2469224
H	-3.5372235	-3.0019798	-1.2148306
C	-1.2683543	2.5885577	0.5520517
C	-0.9979895	3.9406809	0.7202033
C	0.3380242	4.3540736	0.7530544
C	1.3455203	3.3997912	0.6483198
C	2.0197535	0.9735282	0.4521385
H	0.5903147	5.4084806	0.8864169
H	2.3948592	3.6842444	0.7294861
H	-2.2874666	2.2016438	0.5419998
H	-1.8191582	4.6502869	0.8310504
C	3.3006759	1.1636191	-0.0932994
C	1.7040485	-0.2834877	0.9974339
C	2.6344850	-1.3223868	0.9872979
C	4.2259430	0.1220619	-0.0975680
C	3.9159876	-1.1398584	0.4452477
H	0.7637182	-0.4636222	1.5328522
H	3.5696187	2.1221004	-0.5428034

H	5.2131027	0.2867118	-0.5371708
C	4.9409269	-2.2411901	0.4703564
H	2.3581356	-2.2803089	1.4341543
O	-2.0325861	-0.1057626	1.4075753
H	5.5388286	-2.2568372	-0.4536867
H	4.4738266	-3.2278036	0.6022882
H	5.6443060	-2.0942423	1.3081124

Co-Complex 6b

SCF energy:	–2519.836183 hartree
Zero-point correction:	+0.473610 hartree
Enthalpy correction:	+0.500762 hartree
Free energy correction:	+0.409252 hartree
Quasiharmonic free energy correction:	+0.425043 hartree

Cartesian Coordinates

Co	-0.5846666	0.1102732	-0.0512552
O	-1.5078215	-1.4175713	2.2381315
N	0.7080240	1.5362210	0.2599038
C	2.0165315	1.1950712	0.3388803
C	-0.6537891	0.4438810	-2.1198820
C	-0.2948542	-0.9067221	-1.8924610
C	0.9099961	-1.6049796	-2.4287681
C	0.1422731	1.4530503	-2.8788240
C	-1.9147202	0.6883208	-1.4485610
C	-2.3739892	-0.5712989	-0.9003075
C	-3.6655951	-0.7898562	-0.1888814
C	-2.6891431	1.9634171	-1.4213561
C	-1.3682197	-1.5365019	-1.1309606
C	-1.4156769	-2.9573235	-0.6755245
C	-1.8243874	-0.2369623	2.3699379
C	-2.6544611	0.2595657	3.5330329
H	-2.2862507	1.2301488	3.8939991
H	-2.6494328	-0.4828991	4.3407321
H	-3.6927750	0.4059991	3.1924701
H	-1.6433909	-3.0019594	0.3995073
H	-0.4650837	-3.4735182	-0.8564353
H	-2.2078000	-3.4990630	-1.2187392
H	1.7166678	-0.9016156	-2.6701951
H	0.6429492	-2.1400842	-3.3561962
H	1.3037009	-2.3437179	-1.7200833
H	-0.0361072	2.4742094	-2.5164534
H	-0.1432398	1.4255504	-3.9440391
H	1.2201742	1.2504453	-2.8186040
H	-3.0998432	2.1500401	-0.4181995
H	-3.5386660	1.9061798	-2.1230074
H	-2.0762361	2.8252143	-1.7166121
H	-3.9855660	0.1149776	0.3441331
H	-3.5892047	-1.6110610	0.5343590
H	-4.4511966	-1.0447681	-0.9206724
C	0.3282252	2.8191554	0.3549463
C	1.2535768	3.8518605	0.4595057
C	2.6145660	3.5293101	0.4802947
C	2.9985014	2.1911016	0.4325031
C	2.3043661	-0.2493753	0.3839701
H	3.3691441	4.3149842	0.5606159
H	4.0486717	1.9069875	0.5055017
H	-0.7462414	3.0039306	0.3520774
H	0.9110433	4.8852725	0.5295905
C	3.4930012	-0.8093934	-0.1097053
C	1.3447869	-1.1114826	0.9513392
C	1.5641152	-2.4874971	1.0236667
C	3.7053569	-2.1853179	-0.0341267
C	2.7529458	-3.0518490	0.5379069
H	0.4669996	-0.7333392	1.5005125
H	4.2476537	-0.1736851	-0.5779472

H	4.6338051	-2.6028272	-0.4324853
C	3.0223402	-4.5278743	0.6494528
H	0.8028692	-3.1116008	1.4958255
O	-1.4934057	0.6933282	1.5105157
H	3.5062997	-4.9185049	-0.2588759
H	2.0980272	-5.0962437	0.8260858
H	3.7039419	-4.7333928	1.4926910

Transition State TS2b

SCF energy:	–2519.819356 hartree
Zero-point correction:	+0.469295 hartree
Enthalpy correction:	+0.495696 hartree
Free energy correction:	+0.406266 hartree
Quasiharmonic free energy correction:	+0.421915 hartree
Imaginary Frequency	959.8 *i*cm^{-1}

Cartesian Coordinates

Co	-0.5461642	-0.4209310	-0.0085650
O	0.0774893	0.2024282	2.8836614
N	-0.7974178	1.5078435	-0.0376071
C	0.3445390	2.2358424	-0.1257886
C	-1.1231399	-0.5792464	-2.0410542
C	0.1849299	-1.1195070	-1.8489447
C	1.4208130	-0.7095594	-2.5815542
C	-1.4675985	0.5459932	-2.9601299
C	-2.0289304	-1.2833674	-1.1808679
C	-1.2835360	-2.3371325	-0.5297233
C	-1.8703093	-3.3302269	0.4154188
C	-3.4999318	-1.0700045	-1.0262373
C	0.0709763	-2.2393694	-0.9324783
C	1.1590358	-3.2018528	-0.5816545
C	-1.1027012	-0.2071620	2.7470789
C	-1.9794860	-0.3476402	3.9628006
H	-3.0405571	-0.3428568	3.6840150
H	-1.7558553	0.4513497	4.6821827
H	-1.7455570	-1.3092111	4.4489206
H	1.1269575	-3.4949002	0.4773666
H	2.1525639	-2.7922195	-0.7976365
H	1.0382549	-4.1216661	-1.1790739
H	1.3775706	0.3431393	-2.8906128
H	1.5417967	-1.3200724	-3.4926374
H	2.3199814	-0.8409886	-1.9660039
H	-2.3919171	1.0572610	-2.6613820
H	-1.6206783	0.1536184	-3.9797444
H	-0.6626508	1.2920425	-3.0125496
H	-3.7820614	-1.0495393	0.0371917
H	-4.0614422	-1.8919658	-1.5010194
H	-3.8329062	-0.1340221	-1.4949182
H	-2.5103035	-2.8314712	1.1568951
H	-1.0950994	-3.8954010	0.9494962
H	-2.4941943	-4.0516938	-0.1386762
C	-1.9958587	2.1080523	-0.0318113
C	-2.1248883	3.4857279	-0.1713159
C	-0.9636477	4.2538241	-0.3164235
C	0.2794933	3.6269795	-0.2860544
C	1.5662645	1.4407103	0.0293816
H	-1.0291042	5.3379579	-0.4325733
H	1.1996947	4.2078402	-0.3539064
H	-2.8620989	1.4562388	0.0886955
H	-3.1147010	3.9442734	-0.1632988
C	2.8434259	1.8747759	-0.3436076
C	1.3846892	0.1578236	0.6126321
C	2.5198048	-0.6339049	0.8397774
C	3.9460395	1.0478286	-0.1282928
C	3.8096606	-0.2136136	0.4816360
H	0.6267613	0.1193430	1.6822764

H	2.9870925	2.8480848	-0.8183930
H	4.9391816	1.3914384	-0.4295410
C	5.0186056	-1.0638703	0.7672194
H	2.4048820	-1.5925511	1.3508894
O	-1.5912383	-0.5404658	1.6182322
H	5.7272027	-1.0514277	-0.0753736
H	4.7415000	-2.1078807	0.9734361
H	5.5604185	-0.6841055	1.6504073

Co-Complex 7b

SCF energy:	–2519.829161 hartree
Zero-point correction:	+0.474268 hartree
Enthalpy correction:	+0.501482 hartree
Free energy correction:	+0.409400 hartree
Quasiharmonic free energy correction:	+0.427689 hartree

Cartesian Coordinates

Co	-0.2973891	-0.5467997	-0.0291645
C	-2.0107982	-1.8198446	-0.2674662
C	-2.1398134	-0.6200091	-1.0485852
C	-3.3242220	0.2947489	-1.0302496
C	-2.9466782	-2.2514822	0.8147514
C	-0.8531150	-2.5284680	-0.6870783
C	-0.2380778	-1.7534290	-1.7358559
C	0.9887148	-2.1208293	-2.5070705
C	-0.4372578	-3.8818574	-0.1973007
C	-1.0437140	-0.5926146	-1.9785960
C	-0.8223182	0.3973210	-3.0761484
H	0.2475653	0.5871694	-3.2379875
H	-1.2983456	1.3612121	-2.8588129
H	-1.2427069	0.0102851	-4.0201544
H	-3.5771266	0.6165571	-0.0108675
H	-4.2061498	-0.2201569	-1.4474044
H	-3.1529783	1.1912817	-1.6387938
H	-2.4212932	-2.7875216	1.6183820
H	-3.7048756	-2.9400475	0.4038594
H	-3.4813583	-1.3977433	1.2519919
H	0.5763998	-4.1458155	-0.5272272
H	-1.1141638	-4.6584473	-0.5918938
H	-0.4721512	-3.9547695	0.9010602
H	1.6502603	-2.7888367	-1.9394491
H	1.5674013	-1.2305947	-2.7902019
H	0.7070971	-2.6424715	-3.4375760
C	0.1477574	1.3299363	-0.2143172
C	1.5407798	1.5643417	-0.1944195
C	-0.7048894	2.4261513	-0.3603182
C	2.0503001	2.8630923	-0.3707200
C	-0.2129099	3.7323598	-0.5306943
H	-1.7859995	2.2867141	-0.3457062
C	1.1797764	3.9324319	-0.5467274
H	3.1275774	3.0451987	-0.3614874
C	-1.1505925	4.8979911	-0.7080161
H	1.5797734	4.9399066	-0.6869645
N	1.6302130	-0.7407547	0.2976431
C	2.3576336	0.3900671	0.0978856
C	2.2331639	-1.8719921	0.6896509
C	3.7547027	0.3600562	0.2361265
C	3.6104095	-1.9619336	0.8571860
H	1.5868625	-2.7291970	0.8732425
C	4.3847134	-0.8223815	0.6087495
H	4.3339002	1.2667009	0.0613724
H	4.0610532	-2.9034601	1.1738125
H	5.4711323	-0.8557770	0.7179599
O	-0.5076044	-0.3891960	1.9418412
C	-1.2285008	0.2999023	2.6479346
O	-2.2463022	0.9569951	2.1048194

C	-0.9923982	0.4491588	4.1219862
H	-0.2392696	-0.2751385	4.4524612
H	-1.9246577	0.3087670	4.6912317
H	-0.6184494	1.4679629	4.3220142
H	-2.7197662	1.4947365	2.7589568
H	-0.9458144	5.6918126	0.0290687
H	-2.2024494	4.5943767	-0.6018807
H	-1.0300916	5.3530373	-1.7056102

Co-Complex 8b

SCF energy:	–2290.694961 hartree
Zero-point correction:	+0.410117 hartree
Enthalpy correction:	+0.432337 hartree
Free energy correction:	+0.351746 hartree
Quasiharmonic free energy correction:	+0.368080 hartree

Cartesian Coordinates

Co	0.4344620	0.0415594	-0.3430768
C	2.4228798	-0.2296798	-1.0813671
C	2.2142481	1.0973774	-0.5028017
C	2.4290630	2.3771886	-1.2449294
C	2.8079132	-0.4652861	-2.5045950
C	2.1899915	-1.1960813	-0.0859303
C	1.7375858	-0.4920736	1.0969555
C	1.3470298	-1.1191081	2.3931045
C	2.4070692	-2.6721428	-0.1815068
C	1.8706902	0.9318488	0.8617628
C	1.7273398	1.9791586	1.9162912
H	0.8315188	1.8238747	2.5321000
H	1.6812600	2.9905017	1.4960028
H	2.6058907	1.9349390	2.5823581
H	1.8794803	2.3911804	-2.1985514
H	3.4985160	2.5049408	-1.4833056
H	2.1121920	3.2492576	-0.6590216
H	2.6819388	-1.5150995	-2.8012401
H	3.8671689	-0.1967574	-2.6603046
H	2.2178657	0.1620226	-3.1906257
H	1.6882298	-3.2395624	0.4253780
H	3.4129607	-2.9180105	0.1997917
H	2.3591460	-3.0375397	-1.2171857
H	0.8772336	-2.1012998	2.2446509
H	0.6475759	-0.4828450	2.9521839
H	2.2401254	-1.2697559	3.0252305
C	-0.9889220	1.2362908	0.1155469
C	-2.2309143	0.5752214	0.2741780
C	-0.9556121	2.6264764	0.2550895
C	-3.3825530	1.2957143	0.6174708
C	-2.1064739	3.3705721	0.5759111
H	-0.0249222	3.1751976	0.1061567
C	-3.3135861	2.6792851	0.7748367
H	-4.3422161	0.7906222	0.7487882
C	-2.0540585	4.8693231	0.7079333
H	-4.2153857	3.2363203	1.0415977
N	-0.9905469	-1.2990608	-0.4589668
C	-2.2089734	-0.8548993	-0.0341246
C	-0.8503821	-2.5635996	-0.8891282
C	-3.2991063	-1.7319427	0.0157477
C	-1.8998920	-3.4761093	-0.8818388
H	0.1346358	-2.8488125	-1.2539664
C	-3.1434041	-3.0513752	-0.4034922
H	-4.2650083	-1.3733243	0.3710563
H	-1.7403052	-4.4929495	-1.2424620
H	-3.9894030	-3.7416726	-0.3694888
H	-1.0350427	5.2561267	0.5629760
H	-2.4045242	5.1921006	1.7019520
H	-2.7092684	5.3534377	-0.0351812

Co-Complex 9b

SCF energy:	–3489.520856 hartree
Zero-point correction:	+0.646504 hartree
Enthalpy correction:	+0.684959 hartree
Free energy correction:	+0.561832 hartree
Quasiharmonic free energy correction:	+0.605757 hartree

Cartesian Coordinates

Co	1.7909419	0.0284035	-0.9819878
C	2.9756864	1.6703738	-1.4670718
C	3.6776578	0.8635565	-0.5188400
C	4.2375897	1.2955370	0.7965328
C	2.6075705	3.1137771	-1.3271913
C	2.8077026	0.8884585	-2.6824634
C	3.3304817	-0.3989606	-2.4526062
C	3.4529375	-1.5093650	-3.4459869
C	2.1791196	1.4000769	-3.9378845
C	3.8034420	-0.4482087	-1.0791967
C	4.4462678	-1.6243115	-0.4156911
H	4.0401608	-2.5728380	-0.7946018
H	4.2891729	-1.6021540	0.6718749
H	5.5346361	-1.6332833	-0.5966447
H	3.7434763	2.1947106	1.1835097
H	5.3090429	1.5285953	0.6717529
H	4.1503672	0.5087087	1.5574291
H	1.5995509	3.3114675	-1.7201742
H	3.3114264	3.7516596	-1.8880749
H	2.6323111	3.4403082	-0.2788259
H	1.8664311	0.5839711	-4.6034522
H	2.8934029	2.0311765	-4.4934636
H	1.2959901	2.0173736	-3.7199765
H	2.6832792	-1.4546819	-4.2291914
H	3.3973509	-2.4984865	-2.9712874
H	4.4330283	-1.4499489	-3.9498468
C	1.3162521	0.1461114	0.8807712
C	1.0333199	-1.1122584	1.4559962
C	1.1649352	1.2896239	1.6640701
C	0.6918166	-1.2098598	2.8160558
C	0.7926862	1.2128252	3.0182392
H	1.3222894	2.2784564	1.2255553
C	0.5814769	-0.0569133	3.5878598
H	0.4827121	-2.1811759	3.2709991
C	0.6456239	2.4631669	3.8453728
H	0.3071215	-0.1352256	4.6430167
N	1.1228569	-1.8073770	-0.7930279
C	0.9564499	-2.2106263	0.4964544
C	0.8645702	-2.6474826	-1.8042440
C	0.6334436	-3.5473278	0.7753889
C	0.4958654	-3.9726272	-1.5920541
H	0.9469153	-2.2371219	-2.8103897
C	0.4092829	-4.4344606	-0.2737830
H	0.5458792	-3.8767923	1.8109680
H	0.2874113	-4.6226896	-2.4425491
H	0.1497059	-5.4754362	-0.0680043
O	-4.6724330	0.3919593	-2.0323231
H	-5.1230611	0.4740354	0.5633632
H	-4.1981625	3.0385164	-1.9464509
H	-5.1027317	4.7869884	-0.3973481
H	-5.0687247	-0.2527781	2.9476527
C	-4.3981920	-0.2659592	0.9042229
C	-4.3596933	-0.6803791	2.2350778
C	-3.8068075	3.1500991	-0.9350584
C	-4.3080160	4.1175416	-0.0611887
S	-3.4009116	-0.1868145	-1.6381629
C	-3.4796126	-0.8196706	0.0075184
O	-2.5931555	-1.0502775	-2.4887952
N	-2.3173615	1.2630226	-1.3703730

C	-2.7933962	2.2978059	-0.4882600
C	-3.4247942	-1.6318629	2.6738833
H	-4.2002768	-1.6615029	4.6989571
C	-3.7894234	4.2375346	1.2327591
H	-3.3296830	-3.1423595	4.2191630
C	-3.3502682	-2.0457258	4.1181736
H	-4.1818295	5.0021566	1.9070021
C	-1.0337658	0.9834861	-1.4178810
C	-2.5449153	-1.7833459	0.4038925
C	-2.5344280	-2.1845832	1.7348441
C	-2.2755963	2.3962810	0.8096849
N	0.0949456	0.7098432	-1.4532433
C	-2.7709893	3.3809678	1.6650681
H	-1.8537724	-2.2095441	-0.3236281
H	-2.4236116	-1.6593980	4.5768668
H	-1.8191126	-2.9432520	2.0562033
H	-1.4961723	1.7075396	1.1429080
H	-2.3680597	3.4693179	2.6764977
H	0.3953786	3.3330827	3.2192763
H	1.5870895	2.6991588	4.3716892
H	-0.1335755	2.3507710	4.6147029

Transition State TS3b

SCF energy:	–3489.480955 hartree
Zero-point correction:	+0.645294 hartree
Enthalpy correction:	+0.681969 hartree
Free energy correction:	+0.566007 hartree
Quasiharmonic free energy correction:	+0.595909 hartree
Imaginary Frequency	326.2 *i*cm^{-1}

Cartesian Coordinates

Co	0.2544331	1.6975438	-0.3349487
C	-0.5694356	3.1546294	-1.5761269
C	-0.7335913	3.5870440	-0.2116199
C	-2.0163837	3.9168391	0.4818043
C	-1.6342410	2.9577501	-2.6083139
C	0.8437204	3.0689438	-1.8504631
C	1.5417113	3.3412125	-0.6493473
C	3.0249744	3.4489965	-0.4914940
C	1.4283237	2.7017561	-3.1733354
C	0.5538696	3.6389442	0.3829040
C	0.8536092	4.0155075	1.7985096
H	1.7537071	3.5073350	2.1730866
H	0.0185419	3.7597539	2.4660568
H	1.0278196	5.1016061	1.8857472
H	-2.8903753	3.5955989	-0.0975003
H	-2.0922964	5.0076788	0.6256875
H	-2.0785228	3.4452736	1.4732215
H	-1.5112998	1.9937551	-3.1253160
H	-1.5814356	3.7486574	-3.3748796
H	-2.6412310	2.9902077	-2.1722379
H	2.5043320	2.4948920	-3.1012555
H	1.2892723	3.5265635	-3.8922362
H	0.9386490	1.8064726	-3.5822640
H	3.5609545	2.7168563	-1.1134519
H	3.3401962	3.3186184	0.5525188
H	3.3637205	4.4509720	-0.8063455
C	-1.3323384	0.6765687	0.4060841
C	-1.0381233	0.3798801	1.7643295
C	-2.6694329	0.8068039	0.0073400
C	-2.0811423	0.2619884	2.6858193
C	-3.7260332	0.6838496	0.9225903
H	-2.8965202	0.9983912	-1.0427865
C	-3.4053020	0.4161522	2.2634785
H	-1.8748196	0.0735818	3.7419069
C	-5.1556938	0.8001579	0.4717794

H	-4.2113647	0.3243212	2.9957414
N	1.2074686	0.7718449	1.1248621
C	0.3874289	0.2723043	2.0876744
C	2.5343821	0.6424113	1.2433627
C	0.9189662	-0.3132080	3.2440684
C	3.1252802	0.0715915	2.3668432
H	3.1343339	1.0007270	0.4088998
C	2.3008834	-0.3993481	3.3921524
H	0.2496011	-0.7075564	4.0091058
H	4.2108642	-0.0084252	2.4250791
H	2.7316850	-0.8496367	4.2890925
O	-0.2411948	-4.1948431	-0.8769651
H	1.5048488	-3.7374660	0.9956659
H	-2.8291499	-1.7018504	-2.3343043
H	-5.1553196	-2.4879721	-1.8377969
H	3.8039765	-3.3331221	1.8787699
C	2.2185966	-3.1278022	0.4394051
C	3.5073852	-2.9036836	0.9186607
C	-3.0930977	-2.0827405	-1.3453346
C	-4.3857268	-2.5265990	-1.0635453
S	0.2502684	-2.9479741	-1.4473557
C	1.8591398	-2.5805244	-0.7949570
O	0.2198886	-2.6752620	-2.8781487
N	-0.7753169	-1.7102763	-0.6370944
C	-2.1112460	-2.1438668	-0.3492253
C	4.4372408	-2.1509924	0.1804173
H	6.5315441	-2.6608794	0.2053549
C	-4.6886095	-3.0335499	0.2053931
H	6.2127665	-0.9372438	0.4550089
C	5.8405283	-1.9474721	0.6864124
H	-5.6993838	-3.3862102	0.4236609
C	-0.4285917	-0.4178110	-0.9001631
C	2.7552268	-1.8259857	-1.5563599
C	4.0390574	-1.6143039	-1.0558790
C	-2.4014882	-2.6700017	0.9100390
N	0.3933390	0.1885855	-1.5688293
C	-3.6972023	-3.1071438	1.1893441
H	2.4426180	-1.4266902	-2.5207756
H	5.9108317	-2.1089743	1.7722499
H	4.7534708	-1.0336460	-1.6456431
H	-1.6088627	-2.7320799	1.6560164
H	-3.9324261	-3.5185634	2.1734464
H	-5.2518110	1.4600589	-0.4033000
H	-5.8044360	1.1851189	1.2722865
H	-5.5387797	-0.1916208	0.1787726

Co-Complex 10b

SCF energy:	–3489.521270 hartree
Zero-point correction:	+0.647705 hartree
Enthalpy correction:	+0.685687 hartree
Free energy correction:	+0.563565 hartree
Quasiharmonic free energy correction:	+0.605598 hartree

Cartesian Coordinates

Co	1.9267275	-0.4562427	-0.2779924
C	2.8007145	-0.7272201	1.5376651
C	3.6314608	-1.3562426	0.5278527
C	3.9622645	-2.8129565	0.4774518
C	2.1993950	-1.4006850	2.7255215
C	2.7381491	0.6824203	1.2461166
C	3.4669617	0.8989179	0.0411986
C	3.6308874	2.1955937	-0.6790587
C	1.9808163	1.7089732	2.0249188
C	4.0347456	-0.3625603	-0.3974616
C	4.9055171	-0.5376886	-1.6014334
H	4.5489672	0.0597905	-2.4541071

H	4.9633772	-1.5885894	-1.9173590
H	5.9354549	-0.2059417	-1.3845649
H	3.1241869	-3.4300429	0.8308558
H	4.8280355	-3.0284291	1.1257089
H	4.2192304	-3.1395578	-0.5393112
H	1.2148317	-0.9820568	2.9746185
H	2.8541065	-1.2661275	3.6041323
H	2.0745873	-2.4783439	2.5644099
H	1.5511237	2.4761841	1.3672743
H	2.6425864	2.2108502	2.7504035
H	1.1542082	1.2506121	2.5845378
H	2.9387344	2.9558272	-0.3007357
H	3.4568254	2.0828736	-1.7600693
H	4.6611410	2.5693626	-0.5504381
C	-0.9792394	-1.0838695	1.0405372
C	-0.4578376	-2.3765796	0.8074048
C	-1.6215692	-0.7927420	2.2459401
C	-0.6234845	-3.3445607	1.8106900
C	-1.7612498	-1.7530419	3.2589269
H	-2.0317964	0.2095225	2.3897222
C	-1.2490420	-3.0363666	3.0183249
H	-0.2377737	-4.3539999	1.6499543
C	-2.4714916	-1.4134940	4.5418088
H	-1.3437301	-3.8079600	3.7861000
N	1.1737608	-2.0902520	-1.0405689
C	0.1510572	-2.7878714	-0.4846944
C	1.6978922	-2.4911886	-2.2151535
C	-0.3428980	-3.9344101	-1.1307385
C	1.2489133	-3.6137394	-2.8982806
H	2.5069641	-1.8776410	-2.6153455
C	0.2041195	-4.3534818	-2.3397469
H	-1.1794923	-4.4694792	-0.6809647
H	1.7057282	-3.8907890	-3.8494204
H	-0.1881422	-5.2376486	-2.8472130
O	-1.0833521	1.4865386	-2.7367185
H	-2.2808848	3.1608676	1.0065735
H	-2.3776266	-1.5810302	-1.8469931
H	-4.4754481	-2.9427786	-1.9366524
H	-1.0150344	4.9273083	2.2189526
C	-1.3686953	3.5801788	0.5788898
C	-0.6583054	4.5728701	1.2484143
C	-3.2593908	-1.2860400	-1.2738207
C	-4.4341248	-2.0382433	-1.3252388
S	-1.8467056	1.9350972	-1.5739209
C	-0.8947916	3.1259505	-0.6552016
O	-3.2155875	2.4177943	-1.7126092
N	-1.9873209	0.6232087	-0.4136003
C	-3.2098522	-0.1291850	-0.4873418
C	0.4987759	5.1450178	0.6859998
H	1.3080727	6.0493281	2.4800909
C	-5.5611244	-1.6225444	-0.6093425
H	2.2400348	6.3992500	0.9975486
C	1.2277681	6.2515668	1.4007266
H	-6.4831449	-2.2066014	-0.6551980
C	-0.8141325	-0.0178565	0.0035168
C	0.2492055	3.6652006	-1.2387944
C	0.9334353	4.6792453	-0.5650003
C	-4.3377133	0.2971341	0.2182719
N	0.3465870	0.2981893	-0.3928307
C	-5.5136012	-0.4516808	0.1550035
H	0.5879583	3.2887341	-2.2048441
H	0.6845241	7.2057451	1.2908367
H	1.8216229	5.1225099	-1.0225279
H	-4.2878427	1.2164456	0.8032756
H	-6.3984528	-0.1191022	0.7023112
H	-2.1148749	-0.4578859	4.9574066
H	-2.3329129	-2.1942877	5.3028564
H	-3.5550659	-1.3046006	4.3650508

Co-Complex 11b

SCF energy:	–3718.637879 hartree
Zero-point correction:	+0.712258 hartree
Enthalpy correction:	+0.754405 hartree
Free energy correction:	+0.625534 hartree
Quasiharmonic free energy correction:	+0.660054 hartree

Cartesian Coordinates

Co	-0.1627234	-1.9427463	-0.0440631
C	-0.3142768	-2.5426046	1.9209339
C	-0.6996425	-3.6700951	1.0875301
C	-2.0387719	-4.3330185	1.1210337
C	-1.1114726	-2.0113395	3.0649005
C	1.0576491	-2.2259584	1.6315181
C	1.4775189	-3.0709944	0.5688299
C	2.8164951	-3.0803873	-0.0898818
C	1.8702231	-1.1674090	2.3006919
C	0.3940116	-3.9962365	0.2635658
C	0.4681171	-5.0666729	-0.7744291
H	0.8649181	-4.6687213	-1.7189811
H	-0.5147250	-5.5155258	-0.9725441
H	1.1420528	-5.8734665	-0.4403087
H	-2.8457081	-3.6018020	1.2715708
H	-2.0884041	-5.0541666	1.9541833
H	-2.2476487	-4.8819736	0.1929144
H	-0.8747854	-0.9634751	3.2842902
H	-0.8871377	-2.6038596	3.9694087
H	-2.1901497	-2.0846726	2.8831899
H	2.5814049	-0.7050545	1.6054893
H	2.4434062	-1.6042592	3.1360623
H	1.2323478	-0.3726751	2.7088348
H	3.3329672	-2.1230831	0.0490672
H	2.7229621	-3.2710932	-1.1677572
H	3.4505628	-3.8760288	0.3374408
C	-1.1837356	0.9124437	1.2281780
C	-2.4083112	0.2264945	1.1308302
C	-0.9136579	1.6954900	2.3585922
C	-3.3327972	0.3654353	2.1824233
C	-1.8202378	1.8112590	3.4186072
H	0.0320401	2.2411474	2.3951578
C	-3.0427649	1.1285438	3.3089574
H	-4.2889146	-0.1597162	2.1254047
C	-1.5160298	2.6774801	4.6116761
H	-3.7761818	1.1975331	4.1161883
N	-2.0161654	-1.5181907	-0.5855635
C	-2.8171133	-0.5623953	-0.0558527
C	-2.4677222	-2.2578251	-1.6184798
C	-4.0894627	-0.3238548	-0.6073756
C	-3.7216154	-2.0845561	-2.1877857
H	-1.7770387	-3.0067695	-2.0030824
C	-4.5491776	-1.0832206	-1.6769460
H	-4.6961838	0.4806288	-0.1919962
H	-4.0297276	-2.7158905	-3.0224520
H	-5.5348765	-0.8945059	-2.1083260
O	1.4548325	1.6175243	-2.5825774
H	2.6020249	3.1045204	1.2491523
H	-1.8486106	1.6208165	-1.8931673
H	-3.7839527	3.1232254	-2.3929036
H	4.5081119	2.2298964	2.5900742
C	3.1981250	2.2664136	0.8842449
C	4.2708849	1.7764545	1.6242655
C	-1.9019515	2.6223129	-1.4610612
C	-2.9798834	3.4655023	-1.7369929
S	1.5866845	2.3766601	-1.3127836
C	2.8975664	1.6714111	-0.3449596
O	1.7781676	3.8180704	-1.4113921
N	0.2141078	2.1706321	-0.3026310

C	-0.8754151	3.0632529	-0.6161062
C	5.0671259	0.7213930	1.1411992
H	7.0994907	0.9250128	1.8288816
C	-3.0205260	4.7502214	-1.1858674
H	6.0139946	0.1682913	3.0079366
C	6.2484634	0.2304328	1.9340478
H	-3.8599861	5.4132757	-1.4074416
C	-0.1505225	0.8486874	0.1488127
C	3.6624991	0.6243413	-0.8518973
C	4.7475216	0.1611966	-0.1060476
C	-0.9093495	4.3474071	-0.0670509
N	0.3999388	-0.1869375	-0.3017455
C	-1.9825457	5.1911563	-0.3578033
H	3.4086104	0.1798664	-1.8123788
H	6.5846922	-0.7592178	1.5928105
H	5.3615898	-0.6517167	-0.5014710
H	-0.0905961	4.6756434	0.5737424
H	-2.0100129	6.1980447	0.0648029
O	0.4331789	-2.2690526	-2.0938931
C	0.6468024	-1.3382865	-2.8716542
O	-0.2064389	-0.3442240	-2.9759253
C	1.8466714	-1.3211025	-3.7766451
H	2.5847215	-2.0652356	-3.4560035
H	2.2874890	-0.3138598	-3.7968985
H	1.5187782	-1.5540792	-4.8032700
H	0.2937731	0.5103131	-3.0882668
H	-0.4328302	2.7866309	4.7689675
H	-1.9620061	2.2706213	5.5315648
H	-1.9317201	3.6899231	4.4677799

Co-Complex 12b

SCF energy:	–2612.090673 hartree
Zero-point correction:	+0.472797 hartree
Enthalpy correction:	+0.501752 hartree
Free energy correction:	+0.404959 hartree
Quasiharmonic free energy correction:	+0.427185 hartree

Cartesian Coordinates

Co	-1.4329740	-0.7472574	0.1650973
O	-1.1445289	-1.4087841	2.0361733
O	-2.6177642	0.0794322	1.5340931
N	-0.1953972	0.8023891	0.3323556
C	1.1589285	0.8019510	0.3843611
C	-1.3518351	-0.7486151	-1.9330098
C	-0.7920601	-1.9682979	-1.4093112
C	0.5325265	-2.5673225	-1.7487436
C	-0.6437578	0.2328617	-2.8064100
C	-2.6935407	-0.6397724	-1.4607732
C	-2.9881987	-1.8204611	-0.6681648
C	-4.2927281	-2.1011456	0.0008096
C	-3.6550752	0.4735903	-1.7154881
C	-1.8198077	-2.6331372	-0.6523999
C	-1.6267233	-3.9190846	0.0775423
C	-2.0539500	-0.6172462	2.4383994
C	-2.4081246	-0.4783439	3.8832522
H	-3.4479194	-0.1435971	3.9937275
H	-1.7508647	0.2841807	4.3334738
H	-2.2422197	-1.4269192	4.4112055
H	-2.3863518	-4.0693380	0.8549315
H	-0.6365119	-3.9563127	0.5529752
H	-1.6910562	-4.7624665	-0.6309403
H	1.1860381	-1.8504603	-2.2569300
H	0.3817722	-3.4208649	-2.4316710
H	1.0543938	-2.9454162	-0.8595981
H	-1.0305072	1.2521557	-2.6734135
H	-0.7964467	-0.0422242	-3.8638249

H	0.4387584	0.2521143	-2.6278420
H	-4.1957921	0.7495508	-0.7979395
H	-4.4077090	0.1606652	-2.4589392
H	-3.1534454	1.3674517	-2.1089505
H	-4.7282041	-1.1860916	0.4246064
H	-4.1842478	-2.8357977	0.8099892
H	-5.0108189	-2.5115164	-0.7291424
C	-0.8504191	1.9834091	0.2852873
C	-0.2022912	3.2108578	0.2681049
C	1.1922279	3.2277121	0.3162437
C	1.8679868	2.0142308	0.3810890
C	1.9360430	-0.4648413	0.4535800
H	1.7448412	4.1697053	0.3102434
H	2.9561261	1.9864541	0.4378653
H	-1.9379203	1.9254904	0.2706691
H	-0.7875756	4.1305597	0.2236715
C	2.9431918	-0.7287976	-0.5072161
C	1.7816243	-1.3734658	1.5063578
C	2.5971456	-2.5016775	1.6030895
C	3.7613019	-1.8636417	-0.3972037
C	3.6067707	-2.7652690	0.6627926
H	1.0147185	-1.1987234	2.2579811
H	4.5174659	-2.0443651	-1.1639408
C	4.5091044	-3.9615020	0.8028712
H	2.4523583	-3.1894024	2.4404736
C	3.0691255	0.1133285	-1.6621432
N	3.1104721	0.7756398	-2.6152107
H	4.9503991	-4.2532330	-0.1609969
H	3.9691285	-4.8263533	1.2164002
H	5.3408177	-3.7366553	1.4925302

2-(4-Methylphenyl)-pyridine (1b)

SCF energy:	–518.609226 hartree
Zero-point correction:	+0.196925 hartree
Enthalpy correction:	+0.206963 hartree
Free energy correction:	+0.156050 hartree
Quasiharmonic free energy correction:	+0.167287 hartree

Cartesian Coordinates

C	2.7910811	1.1915200	-0.0658236
C	1.3957747	1.2286576	-0.0663444
C	3.4864667	-0.0228977	0.0100839
C	2.7292714	-1.2046556	0.0826570
C	1.3385183	-1.1734688	0.0808724
C	0.6408459	0.0470161	0.0092985
C	-0.8470624	0.0418107	0.0132857
C	-1.6069515	1.2275018	0.0551598
N	-1.4477434	-1.1607209	-0.0202116
C	-2.7760981	-1.2294323	-0.0198465
C	-3.6077396	-0.1059677	0.0134446
C	-2.9978408	1.1493698	0.0535531
H	0.9022163	2.1994672	-0.1352224
H	3.3516563	2.1284983	-0.1281827
C	4.9928210	-0.0714517	0.0117043
H	3.2441657	-2.1683997	0.1414910
H	0.7551013	-2.0930597	0.1343540
H	-3.5991555	2.0617046	0.0861593
H	-1.1197721	2.2016396	0.0953261
H	-3.2112222	-2.2360788	-0.0479059
H	-4.6942606	-0.2157886	0.0107019
H	5.4312311	0.9356268	-0.0487918
H	5.3737454	-0.6584050	-0.8411682
H	5.3753509	-0.5528461	0.9275954

5-Methyl-2-(2-pyridinyl)-benzonitrile (3b)

SCF energy:	–610.853250 hartree
Zero-point correction:	+0.195677 hartree
Enthalpy correction:	+0.207423 hartree
Free energy correction:	+0.153856 hartree
Quasiharmonic free energy correction:	+0.160396 hartree

Cartesian Coordinates

C	2.4844314	-1.8109914	0.1682096
C	1.1061102	-1.6341443	0.1442805
C	3.3625341	-0.7157379	0.0931843
C	2.8003290	0.5563119	-0.0162563
C	1.4037966	0.7484024	-0.0376221
C	0.5246721	-0.3572464	0.0580077
C	-0.9619017	-0.2596570	0.0525122
N	-1.6097727	-1.3293448	-0.4409655
C	-1.6597821	0.8473879	0.5679027
C	-3.0541610	0.8441870	0.5305584
C	-3.7169126	-0.2593630	-0.0064260
C	-2.9395756	-1.3258536	-0.4706130
H	0.4334762	-2.4911144	0.1886791
H	2.8933743	-2.8224986	0.2465493
C	4.8552449	-0.9174217	0.1204985
H	3.4469423	1.4325214	-0.0992345
C	0.9461286	2.0986115	-0.2088207
H	-3.4199509	-2.2200182	-0.8857229
H	-3.6134483	1.6963570	0.9245554
H	-4.8069630	-0.3024453	-0.0580339
N	0.6273442	3.2066773	-0.3501505
H	-1.1304631	1.6946801	1.0000470
H	5.3951857	0.0380158	0.0528677
H	5.1839858	-1.5541344	-0.7176595
H	5.1695356	-1.4206215	1.0500425

9 Optimization with B3LYP-D3BJ/def2-SVP for 1c (R = F)

Co-Complex 5c

SCF energy:	–2579.791143 hartree
Zero-point correction:	+0.439321 hartree
Enthalpy correction:	+0.465515 hartree
Free energy correction:	+0.376789 hartree
Quasiharmonic free energy correction:	+0.391064 hartree

Cartesian Coordinates

Co	-0.9194844	-0.0043384	0.0232015
O	-0.8500344	-0.5348443	1.9549293
O	-2.1223639	1.0316719	1.2155644
N	0.4647480	1.4442291	0.1576352
C	1.8151560	1.3491001	0.2835850
C	-1.0815944	0.0104137	-2.0491425
C	-0.2277240	-1.0922688	-1.6449356
C	1.1419770	-1.3515584	-2.1739634
C	-0.6770345	1.1131201	-2.9719787
C	-2.3664944	-0.1913497	-1.4670753
C	-2.2975252	-1.3659593	-0.6344654
C	-3.4089989	-1.9263272	0.1911071
C	-3.5567162	0.6999577	-1.5821766
C	-0.9868980	-1.9413013	-0.7912310
C	-0.5117398	-3.1706166	-0.0934580
C	-1.7034952	0.3690577	2.2193985
C	-2.1522436	0.6636244	3.6124338
H	-3.1540092	1.1128874	3.6074708
H	-1.4503420	1.3867681	4.0601566
H	-2.1333213	-0.2502047	4.2213304
H	-0.7250549	-3.1129411	0.9847043
H	0.5658128	-3.3251554	-0.2234893
H	-1.0401567	-4.0533515	-0.4902688
H	1.6941423	-0.4215750	-2.3548496
H	1.0592299	-1.8794055	-3.1400567
H	1.7377055	-1.9757682	-1.4984050
H	-1.2957368	2.0100075	-2.8351813
H	-0.7858738	0.7889893	-4.0208015
H	0.3734492	1.3979016	-2.8222132
H	-3.9550080	0.9527594	-0.5884119
H	-4.3544254	0.1841148	-2.1421471
H	-3.3234200	1.6330470	-2.1109907
H	-4.0906694	-1.1360131	0.5341221
H	-3.0225806	-2.4556819	1.0730344
H	-4.0018664	-2.6469691	-0.3972740
C	-0.0976192	2.6674235	0.0251043
C	0.6316893	3.8469606	-0.0008107
C	2.0192281	3.7697353	0.1334135
C	2.6019205	2.5172415	0.2803397
C	2.5018157	0.0401544	0.4048029
H	2.6354708	4.6716835	0.1361309
H	3.6784603	2.4214066	0.4205886
H	-1.1840062	2.6870015	-0.0514991
H	0.1146593	4.8009355	-0.1138093
C	3.6832914	-0.1752544	-0.3328327
C	2.0513118	-0.9746059	1.2650449
C	2.7450801	-2.1802758	1.3680315
C	4.3791896	-1.3781077	-0.2456340
C	3.8966701	-2.3724844	0.6061835
H	1.1650911	-0.8206246	1.8762302
H	4.0493983	0.5961797	-1.0136151

H	5.2852850	-1.5598556	-0.8260645
F	4.5540423	-3.5266375	0.6973747
H	2.4139228	-2.9696167	2.0454029

Transition State TS1c

SCF energy:	–2579.777057 hartree
Zero-point correction:	+0.438152 hartree
Enthalpy correction:	+0.463874 hartree
Free energy correction:	+0.376549 hartree
Quasiharmonic free energy correction:	+0.389393 hartree
Imaginary Frequency	77.0 $i$cm^{-1}

Cartesian Coordinates

Co	-0.7848661	-0.1141536	-0.0584956
O	-0.8606721	-1.8186619	2.0444347
N	-0.1805741	1.6904307	0.3897411
C	1.1397678	1.9806502	0.4822254
C	-0.9231409	0.2870066	-2.1046193
C	0.0102273	-0.7634241	-1.9145770
C	1.4126181	-0.8010134	-2.4236870
C	-0.6527962	1.5957732	-2.7679381
C	-2.1798972	-0.1183974	-1.5095568
C	-2.0271019	-1.4788077	-1.0363886
C	-3.0939814	-2.3047040	-0.4014628
C	-3.4479902	0.6645469	-1.4614878
C	-0.6825707	-1.8608515	-1.2530526
C	-0.0768125	-3.1605248	-0.8408898
C	-1.7665107	-1.0019966	2.2413350
C	-2.7168618	-1.1037297	3.4088459
H	-2.9990298	-0.1080605	3.7776236
H	-2.2649909	-1.7031481	4.2092509
H	-3.6372009	-1.6091101	3.0719612
H	-0.3092070	-3.3725260	0.2127914
H	1.0127199	-3.1595769	-0.9660786
H	-0.4910541	-3.9771251	-1.4556725
H	1.8255030	0.2062117	-2.5593272
H	1.4354726	-1.3072665	-3.4039096
H	2.0784945	-1.3532770	-1.7502359
H	-1.2935516	2.3964958	-2.3754468
H	-0.8558424	1.5091778	-3.8488991
H	0.3951389	1.9030681	-2.6505687
H	-3.9223001	0.5752438	-0.4733226
H	-4.1594335	0.2763232	-2.2101439
H	-3.2858313	1.7283470	-1.6794048
H	-3.7923558	-1.6801877	0.1710967
H	-2.6671782	-3.0572799	0.2734165
H	-3.6728445	-2.8300894	-1.1799890
C	-1.1026939	2.6573481	0.5372940
C	-0.7526143	3.9901283	0.7146005
C	0.6053272	4.3222915	0.7581195
C	1.5550430	3.3097141	0.6550271
C	2.0803107	0.8457118	0.4511895
H	0.9195433	5.3588796	0.8991693
H	2.6185646	3.5316432	0.7462676
H	-2.1431408	2.3323279	0.5192746
H	-1.5302421	4.7473465	0.8244852
C	3.3782791	0.9626406	-0.0766814
C	1.6753824	-0.3926511	0.9849489
C	2.5296633	-1.4940379	0.9841583
C	4.2440865	-0.1292255	-0.0805654
C	3.8096348	-1.3467143	0.4516108
H	0.7206365	-0.5185990	1.5125154
H	3.7126169	1.9067976	-0.5113807
H	5.2521068	-0.0572414	-0.4929118
F	4.6382695	-2.3863481	0.4493780
H	2.2190675	-2.4449249	1.4196408

O	-1.9767969	-0.0084001	1.4262615

Co-Complex 6c

SCF energy:	–2579.777950 hartree
Zero-point correction:	+0.438290 hartree
Enthalpy correction:	+0.464723 hartree
Free energy correction:	+0.375685 hartree
Quasiharmonic free energy correction:	+0.389622 hartree

Cartesian Coordinates

Co	-0.5915892	0.0934332	-0.0533568
O	-1.4892196	-1.4056440	2.2326244
N	0.7307514	1.4917923	0.2635340
C	2.0350873	1.1377430	0.3534318
C	-0.6561755	0.4273714	-2.1194450
C	-0.3006344	-0.9242499	-1.8915178
C	0.9038219	-1.6232712	-2.4280106
C	0.1428117	1.4346739	-2.8771829
C	-1.9179139	0.6745394	-1.4503204
C	-2.3812161	-0.5839725	-0.9022231
C	-3.6733246	-0.7984401	-0.1904917
C	-2.6865785	1.9526424	-1.4193368
C	-1.3782985	-1.5519727	-1.1341545
C	-1.4281459	-2.9715494	-0.6759145
C	-1.8210682	-0.2281542	2.3631141
C	-2.6573636	0.2599181	3.5240638
H	-2.3128532	1.2436016	3.8724615
H	-2.6300404	-0.4733656	4.3396774
H	-3.7007853	0.3748954	3.1869354
H	-1.6609499	-3.0152664	0.3981368
H	-0.4786511	-3.4911211	-0.8528151
H	-2.2194119	-3.5127514	-1.2209436
H	1.7235030	-0.9243768	-2.6369614
H	0.6459932	-2.1292374	-3.3741476
H	1.2753379	-2.3879523	-1.7354385
H	-0.0292352	2.4557738	-2.5116262
H	-0.1471410	1.4114446	-3.9413513
H	1.2197600	1.2259637	-2.8220052
H	-3.0902368	2.1411135	-0.4136768
H	-3.5407183	1.8972185	-2.1155256
H	-2.0723659	2.8117898	-1.7194355
H	-3.9871902	0.1058108	0.3471599
H	-3.6013830	-1.6237272	0.5286278
H	-4.4615133	-1.0452100	-0.9222402
C	0.3640862	2.7794079	0.3548310
C	1.2985665	3.8027934	0.4669217
C	2.6557094	3.4663637	0.5012188
C	3.0257625	2.1241101	0.4575824
C	2.3148266	-0.3091828	0.3979025
H	3.4178701	4.2438390	0.5891570
H	4.0723885	1.8304552	0.5426080
H	-0.7083008	2.9755188	0.3431281
H	0.9657116	4.8396183	0.5330765
C	3.5082956	-0.8670813	-0.0908853
C	1.3493364	-1.1673100	0.9633258
C	1.5560378	-2.5439199	1.0394151
C	3.7290066	-2.2418002	-0.0198741
C	2.7511543	-3.0653859	0.5468033
H	0.4632741	-0.7901267	1.4993431
H	4.2659506	-0.2299550	-0.5514611
H	4.6482325	-2.6916003	-0.3996365
F	2.9690185	-4.3744771	0.6160978
H	0.8144032	-3.1926816	1.5068079
O	-1.4984830	0.7006609	1.4995721

Transition State TS2c

SCF energy:	–2579.762492 hartree
Zero-point correction:	+0.434212 hartree
Enthalpy correction:	+0.459810 hartree
Free energy correction:	+0.373297 hartree
Quasiharmonic free energy correction:	+0.386211 hartree
Imaginary Frequency	881.4 $i$cm^{-1}

Cartesian Coordinates

Co	-0.5648090	-0.3895990	-0.0102653
O	0.0190218	0.2364776	2.8844877
N	-0.6729144	1.5523879	-0.0760273
C	0.5191774	2.1976171	-0.1386638
C	-1.1144870	-0.5456287	-2.0490327
C	0.1562266	-1.1584451	-1.8271657
C	1.4244681	-0.8323942	-2.5462590
C	-1.3777776	0.5815566	-2.9917006
C	-2.0727306	-1.1809966	-1.1918978
C	-1.4006493	-2.2676091	-0.5128768
C	-2.0586244	-3.2102138	0.4367561
C	-3.5299339	-0.8776633	-1.0633208
C	-0.0381414	-2.2574755	-0.8986565
C	0.9858564	-3.2764772	-0.5169153
C	-1.1827546	-0.0879453	2.7217818
C	-2.1023850	-0.1457052	3.9122144
H	-3.1511942	-0.0569757	3.6026029
H	-1.8346211	0.6382960	4.6328838
H	-1.9624024	-1.1193229	4.4104120
H	0.9143565	-3.5590566	0.5429846
H	2.0066920	-2.9299187	-0.7152728
H	0.8228341	-4.1927818	-1.1096776
H	1.4560910	0.2208465	-2.8551359
H	1.5138666	-1.4494157	-3.4565469
H	2.3064677	-1.0266873	-1.9229514
H	-2.2761946	1.1498956	-2.7185821
H	-1.5359861	0.1799373	-4.0069702
H	-0.5300327	1.2786460	-3.0426924
H	-3.8256532	-0.8261898	-0.0048203
H	-4.1314783	-1.6714498	-1.5369919
H	-3.7991375	0.0700287	-1.5491228
H	-2.6927934	-2.6654066	1.1500452
H	-1.3254638	-3.7998752	1.0027527
H	-2.7018246	-3.9134844	-0.1187805
C	-1.8259624	2.2353864	-0.1098438
C	-1.8536912	3.6169034	-0.2665791
C	-0.6377770	4.2999618	-0.3853960
C	0.5568932	3.5877899	-0.3131917
C	1.6791778	1.3215857	0.0563121
H	-0.6232236	5.3846409	-0.5131160
H	1.5168098	4.1028431	-0.3584287
H	-2.7388414	1.6474132	-0.0064177
H	-2.8085977	4.1437659	-0.2912820
C	2.9901114	1.6639793	-0.3002717
C	1.3945555	0.0660363	0.6602470
C	2.4583278	-0.8070857	0.9228449
C	4.0352650	0.7725426	-0.0565630
C	3.7551808	-0.4496740	0.5603354
H	0.5956398	0.0900190	1.6861342
H	3.2078723	2.6181703	-0.7848200
H	5.0657642	1.0109240	-0.3261512
F	4.7550504	-1.2932498	0.8100890
H	2.3034135	-1.7495274	1.4497615
O	-1.6642472	-0.4035367	1.5829618

Co-Complex 7c

SCF energy:	–2579.775478 hartree
Zero-point correction:	+0.439080 hartree
Enthalpy correction:	+0.465500 hartree
Free energy correction:	+0.376469 hartree
Quasiharmonic free energy correction:	+0.391522 hartree

Cartesian Coordinates

Co	-0.4238996	-0.2346832	-0.0655043
C	-2.4631702	-0.7923708	-0.4286742
C	-2.0628729	0.2922518	-1.2799713
C	-2.8061973	1.5811245	-1.4407323
C	-3.5808100	-0.7497067	0.5620816
C	-1.6270682	-1.9165286	-0.6775149
C	-0.6837404	-1.5266007	-1.6934293
C	0.3692199	-2.3917729	-2.3073055
C	-1.7967817	-3.2745925	-0.0680625
C	-0.9605543	-0.1734486	-2.0793639
C	-0.2857868	0.5621308	-3.1918415
H	0.7849084	0.3213822	-3.2433434
H	-0.3750568	1.6493197	-3.0764084
H	-0.7402273	0.2811021	-4.1572782
H	-3.0004620	2.0652957	-0.4739179
H	-3.7807801	1.3966146	-1.9231999
H	-2.2593643	2.2895463	-2.0753487
H	-3.3588239	-1.3552873	1.4527059
H	-4.4989973	-1.1609994	0.1086697
H	-3.8025271	0.2776045	0.8798963
H	-0.9446520	-3.9326933	-0.2847847
H	-2.6945265	-3.7698936	-0.4749037
H	-1.9250297	-3.2256283	1.0246438
H	0.6672920	-3.2155862	-1.6454083
H	1.2699566	-1.8144374	-2.5583879
H	-0.0097354	-2.8365924	-3.2430627
C	0.6821534	1.3418165	-0.2696167
C	2.0630376	1.0679570	-0.1153260
C	0.2939073	2.6545887	-0.5376452
C	3.0247706	2.0806408	-0.2778000
C	1.2645976	3.6480469	-0.6839169
H	-0.7494466	2.9483260	-0.6399623
C	2.6301502	3.3824924	-0.5731299
H	4.0892390	1.8635303	-0.1650379
F	0.8668486	4.8982324	-0.9409770
H	3.3512244	4.1903657	-0.7056301
N	1.2708269	-1.0797049	0.4565803
C	2.3719042	-0.2991128	0.2966535
C	1.3886845	-2.3230210	0.9442945
C	3.6464637	-0.8160795	0.5785890
C	2.6207198	-2.8866851	1.2555152
H	0.4632173	-2.8790327	1.0878972
C	3.7725401	-2.1176555	1.0519310
H	4.5275486	-0.1904650	0.4361171
H	2.6720828	-3.9035145	1.6469182
H	4.7600488	-2.5286531	1.2737161
O	-0.7248307	0.1203678	1.8679509
C	-1.2025084	1.0730248	2.4672598
O	-1.8504484	2.0142151	1.7920475
C	-1.0669004	1.2295276	3.9528442
H	-0.6542203	0.3119474	4.3871775
H	-2.0379475	1.4620592	4.4176534
H	-0.3779936	2.0655259	4.1633127
H	-2.1492924	2.7349693	2.3690300

Co-Complex 8c

SCF energy:	–2350.639932 hartree
Zero-point correction:	+0.374982 hartree
Enthalpy correction:	+0.396374 hartree
Free energy correction:	+0.319269 hartree
Quasiharmonic free energy correction:	+0.330775 hartree

Cartesian Coordinates

Co	0.4407732	0.0725213	-0.3416895
C	2.4213691	-0.2347028	-1.0782633
C	2.2293147	1.1071361	-0.5265901
C	2.4483428	2.3695230	-1.2959702
C	2.7966453	-0.5045067	-2.4977736
C	2.1800755	-1.1768627	-0.0617419
C	1.7409575	-0.4424036	1.1080480
C	1.3483202	-1.0373170	2.4181966
C	2.3769829	-2.6572807	-0.1243401
C	1.8914424	0.9742699	0.8425637
C	1.7695719	2.0451024	1.8756517
H	0.8744576	1.9189520	2.4991476
H	1.7435970	3.0492672	1.4365386
H	2.6498223	1.9970947	2.5391990
H	1.9295266	2.3487637	-2.2663992
H	3.5229884	2.5070173	-1.5044788
H	2.1008414	3.2526912	-0.7450067
H	2.6733854	-1.5619129	-2.7668651
H	3.8532715	-0.2346626	-2.6679398
H	2.1988997	0.1028603	-3.1951691
H	1.6402081	-3.2022208	0.4817570
H	3.3728265	-2.9086145	0.2791270
H	2.3415685	-3.0427532	-1.1530245
H	0.8638389	-2.0155279	2.2924304
H	0.6624994	-0.3794593	2.9690789
H	2.2434881	-1.1883144	3.0474228
C	-0.9783255	1.2801028	0.1093307
C	-2.2303857	0.6293112	0.2555839
C	-0.9301904	2.6674006	0.2535837
C	-3.3846178	1.3519114	0.5885295
C	-2.0962839	3.3738574	0.5653181
H	-0.0151981	3.2446045	0.1261020
C	-3.3214479	2.7358987	0.7537300
H	-4.3473755	0.8503454	0.7070709
F	-2.0264731	4.6984187	0.6934461
H	-4.2028340	3.3263046	1.0088650
N	-0.9949536	-1.2537076	-0.4684750
C	-2.2146443	-0.8006758	-0.0565797
C	-0.8599205	-2.5189365	-0.8999294
C	-3.3118715	-1.6694492	-0.0209448
C	-1.9164010	-3.4227802	-0.9069469
H	0.1269824	-2.8111101	-1.2539245
C	-3.1618252	-2.9890631	-0.4415174
H	-4.2794235	-1.3053934	0.3241663
H	-1.7610561	-4.4400504	-1.2681908
H	-4.0137100	-3.6725187	-0.4185572

Co-Complex 9c

SCF energy:	–3549.465813 hartree
Zero-point correction:	+0.611304 hartree
Enthalpy correction:	+0.649035 hartree
Free energy correction:	+0.528726 hartree
Quasiharmonic free energy correction:	+0.569893 hartree

Cartesian Coordinates

Co	1.7869017	0.0483223	-0.9843814
C	2.9471982	1.6997338	-1.4912649
C	3.6599500	0.9179042	-0.5297259
C	4.2132116	1.3787264	0.7786291
C	2.5564221	3.1390953	-1.3735800
C	2.7934304	0.8965844	-2.6944572
C	3.3345590	-0.3798088	-2.4437226
C	3.4779097	-1.5017916	-3.4210968
C	2.1611983	1.3790208	-3.9592896
C	3.8062692	-0.4006124	-1.0694903
C	4.4671453	-1.5560502	-0.3879198
H	4.0707019	-2.5165413	-0.7463964
H	4.3177425	-1.5151083	0.7001186
H	5.5542616	-1.5546427	-0.5764022
H	3.7141153	2.2825770	1.1478664
H	5.2831523	1.6167346	0.6505510
H	4.1322244	0.6058229	1.5544002
H	1.5494989	3.3166399	-1.7786230
H	3.2561986	3.7797353	-1.9363743
H	2.5670674	3.4808241	-0.3298194
H	1.8602400	0.5482275	-4.6118808
H	2.8697933	2.0091959	-4.5231277
H	1.2704286	1.9893905	-3.7538206
H	2.7061530	-1.4743782	-4.2036879
H	3.4445645	-2.4848580	-2.9320745
H	4.4555878	-1.4285759	-3.9277682
C	1.2949230	0.2004918	0.8707736
C	1.0015301	-1.0469192	1.4702022
C	1.1304906	1.3624025	1.6246686
C	0.6283311	-1.1243836	2.8237853
C	0.7191381	1.2648533	2.9545840
H	1.2899711	2.3579681	1.2076515
C	0.4902710	0.0370389	3.5796780
H	0.4149314	-2.0884046	3.2909539
F	0.5184300	2.3912990	3.6504229
H	0.1901815	0.0134465	4.6283661
N	1.1386856	-1.7919442	-0.7614240
C	0.9511377	-2.1671739	0.5331117
C	0.9071948	-2.6580110	-1.7573937
C	0.6346173	-3.4997295	0.8371455
C	0.5466673	-3.9812491	-1.5215252
H	1.0051958	-2.2706372	-2.7710723
C	0.4387531	-4.4132783	-0.1948879
H	0.5305266	-3.8068506	1.8779900
H	0.3610230	-4.6528834	-2.3604835
H	0.1849652	-5.4516076	0.0304089
O	-4.6972002	0.3986881	-2.0151550
H	-5.1121624	0.5131377	0.5872133
H	-4.2193896	3.0480029	-1.9136819
H	-5.0452596	4.8181684	-0.3453268
H	-5.0572327	-0.2149265	2.9703208
C	-4.3983390	-0.2395502	0.9235219
C	-4.3601912	-0.6552710	2.2537015
C	-3.7926407	3.1594278	-0.9166505
C	-4.2486940	4.1398966	-0.0319312
S	-3.4269319	-0.1881197	-1.6298976
C	-3.4957736	-0.8101203	0.0208270
O	-2.6343456	-1.0646788	-2.4814949
N	-2.3290740	1.2532725	-1.3852290
C	-2.7766308	2.2969853	-0.4969253
C	-3.4426525	-1.6265848	2.6863833
H	-4.2520156	-1.7083038	4.6964384
C	-3.6814565	4.2654304	1.2414475
H	-3.2884848	-3.1354627	4.2298525
C	-3.3711467	-2.0419780	4.1304604
H	-4.0368475	5.0440462	1.9201830
C	-1.0481533	0.9651993	-1.4465986

C	-2.5768595	-1.7910188	0.4113276
C	-2.5678412	-2.1946376	1.7420437
C	-2.2160891	2.3981520	0.7821531
N	0.0820105	0.6967571	-1.4840616
C	-2.6605982	3.3983654	1.6466030
H	-1.8984093	-2.2305221	-0.3205419
H	-2.4786610	-1.6022261	4.6090423
H	-1.8683689	-2.9705028	2.0578696
H	-1.4381198	1.6989128	1.0924714
H	-2.2087810	3.4903455	2.6369062

Transition State TS3c

SCF energy:	–3549.422063 hartree
Zero-point correction:	+0.610123 hartree
Enthalpy correction:	+0.646898 hartree
Free energy correction:	+0.531244 hartree
Quasiharmonic free energy correction:	+0.561274 hartree
Imaginary Frequency	363.9 *i*cm^{-1}

Cartesian Coordinates

Co	0.2282610	1.7101026	-0.3260105
C	-0.6429434	3.1410282	-1.5633999
C	-0.8075800	3.5715624	-0.1968826
C	-2.0948792	3.8666464	0.5041612
C	-1.7108210	2.9149853	-2.5864610
C	0.7684238	3.0892583	-1.8484809
C	1.4690677	3.3796327	-0.6517472
C	2.9504419	3.5221371	-0.5057666
C	1.3521323	2.7331238	-3.1746004
C	0.4822127	3.6598663	0.3868185
C	0.7857753	4.0491270	1.7980194
H	1.6947170	3.5550540	2.1701490
H	-0.0416810	3.7883743	2.4729563
H	0.9471591	5.1378767	1.8760887
H	-2.9633423	3.5046553	-0.0595084
H	-2.2096117	4.9563402	0.6296460
H	-2.1284191	3.4131594	1.5054350
H	-1.5714089	1.9507835	-3.0991188
H	-1.6783235	3.7014082	-3.3586727
H	-2.7159406	2.9318464	-2.1451199
H	2.4331028	2.5518093	-3.1098909
H	1.1881480	3.5533614	-3.8934461
H	0.8808730	1.8258366	-3.5786747
H	3.4981469	2.7994278	-1.1284551
H	3.2765467	3.4050831	0.5364903
H	3.2632111	4.5300305	-0.8284801
C	-1.3308247	0.6267089	0.4017674
C	-1.0298821	0.3334087	1.7612405
C	-2.6705054	0.7322513	0.0021903
C	-2.0637777	0.1816312	2.6902851
C	-3.6813703	0.5565074	0.9432907
H	-2.9416992	0.9229545	-1.0355341
C	-3.3965109	0.2890621	2.2818798
H	-1.8456830	-0.0057050	3.7437972
F	-4.9511944	0.6432492	0.5513493
H	-4.2204363	0.1626942	2.9861639
N	1.2045845	0.7997452	1.1298106
C	0.3990725	0.2596077	2.0823911
C	2.5344952	0.7006378	1.2449857
C	0.9454956	-0.3350771	3.2268304
C	3.1404645	0.1222202	2.3565931
H	3.1247450	1.0905198	0.4177430
C	2.3293802	-0.3892748	3.3727644
H	0.2876711	-0.7638516	3.9833173
H	4.2277666	0.0676887	2.4124480
H	2.7719974	-0.8470040	4.2600920

O	-0.2100101	-4.1932568	-0.9544802
H	1.5070981	-3.7351456	0.9588769
H	-2.8524013	-1.6269051	-2.3123036
H	-5.1955243	-2.3171971	-1.7548568
H	3.7898758	-3.3193532	1.8776225
C	2.2243273	-3.1173501	0.4161107
C	3.5042333	-2.8866521	0.9156184
C	-3.0985961	-2.0331793	-1.3288112
C	-4.4003629	-2.4236538	-1.0139615
S	0.2788624	-2.9325058	-1.4954028
C	1.8791593	-2.5662096	-0.8206810
O	0.2592964	-2.6294934	-2.9198644
N	-0.7580128	-1.7200027	-0.6668241
C	-2.0882422	-2.1597604	-0.3672771
C	4.4399348	-2.1243705	0.1945143
H	5.8880392	-2.0688168	1.8093169
C	-4.6820215	-2.9570758	0.2482929
H	6.5331740	-2.6342452	0.2575427
C	5.8349242	-1.9163075	0.7211578
H	-5.7014239	-3.2628159	0.4942901
C	-0.4150561	-0.4157417	-0.8907975
C	2.7811255	-1.8017110	-1.5647277
C	4.0563632	-1.5844792	-1.0448541
C	-2.3570685	-2.7214725	0.8816980
N	0.4142542	0.1993113	-1.5465251
C	-3.6597604	-3.1163567	1.1898916
H	2.4793977	-1.3987366	-2.5310674
H	6.2114016	-0.9083802	0.4868388
H	4.7754768	-0.9965254	-1.6215045
H	-1.5444421	-2.8358635	1.5997495
H	-3.8791590	-3.5530589	2.1667843

Co-Complex 10c

SCF energy:	–3549.464079 hartree
Zero-point correction:	+0.613013 hartree
Enthalpy correction:	+0.649857 hartree
Free energy correction:	+0.532280 hartree
Quasiharmonic free energy correction:	+0.568514 hartree

Cartesian Coordinates

Co	1.9646218	-0.6430281	-0.0238492
C	2.7251515	-0.3951998	-1.9400142
C	3.5435847	0.2441282	-0.9389198
C	3.8978282	1.6923193	-0.8903996
C	2.0051745	0.2735133	-3.0644664
C	2.6491023	-1.7747536	-1.6052814
C	3.5049691	-2.0178002	-0.4472369
C	3.7140352	-3.3540196	0.1900432
C	1.8338784	-2.8009477	-2.3229956
C	4.0606145	-0.7822806	-0.0491113
C	4.9930242	-0.5315727	1.0925271
H	4.9667848	-1.3437267	1.8317632
H	4.7544456	0.4080234	1.6111096
H	6.0306270	-0.4505699	0.7275858
H	3.1063994	2.3188434	-1.3213127
H	4.8206300	1.8711398	-1.4696782
H	4.0814895	2.0334487	0.1370084
H	0.9966358	-0.1433244	-3.1978881
H	2.5623906	0.1303199	-4.0055002
H	1.9104988	1.3540744	-2.8939258
H	1.5733365	-3.6424340	-1.6653569
H	2.4061294	-3.2149892	-3.1710460
H	0.9045074	-2.3660915	-2.7162717
H	2.7706915	-3.9131332	0.2835516
H	4.1654587	-3.2689262	1.1881063
H	4.3910923	-3.9690582	-0.4271616

C	0.2106278	2.0898414	-0.3330802
C	1.0121592	2.3555521	0.8033543
C	-0.0005366	3.0817818	-1.2959819
C	1.5743191	3.6392052	0.9344970
C	0.6005508	4.3275937	-1.1444168
H	-0.6370455	2.8856807	-2.1594587
C	1.3900012	4.6233344	-0.0340192
H	2.1889549	3.8673867	1.8078516
F	0.4112562	5.2574006	-2.0768526
H	1.8422149	5.6119712	0.0597598
N	1.7233894	0.1556280	1.7403034
C	1.2226598	1.4040778	1.9313459
C	1.9567958	-0.6440486	2.7991634
C	0.9426656	1.8454606	3.2366042
C	1.7053795	-0.2571764	4.1091091
H	2.3586282	-1.6337031	2.5731198
C	1.1802237	1.0176425	4.3302263
H	0.5164843	2.8394338	3.3740880
H	1.9110817	-0.9455097	4.9300758
H	0.9522248	1.3605393	5.3420231
O	-3.5561680	-0.1595984	-2.5023849
H	-4.6575410	-1.0564133	-0.3477103
H	-2.0584559	0.7979639	1.6523311
H	-3.6867401	2.2082985	2.9208974
H	-5.1941826	-2.7423312	1.4078593
C	-3.9299108	-1.8185588	-0.0661473
C	-4.2220290	-2.7699619	0.9090024
C	-2.7429321	1.4671238	1.1256748
C	-3.6583527	2.2519585	1.8294798
S	-2.3072763	-0.6234367	-1.9111902
C	-2.6826723	-1.8644219	-0.6985316
O	-1.1815970	-1.0492383	-2.7424785
N	-1.7386371	0.7397300	-0.9792198
C	-2.7040935	1.5282254	-0.2745069
C	-3.2967631	-3.7718311	1.2505938
H	-4.2222261	-4.3903050	3.1084859
C	-4.5478691	3.0783737	1.1360606
H	-4.2742770	-5.6064884	1.8201173
C	-3.6473452	-4.8193410	2.2736277
H	-5.2698384	3.6870340	1.6852144
C	-0.4041795	0.7420863	-0.5309491
C	-1.7388161	-2.8434509	-0.3820402
C	-2.0568159	-3.7917823	0.5893189
C	-3.5910826	2.3565432	-0.9725007
N	0.2782317	-0.3078680	-0.3697499
C	-4.5157635	3.1232159	-0.2622870
H	-0.7795000	-2.8552755	-0.8963286
H	-2.7500850	-5.3071057	2.6818335
H	-1.3316136	-4.5716587	0.8364093
H	-3.5570322	2.3780339	-2.0613665
H	-5.2119226	3.7664633	-0.8051065

Co-Complex 11c

SCF energy:	–3778.581216 hartree
Zero-point correction:	+0.677043 hartree
Enthalpy correction:	+0.718572 hartree
Free energy correction:	+0.592341 hartree
Quasiharmonic free energy correction:	+0.623011 hartree

Cartesian Coordinates

Co	-1.7557209	-0.8191838	-0.0861913
C	-2.2730614	-1.3026324	1.8342528
C	-3.4411955	-1.5353187	0.9988268
C	-4.7604889	-0.8530799	1.1666843
C	-2.2632374	-0.4875852	3.0835113
C	-1.2298205	-2.1916921	1.3975888

C	-1.7040625	-2.8767953	0.2464736
C	-0.9654930	-3.8760976	-0.5801817
C	0.1173344	-2.3255735	2.0239360
C	-3.0954831	-2.4974462	0.0325917
C	-3.9555539	-3.0496315	-1.0536041
H	-3.4092640	-3.0845497	-2.0053908
H	-4.8691534	-2.4571644	-1.1980069
H	-4.2614627	-4.0788245	-0.7990349
H	-4.6376299	0.1971598	1.4672810
H	-5.3524593	-1.3524291	1.9522188
H	-5.3530774	-0.8752539	0.2419537
H	-1.2557325	-0.1424617	3.3440539
H	-2.6332939	-1.1034840	3.9220552
H	-2.9175297	0.3896450	3.0076966
H	0.8846706	-2.5610970	1.2790751
H	0.1050369	-3.1383398	2.7695219
H	0.4145407	-1.4034943	2.5395506
H	0.0896712	-3.9303357	-0.2885249
H	-1.0095025	-3.6136840	-1.6461670
H	-1.4104829	-4.8774233	-0.4541024
C	0.2147795	1.3746614	1.3867663
C	-1.0274465	2.0359512	1.4715889
C	1.0962763	1.4001883	2.4745683
C	-1.3389700	2.7278114	2.6582771
C	0.7380229	2.0625880	3.6429873
H	2.0716673	0.9185480	2.4060032
C	-0.4764144	2.7387082	3.7505641
H	-2.2947305	3.2498714	2.7371837
F	1.5811146	2.0604958	4.6746194
H	-0.7273970	3.2594387	4.6759086
N	-2.4426936	1.0232712	-0.3016434
C	-2.0012354	2.1255902	0.3539160
C	-3.4085742	1.1514545	-1.2349461
C	-2.5118067	3.3934190	0.0201797
C	-3.9705160	2.3732083	-1.5827304
H	-3.7055807	0.2314479	-1.7358895
C	-3.4994307	3.5249050	-0.9502585
H	-2.1071017	4.2703177	0.5254853
H	-4.7498707	2.4128385	-2.3452248
H	-3.8930460	4.5100739	-1.2106106
O	2.2295902	-0.2724908	-2.5812124
H	3.9846237	-0.4070385	1.3059700
H	0.3588966	2.7780411	-1.5451485
H	0.6685033	5.2500657	-1.6997111
H	4.3401112	-2.4831037	2.6247649
C	3.6508302	-1.3711243	0.9185257
C	3.8564486	-2.5390422	1.6462519
C	1.2296982	3.2557402	-1.0923023
C	1.4066500	4.6380872	-1.1758426
S	2.8956250	0.0348460	-1.2945141
C	3.0230201	-1.4512062	-0.3298358
O	4.1968168	0.6871965	-1.3602151
N	1.9438690	1.0549113	-0.2868129
C	2.1734408	2.4704821	-0.4175301
C	3.4717139	-3.7930021	1.1339098
H	4.8087911	-5.3112822	1.8751029
C	2.5348506	5.2328619	-0.6032852
H	3.4786626	-4.9230236	2.9795857
C	3.7375242	-5.0502582	1.9168943
H	2.6796405	6.3132000	-0.6751215
C	0.6384781	0.6443972	0.1512984
C	2.6276600	-2.6751011	-0.8656762
C	2.8600114	-3.8383081	-0.1282046
C	3.3013669	3.0631189	0.1588478
N	-0.0163271	-0.2614993	-0.4247206
C	3.4822417	4.4429595	0.0570785
H	2.1392530	-2.7228170	-1.8373541
H	3.1713581	-5.9047633	1.5197443
H	2.5602914	-4.8026898	-0.5455604

H	4.0336409	2.4391463	0.6707541
H	4.3667079	4.9055371	0.5006250
O	-1.9418918	-1.1218151	-2.2949821
C	-0.9001029	-0.7986324	-2.8710115
O	0.0637106	-1.6763034	-3.0185293
C	-0.6872762	0.5631677	-3.4719043
H	0.3075187	0.9401728	-3.1976032
H	-1.4711465	1.2561434	-3.1483239
H	-0.7137403	0.4750600	-4.5707337
H	0.9359643	-1.2049092	-3.0911408

Co-Complex 12c

SCF energy:	–2672.031340 hartree
Zero-point correction:	+0.437435 hartree
Enthalpy correction:	+0.465683 hartree
Free energy correction:	+0.371743 hartree
Quasiharmonic free energy correction:	+0.390557 hartree

Cartesian Coordinates

Co	-0.8183383	0.1590634	0.3983512
O	0.0932098	-0.3426547	2.1120721
O	-1.1610551	1.3968004	1.9186302
N	0.6387675	1.3652518	-0.2233266
C	1.8764420	1.0412984	-0.6695832
C	-2.6031169	0.3908693	-0.6074568
C	-1.6202318	-0.0815619	-1.5264765
C	-1.1796969	0.6045249	-2.7765746
C	-3.3737617	1.6673487	-0.6823844
C	-2.7401501	-0.5947521	0.4502213
C	-1.8334152	-1.6566696	0.1724770
C	-1.5859300	-2.8679858	1.0060588
C	-3.6754042	-0.4861201	1.6084969
C	-1.1147694	-1.3375306	-1.0323666
C	-0.1773782	-2.2640359	-1.7332651
C	-0.4125126	0.6857155	2.6631880
C	-0.1134609	1.0602512	4.0780886
H	-0.9423937	1.6399177	4.5051355
H	0.7903096	1.6920913	4.0904989
H	0.0883183	0.1626437	4.6777329
H	0.5778589	-2.6781085	-1.0522285
H	0.3352310	-1.7723080	-2.5668825
H	-0.7461473	-3.1130676	-2.1495252
H	-1.3050573	1.6935685	-2.7116409
H	-1.7932943	0.2518068	-3.6228646
H	-0.1313913	0.3950759	-3.0245816
H	-3.4179711	2.1630624	0.2988637
H	-4.4121466	1.4636595	-0.9944028
H	-2.9411319	2.3667345	-1.4097899
H	-3.3679696	-1.1333097	2.4409825
H	-4.6902193	-0.7935375	1.3042672
H	-3.7328258	0.5463314	1.9791134
H	-1.9466480	-2.7403325	2.0344137
H	-0.5143535	-3.1084210	1.0451767
H	-2.1079232	-3.7348268	0.5661386
C	0.2464300	2.6576661	-0.2586295
C	1.0459687	3.6809291	-0.7498236
C	2.3222272	3.3631938	-1.2153108
C	2.7344657	2.0358120	-1.1654501
C	2.3703028	-0.3629891	-0.6463894
H	2.9887396	4.1358454	-1.6045460
H	3.7307765	1.7507009	-1.5035854
H	-0.7473759	2.8657664	0.1356055
H	0.6693958	4.7049231	-0.7570460
C	2.4991887	-1.0815389	0.5490135
C	2.8326004	-0.9640779	-1.8447382
C	3.4105419	-2.2420447	-1.8371212

C	3.0667405	-2.3559683	0.5665807
C	3.5223698	-2.9179479	-0.6257247
H	2.1489035	-0.6419452	1.4804775
H	3.1765480	-2.9149406	1.4977171
F	4.0712340	-4.1276031	-0.6096033
H	3.7558711	-2.7115937	-2.7588296
C	2.6230715	-0.3035450	-3.1015857
N	2.3856072	0.2279246	-4.1060387

2-(4-Fluorophenyl)-pyridine (1c)

SCF energy:	–578.554741 hartree
Zero-point correction:	+0.161798 hartree
Enthalpy correction:	+0.171030 hartree
Free energy correction:	+0.123333 hartree
Quasiharmonic free energy correction:	+0.130421 hartree

Cartesian Coordinates

C	2.8651886	1.1918302	-0.0429638
C	1.4707752	1.2242760	-0.0409404
C	3.5111363	-0.0412125	0.0060222
C	2.7940893	-1.2351681	0.0545974
C	1.4021204	-1.1866307	0.0549773
C	0.7134716	0.0401908	0.0095537
C	-0.7750664	0.0398279	0.0141483
C	-1.5326134	1.2267483	0.0469487
N	-1.3766287	-1.1619643	-0.0111988
C	-2.7050343	-1.2297943	-0.0095452
C	-3.5350402	-0.1049236	0.0170982
C	-2.9238012	1.1498267	0.0472091
H	0.9787905	2.1967525	-0.0854312
H	3.4583566	2.1072379	-0.0839366
F	4.8510738	-0.0786919	0.0049600
H	3.3341141	-2.1833533	0.0913205
H	0.8116283	-2.1023591	0.0900741
H	-3.5239252	2.0630707	0.0727448
H	-1.0459489	2.2015121	0.0768206
H	-3.1408986	-2.2361115	-0.0308335
H	-4.6216378	-0.2133439	0.0161549

5-Fluoro-2-(2-pyridinyl)-benzonitrile (3c)

SCF energy:	–670.795966 hartree
Zero-point correction:	+0.160237 hartree
Enthalpy correction:	+0.171263 hartree
Free energy correction:	+0.120369 hartree
Quasiharmonic free energy correction:	+0.124160 hartree

Cartesian Coordinates

C	-2.6391214	-1.7816836	-0.1983643
C	-1.2498144	-1.6741976	-0.1665052
C	-3.4027387	-0.6210912	-0.1005751
C	-2.8094290	0.6283812	0.0298251
C	-1.4074813	0.7283852	0.0587558
C	-0.6029651	-0.4346346	-0.0419344
C	0.8800024	-0.3546631	-0.0338395
C	1.6678630	-1.3595191	0.5568804
N	1.4249778	0.7203383	-0.6175047
C	2.7501308	0.8421325	-0.6408424
C	3.6162316	-0.1094193	-0.0920087
C	3.0559256	-1.2319699	0.5207160
H	-0.6476908	-2.5785785	-0.2708577
H	-3.1371817	-2.7464909	-0.3090220
F	-4.7365392	-0.7080443	-0.1320832
H	-3.4332258	1.5185635	0.1168566
C	-0.8593065	2.0449337	0.2499428

H	3.6918051	-1.9950879	0.9765503
H	1.2011105	-2.2099190	1.0566159
H	3.1468824	1.7431830	-1.1226284
H	4.6979600	0.0328267	-0.1393393
N	-0.5404654	3.1425350	0.4520020

10 Optimization with B3LYP-D3BJ/def2-SVP for 1d (R = COMe)

Co-Complex 5d

SCF energy:	–2633.184853 hartree
Zero-point correction:	+0.484511 hartree
Enthalpy correction:	+0.513072 hartree
Free energy correction:	+0.417947 hartree
Quasiharmonic free energy correction:	+0.435965 hartree

Cartesian Coordinates

Co	-0.8978160	-0.0044041	0.0281412
O	-0.8443894	-0.4891123	1.9725352
O	-2.1107730	1.0587689	1.1860832
N	0.4858261	1.4528407	0.1439399
C	1.8344744	1.3647216	0.2904847
C	-1.0664393	-0.0332737	-2.0406311
C	-0.1974291	-1.1191724	-1.6219887
C	1.1720506	-1.3703143	-2.1551725
C	-0.6802277	1.0571328	-2.9858731
C	-2.3472193	-0.2427977	-1.4514888
C	-2.2577248	-1.3985244	-0.5958758
C	-3.3556680	-1.9596243	0.2470009
C	-3.5504050	0.6287232	-1.5812961
C	-0.9404738	-1.9621496	-0.7495665
C	-0.4528861	-3.1769342	-0.0346274
C	-1.7011506	0.4186556	2.2087722
C	-2.1647056	0.7425385	3.5904024
H	-3.1648466	1.1946947	3.5654638
H	-1.4650337	1.4716467	4.0319112
H	-2.1554275	-0.1592602	4.2172383
H	-0.6025656	-3.0755118	1.0513170
H	0.6109131	-3.3674094	-0.2175908
H	-1.0259143	-4.0587771	-0.3655257
H	1.7178660	-0.4371533	-2.3396367
H	1.0881478	-1.8991416	-3.1206586
H	1.7729698	-1.9907727	-1.4808447
H	-1.3080898	1.9494729	-2.8607328
H	-0.7920555	0.7139973	-4.0283279
H	0.3679693	1.3561622	-2.8479503
H	-3.9528996	0.8923134	-0.5921498
H	-4.3397407	0.0916475	-2.1331473
H	-3.3305897	1.5562970	-2.1253444
H	-4.0536095	-1.1751434	0.5701284
H	-2.9557822	-2.4565121	1.1416995
H	-3.9339033	-2.7084695	-0.3204175
C	-0.0770559	2.6730366	-0.0060367
C	0.6498225	3.8551415	-0.0211769
C	2.0341982	3.7839372	0.1411746
C	2.6189012	2.5329963	0.2987793
C	2.5188631	0.0538604	0.4164571
H	2.6468075	4.6882572	0.1535058
H	3.6936981	2.4391703	0.4534117
H	-1.1617316	2.6895620	-0.1039328
H	0.1318789	4.8069326	-0.1478812
C	3.6996143	-0.1670739	-0.3169808
C	2.0482714	-0.9631159	1.2636351
C	2.7169525	-2.1813240	1.3326286
C	4.3671832	-1.3885266	-0.2434702
C	3.8715488	-2.4182458	0.5710781
H	1.1670575	-0.7976362	1.8785457
H	4.0802340	0.6092954	-0.9841944

H	5.2681513	-1.5377127	-0.8404276
H	2.3592312	-2.9825219	1.9820600
C	4.5090584	-3.7841037	0.6626469
O	3.9465362	-4.6645151	1.2801558
C	5.8335262	-4.0126562	-0.0293279
H	6.5927014	-3.2975601	0.3268253
H	5.7351460	-3.8704891	-1.1182651
H	6.1695544	-5.0373931	0.1716880

Transition State TS1d

SCF energy:	–2633.170941 hartree
Zero-point correction:	+0.483327 hartree
Enthalpy correction:	+0.511484 hartree
Free energy correction:	+0.417166 hartree
Quasiharmonic free energy correction:	+0.435546 hartree
Imaginary Frequency	90.7 *i*cm^{-1}

Cartesian Coordinates

Co	-1.0530020	-0.3300343	-0.0603552
O	-0.4336014	-2.0061040	1.9260849
N	-1.1497917	1.5514069	0.4779945
C	-0.0336925	2.3155497	0.5571706
C	-1.3491773	0.0949542	-2.0879335
C	-0.1030598	-0.5662233	-1.9473711
C	1.2142771	-0.0888461	-2.4604771
C	-1.5673886	1.4444897	-2.6857005
C	-2.3737739	-0.7555725	-1.5182929
C	-1.7443372	-1.9955470	-1.1122836
C	-2.4441055	-3.1727719	-0.5225464
C	-3.8360682	-0.4745042	-1.4407972
C	-0.3542193	-1.8656536	-1.3392360
C	0.6810651	-2.8816703	-0.9900362
C	-1.5776670	-1.6137888	2.1833751
C	-2.3725886	-2.1273629	3.3577265
H	-2.9811401	-1.3274355	3.8016727
H	-1.6991493	-2.5653611	4.1051426
H	-3.0611469	-2.9121096	3.0031929
H	0.5300791	-3.2463012	0.0358139
H	1.6940692	-2.4681348	-1.0673755
H	0.6095659	-3.7403808	-1.6786568
H	1.2415330	1.0021122	-2.5691174
H	1.4011845	-0.5285010	-3.4552719
H	2.0431857	-0.3847172	-1.8068424
H	-2.4400834	1.9504955	-2.2518803
H	-1.7487340	1.3423895	-3.7691761
H	-0.6910874	2.0937498	-2.5559903
H	-4.2423021	-0.7876841	-0.4679539
H	-4.3707113	-1.0397584	-2.2231843
H	-4.0603190	0.5898069	-1.5899432
H	-3.2972780	-2.8598299	0.0937928
H	-1.7658541	-3.7701571	0.0997886
H	-2.8298230	-3.8204810	-1.3280486
C	-2.3608672	2.0954996	0.6860308
C	-2.5290372	3.4557075	0.9184739
C	-1.3950271	4.2729235	0.9502863
C	-0.1388671	3.6957949	0.7806405
C	1.2628985	1.6185579	0.4579067
H	-1.4878640	5.3460928	1.1314677
H	0.7676573	4.2968032	0.8568400
H	-3.2041214	1.4046497	0.6722325
H	-3.5295615	3.8604421	1.0779241
C	2.3970082	2.2201241	-0.1159411
C	1.3690595	0.3123037	0.9671727
C	2.5741293	-0.3872460	0.8943500
C	3.5966943	1.5193763	-0.1895300
C	3.7010681	0.2092369	0.3125576

H	0.5505411	-0.1605957	1.5213732
H	2.3339414	3.2286456	-0.5305524
H	4.4846264	1.9624785	-0.6446777
H	2.6160497	-1.3927798	1.3148533
O	-2.1788138	-0.7435544	1.4258767
C	5.0363613	-0.4891790	0.1897199
O	5.9486989	0.0657981	-0.3841981
C	5.1848816	-1.8681192	0.7909602
H	4.4724059	-2.5757626	0.3357605
H	4.9774562	-1.8479248	1.8732697
H	6.2085434	-2.2234969	0.6205083

Co-Complex 6d

SCF energy:	–2633.172359 hartree
Zero-point correction:	+0.483502 hartree
Enthalpy correction:	+0.512306 hartree
Free energy correction:	+0.416877 hartree
Quasiharmonic free energy correction:	+0.434437 hartree

Cartesian Coordinates

Co	-0.5713223	0.0807292	-0.0526355
O	-1.4981961	-1.4709454	2.2220308
N	0.7299047	1.5016531	0.2704403
C	2.0361295	1.1525149	0.3518301
C	-0.6333105	0.4239608	-2.1204506
C	-0.2846813	-0.9302022	-1.8991507
C	0.9141254	-1.6350054	-2.4400172
C	0.1701106	1.4303080	-2.8751474
C	-1.8938224	0.6742598	-1.4500503
C	-2.3629382	-0.5844271	-0.9081567
C	-3.6579279	-0.7959580	-0.2011490
C	-2.6595403	1.9543935	-1.4192650
C	-1.3631456	-1.5555370	-1.1413084
C	-1.4221503	-2.9793469	-0.6963058
C	-1.8148284	-0.2912034	2.3644032
C	-2.6506992	0.1935271	3.5278754
H	-2.2933836	1.1666760	3.8926102
H	-2.6398868	-0.5516312	4.3330591
H	-3.6900815	0.3296883	3.1862005
H	-1.6636368	-3.0315425	0.3752166
H	-0.4716875	-3.4982592	-0.8712054
H	-2.2087363	-3.5140289	-1.2543516
H	1.7355110	-0.9408054	-2.6570900
H	0.6477938	-2.1422993	-3.3831271
H	1.2882470	-2.3995410	-1.7485761
H	0.0038925	2.4507997	-2.5050819
H	-0.1207195	1.4134968	-3.9391674
H	1.2460353	1.2155989	-2.8222616
H	-3.0714717	2.1403273	-0.4164676
H	-3.5081723	1.9049098	-2.1226279
H	-2.0406913	2.8131070	-1.7110186
H	-3.9653541	0.1048613	0.3458797
H	-3.5948613	-1.6299476	0.5085733
H	-4.4462149	-1.0272723	-0.9378912
C	0.3603601	2.7865590	0.3694354
C	1.2946945	3.8112595	0.4855184
C	2.6525144	3.4789980	0.5109179
C	3.0265266	2.1372248	0.4551770
C	2.3099725	-0.2969145	0.3898340
H	3.4126772	4.2583397	0.5993266
H	4.0741510	1.8436468	0.5288245
H	-0.7123758	2.9812803	0.3603406
H	0.9598953	4.8469749	0.5598170
C	3.4838796	-0.8685848	-0.1280785
C	1.3426367	-1.1401193	0.9724064
C	1.5351335	-2.5211694	1.0350121

C	3.6709084	-2.2471611	-0.0687456
C	2.7036404	-3.0890571	0.5118449
H	0.4863981	-0.7415556	1.5364395
H	4.2405443	-0.2390039	-0.6012366
H	4.5697282	-2.7134377	-0.4775376
H	0.7664405	-3.1244687	1.5181986
O	-1.4775926	0.6474990	1.5162408
C	2.9807636	-4.5757168	0.5321131
C	1.9844815	-5.4814533	1.2179454
H	1.8696305	-5.2048672	2.2787175
H	2.3350168	-6.5182751	1.1452995
H	0.9891677	-5.3987805	0.7509277
O	3.9836273	-5.0009557	0.0004952

Transition State TS2d

SCF energy:	–2633.156535 hartree
Zero-point correction:	+0.479184 hartree
Enthalpy correction:	+0.507244 hartree
Free energy correction:	+0.413895 hartree
Quasiharmonic free energy correction:	+0.431043 hartree
Imaginary Frequency	939.0 *i*cm^{-1}

Cartesian Coordinates

Co	-0.7962695	-0.4804023	-0.0391751
O	-0.1054756	0.0510598	2.8587435
N	-1.4138388	1.3610325	0.0904106
C	-0.4418208	2.3036919	0.0044070
C	-1.4705872	-0.6280519	-2.0419273
C	-0.0713624	-0.9045296	-1.9639425
C	1.0049777	-0.2115311	-2.7343908
C	-2.0931744	0.4576125	-2.8560719
C	-2.1577403	-1.5503041	-1.1853324
C	-1.1757958	-2.4698488	-0.6553480
C	-1.4867238	-3.6164696	0.2456968
C	-3.6274026	-1.6462812	-0.9330490
C	0.1010389	-2.0777309	-1.1269286
C	1.3788841	-2.8218563	-0.9095803
C	-1.1967022	-0.5633891	2.7524451
C	-1.9479850	-0.9537357	3.9970012
H	-3.0057557	-1.1412981	3.7746528
H	-1.8368732	-0.1747133	4.7630272
H	-1.5000307	-1.8789001	4.3959207
H	1.4857278	-3.1689276	0.1280426
H	2.2541862	-2.2137731	-1.1660446
H	1.3934146	-3.7162547	-1.5554734
H	0.7408013	0.8318799	-2.9515216
H	1.1683198	-0.7207759	-3.6994533
H	1.9588016	-0.2092766	-2.1913893
H	-3.0762554	0.7550999	-2.4686521
H	-2.2388646	0.1024047	-3.8903127
H	-1.4551978	1.3510039	-2.9031761
H	-3.8331348	-1.7499005	0.1428814
H	-4.0479166	-2.5327209	-1.4367162
H	-4.1694675	-0.7687623	-1.3107361
H	-2.1669138	-3.3061404	1.0512589
H	-0.5804090	-4.0393101	0.6989446
H	-1.9844043	-4.4177999	-0.3262171
C	-2.7012851	1.7135267	0.2062649
C	-3.0987901	3.0470695	0.1914382
C	-2.1185622	4.0358983	0.0548581
C	-0.7788157	3.6627165	-0.0326761
C	0.9192461	1.7536895	0.0275626
H	-2.3968513	5.0919259	0.0359991
H	0.0077087	4.4149780	-0.0978462
H	-3.4182445	0.8986395	0.3143087
H	-4.1552696	3.3014015	0.2881598

C	2.0562772	2.4566882	-0.3919111
C	1.0236084	0.4264602	0.5249407
C	2.3027171	-0.1411712	0.6277250
C	3.3075690	1.8506957	-0.3110955
C	3.4470260	0.5520208	0.2116924
H	0.3622199	0.1672835	1.6240884
H	1.9713446	3.4674012	-0.7973210
H	4.2112103	2.3682250	-0.6392845
H	2.4024435	-1.1299025	1.0763078
O	-1.6912212	-0.9005156	1.6269740
C	4.8407629	-0.0262363	0.2968267
O	5.7775289	0.6030204	-0.1458393
C	5.0197449	-1.3849401	0.9374171
H	4.6524279	-1.3799039	1.9765620
H	6.0852027	-1.6454813	0.9270750
H	4.4517314	-2.1564805	0.3919493

Co-Complex 7d

SCF energy:	–2633.169053 hartree
Zero-point correction:	+0.484358 hartree
Enthalpy correction:	+0.513159 hartree
Free energy correction:	+0.417368 hartree
Quasiharmonic free energy correction:	+0.437160 hartree

Cartesian Coordinates

Co	-0.3606297	-0.2626175	-0.0170943
C	-2.4020774	-0.7992212	-0.4027912
C	-1.9987507	0.3192412	-1.2104881
C	-2.7370854	1.6154282	-1.3254410
C	-3.5191766	-0.7934223	0.5895542
C	-1.5689241	-1.9138679	-0.6964372
C	-0.6228543	-1.4840356	-1.6947658
C	0.4283570	-2.3267034	-2.3425343
C	-1.7390032	-3.2956505	-0.1429867
C	-0.8992920	-0.1170155	-2.0281791
C	-0.2252601	0.6644214	-3.1093843
H	0.8439368	0.4209519	-3.1778100
H	-0.3100014	1.7455857	-2.9438315
H	-0.6864428	0.4303946	-4.0841582
H	-2.9975620	2.0268958	-0.3416781
H	-3.6761458	1.4629002	-1.8840957
H	-2.1566131	2.3741337	-1.8645870
H	-3.3015461	-1.4400813	1.4519176
H	-4.4408301	-1.1760037	0.1184230
H	-3.7331321	0.2201369	0.9534307
H	-0.8813425	-3.9410148	-0.3761556
H	-2.6297124	-3.7790005	-0.5787291
H	-1.8793621	-3.2904604	0.9493194
H	0.7316859	-3.1714012	-1.7096701
H	1.3266072	-1.7396772	-2.5799702
H	0.0454644	-2.7420815	-3.2901330
C	0.7541379	1.3080859	-0.1700792
C	2.1351066	1.0271589	-0.0415409
C	0.3719125	2.6298832	-0.3726686
C	3.0962043	2.0419257	-0.1830797
C	1.3217528	3.6591494	-0.5002911
H	-0.6735799	2.9244709	-0.4353073
C	2.6908016	3.3530424	-0.4244279
H	4.1617490	1.8189024	-0.0939128
H	3.4445456	4.1337908	-0.5345585
N	1.3339570	-1.1319026	0.4739602
C	2.4407492	-0.3573730	0.3233007
C	1.4482269	-2.3895720	0.9212129
C	3.7139533	-0.8892920	0.5730779
C	2.6812374	-2.9712106	1.1997350
H	0.5203961	-2.9435459	1.0585453

C	3.8363061	-2.2066385	1.0057651
H	4.5972193	-0.2649748	0.4382289
H	2.7291959	-3.9999565	1.5594010
H	4.8229802	-2.6318243	1.2029216
O	-0.6610987	0.0516502	1.9250834
C	-1.1300169	1.0087303	2.5264520
O	-1.7710809	1.9525274	1.8521727
C	-0.9890361	1.1635002	4.0115655
H	-0.5844846	0.2418879	4.4450395
H	-1.9564297	1.4064317	4.4784563
H	-0.2913404	1.9927509	4.2197424
H	-2.0501071	2.6895164	2.4185682
C	0.7979608	5.0566313	-0.7072814
O	-0.4008674	5.2625395	-0.6879974
C	1.7883236	6.1769477	-0.9344411
H	2.4618383	6.2852428	-0.0685358
H	2.4199673	5.9697041	-1.8134672
H	1.2401225	7.1145651	-1.0886139

Co-Complex 8d

SCF energy:	–2404.033677 hartree
Zero-point correction:	+0.420102 hartree
Enthalpy correction:	+0.443965 hartree
Free energy correction:	+0.359406 hartree
Quasiharmonic free energy correction:	+0.376787 hartree

Cartesian Coordinates

Co	0.4229257	0.0489820	-0.3046536
C	2.3920484	-0.1532472	-1.0931135
C	2.1736552	1.1515213	-0.4729415
C	2.3532801	2.4572989	-1.1792649
C	2.7377599	-0.3395281	-2.5329437
C	2.2048875	-1.1564404	-0.1217584
C	1.7740345	-0.4967769	1.0919292
C	1.4282725	-1.1661658	2.3794061
C	2.4545072	-2.6228120	-0.2771239
C	1.8630295	0.9377033	0.8935988
C	1.7066881	1.9537363	1.9766256
H	0.8438792	1.7365531	2.6205341
H	1.5912861	2.9717917	1.5867850
H	2.6096647	1.9378378	2.6107734
H	1.7627427	2.5031342	-2.1070155
H	3.4114242	2.5929954	-1.4594491
H	2.0635440	3.3103164	-0.5527787
H	2.6250509	-1.3824513	-2.8575750
H	3.7862000	-0.0427577	-2.7098788
H	2.1161182	0.2973105	-3.1813716
H	1.8161846	-3.2260416	0.3827324
H	3.4994704	-2.8485014	-0.0042328
H	2.3138878	-2.9651981	-1.3126366
H	0.9944260	-2.1627357	2.2183328
H	0.7156831	-0.5685851	2.9641748
H	2.3370537	-1.2942159	2.9938708
C	-0.9835455	1.2664428	0.1303704
C	-2.2489430	0.6425545	0.2735150
C	-0.9162635	2.6497435	0.2617436
C	-3.3906253	1.3921707	0.5810916
C	-2.0550571	3.4183940	0.5634948
H	0.0117502	3.2032524	0.1349855
C	-3.2908088	2.7783643	0.7330373
H	-4.3651129	0.9122713	0.6941316
H	-4.1859043	3.3522105	0.9775582
N	-1.0243093	-1.2790755	-0.3663760
C	-2.2463781	-0.8000242	0.0076532
C	-0.8940856	-2.5659044	-0.7260867
C	-3.3532413	-1.6526555	0.0642679

C	-1.9599760	-3.4606399	-0.7030907
H	0.0961532	-2.8863445	-1.0440695
C	-3.2097451	-2.9941624	-0.2875762
H	-4.3220092	-1.2617726	0.3750190
H	-1.8061568	-4.4973956	-1.0049938
H	-4.0685440	-3.6679946	-0.2469586
C	-1.8569674	4.9081425	0.7054270
O	-0.7400103	5.3747976	0.5969196
C	-3.0611359	5.7769212	0.9834237
H	-3.8182964	5.6626077	0.1906476
H	-2.7423906	6.8250754	1.0385814
H	-3.5380815	5.4896870	1.9349388

Co-Complex 9d

SCF energy:	–3602.859462 hartree
Zero-point correction:	+0.656667 hartree
Enthalpy correction:	+0.696698 hartree
Free energy correction:	+0.570916 hartree
Quasiharmonic free energy correction:	+0.611693 hartree

Cartesian Coordinates

Co	2.3827325	0.2145140	-0.5517576
C	2.6612692	2.0502966	-1.4910939
C	3.3293746	2.0778894	-0.2288140
C	3.1355595	3.0688801	0.8715160
C	1.5898465	2.9809266	-1.9653210
C	3.3105779	1.0435244	-2.3191523
C	4.3053621	0.4148383	-1.5467118
C	5.2841250	-0.6170796	-2.0077320
C	2.9496140	0.7579257	-3.7406105
C	4.2695010	0.9971237	-0.2150645
C	5.1693604	0.6332259	0.9226495
H	5.4280846	-0.4350246	0.9059818
H	4.6951806	0.8489073	1.8904309
H	6.1127546	1.2037362	0.8780709
H	2.1589423	3.5649541	0.8175606
H	3.9116929	3.8493175	0.7927918
H	3.2259348	2.6049163	1.8627042
H	0.8121134	2.4463837	-2.5300546
H	2.0113196	3.7509840	-2.6333337
H	1.1010726	3.5009998	-1.1306219
H	3.3341213	-0.2152135	-4.0749806
H	3.3709699	1.5314841	-4.4048574
H	1.8597272	0.7602879	-3.8842283
H	4.8955955	-1.2168694	-2.8433948
H	5.5812734	-1.2977271	-1.1982942
H	6.2038434	-0.1223088	-2.3645143
C	1.3502809	0.4972016	1.0405791
C	1.5352678	-0.5065554	2.0177041
C	0.4075005	1.4906023	1.2829942
C	0.9003543	-0.4148223	3.2686262
C	-0.2916416	1.5528361	2.5006975
H	0.1676289	2.2415567	0.5291603
C	0.0086754	0.6271452	3.5162598
H	1.0733546	-1.1685313	4.0402847
H	-0.4870838	0.6858449	4.4863114
N	2.6846695	-1.5540048	0.2460954
C	2.3021257	-1.6633494	1.5468630
C	3.2305475	-2.6018391	-0.3834843
C	2.5559445	-2.8398517	2.2626029
C	3.4908155	-3.8054464	0.2659224
H	3.4569703	-2.4694950	-1.4410329
C	3.1646172	-3.9157649	1.6205783
H	2.2582294	-2.9133413	3.3087756
H	3.9350739	-4.6353326	-0.2851617
H	3.3643124	-4.8408447	2.1658300

O	-3.1080368	-2.9172454	-1.9379678
H	-4.9325544	-1.6805578	-0.6339286
H	-3.5008720	-0.7137400	-3.6210705
H	-5.2693732	1.0499953	-3.8465268
H	-6.1302433	-0.6026936	1.2681298
C	-4.4052848	-1.4261873	0.2859236
C	-5.0638899	-0.8244353	1.3546521
C	-3.5320035	0.1429807	-2.9465484
C	-4.5171259	1.1276427	-3.0583858
S	-2.1728939	-2.3475430	-0.9839523
C	-3.0391171	-1.6998068	0.4109448
O	-0.9250744	-2.9910865	-0.5901990
N	-1.6092672	-0.7935678	-1.7607365
C	-2.5799822	0.2588690	-1.9304611
C	-4.3808211	-0.4998179	2.5395855
H	-5.1073158	1.2815311	3.5031488
C	-4.5372882	2.2116390	-2.1730531
H	-6.1389904	-0.1392907	3.7525954
C	-5.0926834	0.1903265	3.6712207
H	-5.3088372	2.9787287	-2.2726436
C	-0.3373358	-0.5212512	-1.5981713
C	-2.3255209	-1.3945593	1.5735829
C	-3.0122836	-0.8075005	2.6329337
C	-2.5981697	1.3296437	-1.0297438
N	0.7962661	-0.3077918	-1.4496806
C	-3.5762922	2.3151071	-1.1609768
H	-1.2593941	-1.6144887	1.6481185
H	-4.5950396	0.0087353	4.6351516
H	-2.4722637	-0.5842548	3.5539788
H	-1.8691378	1.3907305	-0.2215074
H	-3.5811816	3.1478335	-0.4547915
C	-1.3882033	2.5714313	2.6258011
O	-1.7934053	3.1501778	1.6328046
C	-1.9921891	2.8326894	3.9874928
H	-1.2160262	3.0502535	4.7376695
H	-2.5429680	1.9431562	4.3348384
H	-2.6901465	3.6761284	3.9149379

Transition State TS3d

SCF energy:	–3602.819394 hartree
Zero-point correction:	+0.655552 hartree
Enthalpy correction:	+0.694607 hartree
Free energy correction:	+0.573316 hartree
Quasiharmonic free energy correction:	+0.605284 hartree
Imaginary Frequency	357.6 *i*cm^{-1}

Cartesian Coordinates

Co	0.3378058	1.7107002	-0.3417251
C	-0.6975605	3.0074641	-1.6027834
C	-0.8970974	3.4503912	-0.2452994
C	-2.2058856	3.6268233	0.4562092
C	-1.7451453	2.6411441	-2.6069050
C	0.7091784	3.1036841	-1.9013110
C	1.3839272	3.4974629	-0.7193876
C	2.8416717	3.8066751	-0.5923114
C	1.3171487	2.7821960	-3.2253216
C	0.3803806	3.6917705	0.3229751
C	0.6510093	4.1454897	1.7215834
H	1.6112992	3.7621076	2.0954523
H	-0.1378970	3.8128301	2.4109312
H	0.6938101	5.2468992	1.7729238
H	-3.0320731	3.1481587	-0.0836081
H	-2.4392403	4.7016287	0.5391699
H	-2.1824588	3.2138879	1.4750936
H	-1.5033253	1.6881331	-3.1019963
H	-1.8055735	3.4094077	-3.3957447

H	-2.7428352	2.5519037	-2.1575795
H	2.4115882	2.7151097	-3.1674377
H	1.0630538	3.5656193	-3.9588432
H	0.9394193	1.8229697	-3.6070672
H	3.4609158	3.1385397	-1.2089380
H	3.1884021	3.7452979	0.4481888
H	3.0379235	4.8373390	-0.9345599
C	-1.0904476	0.4977551	0.4232993
C	-0.7500485	0.2210480	1.7773650
C	-2.4359950	0.4908881	0.0485845
C	-1.7549418	-0.0484505	2.7090197
C	-3.4505007	0.2332843	0.9809737
H	-2.7343597	0.6783193	-0.9815195
C	-3.0970692	-0.0472039	2.3077804
H	-1.5101464	-0.2345865	3.7569507
H	-3.8689194	-0.2597872	3.0490817
N	1.4128613	0.9296176	1.1244609
C	0.6827071	0.3006861	2.0839749
C	2.7457731	0.9812738	1.2299864
C	1.3011253	-0.2318510	3.2215312
C	3.4224275	0.4707006	2.3348916
H	3.2820102	1.4393203	0.4007234
C	2.6840129	-0.1322306	3.3555770
H	0.7009752	-0.7323760	3.9818758
H	4.5094805	0.5388261	2.3813445
H	3.1832938	-0.5406340	4.2368405
O	0.3774966	-4.2276371	-0.8905174
H	2.0248562	-3.5877570	1.0419482
H	-2.3707557	-1.6983963	-2.4005110
H	-4.7025759	-2.5158774	-2.0076619
H	4.2566610	-2.9782445	1.9778406
C	2.7024696	-2.9334938	0.4911195
C	3.9539202	-2.5942163	1.0004927
C	-2.6449601	-2.1727954	-1.4559949
C	-3.9420841	-2.6361203	-1.2336119
S	0.7770657	-2.9436830	-1.4506210
C	2.3359020	-2.4432991	-0.7650623
O	0.7534451	-2.6712181	-2.8813096
N	-0.3572957	-1.7971610	-0.6557364
C	-1.6810085	-2.3085982	-0.4496981
C	4.8410273	-1.7838379	0.2703289
H	6.2493308	-1.5507802	1.9043484
C	-4.2614349	-3.2548134	-0.0203814
H	6.9594107	-2.1586003	0.3981551
C	6.2093765	-1.4607248	0.8085762
H	-5.2749069	-3.6258847	0.1499490
C	-0.0961039	-0.4739157	-0.8826580
C	3.1890648	-1.6331015	-1.5179955
C	4.4367954	-1.3072438	-0.9881502
C	-1.9874260	-2.9475117	0.7534148
N	0.6872586	0.2024199	-1.5316568
C	-3.2830818	-3.4187081	0.9673137
H	2.8717785	-1.2781187	-2.4980957
H	6.5283184	-0.4458158	0.5251158
H	5.1180966	-0.6818840	-1.5712363
H	-1.2092126	-3.0637494	1.5081408
H	-3.5309922	-3.9205423	1.9054519
C	-4.8702784	0.2774872	0.4792173
O	-5.0881703	0.6809835	-0.6463156
C	-5.9778951	-0.1814853	1.3960338
H	-5.7901564	-1.2089023	1.7455761
H	-6.9317988	-0.1402551	0.8560661
H	-6.0385569	0.4658089	2.2865853

Co-Complex 10d

SCF energy:	–3602.857162 hartree
Zero-point correction:	+0.657971 hartree
Enthalpy correction:	+0.697223 hartree
Free energy correction:	+0.573780 hartree
Quasiharmonic free energy correction:	+0.609731 hartree

Cartesian Coordinates

Co	-2.4168448	0.3195795	-0.2427456
C	-2.6096582	0.7634823	-2.2526147
C	-2.9822964	-0.5984863	-1.9599694
C	-2.3723815	-1.8217605	-2.5578972
C	-1.4836981	1.1961305	-3.1335930
C	-3.4492123	1.6096258	-1.4764956
C	-4.4142496	0.7773260	-0.7665340
C	-5.4899300	1.3006768	0.1305905
C	-3.3737297	3.0990508	-1.3905318
C	-4.1334676	-0.5718149	-1.0738940
C	-4.8487894	-1.7835929	-0.5694132
H	-5.4249205	-1.5720115	0.3414126
H	-4.1494897	-2.6017790	-0.3459835
H	-5.5556661	-2.1532299	-1.3309608
H	-1.3052430	-1.6811530	-2.7746056
H	-2.8787562	-2.0673898	-3.5078894
H	-2.4719578	-2.6921806	-1.8965158
H	-1.0056965	2.1081214	-2.7502444
H	-1.8517967	1.4069552	-4.1515802
H	-0.7138340	0.4161329	-3.2087896
H	-3.6222869	3.4579513	-0.3815556
H	-4.0911783	3.5591537	-2.0915885
H	-2.3698476	3.4667837	-1.6400929
H	-5.1211238	2.1066751	0.7828218
H	-5.9180458	0.5119835	0.7643512
H	-6.3138570	1.7245900	-0.4687912
C	0.5945454	-0.7928375	-0.5688091
C	-0.0640283	-1.9345480	-0.0667696
C	1.4955670	-0.9101122	-1.6314036
C	0.2501493	-3.1865367	-0.6261266
C	1.7864576	-2.1569446	-2.1943821
H	2.0059468	-0.0099413	-1.9735119
C	1.1617104	-3.2988922	-1.6713471
H	-0.2383525	-4.0832930	-0.2382894
H	1.4163416	-4.2706717	-2.0980789
N	-2.0509076	-1.0409879	1.1179130
C	-0.9900551	-1.8863962	1.0962339
C	-2.8790835	-1.0379554	2.1815796
C	-0.7600515	-2.7510774	2.1775036
C	-2.7012339	-1.8698160	3.2785947
C	-1.6122644	-2.7443781	3.2763026
H	0.1167368	-3.3972673	2.1565586
H	-3.3977554	-1.8214281	4.1168458
H	-1.4228415	-3.4035554	4.1261820
O	3.1881860	1.4247483	2.3893102
H	4.5728633	1.3493040	-0.1009040
H	2.3268101	3.0937625	-1.1754256
H	2.1047812	5.5773816	-0.9221974
H	6.1457144	0.1361765	-1.6069553
C	4.5570573	0.2591551	-0.1597465
C	5.4340268	-0.4275772	-0.9984800
C	1.8456236	3.5259555	-0.2952344
C	1.7186087	4.9096285	-0.1486138
S	2.4917247	0.3870524	1.6416144
C	3.6507625	-0.4762660	0.6084029
O	1.6306498	-0.6031697	2.3010399
N	1.4708403	1.2563608	0.4964010
C	1.3518646	2.6751441	0.6973234
C	5.4251252	-1.8329462	-1.0729032

H	7.0291514	-1.8950853	-2.5297020
C	1.1080799	5.4359859	0.9936461
H	6.9314786	-3.3277931	-1.4737729
C	6.3360512	-2.5739985	-2.0133041
H	1.0164322	6.5180525	1.1134045
C	0.3405305	0.5477644	0.0353028
C	3.6371440	-1.8715447	0.5845678
C	4.5232426	-2.5387795	-0.2577955
C	0.7408425	3.1974741	1.8419434
N	-0.8329050	1.0149094	0.0691357
C	0.6231230	4.5797990	1.9886388
H	2.9212456	-2.4146518	1.2015093
H	5.7445254	-3.1122807	-2.7730414
H	4.5051832	-3.6304931	-0.3004412
H	0.3746012	2.5176802	2.6128646
H	0.1555977	4.9925723	2.8855496
H	-3.7108472	-0.3320370	2.1413143
C	2.8201014	-2.3338356	-3.2750571
O	3.2492169	-3.4435962	-3.5195616
C	3.3098224	-1.1075291	-4.0103542
H	3.8767241	-0.4592226	-3.3232815
H	2.4691793	-0.5163432	-4.4067937
H	3.9659973	-1.4199679	-4.8320792

Co-Complex 11d

SCF energy:	–3831.975810 hartree
Zero-point correction:	+0.722054 hartree
Enthalpy correction:	+0.766128 hartree
Free energy correction:	+0.632725 hartree
Quasiharmonic free energy correction:	+0.668806 hartree

Cartesian Coordinates

Co	-2.1338811	-0.1270404	0.2218520
C	-2.2251625	-0.0890102	2.2673991
C	-3.5842613	0.0566691	1.7681386
C	-4.4713169	1.2298492	2.0355141
C	-1.5762127	0.8177645	3.2585098
C	-1.7328577	-1.3740465	1.8501448
C	-2.7222680	-1.9618858	1.0155741
C	-2.6480070	-3.2790977	0.3176443
C	-0.4047558	-1.9549888	2.2004914
C	-3.8895011	-1.0884097	1.0099173
C	-5.1580943	-1.3927290	0.2871340
H	-4.9510318	-1.7743296	-0.7212774
H	-5.8055106	-0.5097147	0.1991303
H	-5.7214374	-2.1671635	0.8353457
H	-3.9042187	2.1716239	2.0464428
H	-4.9565529	1.1274225	3.0208525
H	-5.2669070	1.3208349	1.2833942
H	-0.4823588	0.7480821	3.2279125
H	-1.9018740	0.5360168	4.2753800
H	-1.8584526	1.8666389	3.1036857
H	-0.0099762	-2.5710149	1.3858856
H	-0.4994263	-2.5939008	3.0946789
H	0.3328664	-1.1741853	2.4247194
H	-1.6381337	-3.7018491	0.3749764
H	-2.9119426	-3.1776276	-0.7438543
H	-3.3485630	-3.9948257	0.7799298
C	0.8259054	1.2240531	0.6838649
C	0.0016241	2.3515745	0.8772614
C	1.9128418	1.0119907	1.5333847
C	0.3250588	3.2492142	1.9116054
C	2.2049774	1.8822750	2.5880689
H	2.5646621	0.1517363	1.3830418
C	1.4033209	3.0197326	2.7640499
H	-0.3001107	4.1306363	2.0684407

H	1.6083637	3.7297924	3.5664305
N	-2.1277711	1.7928162	-0.2709666
C	-1.1503178	2.6925923	0.0025005
C	-3.2005704	2.1832836	-0.9892908
C	-1.2350581	4.0024079	-0.5016062
C	-3.3504482	3.4704274	-1.4910077
H	-3.9410437	1.4104502	-1.1895020
C	-2.3360363	4.3988950	-1.2540933
H	-0.4153627	4.6936028	-0.3055347
H	-4.2415139	3.7258481	-2.0665006
H	-2.4001588	5.4149074	-1.6501310
O	0.8944681	-1.6662376	-3.0760698
H	3.4927758	-1.8238598	0.3141213
H	0.6928456	2.0522957	-2.5118181
H	1.8672575	4.1323244	-3.2436253
H	3.4579727	-3.6111075	2.0295593
C	2.7685446	-2.6369696	0.2435800
C	2.7503575	-3.6493905	1.1976040
C	1.7610615	2.1805372	-2.3256694
C	2.4214779	3.3416937	-2.7321319
S	1.9703681	-1.4471595	-2.0811376
C	1.8498939	-2.6832686	-0.8116002
O	3.3359894	-1.3836259	-2.5844720
N	1.7603063	0.0096750	-1.1954939
C	2.4708178	1.1678721	-1.6679054
C	1.8470156	-4.7252265	1.0997413
H	2.7601863	-6.4590353	1.9970323
C	3.7927789	3.4819331	-2.4986885
H	1.8993294	-5.4224765	3.1468853
C	1.8640604	-5.8280737	2.1235128
H	4.3132950	4.3854131	-2.8244661
C	0.5812251	0.2344006	-0.4094113
C	0.9414971	-3.7323240	-0.9383046
C	0.9511873	-4.7489400	0.0196605
C	3.8416500	1.3070665	-1.4292188
N	-0.4910781	-0.3940314	-0.6013049
C	4.5002123	2.4618619	-1.8531137
H	0.2335178	-3.7535708	-1.7653609
H	0.9823055	-6.4787271	2.0366402
H	0.2468262	-5.5788483	-0.0762808
H	4.3832596	0.5086194	-0.9223373
H	5.5724535	2.5681048	-1.6740323
O	-3.0382629	-0.6130900	-1.7644109
C	-2.1535599	-0.8468276	-2.5927831
O	-1.6698827	-2.0600621	-2.7073147
C	-1.6256002	0.1977221	-3.5370358
H	-0.5294907	0.1415768	-3.5826052
H	-1.9543022	1.1967668	-3.2310867
H	-2.0090046	-0.0136563	-4.5491837
H	-0.7393170	-2.0182544	-3.0560336
C	3.3849403	1.5386031	3.4644086
O	3.9967811	0.5084223	3.2657881
C	3.7668105	2.4968636	4.5681317
H	3.9984461	3.4932236	4.1574634
H	4.6444540	2.1051831	5.0968994
H	2.9355762	2.6229139	5.2812718

Co-Complex 12d

SCF energy:	–2725.425648 hartree
Zero-point correction:	+0.482598 hartree
Enthalpy correction:	+0.513243 hartree
Free energy correction:	+0.412767 hartree
Quasiharmonic free energy correction:	+0.435525 hartree

Cartesian Coordinates

Co	-0.8135204	0.1842387	0.4072377
O	0.1098565	-0.2899898	2.1194270
O	-1.1556448	1.4391896	1.9136514
N	0.6323670	1.3949847	-0.2381257
C	1.8678809	1.0752071	-0.6924677
C	-2.6166819	0.3869011	-0.5723009
C	-1.6409615	-0.0766826	-1.5029498
C	-1.2256958	0.6079093	-2.7626444
C	-3.4024217	1.6544751	-0.6400412
C	-2.7228582	-0.5940766	0.4934278
C	-1.8076140	-1.6468876	0.2059301
C	-1.5296455	-2.8496781	1.0422112
C	-3.6396422	-0.4930942	1.6670575
C	-1.1135118	-1.3253118	-1.0115923
C	-0.1832482	-2.2473857	-1.7276835
C	-0.3978242	0.7414511	2.6621920
C	-0.0916254	1.1335215	4.0705664
H	-0.9180498	1.7187407	4.4948291
H	0.8129994	1.7642302	4.0706985
H	0.1130819	0.2428951	4.6796450
H	0.5840958	-2.6596766	-1.0593167
H	0.3133770	-1.7527147	-2.5692504
H	-0.7562822	-3.0979003	-2.1351350
H	-1.3722101	1.6947252	-2.7055640
H	-1.8392388	0.2358370	-3.6006749
H	-0.1753842	0.4174294	-3.0183055
H	-3.4328116	2.1554869	0.3390528
H	-4.4443105	1.4378424	-0.9308776
H	-2.9911663	2.3539921	-1.3796348
H	-3.2925919	-1.1100070	2.5070791
H	-4.6479503	-0.8445578	1.3900246
H	-3.7281935	0.5443252	2.0165751
H	-1.8798517	-2.7228693	2.0742469
H	-0.4537748	-3.0724821	1.0696495
H	-2.0424347	-3.7273406	0.6130221
C	0.2364043	2.6860644	-0.2739118
C	1.0328853	3.7113698	-0.7667516
C	2.3086169	3.3973655	-1.2356021
C	2.7228176	2.0701535	-1.1912538
C	2.3611305	-0.3296983	-0.6760485
H	2.9722461	4.1719917	-1.6257950
H	3.7169014	1.7861727	-1.5368475
H	-0.7568956	2.8920162	0.1226300
H	0.6538166	4.7344693	-0.7728249
C	2.4861629	-1.0551168	0.5155564
C	2.8093354	-0.9270598	-1.8803366
C	3.3736996	-2.2127465	-1.8744551
C	3.0330590	-2.3362683	0.5115972
C	3.4867630	-2.9267263	-0.6781330
H	2.1479086	-0.6137706	1.4506041
H	3.1316876	-2.9089779	1.4359990
H	3.6986648	-2.6441816	-2.8214854
C	2.5977230	-0.2603682	-3.1330703
N	2.3609913	0.2707263	-4.1381956
C	4.0646347	-4.3240759	-0.6015754
O	4.0564696	-4.9085895	0.4600076
C	4.6306241	-4.9405018	-1.8590098
H	3.8530980	-5.0332537	-2.6352913
H	5.4367436	-4.3144227	-2.2750815
H	5.0251392	-5.9362390	-1.6223147

1-[4-(2-Pyridinyl)phenyl]-ethanone (1d)

SCF energy:	–631.949372 hartree
Zero-point correction:	+0.207159 hartree
Enthalpy correction:	+0.218720 hartree
Free energy correction:	+0.164738 hartree

Quasiharmonic free energy correction: +0.173557 hartree

Cartesian Coordinates

C	2.9426709	1.2591031	-0.1315035
C	1.5540125	1.2728383	-0.0707799
C	3.6488196	0.0453227	-0.1643950
C	2.9211975	-1.1558131	-0.1313744
C	1.5304019	-1.1425199	-0.0681766
C	0.8202014	0.0709595	-0.0398594
C	-0.6670994	0.0430259	0.0237072
C	-1.4473408	1.2122733	-0.0541061
N	-1.2412708	-1.1652956	0.1554831
C	-2.5663237	-1.2556773	0.2189712
C	-3.4185244	-0.1481797	0.1554806
C	-2.8358269	1.1120081	0.0137475
H	1.0419310	2.2355646	-0.0394035
H	3.5166951	2.1872488	-0.1530684
H	3.4390500	-2.1163480	-0.1541807
H	0.9591133	-2.0703766	-0.0386694
H	-3.4550640	2.0106550	-0.0460435
H	-0.9806130	2.1898481	-0.1727960
H	-2.9813361	-2.2651480	0.3267113
H	-4.5016403	-0.2749690	0.2130300
C	5.1467254	0.0967508	-0.2303329
O	5.7310463	1.1634447	-0.2476305
C	5.9121627	-1.2123030	-0.2745098
H	5.6174753	-1.8106881	-1.1518121
H	5.7016942	-1.8204299	0.6202472
H	6.9861024	-0.9927347	-0.3238663

5-Acetyl-2-(2-pyridinyl)-benzonitrile (3d)

SCF energy: –724.191114 hartree
Zero-point correction: +0.205568 hartree
Enthalpy correction: +0.218945 hartree
Free energy correction: +0.161569 hartree
Quasiharmonic free energy correction: +0.168351 hartree

Cartesian Coordinates

C	-2.5585193	-1.8175629	-0.2577013
C	-1.1742478	-1.7108522	-0.2002322
C	-3.3657163	-0.6723018	-0.1691639
C	-2.7498911	0.5741192	-0.0196474
C	-1.3501873	0.6910376	0.0369138
C	-0.5376224	-0.4661252	-0.0564681
C	0.9438473	-0.3765358	-0.0182695
C	1.7269984	-1.3905485	0.5625446
N	1.4911278	0.7168148	-0.5646988
C	2.8150352	0.8486239	-0.5602128
C	3.6772184	-0.1090271	-0.0146575
C	3.1144495	-1.2501642	0.5598260
H	-0.5630246	-2.6098868	-0.2991188
H	-3.0494825	-2.7845156	-0.3815688
H	-3.3432127	1.4848791	0.0641816
C	-0.8190708	2.0118353	0.2449744
H	3.7473100	-2.0189613	1.0100911
H	1.2574565	-2.2581086	1.0286504
H	3.2143186	1.7630203	-1.0137944
H	4.7584614	0.0429752	-0.0358187
N	-0.5257263	3.1148310	0.4573058
C	-4.8585111	-0.8440428	-0.2395752
O	-5.3399149	-1.9524354	-0.3643220
C	-5.7311992	0.3908463	-0.1514936
H	-5.4967030	1.0964440	-0.9650589
H	-5.5647575	0.9223799	0.7995510
H	-6.7831962	0.0874915	-0.2217171

11 Optimization with B3LYP-D3BJ/def2-SVP for 1e (R = CN)

Co-Complex 5e

SCF energy:	–2572.779137 hartree
Zero-point correction:	+0.445966 hartree
Enthalpy correction:	+0.473133 hartree
Free energy correction:	+0.381817 hartree
Quasiharmonic free energy correction:	+0.397910 hartree

Cartesian Coordinates

Co	-0.9089102	-0.0025492	0.0207120
O	-0.8439865	-0.5237961	1.9545981
O	-2.1142871	1.0387026	1.2042928
N	0.4759401	1.4500917	0.1557199
C	1.8247231	1.3586669	0.2934764
C	-1.0788779	0.0054642	-2.0502266
C	-0.2134764	-1.0893185	-1.6478337
C	1.1561249	-1.3359496	-2.1832506
C	-0.6888675	1.1088643	-2.9785286
C	-2.3601399	-0.2081742	-1.4639555
C	-2.2759555	-1.3787886	-0.6277050
C	-3.3779172	-1.9489210	0.2037306
C	-3.5598320	0.6700731	-1.5788791
C	-0.9605021	-1.9434631	-0.7893418
C	-0.4741623	-3.1689133	-0.0923813
C	-1.7005541	0.3795956	2.2126414
C	-2.1580412	0.6758539	3.6021899
H	-3.1528334	1.1400292	3.5897553
H	-1.4490495	1.3854149	4.0603847
H	-2.1587186	-0.2408204	4.2072786
H	-0.6551173	-3.0979551	0.9910873
H	0.5971466	-3.3365640	-0.2541099
H	-1.0220797	-4.0511729	-0.4625452
H	1.7054624	-0.4016126	-2.3513204
H	1.0731917	-1.8478399	-3.1577848
H	1.7543170	-1.9712941	-1.5202151
H	-1.3148977	2.0006431	-2.8413624
H	-0.8006108	0.7808172	-4.0258314
H	0.3600204	1.4032595	-2.8359610
H	-3.9604778	0.9196445	-0.5852982
H	-4.3519487	0.1444939	-2.1377826
H	-3.3373501	1.6049724	-2.1090938
H	-4.0687608	-1.1657820	0.5448308
H	-2.9824792	-2.4697148	1.0867227
H	-3.9631493	-2.6794070	-0.3801700
C	-0.0850283	2.6723751	0.0172384
C	0.6452250	3.8527203	0.0023380
C	2.0306367	3.7771225	0.1510141
C	2.6133302	2.5235606	0.2988940
C	2.5091929	0.0467218	0.4212686
H	2.6461039	4.6794917	0.1613088
H	3.6891867	2.4271759	0.4450413
H	-1.1704515	2.6920049	-0.0720991
H	0.1288867	4.8066530	-0.1145506
C	3.6840983	-0.1778940	-0.3222290
C	2.0542327	-0.9577828	1.2894133
C	2.7371168	-2.1677314	1.3889780
C	4.3661703	-1.3868402	-0.2362217
C	3.8899048	-2.3988187	0.6177912
H	1.1717957	-0.7927868	1.9031572

H	4.0518546	0.5901119	-1.0057294
H	5.2635796	-1.5591846	-0.8324995
H	2.3826764	-2.9412961	2.0724603
C	4.5722858	-3.6587542	0.7001834
N	5.1122193	-4.6841498	0.7597792

Transition State TS1e

SCF energy:	–2572.764071 hartree
Zero-point correction:	+0.444793 hartree
Enthalpy correction:	+0.471472 hartree
Free energy correction:	+0.381786 hartree
Quasiharmonic free energy correction:	+0.395774 hartree
Imaginary Frequency	70.4 *i*cm^{-1}

Cartesian Coordinates

Co	-0.8394106	-0.2713789	-0.0606224
O	-0.4386995	-1.9417105	2.0531654
N	-0.7571982	1.6340115	0.3867945
C	0.4325300	2.2737885	0.4882903
C	-1.0879128	0.0670468	-2.1076556
C	0.0941504	-0.6927229	-1.9194641
C	1.4519700	-0.3544713	-2.4386457
C	-1.1832416	1.3965407	-2.7779004
C	-2.1868853	-0.6587273	-1.5037945
C	-1.6714380	-1.9256914	-1.0273317
C	-2.4729784	-3.0069799	-0.3859983
C	-3.6189913	-0.2465403	-1.4543707
C	-0.2745772	-1.9332925	-1.2500718
C	0.6577049	-3.0236082	-0.8401206
C	-1.5297763	-1.3974859	2.2493509
C	-2.4194456	-1.7490722	3.4160028
H	-2.9472462	-0.8621141	3.7926065
H	-1.8270601	-2.2182000	4.2116286
H	-3.1803650	-2.4705986	3.0750439
H	0.5087204	-3.2741378	0.2203469
H	1.7071732	-2.7435438	-0.9919047
H	0.4576750	-3.9291167	-1.4369355
H	1.5871572	0.7277109	-2.5581665
H	1.5930342	-0.8196010	-3.4293459
H	2.2477636	-0.7278971	-1.7834813
H	-2.0104421	1.9996480	-2.3806692
H	-1.3673908	1.2524856	-3.8561550
H	-0.2539892	1.9729536	-2.6744647
H	-4.0516644	-0.4598225	-0.4659566
H	-4.1999655	-0.8129454	-2.2020729
H	-3.7498306	0.8212175	-1.6738127
H	-3.3198783	-2.5944591	0.1780103
H	-1.8587401	-3.6069049	0.2971959
H	-2.8797898	-3.6785494	-1.1611043
C	-1.9068012	2.3123595	0.5367931
C	-1.9312671	3.6890870	0.7312906
C	-0.7161085	4.3775572	0.7876484
C	0.4741915	3.6619012	0.6773172
C	1.6450366	1.4325794	0.4496787
H	-0.6960520	5.4586788	0.9419617
H	1.4376938	4.1630675	0.7744929
H	-2.8206693	1.7185534	0.5083544
H	-2.8858350	4.2052503	0.8439801
C	2.8586940	1.8921305	-0.0881020
C	1.5825481	0.1291021	0.9763536
C	2.6950152	-0.7071922	0.9544226
C	3.9772681	1.0632403	-0.1144732
C	3.9029906	-0.2456184	0.4028148
H	0.7007918	-0.2449181	1.5135012
H	2.9270580	2.8946486	-0.5149563
H	4.9151189	1.4183718	-0.5448274

H	2.6268814	-1.7083959	1.3821315
O	-1.9987360	-0.4946338	1.4347734
C	5.0483997	-1.1085301	0.3541591
N	5.9658692	-1.8167708	0.3000651

Co-Complex 6e

SCF energy:	–2572.764665 hartree
Zero-point correction:	+0.444926 hartree
Enthalpy correction:	+0.472348 hartree
Free energy correction:	+0.380705 hartree
Quasiharmonic free energy correction:	+0.396340 hartree

Cartesian Coordinates

Co	-0.5908182	0.0894515	-0.0562342
O	-1.4600050	-1.4073501	2.2240270
N	0.7363491	1.4876205	0.2667169
C	2.0417574	1.1390425	0.3596190
C	-0.6533970	0.4201608	-2.1224373
C	-0.3038959	-0.9329390	-1.8945626
C	0.8950949	-1.6376887	-2.4360376
C	0.1489128	1.4230828	-2.8822790
C	-1.9144509	0.6731307	-1.4534208
C	-2.3823780	-0.5828079	-0.9035212
C	-3.6743285	-0.7918914	-0.1901655
C	-2.6781326	1.9540835	-1.4248890
C	-1.3836773	-1.5554671	-1.1358653
C	-1.4407538	-2.9747167	-0.6777588
C	-1.8074540	-0.2345160	2.3593578
C	-2.6480212	0.2387679	3.5225353
H	-2.3297407	1.2343986	3.8614709
H	-2.5946715	-0.4878102	4.3428864
H	-3.6967665	0.3201281	3.1920460
H	-1.6653245	-3.0181901	0.3980534
H	-0.4987017	-3.5042917	-0.8648683
H	-2.2416080	-3.5084750	-1.2160123
H	1.7284271	-0.9476279	-2.6194401
H	0.6387119	-2.1135736	-3.3980444
H	1.2461418	-2.4281796	-1.7620477
H	-0.0156450	2.4451546	-2.5158473
H	-0.1454799	1.4023273	-3.9452995
H	1.2247369	1.2072612	-2.8322949
H	-3.0851147	2.1442398	-0.4209035
H	-3.5300615	1.9011152	-2.1240273
H	-2.0601316	2.8109416	-1.7237710
H	-3.9834240	0.1131252	0.3488969
H	-3.6054747	-1.6188928	0.5273837
H	-4.4644815	-1.0345733	-0.9211560
C	0.3672904	2.7734447	0.3641594
C	1.2999840	3.7980574	0.4919751
C	2.6570442	3.4657683	0.5339416
C	3.0304468	2.1238719	0.4791822
C	2.3252825	-0.3093407	0.3909116
H	3.4165811	4.2442172	0.6344743
H	4.0772089	1.8311777	0.5667057
H	-0.7051120	2.9688053	0.3461253
H	0.9639428	4.8335305	0.5640300
C	3.5125853	-0.8597361	-0.1179272
C	1.3629479	-1.1702047	0.9551449
C	1.5687693	-2.5461364	1.0061286
C	3.7268194	-2.2354295	-0.0738890
C	2.7557516	-3.0904067	0.4859921
H	0.4821198	-0.7973145	1.5015211
H	4.2662872	-0.2160683	-0.5756301
H	4.6455311	-2.6611825	-0.4810265
H	0.8156690	-3.1846003	1.4692082
O	-1.4970480	0.6988349	1.4961729

C	2.9693432	-4.5090240	0.5119452
N	3.1282215	-5.6583349	0.5174152

Transition State TS2e

SCF energy:	–2572.750260 hartree
Zero-point correction:	+0.440754 hartree
Enthalpy correction:	+0.467401 hartree
Free energy correction:	+0.378039 hartree
Quasiharmonic free energy correction:	+0.392863 hartree
Imaginary Frequency	858.9 $i\text{cm}^{-1}$

Cartesian Coordinates

Co	-0.5920541	-0.4751555	-0.0156783
O	-0.0389513	0.1986471	2.8744110
N	-1.1207539	1.4013475	-0.0230932
C	-0.1021395	2.2939566	-0.1020844
C	-1.1624343	-0.7105917	-2.0391678
C	0.2146530	-1.0471186	-1.8705401
C	1.3607447	-0.4529109	-2.6223432
C	-1.6892470	0.3503886	-2.9474087
C	-1.9371816	-1.5470433	-1.1678697
C	-1.0302049	-2.4796783	-0.5353586
C	-1.4424939	-3.5565430	0.4096056
C	-3.4204358	-1.5595694	-0.9908783
C	0.2865263	-2.1759600	-0.9597957
C	1.5127209	-2.9632453	-0.6285643
C	-1.1495904	-0.3714278	2.7442615
C	-1.9857766	-0.6535972	3.9635675
H	-3.0380751	-0.8046252	3.6927598
H	-1.8770581	0.1623615	4.6904410
H	-1.6059828	-1.5738591	4.4375505
H	1.5463209	-3.2539436	0.4310549
H	2.4329231	-2.4183789	-0.8695488
H	1.5147751	-3.8946475	-1.2201110
H	1.1678534	0.5926642	-2.8968102
H	1.5327416	-1.0152971	-3.5558459
H	2.2904777	-0.4833425	-2.0400967
H	-2.6727730	0.7191280	-2.6284947
H	-1.8051062	-0.0630420	-3.9636762
H	-1.0043086	1.2065940	-3.0179530
H	-3.6858072	-1.5944232	0.0762665
H	-3.8567634	-2.4521909	-1.4695923
H	-3.8982202	-0.6802237	-1.4434994
H	-2.1554505	-3.1721475	1.1523081
H	-0.5834539	-3.9869406	0.9408798
H	-1.9389948	-4.3703219	-0.1455729
C	-2.3938520	1.8180474	0.0038866
C	-2.7252452	3.1658540	-0.0996748
C	-1.6931968	4.1009184	-0.2299091
C	-0.3700854	3.6629295	-0.2240243
C	1.2293045	1.6858251	0.0213296
H	-1.9188646	5.1661180	-0.3158312
H	0.4536313	4.3750679	-0.2807868
H	-3.1541829	1.0438224	0.1139243
H	-3.7719681	3.4724139	-0.0746378
C	2.4149107	2.3101978	-0.3852557
C	1.2483282	0.3876810	0.5995593
C	2.4859912	-0.2357344	0.7893857
C	3.6356138	1.6584519	-0.2195689
C	3.6792723	0.3820778	0.3756796
H	0.5065636	0.2088603	1.6477974
H	2.3964810	3.2977027	-0.8508957
H	4.5632087	2.1332531	-0.5438996
H	2.5401254	-1.2006140	1.2946840
O	-1.5998796	-0.7545769	1.6125347
C	4.9319882	-0.2948856	0.5539283

N	5.9340763	-0.8647832	0.6869813

Co-Complex 7e

SCF energy:	–2572.765182 hartree
Zero-point correction:	+0.445885 hartree
Enthalpy correction:	+0.473228 hartree
Free energy correction:	+0.381775 hartree
Quasiharmonic free energy correction:	+0.398406 hartree

Cartesian Coordinates

Co	-0.4211274	-0.2317081	-0.0693327
C	-2.4601841	-0.7917695	-0.4256306
C	-2.0639251	0.2949220	-1.2776028
C	-2.8112941	1.5816713	-1.4365364
C	-3.5745733	-0.7499977	0.5685022
C	-1.6230227	-1.9135994	-0.6780529
C	-0.6831201	-1.5208965	-1.6972140
C	0.3679915	-2.3850814	-2.3154517
C	-1.7879732	-3.2731006	-0.0706462
C	-0.9652444	-0.1688096	-2.0825938
C	-0.2978714	0.5692671	-3.1977874
H	0.7729281	0.3307891	-3.2569813
H	-0.3900723	1.6564057	-3.0832913
H	-0.7578603	0.2877783	-4.1604248
H	-3.0147052	2.0600904	-0.4689664
H	-3.7817020	1.3945519	-1.9263124
H	-2.2652449	2.2966563	-2.0645438
H	-3.3552123	-1.3650816	1.4530877
H	-4.4968649	-1.1497806	0.1131284
H	-3.7886501	0.2761915	0.8953693
H	-0.9338552	-3.9282620	-0.2884460
H	-2.6838253	-3.7703947	-0.4790969
H	-1.9176223	-3.2263805	1.0219523
H	0.6781765	-3.2018870	-1.6503276
H	1.2622179	-1.8049503	-2.5828296
H	-0.0193536	-2.8404369	-3.2426664
C	0.6913594	1.3377238	-0.2788450
C	2.0707689	1.0668309	-0.1197212
C	0.3080157	2.6501488	-0.5454151
C	3.0358826	2.0752836	-0.2823397
C	1.2707773	3.6700523	-0.6927279
H	-0.7416845	2.9210597	-0.6406319
C	2.6422101	3.3760162	-0.5769425
H	4.0993466	1.8554936	-0.1679880
H	3.3819646	4.1676275	-0.7039266
N	1.2740745	-1.0789739	0.4582337
C	2.3779349	-0.3034479	0.2962681
C	1.3902283	-2.3205257	0.9486376
C	3.6509853	-0.8180591	0.5795317
C	2.6230816	-2.8846758	1.2613443
H	0.4645721	-2.8757158	1.0938289
C	3.7759368	-2.1197335	1.0568495
H	4.5326526	-0.1935927	0.4356700
H	2.6725165	-3.9006956	1.6553417
H	4.7626107	-2.5316345	1.2802601
O	-0.7258550	0.1345132	1.8627829
C	-1.1976244	1.0954254	2.4550752
O	-1.8207576	2.0447984	1.7691624
C	-1.0824694	1.2509379	3.9421832
H	-0.6763358	0.3332708	4.3823618
H	-2.0609701	1.4822445	4.3919663
H	-0.3986927	2.0881526	4.1641514
H	-2.1169882	2.7732584	2.3382702
C	0.8319657	5.0106346	-0.9566870
N	0.4484439	6.0873650	-1.1606989

Co-Complex 8e

SCF energy:	–2343.628624 hartree
Zero-point correction:	+0.381725 hartree
Enthalpy correction:	+0.404064 hartree
Free energy correction:	+0.324571 hartree
Quasiharmonic free energy correction:	+0.337025 hartree

Cartesian Coordinates

Co	0.4398366	0.0787900	-0.3378173
C	2.4188870	-0.2312571	-1.0739549
C	2.2295323	1.1123885	-0.5245687
C	2.4494145	2.3723752	-1.2973554
C	2.7938633	-0.5035625	-2.4929030
C	2.1757846	-1.1706951	-0.0553452
C	1.7394492	-0.4327175	1.1136899
C	1.3485177	-1.0236789	2.4258850
C	2.3674332	-2.6518623	-0.1149964
C	1.8924371	0.9832843	0.8450074
C	1.7735423	2.0569639	1.8755142
H	0.8812222	1.9324016	2.5034612
H	1.7476214	3.0606569	1.4351042
H	2.6557896	2.0100544	2.5364476
H	1.9228444	2.3536699	-2.2637078
H	3.5231890	2.5016099	-1.5153702
H	2.1140777	3.2593423	-0.7448450
H	2.6747699	-1.5620857	-2.7593291
H	3.8493589	-0.2299985	-2.6640844
H	2.1939025	0.1002390	-3.1915410
H	1.6253097	-3.1935153	0.4876388
H	3.3598920	-2.9061261	0.2948623
H	2.3372883	-3.0383495	-1.1434308
H	0.8652547	-2.0030357	2.3044370
H	0.6637829	-0.3643105	2.9763381
H	2.2450489	-1.1717253	3.0539263
C	-0.9821342	1.2802758	0.1215954
C	-2.2336091	0.6301211	0.2666701
C	-0.9386372	2.6646360	0.2763640
C	-3.3870517	1.3441849	0.6161771
C	-2.1006533	3.3938278	0.6081302
H	-0.0134688	3.2221607	0.1391104
C	-3.3226131	2.7251050	0.7960683
H	-4.3473954	0.8389163	0.7362289
H	-4.2148898	3.2915639	1.0668693
N	-0.9977343	-1.2469580	-0.4763172
C	-2.2176515	-0.8005091	-0.0596288
C	-0.8629397	-2.5072994	-0.9194660
C	-3.3153619	-1.6660980	-0.0323838
C	-1.9212352	-3.4108452	-0.9347550
H	0.1238344	-2.7972992	-1.2757939
C	-3.1661299	-2.9829982	-0.4660397
H	-4.2828758	-1.3046274	0.3155545
H	-1.7657211	-4.4246936	-1.3057111
H	-4.0181476	-3.6663966	-0.4502182
C	-2.0166939	4.8177692	0.7621472
N	-1.9233708	5.9680579	0.8861153

Co-Complex 9e

SCF energy:	–3542.455214 hartree
Zero-point correction:	+0.618080 hartree
Enthalpy correction:	+0.656713 hartree
Free energy correction:	+0.534195 hartree
Quasiharmonic free energy correction:	+0.576377 hartree

Cartesian Coordinates

Co	1.7766816	0.0052846	-0.9792910
C	2.8923337	1.6931355	-1.4662815
C	3.6201297	0.9261443	-0.5052952
C	4.1454025	1.3960761	0.8112392
C	2.4594907	3.1192153	-1.3385515
C	2.7700233	0.8947927	-2.6777442
C	3.3465324	-0.3664176	-2.4310587
C	3.5275059	-1.4808533	-3.4104573
C	2.1341879	1.3702412	-3.9434431
C	3.8071931	-0.3847243	-1.0524573
C	4.4985278	-1.5250959	-0.3757722
H	4.1391923	-2.4942390	-0.7497685
H	4.3359797	-1.5018029	0.7110108
H	5.5871071	-1.4853308	-0.5511153
H	3.6053871	2.2735068	1.1874777
H	5.2038228	1.6846262	0.6916367
H	4.0973690	0.6115692	1.5780507
H	1.4766052	3.2840885	-1.8027413
H	3.1782731	3.7892918	-1.8398899
H	2.3961792	3.4354947	-0.2884517
H	1.8882516	0.5391231	-4.6181936
H	2.8170817	2.0491096	-4.4816865
H	1.2088080	1.9281162	-3.7410062
H	2.7870576	-1.4472844	-4.2222727
H	3.4761836	-2.4679414	-2.9308148
H	4.5245428	-1.4036472	-3.8772220
C	1.2874432	0.0829872	0.8778180
C	1.0327275	-1.1863275	1.4440281
C	1.0648923	1.2145678	1.6577910
C	0.6808424	-1.3176976	2.7985195
C	0.6365358	1.0902222	2.9949990
H	1.1801093	2.2157942	1.2398570
C	0.4862640	-0.1830700	3.5786208
H	0.5149496	-2.3019969	3.2411773
H	0.1853609	-0.2687408	4.6238073
N	1.1595222	-1.8520795	-0.8136963
C	0.9892704	-2.2774628	0.4674135
C	0.9269640	-2.6843842	-1.8368764
C	0.6789048	-3.6182976	0.7294398
C	0.5778202	-4.0186502	-1.6430248
H	1.0139105	-2.2618092	-2.8376103
C	0.4799817	-4.4976183	-0.3330050
H	0.5795717	-3.9607958	1.7596778
H	0.3905235	-4.6620707	-2.5036169
H	0.2310293	-5.5439774	-0.1424670
O	-4.7311744	0.4508975	-1.8247195
H	-4.9690516	0.6327611	0.8176258
H	-4.1293929	3.1004319	-2.0144021
H	-4.9238977	5.0032993	-0.5923311
H	-4.8205311	-0.1102105	3.1915197
C	-4.2743113	-0.1545801	1.1121094
C	-4.1861034	-0.5804884	2.4367834
C	-3.7253233	3.2622385	-1.0148437
C	-4.1639361	4.3174634	-0.2116585
S	-3.4526055	-0.1508450	-1.4952269
C	-3.4502986	-0.7636520	0.1605032
O	-2.7130989	-1.0473485	-2.3736370
N	-2.3287008	1.2776284	-1.3199311
C	-2.7565984	2.3911129	-0.5094507
C	-3.3006912	-1.6030838	2.8152945
H	-4.0160862	-1.6670281	4.8611530
C	-3.6261371	4.5052800	1.0666909
H	-3.1423647	-3.1321713	4.3384168
C	-3.1796339	-2.0352044	4.2509947
H	-3.9668959	5.3407797	1.6823192
C	-1.0554801	0.9652937	-1.3917903
C	-2.5635092	-1.7928765	0.4973191

C	-2.5072248	-2.2082927	1.8233198
C	-2.2273749	2.5554851	0.7771469
N	0.0650754	0.6612778	-1.4501164
C	-2.6538240	3.6285081	1.5612006
H	-1.9486616	-2.2604541	-0.2725217
H	-2.2468003	-1.6404163	4.6900146
H	-1.8361155	-3.0243295	2.0977293
H	-1.4905129	1.8454611	1.1559363
H	-2.2282655	3.7728721	2.5570816
C	0.2858161	2.2727442	3.7272989
N	-0.0514326	3.2428046	4.2694784

Transition State TS3e

SCF energy:	–3542.410602 hartree
Zero-point correction:	+0.616973 hartree
Enthalpy correction:	+0.654622 hartree
Free energy correction:	+0.537163 hartree
Quasiharmonic free energy correction:	+0.567160 hartree
Imaginary Frequency	375.0 *i*cm^{-1}

Cartesian Coordinates

Co	0.2022938	1.7168226	-0.3363915
C	-0.7965948	3.0649486	-1.5697969
C	-0.9596209	3.5015471	-0.2041793
C	-2.2486956	3.7156131	0.5227294
C	-1.8700183	2.7532920	-2.5645272
C	0.6075432	3.1069947	-1.8877027
C	1.3136815	3.4615636	-0.7109613
C	2.7843645	3.7090627	-0.6023098
C	1.1845514	2.7743863	-3.2226137
C	0.3335101	3.6890373	0.3468932
C	0.6416359	4.1204995	1.7446308
H	1.5892255	3.6937226	2.1033931
H	-0.1521742	3.8187281	2.4424645
H	0.7329267	5.2186410	1.8014551
H	-3.1032057	3.2915993	-0.0184800
H	-2.4345052	4.7961991	0.6410495
H	-2.2307067	3.2718555	1.5287671
H	-1.6799298	1.7924291	-3.0671487
H	-1.9045531	3.5276196	-3.3487761
H	-2.8650630	2.7134618	-2.1021914
H	2.2753310	2.6572547	-3.1788355
H	0.9579889	3.5779301	-3.9433182
H	0.7595733	1.8379991	-3.6110658
H	3.3668226	3.0190514	-1.2304961
H	3.1422482	3.6291400	0.4330341
H	3.0175644	4.7323541	-0.9434453
C	-1.2614671	0.5261112	0.4302577
C	-0.9166099	0.2706423	1.7863321
C	-2.6124974	0.5411084	0.0629057
C	-1.9170764	0.0616068	2.7399556
C	-3.6167665	0.3225830	1.0206388
H	-2.8951276	0.7054948	-0.9759465
C	-3.2605725	0.0840471	2.3599641
H	-1.6637681	-0.1002356	3.7896038
H	-4.0433478	-0.0848557	3.1011410
N	1.2670514	0.8912244	1.1111598
C	0.5214443	0.2980478	2.0804254
C	2.6017877	0.8865443	1.2019753
C	1.1269073	-0.2547054	3.2148410
C	3.2671511	0.3520238	2.3028313
H	3.1484760	1.3168741	0.3648986
C	2.5145249	-0.2132587	3.3343404
H	0.5145221	-0.7265363	3.9837610
H	4.3565084	0.3736770	2.3378821
H	3.0049415	-0.6380572	4.2127853

O	0.1255468	-4.2138203	-0.9097015
H	1.8253316	-3.6377727	0.9876064
H	-2.6344938	-1.7850605	-2.3198722
H	-4.9531850	-2.5830606	-1.8006384
H	4.0879030	-3.0838249	1.8834201
C	2.5011749	-2.9837702	0.4342072
C	3.7696166	-2.6754584	0.9209749
C	-2.8776816	-2.2041237	-1.3409105
C	-4.1645505	-2.6573119	-1.0492340
S	0.5347310	-2.9311303	-1.4645429
C	2.1149000	-2.4641264	-0.8042220
O	0.4870468	-2.6405850	-2.8909005
N	-0.5673717	-1.7756955	-0.6363452
C	-1.8796446	-2.2831419	-0.3610459
C	4.6543683	-1.8671621	0.1854618
H	6.1051287	-1.6934421	1.7897316
C	-4.4425628	-3.2042523	0.2081497
H	6.7684505	-2.2789362	0.2538628
C	6.0403180	-1.5782169	0.6975135
H	-5.4522248	-3.5520379	0.4369686
C	-0.3017716	-0.4540798	-0.8687393
C	2.9653236	-1.6553178	-1.5619929
C	4.2301421	-1.3604552	-1.0547002
C	-2.1426854	-2.8596710	0.8826577
N	0.4827487	0.2060651	-1.5364498
C	-3.4304718	-3.3162408	1.1675811
H	2.6332465	-1.2789915	-2.5291350
H	6.3697214	-0.5626757	0.4285126
H	4.9097786	-0.7376475	-1.6425140
H	-1.3378585	-2.9374467	1.6143960
H	-3.6465146	-3.7637203	2.1403630
C	-4.9927414	0.3292968	0.6179319
N	-6.0999140	0.3402851	0.2706558

Co-Complex 10e

SCF energy:	–3542.451112 hartree
Zero-point correction:	+0.619422 hartree
Enthalpy correction:	+0.657448 hartree
Free energy correction:	+0.536671 hartree
Quasiharmonic free energy correction:	+0.575325 hartree

Cartesian Coordinates

Co	1.7473071	-0.8357417	-0.2761960
C	2.6250866	-1.2522740	1.5091499
C	3.2980707	-2.0189675	0.4767541
C	3.3717836	-3.5109870	0.4291459
C	1.9634509	-1.8089132	2.7249833
C	2.7990846	0.1466400	1.2098304
C	3.5087449	0.2305058	-0.0223418
C	3.8697551	1.4770586	-0.7584028
C	2.2650988	1.2915540	2.0081005
C	3.8326455	-1.1124647	-0.4701815
C	4.6135122	-1.4373799	-1.7040949
H	4.3275829	-0.7942706	-2.5501152
H	4.4857284	-2.4856054	-2.0080703
H	5.6915471	-1.2783911	-1.5297954
H	2.4538515	-3.9743648	0.8174765
H	4.2101482	-3.8691708	1.0497429
H	3.5335987	-3.8811397	-0.5922923
H	1.0960150	-1.2067284	3.0273490
H	2.6749011	-1.8146173	3.5690637
H	1.6244686	-2.8408678	2.5709145
H	1.9435353	2.1179136	1.3605961
H	3.0371214	1.6758060	2.6953357
H	1.4008259	0.9859869	2.6131520
H	3.3313644	2.3463219	-0.3650858

H	3.6451201	1.3923589	-1.8325048
H	4.9525476	1.6677870	-0.6641310
C	-1.1789446	-0.8939675	1.1317284
C	-0.9197897	-2.2645786	0.9098568
C	-1.7012375	-0.4682339	2.3538459
C	-1.2183166	-3.1809855	1.9328009
C	-1.9591663	-1.3907241	3.3801022
H	-1.9129740	0.5904248	2.5074047
C	-1.7199304	-2.7585233	3.1605266
H	-1.0359177	-4.2451817	1.7692599
H	-1.9271318	-3.4802078	3.9520109
N	0.6777649	-2.3129830	-0.9873131
C	-0.4449506	-2.7945330	-0.3978282
C	1.0778050	-2.8172120	-2.1704133
C	-1.1776108	-3.8212834	-1.0147687
C	0.3925192	-3.8303218	-2.8298555
H	1.9814530	-2.3803926	-2.5987686
C	-0.7622237	-4.3439560	-2.2368901
H	-2.0891373	-4.1792778	-0.5354043
H	0.7571191	-4.1971499	-3.7904172
H	-1.3379574	-5.1334221	-2.7250132
O	-0.9180625	1.6923649	-2.6390417
H	-1.7424245	3.4951674	1.1462199
H	-2.7198069	-1.1152279	-1.7576356
H	-5.0233389	-2.0957929	-1.7861031
H	-0.1631736	4.9823117	2.3624243
C	-0.7744412	3.7478404	0.7100761
C	0.1119217	4.5851124	1.3819663
C	-3.5169398	-0.6869763	-1.1456485
C	-4.8048216	-1.2252291	-1.1631564
S	-1.5676981	2.2468054	-1.4535111
C	-0.4062224	3.2359237	-0.5377989
O	-2.8383081	2.9544855	-1.5488142
N	-1.9048879	0.9552633	-0.3065573
C	-3.2421060	0.4286394	-0.3455039
C	1.3459351	4.9484793	0.8092922
H	2.3237301	5.6661913	2.6035020
C	-5.8173881	-0.6369051	-0.3986364
H	3.2893069	5.8612376	1.1135773
C	2.2711924	5.8939987	1.5275957
H	-6.8271270	-1.0532079	-0.4168880
C	-0.8552489	0.1131672	0.0714683
C	0.8084369	3.5700527	-1.1323750
C	1.6730934	4.4327219	-0.4549396
C	-4.2540525	1.0278893	0.4086941
N	0.3310611	0.2000742	-0.3624038
C	-5.5424940	0.4924017	0.3801557
H	1.0608582	3.1560439	-2.1094486
H	1.9089247	6.9322501	1.4353054
H	2.6203010	4.7172153	-0.9204848
H	-4.0275823	1.9123297	1.0056613
H	-6.3363383	0.9590146	0.9674314
C	-2.4659574	-0.9311305	4.6416729
N	-2.8670507	-0.5483612	5.6609271

Co-Complex 11e

SCF energy:	–3771.570538 hartree
Zero-point correction:	+0.684085 hartree
Enthalpy correction:	+0.726612 hartree
Free energy correction:	+0.597155 hartree
Quasiharmonic free energy correction:	+0.632298 hartree

Cartesian Coordinates

Co	0.6544413	-1.9242389	-0.2021716
C	1.4715904	-1.8044210	-2.1454782
C	2.4496990	-2.2242787	-1.1867691

C	3.7887944	-1.6072742	-0.9556888
C	1.5495177	-0.6152264	-3.0409905
C	0.3916747	-2.7365004	-2.0899225
C	0.7829687	-3.8277664	-1.2116183
C	-0.0433086	-5.0437159	-0.9614113
C	-0.8732357	-2.6592045	-2.8808554
C	2.0247077	-3.5012564	-0.6317409
C	2.8154860	-4.3110818	0.3453708
H	2.1885325	-5.0413133	0.8755296
H	3.3058301	-3.6738629	1.0956083
H	3.6106533	-4.8736079	-0.1726793
H	3.8093889	-0.5476659	-1.2349804
H	4.5429361	-2.1302814	-1.5692183
H	4.1055024	-1.6931718	0.0923829
H	0.6024567	-0.0592913	-3.0720634
H	1.7751527	-0.9531297	-4.0667684
H	2.3488784	0.0708053	-2.7362984
H	-1.7245964	-3.0508131	-2.3055130
H	-0.7938915	-3.2563474	-3.8053355
H	-1.0904148	-1.6203467	-3.1639785
H	-1.0929421	-4.7785980	-0.7764923
H	0.3225528	-5.6238755	-0.1035666
H	-0.0117610	-5.6988088	-1.8485335
C	1.7338045	1.1867435	-0.0318278
C	2.4585504	0.6086314	1.0299246
C	2.3573969	2.1233455	-0.8647811
C	3.7964698	1.0023614	1.2297880
C	3.7007038	2.4758115	-0.6800141
H	1.7782332	2.5818868	-1.6665265
C	4.4233246	1.9111570	0.3864327
H	4.3661918	0.5624363	2.0505408
H	5.4666534	2.1873823	0.5453356
N	1.2917743	-1.5034181	1.6283924
C	1.8976696	-0.3541087	2.0157100
C	0.9590266	-2.4173822	2.5622671
C	2.0937444	-0.0864280	3.3830646
C	1.1577862	-2.2243039	3.9236797
H	0.4995870	-3.3321617	2.1886695
C	1.7177756	-1.0171645	4.3462121
H	2.5390832	0.8656171	3.6725338
H	0.8685036	-3.0034041	4.6307084
H	1.8672773	-0.8077027	5.4078388
O	-2.1273145	3.3471777	-1.9522226
H	-3.5715031	2.8614603	0.3088617
H	-0.7345855	1.6971413	2.0633189
H	-0.6440293	3.4751402	3.8127725
H	-5.5254100	1.7549203	1.3823245
C	-3.8511926	1.8433250	0.0336110
C	-4.9496063	1.2188501	0.6232975
C	-0.6026257	2.7435119	1.7813880
C	-0.5519394	3.7411856	2.7568818
S	-1.7397114	1.9596698	-1.7229109
C	-3.1194261	1.1484458	-0.9353859
O	-1.2491057	1.1122107	-2.8094272
N	-0.4815527	2.0344334	-0.5520847
C	-0.4784642	3.0797592	0.4248855
C	-5.3485956	-0.0731894	0.2348046
H	-6.6717622	-0.4840625	1.9076898
C	-0.3974802	5.0786743	2.3802646
H	-7.4886138	-0.3193597	0.3464708
C	-6.5778803	-0.7079835	0.8335796
H	-0.3646288	5.8618499	3.1410073
C	0.2987547	0.8594620	-0.2936210
C	-3.4817905	-0.1368685	-1.3353950
C	-4.6003438	-0.7352263	-0.7535025
C	-0.3111770	4.4183441	0.0467209
N	-0.2127231	-0.2892152	-0.3542799
C	-0.2828289	5.4120178	1.0262490
H	-2.8945377	-0.6494695	-2.0953491

H	-6.5859422	-1.8006209	0.6972684
H	-4.9011164	-1.7336475	-1.0812090
H	-0.2194718	4.6681496	-1.0093842
H	-0.1582853	6.4556076	0.7286598
O	-1.1461135	-2.6995365	0.5502015
C	-2.1557873	-2.2630461	1.0856389
O	-3.2557475	-3.0020441	0.9923986
H	-4.0115821	-2.5469893	1.3949314
C	-2.2181953	-0.9723941	1.8419823
H	-1.9724324	-0.1766027	1.1248657
H	-1.4370760	-0.9709963	2.6151732
H	-3.1976542	-0.7823556	2.2977764
C	4.3260376	3.4145679	-1.5675992
N	4.8304779	4.1695710	-2.2898854

Co-Complex 12e

SCF energy:	–2665.017148 hartree
Zero-point correction:	+0.444083 hartree
Enthalpy correction:	+0.473304 hartree
Free energy correction:	+0.376858 hartree
Quasiharmonic free energy correction:	+0.397034 hartree

Cartesian Coordinates

Co	-0.8026248	0.1616241	0.3930435
O	0.1283234	-0.3106069	2.1035892
O	-1.1509552	1.4093710	1.9032528
N	0.6364510	1.3834829	-0.2477835
C	1.8791920	1.0758084	-0.6897505
C	-2.6024746	0.3583861	-0.5948945
C	-1.6219097	-0.1049501	-1.5207066
C	-1.2054983	0.5786353	-2.7804294
C	-3.3941909	1.6218184	-0.6700201
C	-2.7114303	-0.6209002	0.4718454
C	-1.7920765	-1.6716067	0.1911740
C	-1.5197428	-2.8733019	1.0306965
C	-3.6356210	-0.5192166	1.6394684
C	-1.0921238	-1.3502976	-1.0237343
C	-0.1532630	-2.2694562	-1.7326055
C	-0.3890938	0.7150322	2.6500526
C	-0.0891782	1.1036074	4.0606930
H	-0.9210956	1.6809828	4.4849511
H	0.8103792	1.7415626	4.0659408
H	0.1202638	0.2123906	4.6672866
H	0.6063385	-2.6847524	-1.0570276
H	0.3519864	-1.7725714	-2.5677316
H	-0.7201874	-3.1193671	-2.1495445
H	-1.3485118	1.6659165	-2.7227420
H	-1.8214943	0.2092492	-3.6178125
H	-0.1563659	0.3846422	-3.0375918
H	-3.4314038	2.1262452	0.3071107
H	-4.4338663	1.3981680	-0.9635016
H	-2.9846726	2.3207512	-1.4110892
H	-3.3062330	-1.1517648	2.4748126
H	-4.6470963	-0.8504350	1.3493897
H	-3.7105088	0.5155501	2.0002409
H	-1.8587189	-2.7374242	2.0653514
H	-0.4466971	-3.1093699	1.0487779
H	-2.0471226	-3.7467965	0.6107054
C	0.2282042	2.6702608	-0.2909419
C	1.0190311	3.7018938	-0.7805175
C	2.3014910	3.3993139	-1.2378707
C	2.7288491	2.0761614	-1.1853720
C	2.3874863	-0.3244666	-0.6630821
H	2.9604956	4.1791139	-1.6255479
H	3.7286589	1.8013806	-1.5222252
H	-0.7704677	2.8678054	0.0964088

H	0.6300306	4.7212050	-0.7929657
C	2.5270894	-1.0354247	0.5342822
C	2.8383048	-0.9267204	-1.8650190
C	3.4153136	-2.2031551	-1.8555834
C	3.0937120	-2.3086857	0.5486962
C	3.5443669	-2.8998796	-0.6452867
H	2.1828099	-0.5920014	1.4660998
H	3.1986773	-2.8497570	1.4906317
H	3.7458940	-2.6580992	-2.7900363
C	2.6182068	-0.2676877	-3.1209187
N	2.3717099	0.2621419	-4.1238628
C	4.1196555	-4.2148823	-0.6305977
N	4.5738538	-5.2821336	-0.6149181

4-(2-Pyridinyl)-benzonitrile (1e)

SCF energy:	–571.544884 hartree
Zero-point correction:	+0.168630 hartree
Enthalpy correction:	+0.178800 hartree
Free energy correction:	+0.128638 hartree
Quasiharmonic free energy correction:	+0.136612 hartree

Cartesian Coordinates

C	2.9881043	1.1358369	-0.0423854
C	1.5976069	1.1859284	-0.0393683
C	3.6489258	-0.1049700	0.0031324
C	2.8893061	-1.2891228	0.0494698
C	1.5009213	-1.2266379	0.0510379
C	0.8284376	0.0090687	0.0088809
C	-0.6609794	0.0259500	0.0147135
C	-1.4030917	1.2212629	0.0448267
N	-1.2725406	-1.1702627	-0.0070689
C	-2.6010684	-1.2251297	-0.0041749
C	-3.4182286	-0.0898958	0.0209645
C	-2.7956632	1.1586964	0.0471975
H	1.1155441	2.1631513	-0.0805491
H	3.5724096	2.0568696	-0.0815081
H	3.3984131	-2.2541370	0.0840074
H	0.8988597	-2.1346173	0.0845468
H	-3.3867140	2.0776537	0.0709004
H	-0.9061382	2.1908265	0.0706853
H	-3.0476851	-2.2264428	-0.0229251
H	-4.5058732	-0.1870385	0.0215531
C	5.0824017	-0.1608238	0.0010387
N	6.2429723	-0.2050661	-0.0005250

4-(2-Pyridinyl)-1,3-benzenedicarbonitrile (3e)

SCF energy:	–663.784113 hartree
Zero-point correction:	+0.167025 hartree
Enthalpy correction:	+0.178994 hartree
Free energy correction:	+0.125645 hartree
Quasiharmonic free energy correction:	+0.130429 hartree

Cartesian Coordinates

C	-2.7028631	-1.8226720	-0.1875691
C	-1.3172538	-1.7080415	-0.1587807
C	-3.5016880	-0.6694255	-0.0909984
C	-2.8889214	0.5852639	0.0365707
C	-1.4906452	0.6971279	0.0622103
C	-0.6804500	-0.4626118	-0.0387264
C	0.8017744	-0.3722343	-0.0340922
C	1.5978253	-1.3711449	0.5541144
N	1.3333798	0.7083201	-0.6190813
C	2.6568489	0.8420347	-0.6475552
C	3.5321323	-0.1011739	-0.0972574

C	2.9848113	-1.2285548	0.5176041
H	-0.7080033	-2.6074811	-0.2621869
H	-3.1751722	-2.8005850	-0.2944755
H	-3.5003073	1.4841012	0.1244215
C	-0.9490654	2.0168923	0.2501625
H	3.6290940	-1.9845864	0.9730714
H	1.1402983	-2.2273373	1.0523733
H	3.0443511	1.7452547	-1.1323278
H	4.6123177	0.0517802	-0.1457000
N	-0.6358626	3.1161560	0.4509061
C	-4.9327406	-0.7738699	-0.1210938
N	-6.0896603	-0.8653427	-0.1454895

12 References

1 Weigend, F.; Ahlrichs, R. *Phys. Chem. Chem. Phys.* **2005**, *7*, 3297–3305.

2 Becke, A. D. *J. Chem. Phys.* **1993**, *98*, 5648–5652.

3 Lee, C.; Yang, W.; Parr, R. G. *Phys. Rev. B* **1988**, *37*, 785–789.

4 Grimme, S.; Antony, J.; Ehrlich, S.; Krieg, H. *J. Chem. Phys.* **2010**, *132*, 154104.

5 Grimme, S.; Ehrlich, S.; Goerigk, L. *J. Comput. Chem.* **2011**, *32*, 1456–1465.

6 Zhao, Y.; Truhlar, D. G. *J. Chem. Phys.* **2006**, *125*, 194101.

7 Eichkorn, K.; Weigend, F.; Treutler, O.; Ahlrichs, R. *Theor. Chem. Acc.* **1997**, *97*, 119–124.

8 Deglmann, P.; May, K.; Furche, F.; Ahlrichs, R. *Chem. Phys. Lett.* **2004**, *384*, 103–107.

9 Ribeiro, R. F.; Marenich, A. V.; Cramer, C. J.; Truhlar, D. G. *J. Phys. Chem. B* **2011**, *115*, 14556–14562.

10 Klamt, A.; Schüürmann, G. *J. Chem. Soc., Perkin Trans. 2* **1993**, 799–805.

11 Wuttke, A.; Mata, R. A. *J. Comput. Chem.* **2017**, *38*, 15–23.

12 Werner, H.-J.; Knowles, P. J.; Knizia, G.; Manby, F. R.; Schütz, M. *WIREs Comput. Mol. Sci.* **2012**, *2*, 242–253.

13 MOLPRO, version 2015.1, a package of *ab initio* programs, Werner, H.-J.; Knowles, P. J.; Knizia, G.; Manby, F. R.; Schütz, M.; Celani, P.; Györffy, W.; Kats, D.; Korona, T.; Lindh, R.; Mitrushenkov, A.; Rauhut, G.; Shamasundar, K. R.; Adler, T. B.; Amos, R. D.; Bernhardsson, A.; Berning, A.; Cooper, D. L.; Deegan, M. J. O.; Dobbyn, A. J.; Eckert, F.; Goll, E.; Hampel, C. ; Hesselmann, A.; Hetzer, G.; Hrenar, T.; Jansen, G.; Köppl, C.; Liu, Y.; Lloyd, A. W.; Mata, R. A.; May, A. J.; McNicholas, S. J.; Meyer, W.; Mura, M. E.; Nicklass, A.; O'Neill, D. P.; Palmieri, P.; Peng, D.; Pflüger, K.; Pitzer, R.; Reiher, M.; Shiozaki, T.; Stoll, H. ; Stone, A. J.; Tarroni, R.; Thorsteinsson, T.; Wang, M.; see http://www.molpro.net.

14 Schneider, W. B.; Bistoni, G.; Sparta, M.; Saitow, M.; Riplinger, C.; Auer, A. A.; Neese, F. *J. Chem. Theory Comput.* **2016**, *12*, 4778–4792.

15 Riplinger, C.; Sandhoefer, B.; Hansen, A.; Neese, F. *J. Chem. Phys.* **2013**, *139*, 134101.

16 Riplinger, C.; Neese, F. *J. Chem. Phys.* **2013**, *138*, 034106.

17 Liakos, D. G.; Neese, F. *J. Chem. Theory Comput.* **2015**, *11*, 4054–4063.

18 Neese, F. *Wiley Interdiscip. Rev. Comput. Mol. Sci.* **2012**, *2*, 73–78.

19 TURBOMOLE V7.1, 2015, a development of University of Karlsruhe and Forschungszentrum Karlsruhe GmbH, 1989–2007, TURBOMOLE GmbH, since 2007; available from http://www.turbomole.com/.

20 Furche, F.; Ahlrichs, R.; Hättig, C.; Klopper, W.; Sierka, M.; Weigend, F. *Wiley Interdiscip. Rev.: Comput. Mol. Sci.* **2014**, *4*, 91–100.

21 Johnson, E. R.; Keinan, S.; Mori-Sánchez, P.; Contreras-García, J.; Cohen, A. J.; Yang, W. *J. Am. Chem. Soc.* **2010**, *132*, 6498–6506.

22 Contreras-García, J.; Johnson, E. R.; Keinan, S.; Chaudret, R.; Piquemal, J.-P.; Beratan, D. N.; Yang, W. *J. Chem. Theory Comput.* **2011**, *7*, 625–632.
